# Posicionamento Brasileiro sobre o Uso da Multimodalidade de Imagens na Cardio-Oncologia – 2021

**DOI:** 10.36660/abc.20200266

**Published:** 2021-10-06

**Authors:** Marcelo Dantas Tavares de Melo, Marcelo Goulart Paiva, Maria Verônica Câmara Santos, Carlos Eduardo Rochitte, Valéria de Melo Moreira, Mohamed Hassan Saleh, Simone Cristina Soares Brandão, Claudia Cosentino Gallafrio, Daniel Goldwasser, Eliza de Almeida Gripp, Rafael Bonafim Piveta, Tonnison Oliveira Silva, Thais Harada Campos Espirito Santo, Waldinai Pereira Ferreira, Vera Maria Cury Salemi, Sanderson A. Cauduro, Silvio Henrique Barberato, Heloísa M. Christovam Lopes, José Luiz Barros Pena, Heron Rhydan Saad Rached, Marcelo Haertel Miglioranza, Aurélio Carvalho Pinheiro, Bárbara Athayde Linhares Martins Vrandecic, Cecilia Beatriz Bittencourt Viana Cruz, César Higa Nomura, Fernanda Mello Erthal Cerbino, Isabela Bispo Santos da Silva Costa, Otavio Rizzi Coelho, Adriano Camargo de Castro Carneiro, Ursula Maria Moreira Costa Burgos, Juliano Lara Fernandes, Marly Uellendahl, Eveline Barros Calado, Tiago Senra, Bruna Leal Assunção, Claudia Maria Vilas Freire, Cristiane Nunes Martins, Karen Saori Shiraishi Sawamura, Márcio Miranda Brito, Maria Fernanda Silva Jardim, Renata Junqueira Moll Bernardes, Tereza Cristina Diógenes, Lucas de Oliveira Vieira, Claudio Tinoco Mesquita, Rafael Willain Lopes, Elry Medeiros Vieira Segundo, Letícia Rigo, Valeska Leite Siqueira Marin, Marcelo José Santos, Gabriel Blacher Grossman, Priscila Cestari Quagliato, Monica Luiza de Alcantara, José Aldo Ribeiro Teodoro, Ana Cristina Lopes Albricker, Fanilda Souto Barros, Salomon Israel do Amaral, Carmen Lúcia Lascasas Porto, Marcio Vinícius Lins Barros, Simone Nascimento dos Santos, Armando Luís Cantisano, Ana Cláudia Gomes Pereira Petisco, José Eduardo Martins Barbosa, Orlando Carlos Glória Veloso, Salvador Spina, Ricardo Pignatelli, Ludhmilla Abrahão Hajjar, Roberto Kalil, Marcelo Antônio Cartaxo Queiroga Lopes, Marcelo Luiz Campos Vieira, André Luiz Cerqueira Almeida

**Affiliations:** 1 Universidade Federal da Paraíba João PessoaPB Brasil Universidade Federal da Paraíba, João Pessoa, PB – Brasil; 2 Hospital 9 de Julho Cardiologia São PauloSão Paulo Brasil Hospital 9 de Julho, Cardiologia, São Paulo, São Paulo – Brasil; 3 Sociedade Brasileira de Oncologia Pediátrica São PauloSP Brasil Sociedade Brasileira de Oncologia Pediátrica, São Paulo, SP – Brasil; 4 Universidade de São Paulo Hospital das Clínicas da Faculdade de Medicina Instituto do Coração (Incor) São PauloSP Brasil Instituto do Coração (Incor) do Hospital das Clínicas da Faculdade de Medicina da Universidade de São Paulo (HCFMUSP), São Paulo, SP – Brasil; 5 Hospital do Coração São PauloSP Brasil Hospital do Coração (HCOR), São Paulo, SP – Brasil; 6 Instituto Dante Pazzanese de Cardiologia São PauloSP Brasil Instituto Dante Pazzanese de Cardiologia, São Paulo, SP – Brasil; 7 Universidade Federal de Pernambuco Hospital das Clínicas RecifePE Brasil Hospital das Clínicas, Universidade Federal de Pernambuco, Recife, PE - Brasil; 8 Clínica Diagson João PessoaPB Brasil Clínica Diagson, João Pessoa, PB – Brasil; 9 Instituto de Oncologia Pediátrica São PauloSP Brasil Instituto de Oncologia Pediátrica, São Paulo, SP – Brasil; 10 Hospital Federal de Ipanema Rio de JaneiroRJ Brasil Hospital Federal de Ipanema, Rio de Janeiro, RJ – Brasil; 11 Hospital Copa D'Or Rio de JaneiroRJ Brasil Hospital Copa D'Or, Rio de Janeiro, RJ – Brasil; 12 Casa de Saúde São José Rio de JaneiroRJ Brasil Casa de Saúde São José, Rio de Janeiro, RJ – Brasil; 13 Hospital Pró-Cardíaco Rio de JaneiroRJ Brasil Hospital Pró-Cardíaco, Rio de Janeiro, RJ – Brasil; 14 Hospital Universitário Antônio Pedro Rio de JaneiroRJ Brasil Hospital Universitário Antônio Pedro, Rio de Janeiro, RJ – Brasil; 15 Hospital Israelita Albert Einstein São PauloSP Brasil Hospital Israelita Albert Einstein, São Paulo, SP – Brasil; 16 Hospital Cardio Pulmonar Centro de Estudos em Cardiologia SalvadorBA Brasil Hospital Cardio Pulmonar – Centro de Estudos em Cardiologia, Salvador, BA – Brasil; 17 Escola Bahiana de Medicina e Saúde Pública SalvadorBA Brasil Escola Bahiana de Medicina e Saúde Pública, Salvador, BA – Brasil; 18 Hospital Ana Nery SalvadorBA Brasil Hospital Ana Nery, Salvador, BA – Brasil; 19 Diagnoson/Fleury SalvadorBA Brasil Diagnoson/Fleury, Salvador, BA – Brasil; 20 ACCamargoCancer Center IMAGE São PauloSP Brasil ACCamargoCancer Center – IMAGE, São Paulo, SP – Brasil; 21 Hospital Erasto Gaertner CuritibaPR Brasil Hospital Erasto Gaertner, Curitiba, PR – Brasil; 22 CardioEco Centro de Diagnóstico Cardiovascular CuritibaPR Brasil CardioEco Centro de Diagnóstico Cardiovascular, Curitiba, PR – Brasil; 23 Quanta Diagnóstico CuritibaPR Brasil Quanta Diagnóstico, Curitiba, PR – Brasil; 24 Hospital de Amor São PauloSP Brasil Hospital de Amor, Barretos, São Paulo, SP – Brasil; 25 Faculdade de Ciências Médicas de Minas Gerais Belo HorizonteMG Brasil Faculdade de Ciências Médicas de Minas Gerais, Belo Horizonte, MG – Brasil; 26 Hospital Leforte Liberdade São PauloSP Brasil Hospital Leforte Liberdade, São Paulo, SP – Brasil; 27 Instituto de Cardiologia do Rio Grande do Sul Laboratório de Pesquisa e Inovação em Imagem Cardiovascular Porto AlegreRS Brasil Instituto de Cardiologia do Rio Grande do Sul – Laboratório de Pesquisa e Inovação em Imagem Cardiovascular, Porto Alegre, RS – Brasil; 28 Hospital Mãe de Deus Porto AlegreRS Brasil Hospital Mãe de Deus, Porto Alegre, RS – Brasil; 29 Hospital Adventista de Manaus ManausAM Brasil Hospital Adventista de Manaus, Manaus, AM – Brasil; 30 Biocor Instituto Nova LimaMG Brasil Biocor Instituto, Nova Lima, MG – Brasil; 31 Hospital Sírio-Libanês São PauloSP Brasil Hospital Sírio-Libanês, São Paulo, SP – Brasil; 32 Clínica de Diagnóstico por Imagem Rio de JaneiroRJ Brasil Clínica de Diagnóstico por Imagem, Rio de Janeiro, RJ – Brasil; 33 Diagnósticos da América AS Rio de JaneiroRJ Brasil Diagnósticos da América AS, Rio de Janeiro, RJ – Brasil; 34 Universidade de São Paulo Instituto do Câncer do Estado de São Paulo São PauloSP Brasil Universidade de São Paulo Instituto do Câncer do Estado de São Paulo, São Paulo, SP – Brasil; 35 Universidade Estadual de Campinas CampinasSP Brasil Universidade Estadual de Campinas (UNICAMP), Campinas, SP – Brasil; 36 Universidade Tiradentes AracajuSE Brasil Universidade Tiradentes, Aracaju, SE – Brasil; 37 Radiologia Clínica de Campinas CampinasSP Brasil Radiologia Clínica de Campinas, Campinas, SP – Brasil; 38 Instituto de Ensino e Pesquisa José Michel Kalaf CampinasSP Brasil Instituto de Ensino e Pesquisa José Michel Kalaf, Campinas, SP – Brasil; 39 Universidade Federal de São Paulo São PauloSP Brasil Universidade Federal de São Paulo (UNIFESP), São Paulo, SP – Brasil; 40 Universidade Federal de Pernambuco Hospital das Clínicas RecifePE Brasil Hospital das Clínicas da Universidade Federal de Pernambuco, Recife, PE – Brasil; 41 Universidade Federal de Minas Gerais Belo HorizonteMG Brasil Universidade Federal de Minas Gerais (UFMG), Belo Horizonte, MG – Brasil; 42 ECOCENTER Belo HorizonteMG Brasil ECOCENTER, Belo Horizonte, MG – Brasil; 43 Universidade de São Paulo Instituto da Criança São PauloSP Brasil Instituto da Criança da Universidade de São Paulo (USP), São Paulo, SP – Brasil; 44 Universidade Federal do Tocantins Campus de Araguaina AraguainaTO Brasil Universidade Federal do Tocantins – Campus de Araguaina, Araguaina, TO – Brasil; 45 Hospital Municipal de Araguaina AraguainaTO Brasil Hospital Municipal de Araguaina, Araguaina, TO – Brasil; 46 Hospital Samaritano de São Paulo São PauloSP Brasil Hospital Samaritano de São Paulo, São Paulo, SP – Brasil; 47 Instituto D'Or de Pesquisa e Ensino Rio de JaneiroRJ Brasil Instituto D'Or de Pesquisa e Ensino, Rio de Janeiro, RJ – Brasil; 48 Hospital Infantil Albert Sabin FortalezaCE Brasil Hospital Infantil Albert Sabin, Fortaleza, CE – Brasil; 49 Hospital São Rafael SalvadorBA Brasil Hospital São Rafael, Salvador, BA – Brasil; 50 Rede D'Or SalvadorBA Brasil Rede D'Or, Salvador, BA – Brasil; 51 Universidade Federal Fluminense Rio de JaneiroRJ Brasil Universidade Federal Fluminense (UFF), Rio de Janeiro, RJ - Brasil; 52 Hospital Vitória Rio de JaneiroRJ Brasil Hospital Vitória, Rio de Janeiro, RJ – Brasil; 53 Hospital Beneficência Portuguesa São PauloSP Brasil Hospital Beneficência Portuguesa, São Paulo, SP – Brasil; 54 Hospital Samaritano São PauloSP Brasil Hospital Samaritano, São Paulo, SP – Brasil; 55 Santa Casa de Misericórdia São PauloSP Brasil Santa Casa de Misericórdia, São Paulo, SP – Brasil; 56 Hospital de Câncer de Barretos BarretosSP Brasil Hospital de Câncer de Barretos, Barretos, SP – Brasil; 57 Clínica Cardionuclear Porto AlegreRS Brasil Clínica Cardionuclear, Porto Alegre, RS – Brasil; 58 Hospital Moinhos de Vento Porto AlegreRS Brasil Hospital Moinhos de Vento, Porto Alegre, RS – Brasil; 59 Americas Medical City Rio de JaneiroRJ Brasil Americas Medical City, Rio de Janeiro, Rio de Janeiro, RJ – Brasil; 60 Americas Serviços Médicos Rio de JaneiroRJ Brasil Americas Serviços Médicos, Rio de Janeiro, RJ – Brasil; 61 Rede D'Or Rio de JaneiroRJ Brasil Rede D'Or, Rio de Janeiro, RJ – Brasil; 62 Prenoto Medicina Diagnóstica Ribeirão PretoSP Brasil Prenoto Medicina Diagnóstica, Ribeirão Preto, SP – Brasil; 63 Centro Universitário Unihorizontes Belo HorizonteMG Brasil Centro Universitário Unihorizontes, Belo Horizonte, MG – Brasil; 64 Angiolab Vitória VitóriaES Brasil Angiolab Vitória – Diagnóstico Vascular, Vitória, ES – Brasil; 65 Casa de Saúde Nossa Senhora do Carmo Rio de JaneiroRJ Brasil Casa de Saúde Nossa Senhora do Carmo, Rio de Janeiro, RJ – Brasil; 66 Universidade do Estado do Rio de Janeiro Faculdade de Ciências Médicas Rio de JaneiroRJ Brasil Universidade do Estado do Rio de Janeiro Faculdade de Ciências Médicas, Rio de Janeiro, RJ – Brasil; 67 Mater Dei Rede de Saúde Belo HorizonteMG Brasil Mater Dei Rede de Saúde, Belo Horizonte, MG – Brasil; 68 Hospital Vera Cruz Belo HorizonteMG Brasil Hospital Vera Cruz, Belo Horizonte, MG – Brasil; 69 Hospital Brasília – Ecocardiografia BrasíliaDF Brasil Hospital Brasília – Ecocardiografia, Brasília, DF – Brasil; 70 Eccos Diagnóstico Cardiovascular Avançado BrasíliaDF Brasil Eccos Diagnóstico Cardiovascular Avançado, Brasília, DF – Brasil; 71 Hospital Barra D'Or Rio de JaneiroRJ Brasil Hospital Barra D'Or, Rio de Janeiro, RJ – Brasil; 72 Rede UHG Rio de JaneiroRJ Brasil Rede UHG, Rio de Janeiro, RJ – Brasil; 73 Hospital Aeronáutico Central Buenos Aires Argentina Hospital Aeronáutico Central, Buenos Aires – Argentina; 74 Texas Children's Hospital HoustonTexas EUA Texas Children's Hospital, Houston, Texas – EUA; 75 Baylor College of Medicine HoustonTexas EUA Baylor College of Medicine, Houston, Texas – EUA; 76 Hospital Alberto Urquiza Wanderley João PessoaPB Brasil Hospital Alberto Urquiza Wanderley – Hemodinâmica e Cardiologia Intervencionista, João Pessoa, PB – Brasil; 77 Hospital Metropolitano Dom José Maria Pires João PessoaPB Brasil Hospital Metropolitano Dom José Maria Pires, João Pessoa, PB – Brasil; 78 Sociedade Brasileira de Cardiologia Rio de JaneiroRJ Brasil Sociedade Brasileira de Cardiologia, Rio de Janeiro, RJ – Brasil; 79 Santa Casa de Misericórdia de Feira de Santana Feira de SantanaBA Brasil Santa Casa de Misericórdia de Feira de Santana – Cardiologia, Feira de Santana, BA – Brasil; 80 Sociedade Brasileira de Cardiologia Departamento de Imagem Cardiovascular São PauloSP Brasil Departamento de Imagem Cardiovascular da Sociedade Brasileira de Cardiologia, São Paulo, SP – Brasil

**Table:** 

Declaração de potencial conflito de interesses dos autores/colaboradores do Posicionamento Brasileiro sobre o Uso da Multimodalidade de Imagens na Cardio-Oncologia – 2021							
Se nos últimos 3 anos o autor/colaborador do documento:							
Nomes Integrantes do Posicionamento	Participou de estudos clínicos e/ou experimentais subvencionados pela indústria farmacêutica ou de equipamentos relacionados ao posicionamento em questão	Foi palestrante em eventos ou atividades patrocinadas pela indústria relacionados ao posicionamento em questão	Foi (é) membro do conselho consultivo ou diretivo da indústria farmacêutica ou de equipamentos	Participou de comitês normativos de estudos científicos patrocinados pela indústria	Recebeu auxílio pessoal ou institucional da indústria	Elaborou textos científicos em periódicos patrocinados pela indústria	Tem ações da indústria
Adriano Camargo de Castro Carneiro	Não	Não	Não	Não	Não	Não	Não
Ana Cláudia Gomes Pereira Petisco	Não	Não	Não	Não	Não	Não	Não
Ana Cristina Lopes Albricker	Não	Não	Não	Não	Não	Não	Não
André Luiz Cerqueira de Almeida	Não	Não	Não	Não	Não	Não	Não
Armando Luís Cantisano	Não	Não	Não	Não	Não	Não	Não
Aurélio Carvalho Pinheiro	Não	Não	Não	Não	Não	Não	Não
Bárbara Arhayde Lihares Martins Vrandecic	Não	Não	Não	Não	Não	Não	Não
Bruna Leal Assunção	Não	Não	Não	Não	Não	Não	Não
Carlos Eduardo Rochitte	Não	Não	Não	Não	Não	Não	Não
Carmen Lucia Lascasas Porto	Não	Não	Não	Não	Não	Não	Não
Cecilia Beatriz Bittencourt Viana Cruz	Não	Não	Não	Não	Não	Não	Não
César Higa Nomura	Não	Não	Não	Não	Não	Não	Não
Cláudia Cosentino Gallafrio	Não	Não	Não	Não	Não	Não	Não
Cláudia Maria Vilas Freire	Não	Não	Não	Não	Não	Não	Não
Claudio Tinoco Mesquita	Não	Não	Não	Não	Não	Não	Não
Cristiane Nunes Martins	Não	Não	Não	Não	Não	Não	Não
Daniel Goldwasser	Não	Não	Não	Não	Não	Não	Não
Eliza de Almeida Gripp	Não	Não	Não	Não	Não	Não	Não
Elry Medeiros Vieira Segundo Neto	Não	Não	Não	Não	Não	Não	Não
Eveline Barros Calado	Não	Não	Não	Não	Não	Não	Não
Fanilda Souto Barros	Não	Não	Não	Não	Não	Não	Não
Fernanda Mello Erthal Cerbino	Não	Não	Não	Não	Não	Não	Não
Gabriel Blacher Grossman	Não	Não	Não	Não	Não	Não	Não
Heloísa Helena M. Christovam Lopes	Não	Não	Não	Não	Não	Não	Não
Heron Rhydan Saad Rached	Não	Não	Não	Não	Não	Não	Não
Isabela Bispo Santos da Silva Costa	Não	Não	Não	Não	Não	Não	Não
José Aldo Ribeiro Teodoro	Não	Não	Não	Não	Não	Não	Não
José Eduardo Martins Barbosa	Não	Não	Não	Não	Não	Não	Não
José Luiz Barros Pena	Não	Não	Não	Não	Não	Não	Não
Juliano Lara Fernandes	Não	Não	Não	Não	Não	Não	Não
Karen Saori Shiraishi Sawamura	Não	Não	Não	Não	Não	Não	Não
Letícia Rigo	Não	Não	Não	Não	Não	Não	Não
Lucas de Oliveira Vieira	Não	Não	Não	Não	Não	Não	Não
Ludhmila Abrahão Hajjar	Não	Não	Não	Não	Não	Não	Não
Marcelo Antônio Cartaxo Queiroga Lopes	Não	Não	Não	Não	Não	Não	Não
Marcelo Dantas Tavares de Melo	Não	Não	Não	Não	Não	Não	Não
Marcelo Goulart Paiva	Não	Não	Não	Não	Não	Não	Não
Marcelo Haertel Miglioranza	Não	Não	Não	Não	Não	Não	Não
Marcelo Luiz Campos Vieira	Não	Não	Não	Não	Não	Não	Não
Marcelo Santos	Não	Não	Não	Não	Não	Não	Não
Márcio Miranda Brito	Não	Não	Não	Não	Não	Não	Não
Márcio Vinícius Lins Barros	Não	Não	Não	Não	Não	Não	Não
Maria Fernanda Silva Jardim	Não	Não	Não	Não	Não	Não	Não
Maria Verônica Câmara dos Santos	Não	Não	Não	Não	Não	Não	Não
Marly Uellendahl	Não	Não	Não	Não	Não	Não	Não
Mohamed Hassan Saleh	Não	Não	Não	Não	Não	Não	Não
Mônica Luiza de Alcantara	Não	Não	Não	Não	Não	Não	Não
Orlando Carlos Glória Veloso	Não	Não	Não	Não	Não	Não	Não
Otávio Rizzi Coelho-Filho	Não	Não	Não	Não	Não	Não	Não
Priscila Cestari Quagliato	Não	Não	Não	Não	Não	Não	Não
Rafael Bonafim Piveta	Não	Não	Não	Não	Não	Não	Não
Rafael Willain Lopes	Não	Não	Não	Não	Não	Não	Não
Renata Junqueira Moll Bernardes	Não	Não	Não	Não	Não	Não	Não
Ricardo Pignatelli	Não	Não	Não	Não	Não	Não	Não
Roberto Kalil Filho	Não	Não	Não	Não	Não	Não	Não
Salomon Israel do Amaral	Não	Não	Não	Não	Não	Não	Não
Salvador Spina	Não	Não	Não	Não	Não	Não	Não
Sanderson A. Cauduro	Não	Não	Não	Não	Não	Não	Não
Silvio Henrique Barberato	Não	Não	Não	Não	Não	Não	Não
Simone Cristina Soares Brandão	Não	Não	Não	Não	Não	Não	Não
Simone Nascimento dos Santos	Não	Não	Não	Não	Não	Não	Não
Tereza Cristina Diógenes	Não	Não	Não	Não	Não	Não	Não
Thais Harada Campos Espirito Santo	Não	Não	Não	Não	Não	Não	Não
Tiago Senra	Não	Não	Não	Não	Não	Não	Não
Tonnison de Oliveira Silva	Não	Não	Não	Não	Não	Não	Não
Ursula Maria Moreira Costa Burgos	Não	Não	Não	Não	Não	Não	Não
Valéria de Melo Moreira	Não	Não	Não	Não	Não	Não	Não
Valeska Leite	Não	Não	Não	Não	Não	Não	Não
Vera Maria Cury Salemi	Não	Não	Não	Não	Não	Não	Não
Waldinai P. Ferreira	Não	Não	Não	Não	Não	Não	Não

## 1. Aspectos Gerais

### 1.1. Situação Atual da Cardio-Oncologia no Brasil e no Mundo

Estima-se que a incidência de câncer no Brasil seja de 600 mil casos/ano no biênio 2018-2019. ^1^ Somente a partir de 2005, a taxa de sobrevida superou a de mortalidade global por câncer, levando a um aumento no número de sobreviventes expostos ao risco decardiotoxicidade (CTX), sendo atualmente a segunda causa de morbimortalidade nessa população. ^2^

As complicações cardiovasculares decorrentes do seu tratamento, foco deste consenso, poderão resultar em mortes prematuras, internações hospitalares custosas e afastamento do trabalho, o que leva à necessidade de diagnóstico e de intervenções precoces. ^3^

Idade (crianças e idosos), doença miocárdica ou coronariana prévia, hipertensão arterial sistêmica (HAS), diabetes melito (DM), tabagismo, consumo de álcool e sedentarismo são fatores associados ao risco aumentado de CTX. ^4^

Estudos recentes sugerem que variantes genotípicas possam modificar a suscetibilidade à CTX, inserindo o mapeamento genético em campo promissor para identificação de subgrupos de risco. ^5^

Recomenda-se que sejam considerados pacientes de alto risco para o desenvolvimento de CTX aqueles cujo tratamento inclua: ^6^

- Antraciclina em dose alta (doxorrubicina > 250mg/m² ou epirrubicina > 600mg/m²)
- Radioterapia (RT) em dose ≥ 30 Gy (envolvendo o coração) ou > 2 Gy /sessão
- Doses menores de antraciclina e RT combinadas
- Doses menores de antraciclinas ou trastuzumabe isolados, porém associadas a:
  ➢ Mais de dois fatores de risco cardiovascular (tabagismo, HAS, DM, dislipidemia, obesidade – durante ou após terapia)
  ➢ Idade ≥ 60 anos
  ➢ Cardiopatia estrutural antes ou durante o tratamento (fração de ejeção [FE]: 50 a 55%, infarto agudo do miocárdio [IAM], doença valvar moderada/importante)
  ➢ Combinação de doses baixas de antraciclina e trastuzumabe.

### 1.2. Definição de Cardiotoxicidade

A definição da CTX baseada no grau de redução da fração de ejeção do ventrículo esquerdo (FEVE) omite, entretanto, as alterações que precedem a queda da FEVE e todos os outros efeitos tóxicos que acontecem além desse parâmetro. ^6^^-^^8^ A falta de uma definição mais abrangente e, por vezes, a limitação clínica, laboratorial e de imagem em documentar alguns eventos na sua fase inicial, fazem da CTX uma condição clínica subdiagnosticada. A Sociedade Europeia de Cardiologia, no ano de 2017, revisou a definição de CTX e a estendeu para toda e qualquer alteração estrutural ou funcional do coração e circulação, seja na vigência ou no pós-tratamento imediato ou tardio do câncer, considerando como agentes agressores a quimioterapia (QT), a RT ou a própria doença. ^4^

### 1.3. Mecanismos de Cardiotoxicidade

Apesar de termos conhecimento de alguns mecanismos relacionados à CTX, ainda permanece um grande desafio a identificação do mecanismo predominante, visto que a combinação de diferentes fármacos e o protocolo de tratamento e fatores constitucionais inerentes ao próprio paciente compõem uma complexa combinação que resulta na lesãodo sistema cardiovascular ( Tabela 1 ). Na dependência da classe de quimioterápicos, o dano celular pode acontecer de forma direta ou indireta e, ainda, com potencial ou não de reversibilidade. ^9^ Ewer et al. ^10^ propuseram, em 2005, a classificação da CTX em tipos 1 e 2, que embora venha sendo alvo de muitas críticas, tem ajudado a separar em danos celulares irreversíveis (Tipo 1), atribuídos ao grupo das antraciclinas, e disfunções reversíveis (Tipo 2), atribuídas ao trastuzumabe. Com o desenvolvimento de novas terapias anticâncer, incluindo os inibidores da tirosinoquinase de Bruton, inibidores de proteasomas, inibidores de *checkpoints* , dentre outros sabidamente com potencial cardiotóxico, parece que tal proposta de classificação merece ser revisada e ampliada.

**Tabela 1:** Resumo dos principais mecanismos sugeridos de CTX por grupo de fármacos

Antraciclinas	Quebra da cadeia de dupla hélice do DNA (topoisomerase IIB)
	Estresse oxidativo (espécies reativas de oxigênio)
	Hiperpermeabilidade da membrana celular (peroxidação lipídica)
	Alterações ultraestruturais
	Vacuolização citoplasmática
	Apoptose celular
Trastuzumabe	Interrupção de sinalização receptor HER 2/ERBB2 – Neuregulina 1
	Inibe reparação celular
	Disfunção celular
Cisplatina Ciclofosfamida	Lesão endotelial direta
	Ativação e agregação plaquetária
	Trombose coronariana
5- Fluoracil	Atua na via de sinalização molecular que regula o tônus muscular liso
	Vasospasmo– vasoconstricção
Inibidores do fator de crescimento endotelial vascular (VEGF)	Inibe a atividade do óxido nítrico sintase
	Aumento na produção de endotelina
	Inibe a ativação da Rho-quinase
	Vasospasmo
Inibidores do proteasoma	Interferência na degradação de proteínas disfuncionais
	Alterações funcionais do miócito
Inibidores do *checkpoint* imunológico	Aumento de atividade dos linfócitos T
	Atividade autoimune no músculo cardíaco

### 1.4. Manifestações Clínicas de Cardiotoxicidade

As manifestações clínicas cardiovasculares decorrentes do tratamento oncológico remetem à ponta de um *iceberg* , cuja base consiste em alterações estruturais e funcionais que precedem sinais e sintomas. Com propósitos didáticos, optamos por dividir as manifestações de CTX em três subgrupos: clínicas, laboratoriais e imagens/traçados ( Tabela 2 ). Cabe ressaltar que tal proposta poderá, a princípio, ser alvo de críticas, uma vez que ainda não é factível o mapeamento genético de rotina, a fim de melhor atribuirmos a culpabilidade da expressão fenotípica.

**Tabela 2:** Fenótipos de cardiotoxicidade

Quadro clínico	HAS
	Hipertensão pulmonar
	Acidentes embólicos venosos e arteriais
	Doença arterial carotídea
	Insuficiência cardíaca/miocardite
	Derrame pericárdico/pericardite
	Disfunções valvares
	Isquemia miocárdica/infarto
	Doença pericárdica
Laboratoriais	Elevação de troponinas (T ou I) e/ou CKMB
	Elevação de peptídio natriurético (BNP/NT-pró-BNP)
Imagem/Traçados	Transtornos do ritmo cardíaco (extrassístoles, bloqueios, taquicardias supraventriculares e ventriculares, bradiarritmias, aumento do intervalo QT corrigido do eletrocardiograma)
	Dilatação das câmaras cardíacas com FEVE preservada
	Redução da FEVE > 10% da basal ou > 15% do strain longitudinal global
	Disfunção diastólica do ventrículo esquerdo
	Espessamento e/ou derrame pericárdico
	Disfunções valvares (estenoses, insuficiências)
	Alterações em exames de imagem que indiquem sinais inflamatórios em atividades ou necrose (cintilografia/ressonância cardíaca)
	Alterações em exames de angiotomografia das artérias coronárias ou escore de cálcio que teve seu início ou agravamento durante ou após o tratamento oncológico (QT e/ou RT)

HAS: hipertensão arterial sistêmica; BNP: peptídío natriurético cerebral; FEVE: fração de ejeção do ventrículo esquerdo; QT: quimioterapia; RT: radioterapia

Antraciclinas e anticorpos monoclonais anti-HER2 respondem pela maior parte dos casos documentados de disfunção ventricular esquerda. Cardinale et al. ^11^ demonstraram que a incidência de CTX por uso de antraciclinas em uma população de 2.625 pacientes foi de 9%, com 98% dos casos no primeiro ano de tratamento. ^11^ Agentes alquilantes, inibidores do proteassoma e alguns inibidores da tirosinoquinase também causam disfunção por diversos mecanismos. ^4^ Miocardite inflamatória grave pode estar associada ao uso de inibidores de *checkpoint* imunológicos em 0,27% nos pacientes com associação de nivolumab e ipilimumab. ^12^

A doença arterial coronariana (DAC) manifesta clinicamente como angina estável, instável ou IAM, pode ser secundária àlesão endotelial direta, trombose arterial aguda ou vasospasmo, dependendo da classe terapêutica usada. Aterosclerose obstrutiva, rotura de placa e trombose coronariana, degenerações anulovalvares e pericardites estão relacionadas com RT mediastinal e são dependentes da dose de radiação utilizada.HAS encontra-se intimamente ligada ao uso de inibidores do fator de crescimento endotelial. Trombose venosa profunda (TVP), doença arterial periférica e hipertensão pulmonar (HP) também compõem o leque de manifestações clínicas da CTX. ^4^

## 2. Cardiotoxicidade Miocárdica

### 2.1. Contribuição da Ecocardiografia

#### 2.1.1. Avaliação Estrutural e Funcional Miocárdica do Ventrículo Esquerdo

##### 2.1.1.1. Ecodopplercardiografia Padrão

A partir do momento que a disfunção miocárdica foi reconhecida como um potencial efeito adverso do tratamento oncológico, diversas estratégias foram testadas para monitorar a função miocárdica. Considerada como o método de maior acurácia, a biópsia endomiocárdica rapidamente caiu em desuso devido ao seu caráter invasivo, sendo substituída então pelo monitoramento seriado da função sistólica ventricular esquerda por exames não invasivos de imagem cardiovascular.

A ecocardiografia consolidou-se como a base do monitoramento da CTX por meio da FEVE, por ser um método amplamente disponível, custo-efetivo e inócuo, possibilitando ser repetido inúmeras vezes, além de fornecer diversas outras informações anatômicas e funcionais.

A aplicação do método de Simpson melhora a estimativa dos volumes cavitários, superando as limitações do encurtamento fracional e da fórmula de *Teichholz* , obtidos a partir de medidas lineares da ecocardiografia modo M ou bidimensional (2D). Entretanto, sua sensibilidade em detectar pequenas variações longitudinais na função sistólica ainda é baixa, principalmente devido a variações frequentes de pré e pós-carga durante a QT e da variabilidade intra e interobservador que podem atingir até 10% (justamente um dos parâmetros mais aceitos para o diagnóstico de CTX). ^13^ Importante lembrar que, em virtude dessas variações, exames com resultado fora dos parâmetros esperados devem ser repetidos e confirmados após 2 a 3 semanas do achado inicial.

Considera-se que o risco de CTX varia de 3,6 a 11,8 vezes no caso do emprego de fármacos cardiotóxicos (em especial das antraciclinas) se a FEVE pré-tratamento estiver entre 50% e 55%. Durante o monitoramentoe após o tratamento oncológico, sugere-se que a identificação da CTX pelo bidimensional se baseie na queda da FEVE > 10% (em relação aos valores pré-tratamento) para menos de 50%. ^14^ Essa situação é alvo de importante debate nas equipes médicas no que tange a risco cardiológico e benefício oncológico. Entre as condutas debatidas, encontram-se a troca por tratamentos com menor risco cardiotóxico, o emprego de medidas cardioprotetoras e até mesmo a suspensão do tratamento sempre em acordo com o oncologista. ^14^^,^^15^

O estudo da função sistólica longitudinal, em especial na indisponibilidade das metodologias avançadas (ecocardiografia tridimensional [3D] e estudo da deformação miocárdica) deve ser avaliado conjuntamente. Embora não exista valores de referência para o diagnóstico, é necessário valorizar o progressivo declínio da medida do pico de velocidade sistólica do anel mitral pelo Doppler tecidual (onda s’) e do deslocamento sistólico do anel mitral (MAPSE). ^16^

O número de vezes que se faz necessária a realização do estudo ecocardiográfico ainda gera discussão na literatura, variando conforme o risco individual, protocolo terapêutico (fármacos empregados e dosagem total) e identificação de sinais e sintomas de CTX.

É importante lembrar que a CTX, na forma de alterações quantitativas nos parâmetros convencionais de avaliação da função sistólica, pode não ser evidente até que haja uma redução substancial na reserva miocárdica. Assim, o dano cardíaco pode não se tornar aparente por anos ou até mesmo décadas após o término do tratamento cardiotóxico, fato que é particularmente aplicável a adultos sobreviventes de tumores durante a infância.

##### 2.1.1.2. Strain Miocárdico

O *strain* , ou deformação, é definido como a quantidade de deformação ou a mudança fracional no comprimento de um segmento do miocárdio, relacionada ao seu comprimento inicial. Tal parâmetro é expresso em porcentagem (%) e com o sinal negativo. ^17^

O *strain* 2D, derivado da técnica do rastreamento de pontos ( *speckle tracking* ), não sofre dependência do ângulo (fator limitante quando empregada a técnica pelo Doppler tecidual), sendo mais reprodutível e utilizada na prática clínica geral com o intuito de se detectar precocemente as alterações da mecânica miocárdica. ^17^ O *strain* 3D representa um aprimoramento da técnica. Nessa modalidade, todo um volume piramidal é analisado, obtido pela captura registrada em posição apical, de maneira bem mais rápida que as outras modalidades, mascom menor resolução espacial e temporal.

A queda da FEVE reflete um marcador de dano miocárdico tardio, acompanhado por pior prognóstico, com menor possibilidade de recuperação da função ventricular em 58% dos pacientes, apesar da intervenção com medicamentos cardioprotetores. A disfunção cardíaca somente se torna evidente quando o dano miocárdico é significativo; por conseguinte, a ausência de redução da FEVE não exclui CTX. ^18^^,^^19^

Deste modo, a aplicação do *strain* pela técnica do *speckle tracking* para analisar a mecânica ventricular gradativamente está se estendendo a todas as cardiopatias, especialmente aquelas associadas ao uso de antineoplásicos, como antracíclicos e trastuzumabe. ^20^ A possibilidade da detecção de lesões subclínicas tem sido uma das grandes vantagens na sua utilização. De um modo geral, embora a detecção precoce das mudanças seja conceitualmente importante, o valor dessas reais alterações deve comprovadamente se correlacionar com os desfechos.

Revisão de vários estudos demonstraram a capacidade do *strain* na detecção das alterações de deformação miocárdica de forma mais precoce que a queda da FEVE, seja imediatamente após a infusão da terapia ou em estágios mais tardios. ^21^

Ganame et al. ^22^ demonstraram os efeitos agudos dos antracílicos, capazes de induzir disfunção sistólica. ^22^ O mesmo grupo estudou 56 pacientes sem fatores de risco para doença cardiovascular (DCV), com diagnóstico de linfoma, leucemia e outros tumores malignos, tratados somente com antracíclicos (dose menor que 300mg/m^2^) e comparados com um grupo controle. ^23^ Após seguimento médio de 5,2 anos, foi demonstrada uma redução significativa do *strain* longitudinal global (SLG) em um momento em que a FEVE ainda era normal, sinalizando que novas ferramentas diagnósticas eram capazes de prever precocemente esse declínio.

Sawaya et al., ^24^ utilizando o *speckle tracking* 2D, demonstraram que o SLG e a troponina foram preditores de disfunção sistólica em pacientes com câncer de mama submetidos ao tratamento com antracíclicos e trastuzumabe. ^24^ Quarenta e três pacientes realizaram ECO antes, no terceiro e sexto mês do tratamento. Foram avaliados FEVE pelo método de Simpson biplanar, SLG, radial, circunferencial e biomarcadores. Nesse estudo, o SLG foi capaz de prever CTX em sete dos nove pacientes, com sensibilidade de 78% e especificidade de 79%. O evento ocorreu no terceiro mês de seguimento em uma das pacientes e no sexto mês entre as demais.

Tan et al. ^25^ examinaram a FEVE e o SLG em 19 pacientes com câncer de mama em uso de trastuzumabe, acompanhadas por um período de 34 meses (média de 24,7 meses). Observaram que as pacientes mantiveram alterações da função ventricular por longo período, com aumento dos diâmetros intracavitários e redução do SLG ao longo de todo o acompanhamento, questionando a reversibilidade da lesão causada pelo trastuzumabe. ^25^

Almeida et al. analisaram 40 pacientes com câncer de mama que tinham usado a doxorrubicina dois anos antes do ecocardiograma e compararam com 41 mulheres saudáveis. Os autores demonstraram que o SLG e a onda S´ do anel mitral estavam reduzidos nas pacientes que fizeram QT, apesar de manterem FEVE normal, sugerindo a presença de disfunção ventricular subclínica. Os autores mostraram ainda que a idade e o uso prévio da doxorrubicina foram marcadores independentes de redução do SLG. ^26^

Recentemente, Piveta et al. ^27^ avaliaram o papel do *strain* 3D em pacientes com câncer de mama submetidos a tratamento com antracíclicos. Após exposição à baixa dose do quimioterápico (120 mg/m^2^) apenas o *strain* circunferencial 3D e a *área do strain* 3D apresentaram alteração, enquanto os parâmetros derivados do *strain* 2D permaneceram inalterados. ^27^

Revisão sistemática com 1.504 pacientes mostrou que a redução relativa de 10% a 15% do SLG em relação ao valor basal foi importante preditor para o declínio da FEVE. Medidas do strain radial e circunferencial também apresentam alteração, porém tais variáveis ainda não são utilizadas rotineiramente. Na ausência de valores pré-QT para comparação, valores de SLG superiores a -19% são sugestivos de CTX, sendo que a associação com biomarcadores, em especial a troponina ultrassensível, aumenta a sensibilidade para o diagnóstico de CTX. É importante ressaltar que os valores de referência de normalidade sofrem variações a depender dos *softwares* dos aparelhos, idade e sexo dos pacientes. Daí a recomendação para se repetir os exames sempre no mesmo aparelho e, de preferência, com o mesmo examinador. ^21^

**Figura 1:**
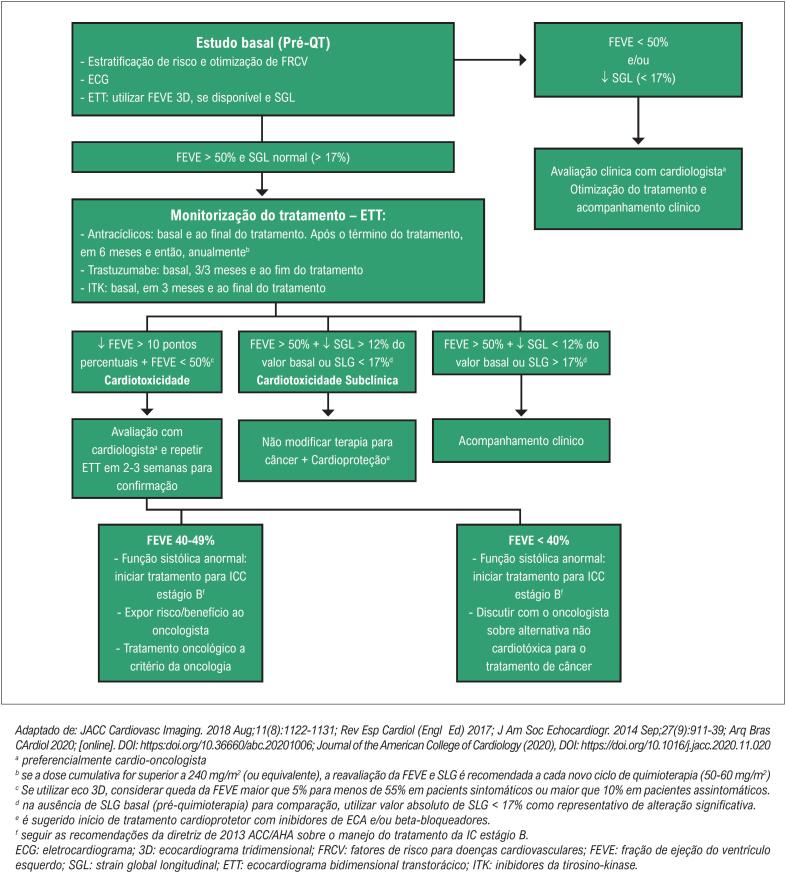
Monitoramento e manejo clínico durante terapia cardiotóxica.

Consenso das Sociedades Americana e Europeia de Imagem Cardiovascular sugere que as alterações de deformação precedem a disfunção ventricular. ^28^ Uma redução de >15% do SLG, imediatamente após ou durante o tratamento com antracíclicos, mostra-se o parâmetro mais útil em predizer CTX, enquanto uma redução menor que 8%, provavelmente, excluiria o diagnóstico de CTX ( Figura 1 ).

Em 2018, Liu et al. ^29^ descreveram algoritmo de acompanhamento de pacientes submetidos a tratamento com antineoplásicos, no qual as ferramentas ecocardiográficas utilizadas foram a FEVE e o SLG. Nos pacientes que apresentaram FEVE > 60%, a recomendação foi otimizar o controle dos fatores de risco cardiovasculares existentes. Aqueles com FEVE entre 50% e 59% e com SLG menor que-16% ou no limite inferior da normalidade foram classificados como função miocárdica preservada; já os que apresentavam o SLG maior que -16% ou uma redução percentual de 15% em relação ao valor basal foram considerados com disfunção subclínica. Os pacientes que apresentaram FEVE entre 40% e 49% foram considerados portadores de disfunção miocárdica, sendo indicados início de terapia cardioprotetora e avaliação em conjunto com o oncologista sobre riscos e benefícios da terapia antineoplásica nesse grupo específico, podendo eventualmente ser indicada redução da dose ou troca da medicação. Empacientes com FE<40%, recomenda-se iniciar terapia cardioprotetora e discutir com o oncologista o uso de terapia alternativa não cardiotóxica.

Não há consenso a respeito dos índices de função sistólica a serem acompanhadas ao longo do tratamento. ^14^^,^^15^^,^^29^ Porém, recentemente, foi publicado o estudo SUCCOUR ( *Strain sUrveillance of Chemotherapy for improving Cardiovascular Outcomes* ) em que foi demonstrado que o tratamento guiado pela queda maior de 12% do strain global longitudinal do VE nos pacientes tratados com antracíclicos é capaz de evitar a queda da fração de ejeção e de cardiotoxicidade em 1 ano. ^30^

Além do diagnóstico de CTX, a identificação da redução do SLG apresenta valor prognóstico, tendo sido associado à maior mortalidade tardia em estudo retrospectivo, envolvendo 120 pacientes acompanhados por um período de 21,6 ± 13,9 meses. ^31^

##### 2.1.1.3. Fração de Ejeção do Ventrículo Esquerdo pelo Método 3D

**Figura 2:**
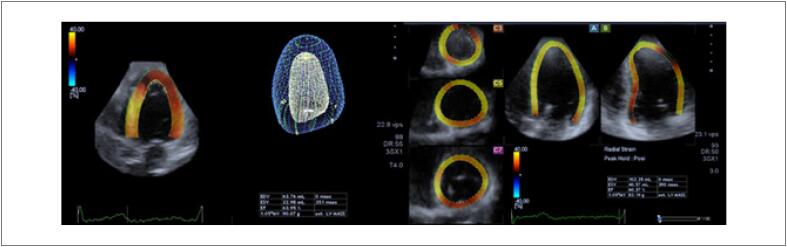
Exemplo de estudo ecocardiográfico tridimensional com análise do full volume e estimativa dos volumes e FEVE.

O estudo 3D é o método ecocardiográfico de escolha para o cálculo da FEVE durante o tratamento oncológico ( Figura 2 ). ^32^ Ao proporcionar maior proximidade à anatomia cardíaca, redunda em grande convergência com resultados obtidos pela ressonância magnética cardíaca (RMC) no cálculo dos volumes, massa e FEVE. ^33^ O estudo 3D não depende de presunções geométricas, como ocorre com a análise 2D, além de minimizar limitações relacionadas a tal técnica como o “encurtamento apical”.

A alteração predominante na CTX para consequente queda na FEVE é o aumento do volume sistólico final do VE. ^15^ Na população oncológica, estudos sugerem que a técnica 3D é preferível à 2D principalmente por ter demonstrado maior reprodutibilidade e maior acurácia no reconhecimento de FEVE limítrofe ou discretamente reduzida. Em sobreviventes de câncer tratados com antracíclicos, Armstrong et al. ^34^ demonstraram maior capacidade da técnica 3D em identificar pacientes com FEVE < 50% do que a avaliação 2D, com acurácia muito similar à RMC, o que possibilita a identificação mais precoce dos casos de CTX subclínica. ^34^ O estudo SUCCOUR utilizou dois critérios de cardiotoxicidade pelo ecocardiograma preferencialmente 3D: queda maior de 5% nos pacientes com sintomas de insuficiência cardíaca, ou maior de 10% nos assintomáticos, comparado com o exame inicial ( *baseline* ) para valores da fração de ejeção menores de 55%. ^30^

Em pacientes sob tratamento quimioterápico, Thavendiranathan et al. ^13^ compararam diferentes técnicas ecocardiográficas para a avaliação seriada da FEVE ao longo de 1 ano e demonstraram que a técnica 3D apresentou a menor variabilidade temporal intra e interobservador (5,6%). ^13^ Este dado sugere que, além de confiável, o estudo 3D representa um método consistente e reprodutível para a avaliação dos pacientes oncológicos. ^32^ Outros trabalhos também destacam a maior reprodutibilidade da técnica 3D no cálculo da FEVE, principalmente porque, tratando-se de técnica semiautomática para o traçado endocárdico, é menos afetada pela variabilidade na aquisição das imagens. ^35^

##### 2.1.1.4. Ecocardiografia com Contraste

A visibilização inadequada das bordas endocárdicas do VE ocorre frequentemente em pacientes sob tratamento quimioterápico de câncer de mama, em particular após mastectomia e RT. Como consequência, pode ocorrer subestimativa de volumes e determinação não acurada da FEVE. De acordo com diretrizes internacionais, um agente ultrasônico de contraste deve ser utilizado para a melhora do delineamento de bordas endocárdicas e da análise da função ventricular esquerda quando a visibilização endocárdica em dois ou mais segmentos estiver limitada. ^36^ Por outro lado, os agentes de contraste não são recomendados quando se estima a FEVE ao ecocardiograma 3D, pois acarretam menor reprodutibilidade e maior variabilidade temporal da FEVE do que ao se empregar o 3D isoladamente. ^13^

##### 2.1.1.5. Ecocardiografia sob Estresse

A ecocardiografia sob estresse físico ou farmacológico é um método estabelecido para detectar DAC obstrutiva e alterações subclínicas da função miocárdica. Pacientes oncológicos frequentemente têm diminuição da reserva cardiovascular global, atribuída aos efeitos diretos da terapia adjuvante do câncer e/ou aos efeitos indiretos das mudanças de estilo de vida associadas ao tratamento. ^37^ Assim, as potenciais utilidades para o emprego da ecocardiografia sob estresse nos pacientes sob terapia do câncer incluem: (a) investigação inicial da presença de DAC obstrutiva em pacientes com probabilidade pré-teste intermediária a alta, ECG não interpretável (físico) ou incapazes de se exercitar (dobutamina), especialmente se recebendo quimioterápicos associados à isquemia ou após RT de longa data; (b) determinação da RCVE como preditor de CTX em pacientes com FEVE e SLG normais em repouso; (c) determinação da RCVE na CTX instalada, na medida em que a recuperação transitória da função do VE durante o estresse poderia indicar melhor prognóstico. ^28^ Apesar dessas potencialidades, a ecocardiografia sob estresse tem sido pouco empregada no campo da cardio-oncologia.

Utilizando o ecocardiograma sob estresse físico em 57 mulheres assintomáticas com FEVE normal, tratadas de câncer de mama com antraciclinas, Khouri et al. ^38^ demonstraram redução da RCVE por meio da redução no volume sistólico em 12% e no índice cardíaco em 24% em relação ao repouso quando comparadas aos controles. ^38^

Civelli et al. ^39^ mediram prospectivamente a RCVE (definida pela diferença entre a FEVE no pico e no repouso) por meio do ecocardiograma com dobutamina em baixa dose durante e após QT em alta dose em 49 mulheres com câncer de mama avançado. Um declínio assintomático ≥ 5% da RCVE em relação ao basal foi capaz de predizer queda na FEVE para < 50%. ^39^

A única revisão sistemática publicada sobre a utilidade dos métodos de estresse cardíaco para detecção de DCV em sobreviventes do câncer de mama concluiu que parece haver evidências de que a ecocardiografia sob estresse traga benefício para a avaliação prognóstica precoce e seguimento tardio após terapia com antraciclinas. ^40^

Antes que a incorporação da ecocardiografia sob estresse possa ser rotineiramente adicionada à prática clínica cardio-oncológica, mais estudos são necessários para determinar o melhor agente estressor, quaisparâmetros devem ser medidos durante o exame, o melhor momento em que deve ser realizado o exame de acordo com os diferentes tipos de tratamento, o custo-benefício e a exequibilidade na população oncológica e, finalmente, a presença de valor incremental prognóstico sobre os parâmetros tradicionais medidos no repouso (FEVE e SLG).

##### 2.1.1.6. Função Diastólica

Anormalidades em parâmetros relacionados com a função diastólica, tais como ondas E e A, relação E/A, tempo de relaxamento isovolumétrico e índice de *performance* miocárdica, foram descritas precocemente após QT. ^41^^,^^42^ Entretanto, estudos longitudinais não foram capazes de reproduzir o valor prognóstico desses achados e não há evidências suficientes para recomendar essa análise no diagnóstico de CTX induzida por quimioterápicos. ^43^

Estudos demonstraram a utilidade da análise derivada do Doppler tecidual (DT) durante a avaliação da função diastólica em pacientes sob tratamento contra o câncer. Trabalhos revelaram redução na velocidade diastólica precoce (onda e’) derivada do DT do anel mitral em pacientes tratados com antracíclicos, que permaneceu reduzida durante o tratamento e anos após seu término, não demonstrando, entretanto, valor preditivo de CTX. ^16^ Negishi et al. ^44^ revelaram que uma redução de 10% na velocidade da onda e’ foi observada em pacientes que desenvolveram CTX após tratamento com maiores doses cumulativas de doxorrubicina, mas esse parâmetro não demonstrou papel preditivo de queda da FEVE. ^44^

Questiona-se a utilização da disfunção diastólica como marcador específico de CTX. As alterações podem ocorrer por mudanças nas condições de pré-carga, como resultado de reposições volêmicas associadas ao tratamento oncológico ou depleção de volume devido a efeitos colaterais da QT, como náuseas, vômito e diarreia. Nesses casos, podem não representar uma mudança real no desempenho diastólico do ventrículo esquerdo (VE).

#### 2.1.2. Avaliação Estrutural e Funcional Miocárdica do Ventrículo Direito

A prevalência do envolvimento do ventrículo direito (VD) e seu valor prognóstico ainda não foram adequadamente estudados. Dados a respeito da influência da QT no remodelamento, na função e na mecânica ventricular direita são escassos e, por vezes, conflitantes.

A dificuldade inerente à avaliação do VD e às variações determinadas pelas condições hemodinâmicas sugerem que parâmetros tais como excursão sistólica do anel tricúspide (TAPSE), velocidade tecidual da região basal da parede livre do VD (onda S`), fração da variação da área do VD (FAC), além da deformidade miocárdica pelo *strain* sejam os métodos ideais pela ecocardiografia, deixando a avaliação da fração de ejeção do ventrículo direito (FEVD) para a Ressonância Magnética Cardíaca, ou ecocardiografia 3D.

Entre os fármacos mais frequentemente relacionados com disfunção do VD e alterações na circulação pulmonar, temos: antraciclinas, trastuzumabe, ciclofosfamida e dasatinibe. ^45^ Boczar et al, avaliaram o *strain* longitudinal do VD em pacientes com câncer de mama tratadas com antraciclina. Após 3 meses, observaram uma redução do *strain* de -16,2% para -13,81%. ^46^ A diferença do *strain* longitudinal do VD é mais pronunciada quando desconsiderada a região septal, sugerindo uma sensibilidade maior do miocárdico do VD. ^47^

#### 2.1.3. Seguimento Ecocardiográfico Tardio

**Figura 3:**
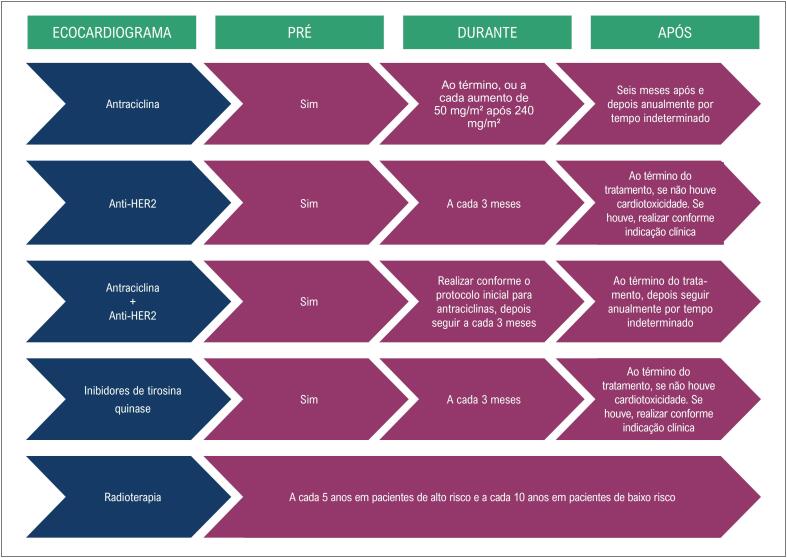
Recomendações para o seguimento ecocardiográfico tardio.

As recomendações acerca do acompanhamento dos pacientes após a finalização da QT variam de acordo com as características clínicas da população estudada, do protocolo utilizado na QT (com ou sem RT) e da dose cumulativa dos fármacos (principalmente trastuzumabe e antracíclicos). Devido ao maior risco de desenvolver CTX nos primeiros 12 meses após antraciclina, à queda da FEVE durante o uso de trastuzumabe e ao surgimento de complicações tardias pós RT, sugerimos o acompanhamento tardio, conforme mostrado na Figura 3 .

### 2.2. Contribuição da Medicina Nuclear

#### 2.2.1. Ventriculografia Radionuclídica ou Radiosiotópica

A ventriculografia radioisotópica (do inglês, *multigated radionuclide angiography* [MUGA]) é um exame cintilográfico, realizado em equipamento de gama-câmara, no serviço de medicina nuclear (MN). Trata-se de um método não invasivo, com baixa taxa de exposição à radiação e que não determina efeitos colaterais graves, com ótima reprodutibilidade e baixa variabilidade inter e intraobservadores na avaliação da função ventricular. ^48^

Juntamente com a ecocardiografia, a ventriculografia radioisotópica é o método mais amplamente aceito para avaliar a FEVE de pacientes antes e durante tratamento do câncer na identificação do risco para IC crônica (90% de sensibilidade e 72% de especificidade). ^49^

**Figura 4:**
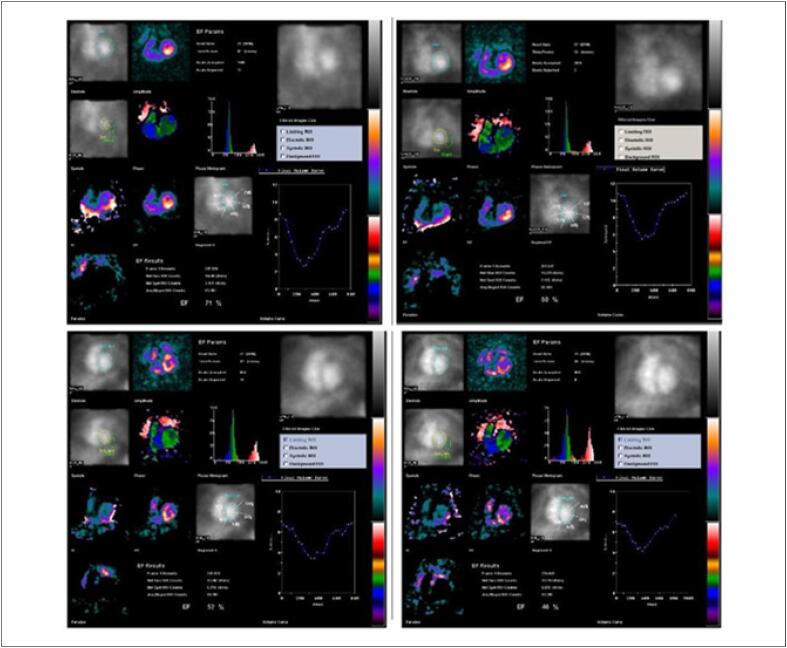
Exemplos de dois pacientes que realizaram ventriculografia radioisotópica para medição da FEVE antes e após o término da quimioterapia (QT) com antracíclicos. Na fileira superior, à esquerda, está a FEVE basal do primeiro paciente, que foi de 71% (normal), e após o término da QT e início de tratamento com trastuzumabe, houve queda da FEVE para 50% (superior à direita). Na fileira inferior, paciente com câncer de mama tinha uma FEVE de 52% antes do início da QT e, no acompanhamento, houve redução da FEVE para 46%, o que motivou início de medicações cardioprotetoras. Imagens gentilmente cedidas pela Dra. Márcia Modesto (Serviço de Medicina Nuclear do Instituto Brasileiro de Controle do Câncer, São Paulo, Brasil).

Com uma FEVE basal normal, as próximas medições devem ser feitas em doses cumulativas de 250 a 300 e 400 a 450mg/m^2^. Para pacientes com FEVE basal anormal (< 50%), estudos seriados são recomendados antes de cada dose subsequente de doxorrubicina. A Figura 4 mostra exemplos de comportamento da FEVE, avaliada pela ventriculografia radioisotópica, em pacientes realizando QT.

#### 2.2.2. Avaliação da Atividade Simpática Cardíaca com mIBG

A metaiodobenzilguanidina (mIBG) é uma molécula com estrutura similar a NE e age seletivamente sobre os nervos simpáticos sem, no entanto, ser metabolizada pela monoamina oxidase ou pela catecol-O-metil transferase, e não exercendo efeito estimulatório, como a norepinefrina (NE). A cintilografia cardíaca com mIBG-I ^123^ avalia diretamente a função simpática global e regional do coração, incluindo a captação, a recaptação, o armazenamento e os processos de liberação da NE nas terminações nervosas pré-sinápticas. ^50^

Guimarães et al. ^51^ realizaram a cintilografia cardíaca com mIBG-I ^123^ em 20 mulheres com câncer de mama e FEVE normal que tinham sido submetidas a tratamento com derivados das antraciclinas associado ou não ao trastuzumabe. Os autores observaram que o uso da antraciclina com o trastuzumabe proporcionou maior frequência e intensidade de hiperatividade adrenérgica cardíaca. ^51^ Outro estudo identificou captação anormal de mIBG-I ^123^ em pacientes que usaram antracíclico. Além disso, a relação da captação entre o coração e o mediastino (RC/M) de mIBG-I ^123^ foi mais baixa à medida que a dose cumulativa dessa medicação aumentava. ^52^ Esses resultados dão suporte à hipótese de que a instituição precoce de cardioproteção com fármacosbetabloqueadores pode ser benéfica para esses pacientes.

#### 2.2.3. Metabolismo Miocárdico – 18F-FDG PET-CT

Recentemente, Borde et al. ^53^ analisaram retrospectivamente a captação miocárdica de ^18^ F-FDG em pacientes com linfoma tratados com QTà base de adriamicina. ^53^ Os autores demostraram que um aumento na atividade metabólica do miocárdio pode ser um possível marcador de alteração celular que antecede a cascata de CTX. Neste estudo, o aumento do metabolismo glicolítico no músculo cardíaco foi diretamente proporcional à dose cumulativa de adriamicina recebida em mg/m^2^. Dados semelhantes também foram propostos em pacientes com dano cardíaco induzido por radiação. ^54^ Toubert et al. ^55^ demonstraram modificação na captação miocárdica de ^18^ F-FDG em paciente tratado com uma combinação de inibidores de tirosina quinase (imatinibe e sorafenibe) que posteriormente desenvolveu IC fatal ( Tabela 3 ). ^55^

**Tabela 3:** Estudos com PET-CT que avaliaram a relação da captação miocárdica de 18F-FDG em pacientes submetidos à quimioterapia ou radioterapia ^55^

PET-CT ^18^ F-FDG miocárdico x fármacos: o que dizem os estudos?	
Borde et al. ^53^	Adriamicina: aumento da concentração de ^18^ F-FDG no miocárdio pode preceder a redução da função ventricular esquerda e o grau de cardiotoxidade é proporcional à dose recebida.
Toubert et al. ^55^	Inibidores da tirosina quinase (imatinibe e sorafenibe): relato de caso que mostrou modificação na concentração miocárdica de ^18^ F-FDG previamente a evento cardíaco fatal.
Evans et al. ^58^	Aumento na concentração ^18^ F-FDG em pacientes que receberam radioterapiatorácica >20 Gy com área cardíaca > 5 cm^2^: indicador de lesão miocárdica.

As imagens de PET-CT utilizando o radiofármaco ^18^ F-FDG, bem como as obtidas pela ressonância magnética, podem ser úteis no diagnóstico da CTX induzida pelos inibidores dos *checkpoints* imunológicos (ICI), visto que permitem detectar, avaliar a extensão e até mesmo quantificar o processo inflamatório de diversas afecções cardiovasculares, tais como miocardite, pericardite e vasculites. ^56^^-^^58^

### 2.3. Contribuição da Ressonância Magnética Cardiovascular

#### 2.3.1. Avaliação da Cardiotoxicidadedurante o Tratamento Antineoplásico

A RMC é considerada o padrão-ouro para avaliação da FEVE, massa e volume das cavidades, sendo uma ferramente valiosa na avaliação de CTX induzida por QT. ^34^^,^^59^^,^^60^ A FEVE pode reduzir a partir da alteração de um ou ambos os volumes. Em geral, a redução do volume diastólico encontra-se relacionada com condições de pré-carga que sofrem alterações dinâmicas e constantes em um paciente oncológico seja por vômitos, sangramentos, diarreia ou desidratação. Estas são prontamente reversíveis com a restauração da volemia. No entanto, o aumento progressivo do volume sistólico é considerado um marcador de lesãomiocárdica relacionada à QT. Outro parâmetro que merece atenção durante o acompanhamento quimioterápico é a avaliação da massa ventricular. A CTX provoca aumento inicial da massa ventricular, provavelmente pela indução da resposta inflamatória pela QT, levando a aumento do volume do interstício e dos cardiomiócitos. No entnto, nas fases tardias, geralmente após 6 meses, ocorre um processo de redução da massa ventricular (apoptose, fibrose, hipotrofia cardiomiocitária etc.). Dessa forma, quando estamos diante de um aumento do volume sistólico e redução da massa ventricular, tal combinação é muito sugestiva de CTX, mesmo com fraçãode ejeção ainda preservada.

Mais recentemente, a análise de deformação miocárdica ( *strain* ) pela RMC vem ganhando destaque. Na população em tratamento quimioterápico, a literatura destaca o seu potencial em diagnosticar alterações subclínicas. ^61^^-^^63^ Jolly et al. ^61^ estudaram 72 pacientes em tratamento quimioterápico para câncer de mama, sarcoma ou linfoma com RMC seriadas. ^61^ Comparado com a RMC basal, houve uma piora significativa do *strain* circunferencial global (SCG) 3 meses após tratamento. Além disso, o SCG se correlaciou fortemente com a queda subclínica da FEVE. Em outro estudo com 41 pacientes portadoras de câncer de mama em tratamento com trastuzumabe, tanto o SLG quanto o SCG reduziram durante o tratamento e correlacionaram-se com a queda da FEVE. ^62^ O *strain* pela RMC ainda não é amplamente utilizado na prática clínica, mas os estudos demonstram sua aplicabilidade potencial nos pacientes da cardio-oncologia.

#### 2.3.2. Ressonância Magnética Cardiovascular no Seguimento Tardio

A avaliação tardia fornece importantes informações diagnósticas e prognósticas pela RMC através de sua complementação com a análise tecidual. É importante compreender que alterações precoces durante o tratamento são marcadores para algumas alterações tardias, como a presença de realce tardio. O edema miocárdico é indicativo de processo inflamatório no miocárdio e, quanto maior for o conteúdo líquido, maior será a intensidade de sinal local. ^64^ A sequência que auxilia neste contexto é o *triple-IR* , que é adquirida com a adição de um terceiro pulso de saturação, para eliminar todo o sinal de tecido adiposo (saturação de gordura) da imagem. ^64^^,^^65^ Alguns estudos têm demonstrado edema miocárdico na RMC logo após a terapia com antraciclina, usando sequências ponderadas em T2. ^66^ O aumento precoce do edema e o aumento subagudo da fibrose vistos em ratos que receberam antraciclinas foram fortemente relacionados e são ambos indicadores de mortalidade tardia. ^67^

O realce tardio é uma técnica amplamente validada para identificar e quantificar a fibrose miocárdica de maneira não invasiva utilizando contraste paramagnético de gadolíneo. Padrões distintos de realce tardio são reconhecidos na RMC permitindo diferenciar os diferentes tipos de acometimento miocárdico, distinguindo entre miocardiopatias isquêmicas e não isquêmicas. ^68^ Isso é de particular importância durante a avaliação da cardiomiopatia induzida por QT em indivíduos com condições cardíacas preexistentes, como DAC.

**Figura 5:**
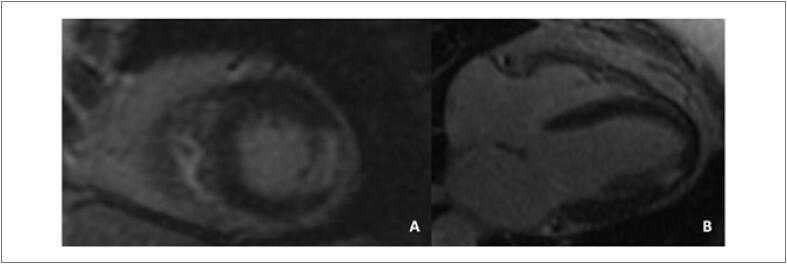
Realce tardio focal em parede ínferolateral do ventrículo esquerdo em paciente com câncer de mama tratada com antraciclina e trastuzumabe.

A prevalência de realce tardio na CTX é baixa. ^59^ Embora seja o padrão-ouro para identificar áreas focais de fibrose miocárdica, ele não é adequado para avaliar a fibrose intersticial difusa, o que geralmente é encontrado na cardiomiopatia causada por CTX.Não há um padrão típico de realce tardio encontrado de CTX relacionada comantraciclinas e trastuzumabe, podendo haver queda da FEVE mesmo sem realce tardio. ^60^^,^^69^ Quando presente, caracteristicamente, assemelha-se aos padrões já conhecidos das miocardiopatias não isquêmicas (epicárdico, mesocárdico, focal e/ou juncional) ( Figura 5 ). ^68^^,^^70^

A miocardite é uma complicação grave e potencialmente fatal nos pacientes em uso de imunoterápicos, com mortalidade de aproximadamente 40%. ^71^ Pode ter uma incidência mais elevada e até se manifestar como quadro fulminante naqueles pacientes em uso de inibidores de *checkpoint* imunológico, principalmente se estiverem em terapia combinada e em média 34 dias após o início da terapia. ^72^

#### 2.3.3. Caracterização Tecidual pela RMC

##### 2.3.3.1. Mapas de T2

A RMC tem a vantagem de fornecer informações detalhadas sobre o remodelamento cardíaco, que são complementares à tradicional avaliação morfológica e funcional do miocárdio.Dados consistentes indicam que edema, inflamação, expansão da matriz de tecido conectivo e alteração da deformação regional do miocárdio ocorrem antes da disfunção miocárdica em pacientes submetidos a tratamento cardiotóxico, reforçando o potencial de aplicabilidade dessa modalidade em indivíduos expostos a terapias cardiotóxicas.

Estudos clínicos mostraram, recentemente, que a utilização de protocolos de RMC que incorporam sequências multiparamétricas com mapas de T1 e T2 proporcionoua detecção de alterações pré-clínicas e precoces da CTX. ^73^^,^^74^ A detecção de edema no miocárdio, que normalmente é baseada no aumento da intensidade de sinal nas imagens ponderadas em T2 no miocárdio normalizadas para os valores do músculo esquelético, vem sendo aplicada com sucesso para doença isquêmica e não isquêmica do músculo cardíaco. ^75^^-^^77^

Utilizando sequências amplamente disponíveis e mais simples que os mapas de T2, Ferreira de Souza documentou aumento significativo da intensidade de sinal em T2 em mulheres tratadas com doses moderadas de doxorrubicina (240 mg/m^2^). ^74^ Diversos grupos estão atualmente investigando a aplicabilidade dos mapas de T2 na avaliação de edema miocárdico em sobreviventes de câncer tratados com terapias antineoplásicas, mas, até o momento, poucos dados clínicos estão disponíveis para justificar o seu emprego rotineiro na pratica do diaadia.

##### 2.3.3.2. Mapas de T1

Apesar de a técnica do realce tardio demonstrar de forma precisa a presença de cicatriz e fibrose de substituição, esta técnica não é capaz de fornecer dados completos e definitivos a respeito da quantidade total de fibrose no miocárdio, sobretudo sobre a fibrose intersticial. ^78^ Como a técnica do realce tardio é fundamentadaem diferenças relativas da intensidade de sinal em T1 após a administração de meio de contraste à base de gadolínio, é possível que a fibrose intersticial difusa seja visualizada de forma incompleta ou limitada por essa técnica.

Em diversos estudos, incluindo observações retrospectivas e prospectivas, o realce tardio não foi uniformemente detectado em pacientes submetidos ao tratamento com QT à base de antracíclico. ^79^^,^^80^ De fato, um exame de RMC com realce tardio negativo ou sem a presença de áreas com fibrose não excluinecessariamente a presença de fibrose miocárdica, reforçando a limitação dessa metodologia nesse contexto. Apesar de Fallah-Rad ter demonstrado a presença de realce tardio subepicárdico em todos os pacientes que apresentaram disfunção ventricular esquerda induzida pelo trastuzumabe, ^79^ diversos outros estudos evidenciaram resultados conflitantes, sendo que os dados de Lawley et al. ^81^ mostraram que apenas < 10% das mulheres tratadas com trastuzumabe apresentaramrealce tardio positivo. ^81^

Novas técnicas baseadas em medidas sucessivas de T1 realizada antes e após a administração de meio de contraste à base de gadolínio proporcionam uma avaliação mais precisa e acurada acerca do volume extracelular no miocárdio (ECV), um marcador da matriz de tecido conectivo no miocárdio e da fibrose intersticial. Mesmo antes do advento dos mapas de T1, autores já mostravam que medidas de T1 no miocárdio conseguem detectar o aumento da distribuição do gadolínio no miocárdio em sobreviventes de câncer tratados com QT. ^79^ Esses achados indicam que a expansão da matriz de tecido conectivo avaliado por mapas de T1 emerge como um marcador precoce da CTX, e que é capaz de predizer inclusive a piora da capacidade funcional.

Pacientes previamente expostos a antracíclicosapresentam valores de ECV significativamente maiores quando comparados a controles saudáveis pareados para idade e sexo.Jordan et al. investigaram pacientes com e sem câncer, incluindo indivíduos tratados e não tratados com antraciclina, assim como indivíduos tratados com outros quimioterápicos. ^82^ Os valores do T1 nativo (antes da administração de contraste) foram consistentemente maiores antes e após o tratamento nos indivíduos com câncer comparado aos indivíduos sem câncer.Nesse estudo ainda foi demonstrado que o ECV aumentou significativamente após o tratamento com antracíclicos (30,4 ± 0,7% *vs.* 27,8 ± 0,7%; p < 0,01). Além disso, utilizando modelos estatísticos multivariados, tanto os valores de T1 nativo como do ECV permaneceram consistentemente maiores nos pacientes com câncer independentemente do ajuste para diversas outras variáveis.

Recentemente, foi demonstrado que a quantificação do tempo intracelular da água (T_1_) no miocárdio emerge como uma abordagem inovadora para detectar o remodelamento miocárdico após o tratamento de câncer, oferecendo informações complementares à avaliação isolada do ECV. ^15^ Estudo recente avaliou 27 mulheres com câncer de mama antes e após dose cumulativa de doxorrubicinade 240mg/m^2^, incluindo a realização de biomarcadores. No período de 1 a 2 anos após doxorrubicina, observou-se queda da FEVE e da massa indexada do VE. Por outro lado, houve aumento do ECV e redução do tempo intracelular da água. ^74^

## 3.Toxicidade Vascular

### 3.1. Contribuição da Ultrassonografia Vascular

#### 3.1.1. Tromboembolismo Venoso e Câncer

##### 3.1.1.1. Introdução

A associação entre câncer e TVP e/ou superficial foi relatada pela primeira vez por Armand Trousseau em 1865. ^83^ O risco relativo de tromboembolismo venoso (TEV) é cerca de sete vezes maior em pacientes com câncer ativo, sendo que o TEV pode ser a primeira manifestação de um câncer oculto (7% a 12% dos casos), constituindo um “fenômeno paraneoplásico”. ^84^^,^^85^

Ocorrência de trombose complica o manejo do paciente com câncer em virtudeda necessidade de terapia anticoagulante e o risco potencial de sangramento. Portadores de neoplasia e TEV aguda apresentam risco maior de recorrência de trombose do que os nãoportadores. Por fim, um episódio de TEV aumenta a mortalidade dos pacientes oncológicos e isso pode ser resultado de embolia pulmonar maciça ou avanço do tumor. ^86^

##### 3.1.1.2. Epidemiologia

Existem diversos fatores associados que podem aumentar o risco de trombose em pacientes com câncer: tratamento (RT e/ou QT), pós-operatório e dispositivos intravasculares. ^87^ A incidência de TVP em pacientes com câncer é 4,7 vezes maior do que na população sem câncer, após ajustes para comorbidades, como foi demonstrado na população da Dinamarca. ^88^ A incidência nos primeiros 2anos varia de 0,8% a 8,0% e está associada a altas taxas de recorrência e mortalidade. A maior incidência de TEV no primeiro ano após o diagnóstico ocorre naqueles com doença avançada do cérebro, pulmão, útero, bexiga, pâncreas, estômago e rim (4 a 13 vezes maior na doença metastática do que naqueles com doença localizada). QT aumenta em 7 vezes o risco de ocorrência de TEV em paciente com câncer.

##### 3.1.1.3. Diagnóstico de TVP

Ultrassonografia venosa é o exame de imagem padrão indicado para os pacientes com suspeita de trombose venosa (profunda e/ou superficial), seja em vasos centrais, abdominais ou extremidades (região cervical, membros superiores e membros inferiores). Além de confirmar ou afastar a presença de trombos, permite diagnóstico diferencial com outras patologias.

Na prática clínica, a definição do segmento do corpo a ser examinado depende dos sinais e sintomas apresentados pelo paciente. Nos casos de suspeita de embolismo pulmonar e ausência de indícios da localização da fonte de trombos, o rastreamento com ultrassom venoso deverá dar prioridade aos vasos dos membros inferiores (sítio de 85% dos casos de TVP). Se a pesquisa for negativa nesse território, a sequência da investigação incluirá as veias ilíacas e cava inferior; por fim, vasos cervicais e de membros superiores.

##### 3.1.1.4. Protocolos de Ultrassonografia Venosa

Recomenda-se que pacientes com “escore de Wells” ≥ 2 devem realizar a ultrasonografia venosa (envolvendo a compressão de todo o membro inferior a cada 2 cm desde o ligamento inguinal até o tornozelo, incluindo-se as veias tibiais posteriores, fibulares e musculares da panturrilha – gastrocnêmias e soleares; e as imagens coloridas com a análise espectral pelo menos das veias femoral comum e poplítea), pois se somente o segmento femoropoplíteo tiver sido examinado e restar dúvida, o exame completo deverá ser repetido em 5 a 7 dias para afastar TVP (retardando diagnóstico e aumentando riscos e custos). Se houver áreas sintomáticas não incluídas no protocolo, estas deverão obrigatoriamente ser avaliadas (p. ex., veias tibiais anteriores, veias plantares etc). ^89^

Caso não haja possibilidade de realização do exame completo, é aceito que inicialmente seja feito o exame de dois pontos (CUS) até que novo estudo (protocolo completo) possa ser efetuado. É sabido que a disponibilidade dessa modalidade de diagnóstico é limitada em centros médicos menores, ambulatórios, nas noites e finais de semana.

**Figura 6:**
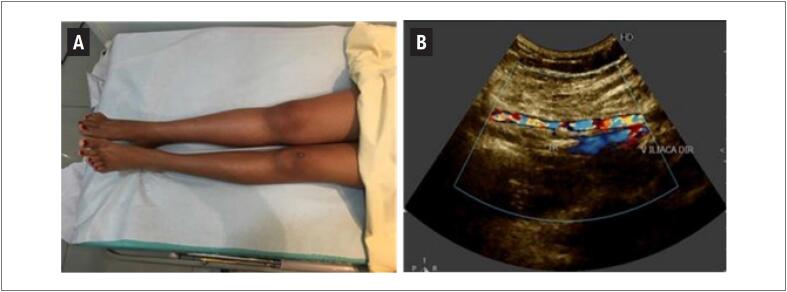
A e B – Edema rizomélico em paciente adolescente com câncer e trombo ocluindo veia ilíaca direita.

Exames específicos das veias ilíacas e da veia cava inferior devem ser reservados para casos duvidosos; tromboses rizomélicas, desde a raiz da coxa ( Figura 6 A-B ); ou quando as curvas espectrais de fluxo (Doppler pulsátil) em femoral comum apresentarem padrão contínuo (perda de fasicidade), sugerindo comprometimento proximal.

##### 3.1.1.5. Diagnóstico Diferencialde TVP

Portadores de neoplasia (oculta ou em atividade, acamados ou não, em QT ou RT, em uso de anticoagulantes, com cateter venoso para infusão de medicamento) e outras comorbidades podem apresentar complicações que exigem diagnóstico diferencial com trombose venosa. Dentre essas, as mais frequentes são edema (uni ou bilateral) em qualquer localização e dor na panturrilha.

A ultrassonografia, além de afastar a presença de trombos na luz dos vasos em segmentos edemaciados, possibilita o diagnóstico de massas tumorais compressivas de veias ilíacas como causa de edema uni ou bilateral; “síndrome de May-Turner” e edema de membro inferior esquerdo; celulites extensas; massas mediastinais comprimindo veia cava superior, com edema de membros superiores e pescoço. Edema bilateral raramente é causado por trombose, devendo-se afastar condições sistêmicas (insuficiência cardíaca, insuficiência hepática, falência renal, hipotireoidismo) ou uso de medicamentos.

Outra vantagem da ultrassonografia nos casos de aumento significativo de volume dos membros decorrentes de compressão por massas tumorais, TVP extensa ou hematomas volumosos é a avaliação rápida de possível comprometimento associado ao fluxo arterial regional (“síndrome compartimental”), que pode levar à isquemia grave.

#### 3.1.2. Trombose Relacionada a Cateter em Pacientes com Câncer

##### 3.1.2.1. Introdução

A utilização de cateteres de longa permanência é frequente na população de pacientes com câncer devido à necessidade de infusão de quimioterápicos, além da administração endovenosa de terapias de suporte. A trombose relacionada ao cateter (TRC) é definida como um trombo mural que se estende do cateter ao lúmen do vaso, levando à obstrução parcial ou oclusão do cateter, com ou sem sintomas clínicos. ^90^

A maioria dos casos de TRC ocorre dentro dos primeiros 100 dias após a inserção do cateter. A taxa de TRC situa-se na faixa de 14% e 18% quando realizado rastreamento com venografia ou ultrassonografia vascular (USV). Destes, menos de 5% dos pacientes são sintomáticos. ^91^

A TRC pode causar embolia pulmonar em 15% e perda do acesso em 10% dos pacientes, elevando significantemente os custos relacionados ao tratamento e manuseio desses pacientes. ^92^

##### 3.1.2.2. Fatores de Risco

Uma metanálise que incluiu cinco estudos randomizados de indivíduos com e sem câncer avaliou prospectivamente as variáveis relacionadas com TRC, sendo significantes: ^93^

- Inserção em veia subclávia × membro superior (OR 2,16; 95% IC 1,07-4,34).
- História prévia de TVP (OR 2,03; 95% IC 1,05-3,92).
- Posição imprópria da ponta do cateter (OR 1,92; 95% IC 1,22-3,02).

Em análise multivariada, o cateter *port-a-cath* apresentou uma redução de 57% no risco de TRC quando comparado ao cateter *PICC (peripherallyinserted central catheter)* . ^93^

##### 3.1.2.3. Diagnóstico e Complicações

**Figura 7:**
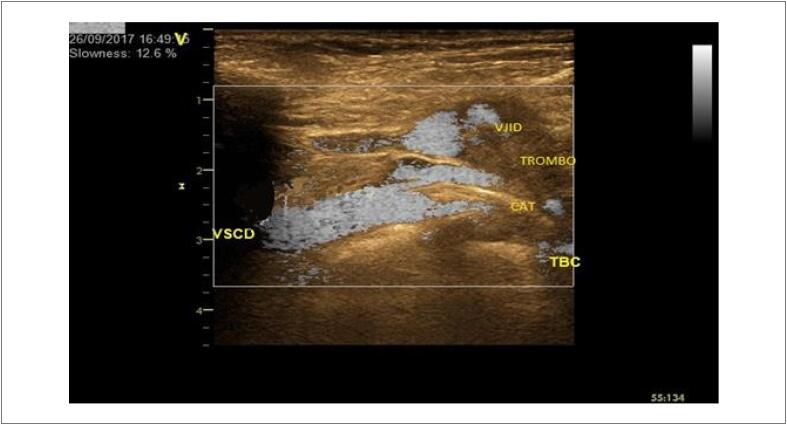
Ultrassonografia vascular (USV) na região supraclavicular proximal, evidenciando trombose na veia jugular interna direita (VJID) proximal, tronco venoso braquiocefálico (TBC) e veia subclávia direita proximal (VSCD). Observa-se o cateter central em VSCD direcionado ao TBC.

Na trombose envolvendo as veias do braço, axilar, subclávia distal ou jugular, a sensibilidade e a especificidade sãobastante elevadas (> 95%) ( Figura 7 ). Esses valores, contudo, caem para a faixa de 55% nas tromboses envolvendo a veia subclávia proximal, o tronco venoso braquiocefálico e a veia cava superior. Isso ocorre devido à natural dificuldade de abordagem, imposta pela barreira anatômica de osso e pulmão nesta região. Uma alternativa é o uso de sondas com menor área de superfície de contato como sondas setoriais pediátricas e/ou adultas e sondas microconvexas.As complicações mais frequentes dos trombos são infecção secundária (colonização bacteriana), embolia pulmonar e síndrome pós-trombótica. ^94^^,^^95^

#### 3.1.3. Hipertensão Pulmonar no Paciente Oncológico

Conceitualmente, a HP é definida por um aumento da pressão arterial média superior a 20 mmHg durante estudo hemodinâmico invasivo. A forma mais comum é decorrente do acometimento do VE (Tipo II da Organização Mundial de Saúde).

O ecocardiograma continua sendo a ferramenta de primeira linha na investigação diagnóstica de HP, porém é importante reforçar a complexidade clínica do diagnóstico etiológico da HP nos pacientes oncológicos, em que podemos encontrar:

- Massas ou tumores no átrio esquerdo, causando efeito obstrutivo e, com isso, levar a um aumento da pressão capilar;
- Compressão tumoral extrínseca, como visto nos casos de grandes timomas, linfomas ou mediastinite fibrosante;
- Tromboembolismo pulmonar, secundário aos inibidores de tirosina-quinase como o dasatinibe ou ao acometimento do VE (CTX, cardiopatia preexistente, valvopatias etc.). ^96^

Em pacientes com HP, a tomografia computadorizada tem um valor especial na avaliação da vascularização pulmonar e do parênquima pulmonar, fornecendo informações sobre possíveis mecanismos e consequências da HP. Os achados parenquimatosos pulmonares podem ser variados e dependem de sua etiologia. ^97^

A tomografia sem contraste permite a detecção de anormalidades relacionadas com HP como aumento das dimensões do tronco pulmonar, dilatação de câmaras direitas e identifica quadros secundários à doença pulmonar, sendo suficiente para a avaliação da maioria das doenças pulmonares. É indicada na avaliação do parênquima pulmonar em doenças pulmonares difusas, como pneumopatias intersticiais e doença pulmonar obstrutiva crônica, detecção de malformações arteriovenosas e no diagnóstico de doença veno-oclusiva ou hemangiomatose capilar pulmonar.

A tomografia com contraste define a anatomia e a estrutura vascular pulmonar, obtendo imagens angiográficas precisas do tronco pulmonar até o nível vascular subsegmentar. É amplamente disponível, de fácil execução e mais sensível que a angiografia invasiva na detecção de êmbolos pulmonares e, assim, tornou-se a modalidade padrão para o diagnóstico não invasivo de embolia pulmonar aguda.

No cenário de HP, o VD vai refletir a sobrecarga pressórica com hipertrofia e dilatação, podendo apresentar disfunção ventricular. Distorção significativa da geometria ventricular pode estar presente, com o VD assumindo conformação globosa, levando à compressão ventricular esquerda em estágios mais avançados.Pacientes com HP com dilatação de VD e fração de ejeção reduzida de VD têm pior prognóstico. ^98^

A aquisição em cine fornece uma avaliação precisa da morfologia e função ventricular com imagens adquiridas em eixo curto, obtendo-se uma cobertura volumétrica completa do VD, embora imagens adquiridas em orientação transaxial também possam ser usadas. Várias informações são obtidas como os volumes sistólico e diastólico final, fração de ejeção, débito cardíaco e massa miocárdica. ^99^ A massa do VD é medida traçando as bordas epicárdica e endocárdica, e o índice de massa ventricular é normalmente calculado como a razão da massa do VD para a massa do VE.

A avaliação da geometria do septo interventricular também é avaliada na ressonância e a sua projeção para a esquerda durante a sístole denota aumento pressórico de câmaras direitas. À medida que a pressão no território pulmonar aumenta, a pressão sistólica do VD pode exceder a do VE e levar a um comprometimento do volume sistólico do VE. Em quadros de HP mais grave, a falência progressiva do VD acomete a função diastólica do VE devido à projeção do septo interventricular também durante a diástole, comprometendo o enchimento diastólico do VE, reduzindo ainda mais o débito cadíaco. A curvatura interventricular septal é uma medida efetiva da deformação geométrica envolvendo os ventrículos, existindo forte correlação entre a curvatura paradoxal do septo interventricular e a gravidade da HP. ^100^

Novas técnicas estão sendo implementadas como a avaliação do *strain* miocárdico, imagem de contraste de fase ( *phasecontrast* ) e o estudo do realce tardio com possível aplicação nos casos de pacientes com HP, permitindo a análise da função miocárdica regional, repercussão hemodinâmica e extensão do comprometimento do VD. ^101^

## 4. Cardiotoxicidade Induzida por Radioterapia

### 4.1. Papel da Ecocardiografia

#### 4.1.1.Epidemiologia

A RT ocupa espaço importante no tratamento do câncer. Nos EUA, mais de 3 milhões de pacientes sobreviventes de câncer foram submetidos à RT em 2016, número correspondente a 29% da totalidade dos sobreviventes após 5 anos do diagnóstico inicial. A proporção de pacientes com câncer submetidos à RT torácica chega a 45,6% se agruparmos os pacientes com neoplasia de mama e linfoma. Vários estudos demonstram incidência aumentada de DAC, IAM e morte súbita cardíaca em pacientes submetidos à RT, em especial nos pacientes com linfoma de Hodgkin ou neoplasia de mama, reforçando o potencial de efeitos colaterais desse tratamento. ^102^^,^^103^

Denomina-se doença cardíaca induzida por radiação (DCIR) os diversos efeitos deletérios no sistema cardiovascular gerados pela dosagem cumulativa total de RT e potencializados pela QT adjuvante. Seu espectro é altamente variável e engloba o acometimento de qualquer estrutura do sistema cardiovascular, sendo a manifestação tardia muito mais frequente. Perfazem as complicações agudas, geralmente mais sutis e clinicamente menos importantes, os quadros de miopericardite e de pericardite. Já os achados tardios, que se apresentam anos ou décadas após sua a exposição, costumam ser clinicamente mais relevantes e incluem os quadros de pericardite crônica, valvulopatias, vasculopatia de grandes e médios vasos (aorta em porcelana e estenose carotídea), miocardiopatias (formas dilatada e restritiva), doenças do sistema de condução e DAC. ^104^ É farta a literatura demonstrando que a exposição à radiação, especialmente do tórax esquerdo, está associada a um aumento da mortalidade cardiovascular, atribuída principalmente à DAC e ao maior risco de desenvolvimento de insuficiência cardíaca. ^105^^,^^106^ Os fatores de risco mais relevantes ao desenvolvimento de DCIR estão ilustrados na Tabela 4 .

**Tabela 4:** Fatores de risco para doença cardíaca induzida pela radioterapia (RT)

FATORES DE RISCO PARA DOENÇAS CARDÍACAS INDUZIDA PELA RT	
	Idade < 50 anos
	Dose cumulativa de radiação > 30 Gy
	Altas doses fracionadas de radiação > 2 Gy/dia
	Presença e extensão do tumor próximo ao coração
	Uso concomitante de quimioterápicos (principalmente antracíclicos)
	Fatores de risco para doença cardiovascular (HAS, DM, DLP, tabagismo)
	Doença cardiovascular preexistente
	Inadequada proteção da área cardíaca
	Irradiação na região anterior ou hemitórax esquerdo
**Definição de alto risco: exposição à radiação torácica em região anterior ou torácica esquerda adicionalmente a pelo menos um dos fatores listados**	

RT: radioterapia; HAS: hipertensão arterial sistêmica; DM: diabetes melito; DLP: dislipidemia.

#### 4.1.2. Fisiopatologia

Os cardiomiócitos são relativamente resistentes ao dano provocado pela radiação, pelo seu estado pós-mitótico. Entretanto, células endoteliais continuam sensíveis à radiação, e a fisiopatologia da maioria das formas de DCIR parece estar relacionada a essas células. Outros mecanismos perpetuadores da agressão celular também parecem estar relacionados, como a isquemia e a hipóxia tecidual, além da agressão direta manifestada por aterosclerose acelerada, trombose, indução ao estresse oxidativo, miocitólise e ativação de mecanismos neuro-humorais levando à aterosclerose e, finalmente, à fibrose. ^107^

#### 4.1.3. Avaliação Inicial e Seguimento

Em se tratando de pacientes oncológicos, a avaliação complementar cardiovascular é classicamente ditada pelo *status* sintomático ou pela presença de achados sugestivos no exame físico. A ecocardiografia assume um papel central na avaliação da morfologia e função do coração e representa a primeira modalidade de imagem na maioria dos casos.

**Figura 8:**
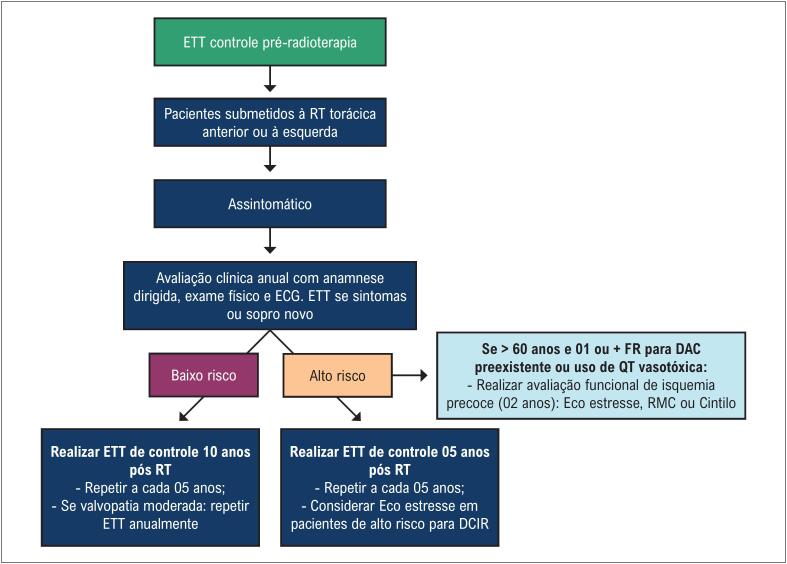
Algoritmo de acompanhamento evolutivo ecocardiográfico após radioterapia torácica.

Em relação ao seguimento pós-tratamento com RT, recomendam-se avaliações clínicas anuais contemplando anamnese dirigida, exame clínico e eletrocardiograma de repouso. Na presença de sintomas e/ou novos achados sugestivos ao exame físico, sugere-se a realização de novo ecocardiograma transtorácico (ETT). Nos pacientes que permanecem assintomáticos e categorizados como de baixo risco para desenvolvimento de DCIR, o ETT de vigilância é recomendado após 10 anos da exposição e reavaliações seguintes a cada 5 anos. Já em indivíduos assintomáticos, no entanto, classificados como de alto risco, sugere-se a antecipação da avaliação, incluindo a pesquisa de isquemia miocárdica por meio de teste funcional não invasivo, conforme ilustrado na Figura 8 . ^104^^,^^108^^,^^109^

#### 4.1.4. DCIR e o Papel do Ecocardiograma

##### 4.1.4.1. Doença Pericárdica e Miocárdica Induzida pela Radiação

A doença pericárdica é descrita como o efeito colateral mais comum da RT torácica, surgindo habitualmente algumas semanas após o tratamento. Novos protocolos, incluindo doses mais baixas e implementação de técnicas cardioprotetoras,diminuíram a incidência de 20% para 2,5%, guardando correlação proporcional à dose e ao volume exposto durante o tratamento. ^103^^,^^104^ A pericardite geralmente é autolimitada. No entanto, 10% a 20% dos pacientes desenvolvem pericardite crônica ou constritiva 5 a 10 anos após o tratamento, o que sinaliza para um prognóstico mais reservado. ^109^ O ETT, além do diagnóstico, permite a quantificação de derrames pericárdicos e auxilia pericardiocentese. Uma das grandes vantagens do método é permitir a análise da sua fisiologia constrictiva, caracterizada por achados ecocardiográficos como espessamento pericárdico, *bounce* septal, padrão de enchimento diastólico restritivo, variação inspiratória significativa do influxo mitral (> 25%), pletora de veia cava inferior, redução do *strain* circunferencial e reversão do fluxo diastólico expiratório nas veias hepáticas. O impacto na sobrevida tem sido atribuído ao encontro concomitante de outras lesões cardíacas associadas à radiação, sendo comumente acompanhado por fibrose miocárdica, estenose prematura da artéria coronária e lesões valvares importantes.

##### 4.1.4.2. Doença Coronária Induzida por Radiação

Habitualmente, o tratamento de linfoma de Hodgkin e câncer de mama inclue a RT torácica, resultando na exposição dos tecidos cardíacos à radiação. A incidência cumulativa de doença arterial coronariana induzida por radiação (DACIR) é estimada em quase 60% em sobreviventes do linfoma de Hodgkin, com um risco relativo de 3,2 vezes maior em comparação com a população em geral. ^109^ Já em relação ao câncer de mama, o mais frequente entre as mulheres, metanálises mostraram que o risco relativo de desenvolver DACIR é mais alto em pacientes que recebem RT torácica esquerda. ^110^

Portanto, pacientes com neoplasias torácicas têm um risco consideravelmente maior de desenvolver DACIR em comparação com a população em geral, em especial na presença de fatores de risco como dose cumulativa utilizada (em geral > 30 Gy), idade precoce durante a exposição, volume de tecido acometido, falta de proteção cardíaca e presença de fatores de risco cardiovasculares tradicionais preexistentes. ^104^^,^^105^^,^^109^

Apesar de a DCIR se manifestar tardiamente, a DAC, em especial, pode ter surgimento mais precocemente, já a partir do quinto ano pós-exposição. ^105^ É sabido que as lesões coronarianas induzidas pela radiação exibem, em sua maioria, acometimento ostial e proximal, caracteristicamente envolvendo o tronco da coronária esquerda, artéria descendente anterior ou a artéria coronária direita. ^107^^,^^111^ A RT predispõe ou acelera a aterosclerose, inclusive naqueles pacientes sem fatores de riscos cardiovasculares clássicos, e pode ter como primeira apresentação clínica a morte súbita. Dessa forma, considerando-se a alta morbimortalidade, apresentação clínica heterogênea e por vezes atípica, advoga-se a realização de rastreamento por meio do ecocardiograma de estresse (ETT estresse), utilizando-se estresse físico ou farmacológico (dobutamina) quando pertinente. Esse método funcional não invasivo oferece vantagens como acurácia satisfatória, ausência de radiação, relativo baixo custo e disponibilidade.

##### 4.1.4.3. Doença Valvular Induzida pela Radiação (DVIR)

Relata-se maior prevalência de DVIR nos subgrupos de portadores de linfoma de Hodgkin (17%) e neoplasia de mama (4,2%). Assim como na DACIR, o seu risco está relacionado com dose total de radiação e campo de exposição.

O acometimento valvar com repercussão hemodinâmica geralmente ocorre após 10 anos da RT e mais comumente acomete as valvas do lado esquerdo do coração. A valva aórtica é mais frequentemente acometida que a mitral. O surgimento de regurgitações valvares é mais precoce que as lesões estenóticas, que costumam aparecer cerca de 20 anos após a RT. Os achados ecocardiográficos são variáveis e incluem desde alterações mínimas sem disfunção valvar associada, até fibrose difusa, espessamento e calcificação. São característicos o acometimento de qualquer componente do aparato valvar, poupando comissuras, permitindo assim diferenciação sobretudo da etiologia reumática. O comprometimento da cortina mitroaórtica (espessamento e calcificação) é um achado característico pós-RT e está associado à pior sobrevida a longo prazo. ^103^^,^^108^

Com a progressão dos sintomas e gravidade das lesões, pode haver necessidade de intervenção cirúrgica ou manejo percutâneo. Técnicas ecocardiográficas diferenciadas como o eco 3D e o eco transesofágico (ETE) auxiliam na melhora da avaliação anatômica e funcional, especialmente nas lesões valvares de maior repercussão hemodinâmica e estrutural. Recomenda-se, entretanto, cuidado na realização do ETE em pacientes submetidos à RT torácica, dada a possibilidade de comprometimento associado da estrutura esofágica nesses pacientes.

### 4.2. Arterite Induzida por Radiação – Arterite Actínica

A arterite induzida por radiação, também chamada de arterite actínica, se dá por alteração da estrutura da parede arterial e aparecimento de estenoses ou oclusões arteriais. Esses fenômenos podem resultar em um processo aterosclerótico acelerado, de caráter inflamatório, por lesão das celúlas endoteliais, podendo acometer artérias de médio ou grande calibre. Nos casos de radioterapida para tumores da cabeça e pescoço, as artérias comprometidas podem estar nos segmentos intra e extracranianos.

A estenose carotídea induzida pela RT teve sua evolução estudada por Cheng et al, ^112^ estes autores demonstraram que, comparados com controles não submetidos à irradiação, houve progressão anual do grau de estenose, de menor que 50% para maior que 50%, em 15,4% *versus* 4,8% (pacientes não irradiados). Nesse estudo, não houve diferença no surgimentos de sintomas ou mortalidade entre os grupos estudados. Porém, tais alterações podem levar, segundo outros autores, ao aparecimento de sintomas cerebrovasculares, tendo sido demonstrado que pacientes sintomáticos, com lesões carotídeas pós-RT, exibem fluxo cerebral reduzido. ^113^

Arteriopatia periférica, apesar de mais rara, também tem sido relacionada após a radioterapia, como por exemplo após o tratamento de cânceres da cérvix uterina, com acometimento de artérias ilíacas e femorais; os sintomas podem ser de isquemia crônica, porém, também podem ocorrer casos de oclusão arterial aguda. ^114^

#### 4.2.1. Diagnóstico

O diagnóstico da estenose carotídea pode ser feito clinicamente, inferido pela ausculta de sopro na região do pescoço que recebeu a RT. Os exames de imagem, incluindo a ultrassonografia vascular, a angiotomografia, a ressonância nuclear magnética ou a angiografia convencional contrastada, podem, além de detectar e estenose, também quantificá-la. ^115^

A ultrassonografia vascular tem vantagens por ser não invasiva, de baixo custo, sem uso de contraste nefrotóxico, podendo assim ser utilizada, se de forma segura, no seguimento a médio e longo prazo dos pacientes.

#### 4.2.2. Características Ultrassonográficas

**Figura 9:**
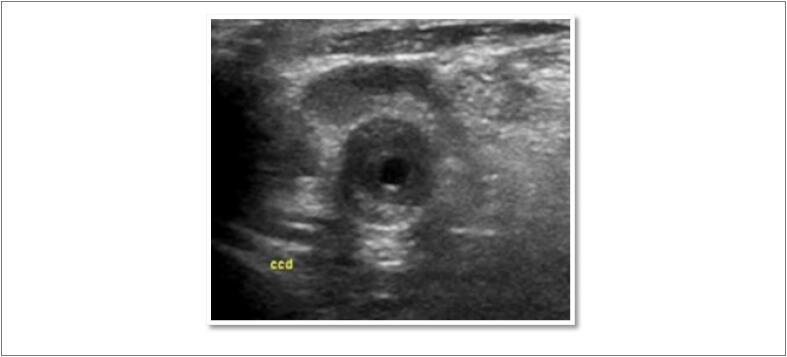
Artéria carótida comum direita em corte transverso com importante espessamento da camada mediointimal levando à grande redução luminal.

As lesões arteriais induzidas pela radiação ionizante podem ocorrer nas diversas artérias do corpo, tendo sido descritas, principalmente, nos troncos supra aórticos (artérias carótidas e subclávias). Após o primeiro ano do uso da RT para o tratamento de cânceres localizados na cabeça e no pescoço, há um espessamento acelerado e progressivo da camada mediointimal de ambas as artérias carótidas, podendo levar a importante redução luminal. ^116^ Wilbers et al. ^117^ encontraram resultados semelhantes após 7 anos do uso da radioterapia nessa região. Estudo realizado com jovens submetidos à radioterapia, em um seguimento de 20 anos, mostrou os seguintes achados: maior prevalência de placas ateroscleróticas no lado irradiado do que no contralateral (18% versus 2%), maior espessura mediointimal (EMI) em pacientes tratados que nos controles e um aumento linear da EMI pelo tempo desde a realização da RT ^118^ ( Figuras 9 e 10 ).

**Figura 10:**
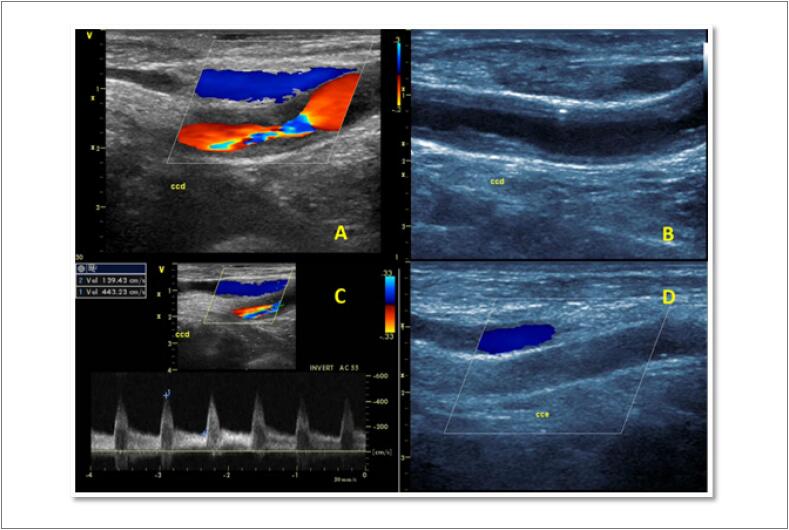
Paciente com arterite actínica, 6 anos após a radioterapia. A. Estenose maior que 70% da artéria carótida comum direita ao Doppler colorido – observa-se grande redução luminal e turbulência do fluxo. B. Artéria carótida comum direita com grande aumento da espessura mediointimal (EMI) ao modo B causando com redução luminal. C. Doppler pulsado com elevação da velocidade de fluxo. D. Carótida comum esquerda ocluída.

**Figura 11:**
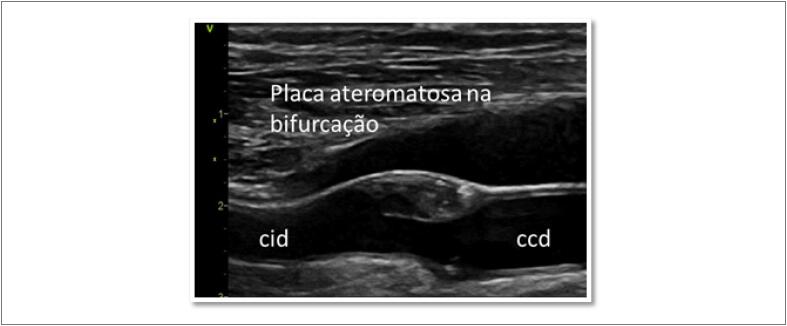
Placa aterosclerótica na bifurcação carotídea.

É frequente o acometimento da artéria carótida comum por um processo aterosclerótico acelerado, que também pode ser observado na artéria carótida interna. O diagnóstico diferencial apenas pela imagem ultrassonográfica nem sempre é possível, sendo que a história de radioterapia na região acometida é fundamental para que o diagnóstico seja estabelecido. A doença aterosclerótica e a arterite de Takayassu são os principais diagnósticos diferenciais. Na doença aterosclerótica, em geral, o acometimento se dá mais comumente na bifurcação carotídea e na carótida interna, mas também pode ocorrer na carótida comum ( Figura 11 ). Nos casos da arterite actínica, o acometimento da artéria carótida comum é mais evidente, apresentando uma aterosclerose mais difusa, acentuada e progressiva. ^112^^,^^115^

**Figura 12:**
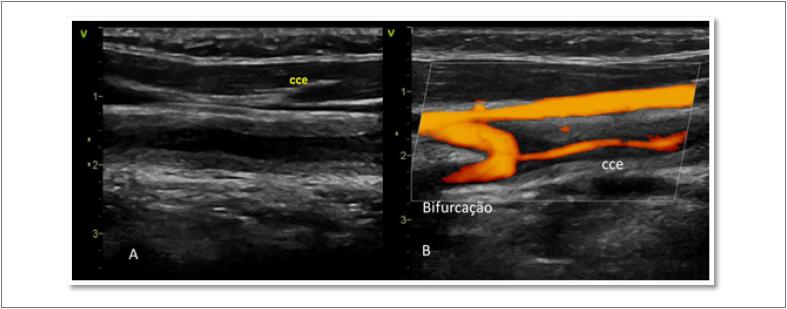
Arterite de Takayassu, sexo feminino, 17 anos de idade. A. Corte longitudinal da artéria carótida comum esquerda mostra importante espessamento concêntrico da parede. B.119 Imagem ao powerDoppler com grande redução luminal da artéria carótida comum, com lúmen preservado a partir da bifurcação carotídea.

Na arterite de Takayassu, há um espessamento homogêneo e concêntrico que pode levar à estenose significativa e oclusão do vaso, podendo também haver dilatações (aneurismas). Caracteristicamente acomete a carótida comum até a bifurcação, mas poupa a carótida interna ^119^ ( Figura 12 A -B ).

#### 4.2.3. Seguimento das Estenoses Arteriais

Em função do caráter progressivo das estenoses arteriais induzidas pela radiação, sugere-se um seguimento mais rigorosos por métodos de imagem a fim de detectar as estenoses significativas passíveis de tratamento. Inicialmente, a ultrassonografia vascular pede ser realizada anualmente e ter seu uso individualizado, com intervalos menores caso haja mais rápida progressão da doença e também após o tratamento. ^120^

**Figura 13:**
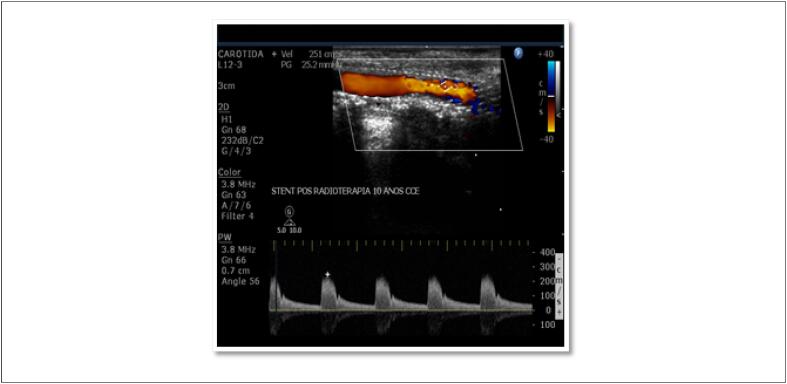
Paciente após 10 anos de radioterapia, com stent na artéria carótida comum esquerda; corte longitudinal demostrando a hiperplasia intimal e fluxo intrastent ao Doppler colorido; Doppler pulsado demonstrando aumento da velocidade local.

A endarterectomia de carótida (ECA) e a abordagem via endovascular (angioplastia carotídea com STENT) se mostraram opções eficazes no tratamento das estenoses induzidas pela RT. ^121^ A extensão da doença e a grande área arterial acometida podem favorecer fenômenos embólicos durante o tratamento com STENT e levar ao surgimento de eventos cerebrovasculares. Além disso, os STENTS podem apresentar hiperplasia intimal com recorrência da estenose ( Figura 13 ). O mecanismo que leva ao surgimento da hiperplasia intimal intra STENT nesses pacientes parece estar relacionado à proliferação de células musculares lisas. ^122^ A recorrência da estenose tem se mostrado um problema após o tratamento endovascular, aparentando ser maior nos pacientes com radioterapia prévia quando comparados com os controles. Comparados com os pacientes submetidos à ECA, a taxa de reestenose acima de 50% é maior nos tratados com STENT. ^123^

O acometimento de outras artérias, que não as artérias cervicais, deve ser individualizado, tendo, na literatura, casos relatados com tratamento cirúrgico e, via endovascular, angioplastia com STENT. ^114^^,^^124^

### 4.3. Avaliação Cardiovascular após Radioterapia | Papel da Medicina Nuclear

#### 4.3.1. Avaliação e Seguimento de DAC após RT

Lind et al. ^125^ avaliaram 69 mulheres submetidas à RT torácica nas doses de 46 a 50 Gy, com frações de até 2 Gy por sessão, utilizando blindagem cardíaca adequada. Estas foram submetidas à cintilografia de perfusão miocárdica em repouso com sestamibi-^99m^Tc ou tetrofosmin-^99m^Tc antes e com 6, 12 e 18 meses após RT. Com 6 meses foram observadas alterações perfusionais, em especial no território da artéria coronária descendente anterior. ^125^ Este achado também foi corroborado por Hardenbergh et al., ^126^ que mostraram alterações perfusionais em cerca de 60% dos indivíduos 6meses após irradiação torácica. ^126^ A análise da função ventricular ao ecocardiograma mostrou que somente um desses pacientes apresentou redução de mais de 10% da FEVE. Mesmo associado ao dano de microcirculação, os autores destacam a importância deste achado, que pode levar à disfunção diastólica e sistólica, subsequentemente.

As alterações coronárias tanto micro quanto macrovasculares são sequelas tardias reconhecidas da RT torácica. É conhecida a importância de uma triagem regular para os efeitos coronários da RT. No entanto, ainda não há regimes de rastreamento específico para essa população, mesmo diante do grande arsenal de métodos não invasivos disponíveis. ^127^^,^^128^

### 4.4. Avaliação de DAC após Radioterapia – Papel da Tomografia Computadorizada

A cardiotoxicidade induzida pelo tratamento radioterápico apresenta vários mecanismos fisiopatológicos que desencadeiam diversas injúrias ao sistema cardiovascular: nas artérias coronárias, podem ocorrer um efeito vasoespástico direto, lesão endotelial ou alterações no metabolismo lipídico e aterosclerose prematura consequente. O risco de eventos coronários subsequentes é proporcional à dose de radiação.

A elevada acurácia na detecção de DAC pela angiotomografia coronária confere ao método o potencial de fornecer informações precisas na suspeita de DAC nos pacientes com câncer, especialmente na dor torácica e nos sintomas de insuficiência cardíaca potencialmente relacionados à etiologia isquêmica.

Numerosos estudos demonstram incidência aumentada de DAC, infarto agudo do miocárdio e morte súbita cardíaca em pacientes submetidos à radioterapia, em especial nos pacientes com linfoma de Hodgkin ou neoplasia de mama, reforçando o potencial nocivo deste tratamento. As diretrizes atuais sugerem triagem para DAC no período de 5 a 10 anos após a irradiação torácica como vigilância para cardiotoxicidade tardia. ^129^

Diversos estudos incluindo a tomografia confirmam esses achados clínico-epidemiológicos em pacientes com linfoma de Hodgkin. Mulrooney et al. ^130^ identificaram placas pela angiotomografia coronária em 39% dos sobreviventes. Já Girinsky et al. ^131^ encontraram placas em 26% dos pacientes submetidos à radioterapia num estudo prospectivo com seguimento médio de 9,5 anos incluindo 179 pacientes. Na análise multivariada, a dose de radiação aplicada na origem das artérias coronárias associou-se ao achado de placas. Van Rosendael et al. ^132^ compararam 71 pacientes submetidos à radioterapia com 237 controles não irradiados pareados para múltiplos fatores de risco para DAC e encontraram maior prevalência de placas, doença mais proximal, maior grau de estenose e número de vasos com lesões importantes no grupo submetido à radioterapia.

### 4.5. Avaliação de DAC após Radioterapia – Papel da Ressonância Magnética

A RMC tem excelente acurácia no diagnóstico de isquemia miocárdica quando associada ao estresse farmacológico, e é considerada o padrão-ouro na avaliação de infarto e viabilidade miocárdica, além da análise da função ventricular, apresentando valor prognóstico na predição eventos cardiovasculares.

O estudo CE-MARC comparou a pesquisa de isquemia da perfusão sob estresse pela RMC com a da perfusão sob estresse pela cintilografia em 628 pacientes submetidos à cinecoronariografia como referência, em uma população com prevalência de DAC obstrutiva de 39%. ^133^ Ambos os métodos apresentaram especificidades semelhantes (RMC 83,4% x SPECT 82,6%, p = 0,916), porém a RMC foi significativamente mais sensível para o diagnóstico de isquemia miocárdica (RMC 86,5% x SPECT 66,5%, p < 0,0001).

A técnica de realce tardio, em que as imagens são adquiridas após a infusão de gadolínio e sem a necessidade de agentes estressores, é considerada o principal meio não invasivo para a detecção de fibrose miocárdica. A análise da extensão do infarto do miocárdio prediz a viabilidade miocárdica relacionada ao segmento em investigação. A presença e a extensão do infarto pela RMC apresenta estabelecido valor prognóstico, inclusive a presença de infartos desconhecidos em pacientes submetidos à RMC está associada com pior prognóstico cardiovascular e mortalidade. ^134^

## 5. Tumores e Massas Cardíacas

### 5.1. Contribuição da Ecocardiografia

Massas cardíacas costumam ser desafiadoras ao diagnóstico ecocardiográfico. Trombos, vegetações e pseudotumores representam 75% dos casos. Os tumores cardíacos primários (TCP) propriamente ditos são extremamente raros com frequência estimada de 0,02%, podendo ser benignos ou malignos quanto à histologia. Já as metástases cardíacas são até 100 vezes mais frequentes. Recentemente, a Organização Mundial da Saúde atualizou a classificação de tumores cardíacos e pericárdicos. ^135^

O ecocardiograma transtorácico (ETT) avalia localização, morfologia, dimensões, ecogenicidade, mobilidade, extensão, calcificações, tipo de inserção e presença ou não de derrame pericárdico (DP). ^136^ As diferentes modalidades de Doppler permitem avaliação do fluxo sanguíneo e consequente repercussão hemodinâmica, como obstrução ao fluxo ou disfunção valvar. O mapeamento de fluxo em cores pode revelar apresença de vascularização em certos tipos de tumores, assim como o uso de contrastes de perfusão, permitindo identificação e diferenciação entre trombos e tumores. Em geral, trombos não realçam e são bem delineados na cavidade contrastada. O contraste auxilia também na diferenciação entre tumores benignos e malignos. ^137^ Tumores malignos tendem a apresentar realce completo, enquanto tumores benignos apenas parcialmente. A análise da perfusão pós-impulso ( *replenishment* ) pode ser qualitativa (visual) ou quantitativa, por meio de curvas de captação de intensidade tempo-sinal e através de mapas paramétricos (codificação colorida da intensidade de sinal). A diferença entre o nível máximo de intensidade de *pixel* estacionário pós-impulso ( *A* ) da massa e o miocárdio vizinho pode ser calculada (Delta *A*_massa-miocárdio_). A sensibilidade e a especificidade relatadas do Delta *A*_massa-miocárdio_ em diferenciar um trombo de tumores benignos e malignos foram 93% e 100%, respectivamente, com valores de corte de 23,2 dB e de 100% e 97%, com valor de corte de 0,45 dB. ^138^ Estimativas do volume, velocidade e fluxo sanguíneo microvascular das massas também podem ser obtidas. Imagens com *strain* podem diferenciar a natureza e o padrão contrátil de certos tipos de tumores, como fibroma e rabdomioma. Rabdomiomas apresentam deformação oposta à do miocárdio, enquanto fibromas não apresentam deformação. ^139^ O ETE tem maior sensibilidade (>95%) e melhor resolução acústica que o ETT, permitindo melhor caracterização de tumores e massas quanto à localização, inserção e relação com a veia cava, especialmente nas lesões atriais e valvares. O ETE tem papel importante em casos com embolização sistêmica, na avaliação intraoperatória de ressecção tumoral, bem como nas lesões ventriculares em pacientes com janela acústica torácica limitada. O ecocardiograma 3D em tempo real é a mais nova ferramenta de avaliação de massas cardíacas, fornecendo dados mais acurados sobre volume, morfologia e relação com estruturas vizinhas, além de aspectos como local e tipo de inserção, homogeneidade, vascularização, calcificação e áreas de necrose. ^140^

Toda massa cardíaca encontrada ao ecocardiograma deve ser colocada no contexto clínico e epidemiológico. Idade, sexo, sintomatologia e doença oncológica concomitante são elementos importantes para o diagnóstico. Artefatos devem ser excluídos analisando múltiplos planos de corte e fazendo ajustes nos parâmetros de imagem. Valva de Eustáquio e crista terminalis proeminentes, rede de Chiari e crista coumadínica são variantes anatômicas que podem ser mal interpretadas e confundidas com massas. Escrescências de Lambl são estruturas filiformes que variam entre 3 e 5mm, encontradas no local de fechamento das valvas, a jusante do fluxo. Não causam disfunção valvar, mas podem embolizar. Hipertrofia lipomatosa do septo interatrial se caracteriza por infiltração de gordura madura, que poupa a fossa oval conferindo-lhe típica forma de haltere. Tumores amorfos calcificados são massas não neoplásicas heterogêneas, hiperecoicas, com áreas hipo ou isoecoicas, de mobilidade variável, frequentemente associados à calcificação do anel mitral e que podem ser fontes de embolias. ^141^ Cistos pericárdicos são hipoecoicos, usualmente loculados, com dimensões variadas e adjacentes à borda cardíaca.Trombos podem ser encontrados em qualquer cavidade e variam em tamanho, morfologia e mobilidade, mas não costumam ocorrer fora de condições predisponentes. Quando no interior do átrio esquerdo, ocorrem principalmente em pacientes que apresentam contraste espontâneo, fibrilação atrial, estenose ou prótese mitral. No átrio direito (AD), são relativamente comuns em pacientes oncológicos com cateter de longa permanência e estado de hipercoagulabilidade. Trombos migrantes são vistos no lado direito do coração em pacientes com TVP e contexto de embolia pulmonar. Trombo no VE associa-se a alterações da contratilidade segmentar e cardiomiopatia dilatada. Vegetações acometem principalmente as valvas cardíacas esquerdas, apresentam grande mobilidade e movimentação independente. São encontradas na superfície atrial das valvas atrioventriculares e na superfície ventricular das valvas semilunares, frequentemente causando disfunção valvar, em um contexto de endocardite.

#### 5.1.1. Tumores Cardíacos Primários Benignos (TCPB)

Representam 85% a 90% de todos os TCP. Destes, cerca de 50% são mixomas. ^136^ Rabdomioma é o tipo mais frequente na infância, correspondendo a aproximadamente 50% dos casos, seguido por fibroma e mixoma. ^142^

##### 5.1.1.1. Mixomas Cardíacos

São mais frequentes entre 30 e 60 anos, apresentando sintomas constitucionais, obstrutivos ou embólicos. Localizam-se no AE em 75% dos casos, 20% no AD e 5% nos ventrículos. Tipicamente, caracterizam-se como massas polipoides oscilantes, fixadas no septo interatrial através de pedículo estreito. São heterogêneos, com núcleos ecogênicos, áreas císticas e superfícies lisas. Dependendo do tamanho, podem obstruir o fluxo da valva atrioventricular. Mixomas papilares são menores, com aparência esticada, superfície irregular, maior mobilidade e maior risco de embolização. O ETE (2D ou 3D) e o uso de contrastes de perfusão auxiliam na caracterização desses tumores. Mixomas cardíacos podem ser recorrentes, quando associados ao Complexo de Carney. Nesses casos, o ETT é a técnica de imagem ideal para o seguimento. ^143^

##### 5.1.1.2. Fibroelastomas Papilares

São pequenos tumores avasculares (em geral, < 10mm), sésseis ou pedunculados, arredondados, gelatinosos, com múltiplas projeções papilares estreitas, alongadas e ramificadas. Lembram anêmonas do mar quando colocados sob a água. Essas características lhes conferem friabilidade e predisposição para adesão de trombos. ^144^ Episódios de embolização são mais comuns em tumores maiores que 10mm e podem ocorrer a partir de trombos aderidos ou da fragmentação de papilas. Geralmente são únicos, móveis e fixos a qualquer superfície endocárdica por um pedículo curto. Acometem a superfície endocárdica valvular em 77% dos casos e ocorrem principalmente em idosos. Comumente são vistos na porção média dos folhetos das valvas cardíacas esquerdas. Na valva aórtica, costumam protrair no lúmen arterial e na mitral, na superfície atrial. ^144^^,^^145^ Ao ecocardiograma, exibem mobilidade independente, bordas pontilhadas e cintilantes e pedículo central estreito. Usualmente, não causam disfunção valvar. O ETE apresenta papel importante na avaliação desses tumores.

##### 5.1.1.3. Rabdomiomas

Geralmente são diagnosticados no primeiro ano de vida ou na vida fetal. Associam-se à esclerose tuberosa, costumam ser múltiplos e tendem a regredir espontaneamente. ^146^ Acometem principalmente o septo interventricular, paredes ventriculares e valvas atrioventriculares. Aparecem como múltiplas massas nodulares ou pedunculadas, lobuladas, bem delimitadas, homogêneas e hiperecogênicas em relação ao miocárdio circundante. Apresentam deformação oposta à do miocárdio nas imagens com *strain* . ^139^

##### 5.1.1.4. Fibromas Cardíacos

Raramente ocorrem em adultos. Aproximadamente 3% a 5% são associados à síndrome de Gorlin (síndrome do carcinoma basocelular). ^147^ Caracterizam-se como massa sólida intramural, única, hiperecogênica, bem delimitada, não contrátil, com calcificações e que se estendem para a cavidade do VE. Podem provocar arritmias, disfunção ventricular e obstrução ao fluxo. Não apresentam deformação nas imagens com *strain* . ^139^

##### 5.1.1.5. Lipomas Cardíacos

São massas de tecido adiposo encapsulado, localizadas principalmente no subendocárdio do VE, mas ocorre em outras câmaras, no pericárdio e valvas cardíacas, podendo atingir vários centímetros. Apresentam-se com base larga, homogêneas, imóveis e sem calcificação. Na cavidade, são hiperecoicase, no pericárdio, hipoecoicas. ^148^

##### 5.1.1.6. Teratomas

São tumores de células germinativas encontrados em lactentes, crianças e fetos. ^147^ Podem ser maduros ou imaturos e raramente ocorrem em adultos.Localizam-se preferencialmente no espaço pericárdico e podem provocar tamponamento cardíaco ou compressão de estruturas adjacentes. ^149^ Caracterizam-se como massas heterogêneas contendo áreas sólidas, císticas e calcificações, frequentemente acompanhadas de derrame pericárdico.

##### 5.1.1.7. Hemangiomas Cardíacos

São tumores vasculares raramente encontrados. Ocorrem em qualquer idade, inclusive na vida fetal. Localizam-se principalmente no VE e AD, medindo entre 2 e 10 cm na maioria dos casos. São pedunculados e móveis na metade dos casos. Aparecem como massas bem circunscritas, císticas ou sólidas, uni ou multilobuladas, dependendo do tipo: cavernoso, capilar ou arteriovenoso. ^150^

##### 5.1.1.8. Paragangliomas Cardíacos

São tumores neuroendócrinos muito raros. Associam-se a várias síndromes, como doença de Von HippelLindau, neurofibromatose I, complexo de Carney, entre outras. Podem ser benignos ou malignos e hormonalmente ativos ou inativos. São extremamente vascularizados e não encapsulados. Ocorrem mais frequentemente entre a 4ª e 5ª décadas de vida e se localizam principalmente no espaço pericárdico, junto ao sulco atrioventricular, raiz dos grandes vasos e AE. Caracterizam-se como massas ecogênicas, com base larga, usualmente de forma ovoide, bem delimitadas, com tamanho médio de 5cm, circundando artéria coronária, podendo comprimir estruturas adjacentes. ^151^

##### 5.1.1.9. Schwanomas Cardíacos

São tumores de crescimento lento, extremamente raros, com apenas 25 casos relatados na literatura inglesa (18 intracardíacos e 7 intrapericárdicos). Originam-se do plexo cardíaco ou de ramos do nervo vago e envolvem mais o lado direito do coração. São benignos, mas podem assumir comportamento maligno. Constituem massas ecogênicas, hipodensas, não pedunculadas, delimitadas, podendo atingir vários centímetros, causando sinais e sintomas relacionados à compressão de estruturas adjacentes. ^152^

#### 5.1.2. Tumores Cardíacos Primários Malignos (TCPM)

São extremamente raros com frequência de 0,008%. Correspondem a 10% a 15% de todos os TCP. São representados por diferentes tipos de sarcomas, linfomas e mesoteliomas. Acometem principalmente adultos na quinta década de vida. Sarcomas representam 65% a 95% dos casos em adultos e 70% em crianças e adolescentes (< 18 anos). ^143^^,^^154^ Angiossarcoma é o tipo mais comum. Geralmente produz sintomas como dispneia e dor torácica pleurítica. ^155^

##### 5.1.2.1. Angiossarcomas

Apresentam comportamento agressivo e podem metastatizar. Localizam-se preferencialmente no AD. Apresentam-se como volumosas massas ecogênicas lobuladas, homogêneas, imóveis, não pedunculadas e sem hastes, com ampla base de inserção endocárdica e bordas intracardíacas lisas. Devido à sua localização junto ao sulco atrioventricular, costumam causar obstruções na valva tricúspide e, na maioria dos casos, acometem o pericárdio originando derrames hemorrágicos. Devido, em parte, à sua vascularização esguia, não realçam significativamente com o uso de contrastes de perfusão. A sensibilidade do ETT em sua identificação é de 75%. ^155^

###### 5.1.2.1.1. Sarcomas Indiferenciados

Tipicamente se localizam no AE, podendo mimetizar mixomas. Tendem a envolver a valva mitral causando obstrução ao fluxo. Apresentam bases largas, certa mobilidade, aspecto macio e homogêneo. ^156^

###### 5.1.2.1.2. Rabdomiossarcoma

É o tipo mais comum em crianças. Usualmente surge nas paredes ventriculares e frequentemente interferem na função valvar devido ao crescimento intracavitário. ^148^ Tendem a ocorrer em mais de um local, podendo ocasionar obstrução em múltiplos níveis. Crescem rapidamente e costumam invadir o pericárdio. ^136^

###### 5.1.2.1.3. Leiomiossarcomas

Surgem mais comumente em vasos como a veia cava inferior e artéria pulmonar, mas podem ocorrer no AE.

##### 5.1.2.2. Linfomas Cardíacos Primários

Representam aproximadamente 27% dos TCPM, sendo o linfoma nãoHodgkin de grandes células B o tipo mais comum. ^153^ Predominam na 6ª década de vida, mas ocorrem entre a 1ª e 9ª décadas, sendo frequentes em pacientes imunocomprometidos. Localizam-se principalmente no lado direito do coração e no pericárdio. Envolvem a veia cava superior em 25% dos casos e o septo interatrial em 48%. Aparecem como massas infiltrativas ou nodulares. Formas infiltrativas são homogêneas, promovendo espessamento da parede e hemodinâmica restritiva. Formas nodulares adentram a cavidade e podem comprometer o nó atrioventricular, artéria coronária direita e pericárdio. ^136^ Em 75% dos casos, mais de uma câmara está envolvida. Dependendo da localização e dimensões, pode causar obstrução ao fluxo de entrada, síndrome da veia cava superior, arritmias, bloqueio atrioventricular, síndromes restritivas ou constritivas, DP e fenômenos embólicos. O ETE (2D ou 3D) e o uso de contrastes de perfusão são úteis na caracterização desses tumores. ^157^

##### 5.1.2.3. Mesoteliomas Pericárdicos Malignos Primários

Representam 8% dos TCPM e aproximadamente 50% dos tumores primários do pericárdio. ^153^^,^^158^ São mais frequentes entre a quinta e sétima décadas de vida e apresentam baixa sobrevida. Associam-se, em muitos casos, com exposição ao asbesto e RT. ^159^ A sintomatologia é vaga e inclui sintomas de pericardite constritiva e DP com tamponamento cardíaco. Infiltração miocárdica pode ocorrer e resultar em anormalidades de condução. O ETT tem baixa sensibilidade em sua detecção. ^158^

#### 5.1.3. Tumores Cardíacos Metastáticos

São por definição malignos e sua incidência tem aumentado nas últimas décadas, chegando a 18,35% em pacientes com doença oncológica avançada. ^159^^,^^160^ Predominam entre a sexta e sétima década de vida. Metástases cardíacas podem ocorrer por extensão direta do tumor, via hematogênica, linfática e por difusão intracavitária através da veia cava inferior ou de veias pulmonares. Com exceção dos tumores do sistema nervoso central, qualquer tumor maligno pode enviar metástases para o coração, mas carcinomas de pulmão, mama, esôfago, melanoma e neoplasias hematológicas (leucemia e linfoma) são responsáveis pela maioria dos casos. ^159^^,^^161^ Melanomas malignos são os mais propensos a acometerem o coração. Pericárdio, epicárdio, miocárdio e endocárdio, em ordem decrescente de frequência, são os locais que podem ser acometidos. Quando intracavitários, localizam-se preferencialmente nas câmaras direitas. Derrames pericárdicos são frequentes e podem ser assintomáticos. Deve-se suspeitar de envolvimento cardíaco em todos os pacientes com doença oncológica que desenvolvam novos sintomas cardiovasculares. ^162^ O ETT é o método de avaliação inicial para detecção de trombos tumorais na veia cava inferior, câmaras cardíacas e presença de DP. Outras modalidades de imagem são necessárias para melhor caracterização das lesões, sua extensão e relação com estruturas vizinhas, bem como para revelar o sítio tumoral primário. ^161^

### 5.2. Contribuição da Ressonância Magnética Cardíaca

A RMC, sobretudopela propriedade de caracterização tecidual, tem se estabelecido como uma metodologia robusta na avaliação das massas cardíacas, especialmente na diferenciação entre tumores benignos e malignos. Além da caracterização tecidual, a RMC poderá fornecer importantes informações quanto a localização, dimensões, extensão e sinais de compressão ou obstrução das estruturas cardíacas. ^163^

As sequências de cinerressonância SSFP ( *steady-state-free-precession* ) são utilizadas para avaliação da morfologia e mobilidade da massa e sua relação com o miocárdio; e ainda se causa obstrução hemodinâmica às estruturas valvares. As sequências de sangue escuro pré-contraste ponderadas em T1 e T2 auxiliam na definição da relação com a anatomia adjacente, o tamanho, a extensão e a caracterização da massa. A intensidade de sinal da massa na ressonância depende do tempo de relaxamento T1 e T2. Esta intensidade de sinal deve ser avaliada em relação à intensidade de sinal do miocárdio, se é isointenso, hipointenso ou hiperintenso em relação ao sinal do miocárdio. ^164^

A saturação de gordura permite a identificação do sinal da gordura na composição da massa, uma vez que estase apresenta hiperintensa nas sequências ponderadas em T1 e T2; a saturação deste sinal sugere componente gorduroso na massa. Esta técnica permite identificar estruturas benignas como a infiltração lipomatosa do septo interatrial, tumores benignos como o lipoma e detectar a presença de gordura como componente de um lipossarcoma.

A vascularização do tumor pode ser avaliada por meio da perfusão de primeira passagem do gadolínio quanto à presença ou não de perfusão e se é isoperfundida, hipoperfundida ou hiperperfundida em relação ao miocárdio do VE; enquanto o realce tardio detecta a presença de fibrose na composição da massa. ^165^

O protocolo da RMC para avaliação de massa cardíaca ou pericárdica deve começar com cortes axiais de alta resolução, seguidos de sequências de cinerressonância para sua localização e análise da função cardíaca e da sua repercussão hemodinâmica. A seguir, de acordo com a suspeita diagnóstica, são realizados cortes sobre a massa para sua adequada caracterização tecidual. Podem ser usadas sequências ponderadas em T1 e T2, sequências com supressão de gordura para avaliar o componente lipídico da massa suspeita, perfusão de primeira passagem com gadolínio para estudo da sua vascularização e sequências de realce tardio para avaliação de fibrose.

Na suspeita de trombo, sequências de realce tardio com longo tempo de inversão também podem ser usadas para confirmar o diagnóstico. A RMC permite a diferenciação entre trombo e tumor, uma vez que, no trombo, não há perfusão pelo agente paramagnético, apresentando um aspecto muito escuro nas sequências de realce tardio, mesmo com tempo de inversão prolongado de até 600ms, podendo ser observada área adjacente de hipersinal pelo sangue circunjacente ou tardiamente apresentar leve contraste de permeio em trombos antigos, muito organizados.

#### 5.2.1. Tumores Primários Benignos

##### 5.2.1.1. Mixoma

**Figura 14:**
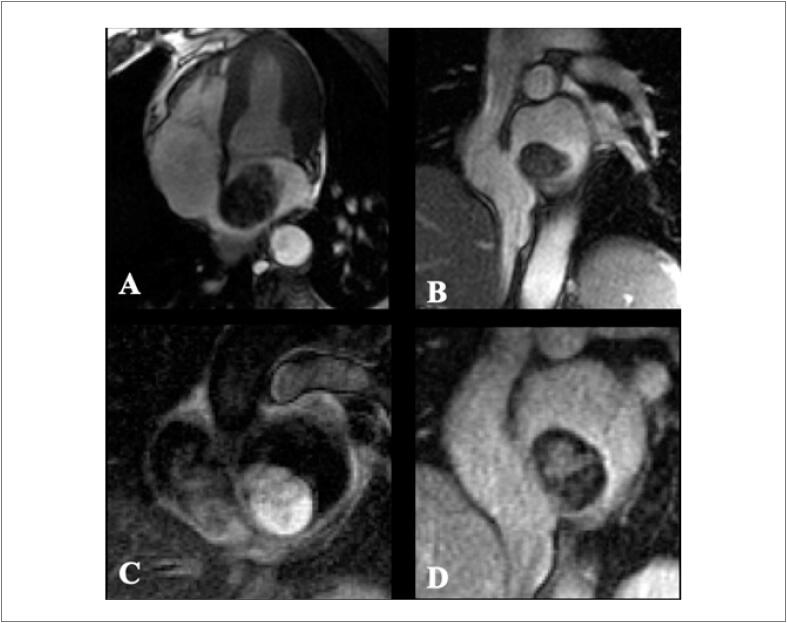
Mixoma em átrio esquerdo.

Os achados pré-contraste podem ser explicados por sua histologia: hipersinal em sequências ponderadas em T2 está relacionado com a matriz mixoide com alto conteúdo de água e polissacarídeos, enquantoos focos de hipossinal estão relacionados a áreas de hemorragia, hemossiderina (degradação da hemogolobina), calcificações e trombos de superfície. Os mixomas são isointensos nas imagens ponderadas em T1 e hiperintensos nas ponderadas em T2 ( Figura 14 ). Observamos um discreto realce nas imagens de perfusão de primeira passagem em 16% a 66% dos casos.O realce tardio tem um padrão variável e heterogêneo, podendo ser observados uma distribuição mais difusa (>50% do tumor – mais frequente) e um padrão menos intenso (< 50% do tumor). ^166^ Cerca de 10% a 20% dos mixomas apresentam calcificações e alguns podem ter trombos em sua superfície.

##### 5.2.1.2. Lipoma

**Figura 15:**
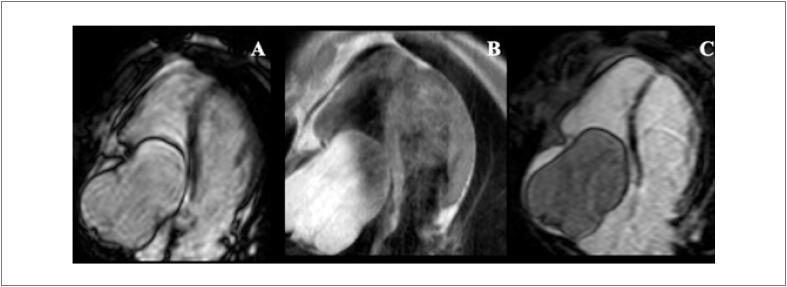
Lipoma em átrio direito. A. Cinerressonância em quatro câmaras mostrando grande lipoma em átrio direito. B. Imagem ponderada em T1 demonstrando que o lipoma é hiperintenso. C. Ausência de realcetardio local.

Em 50% dos casos, tem origem na camada subendocárdica; os outros 50%, mesocárdica ou epicárdica. Pode determinar alterações hemodinâmicas, a depender do tamanho. Apresentam sinal homogêneo semelhante aotecido gorduroso: hiperintenso em T1 e com queda do sinal nas sequências de saturação de gordura e iso/hipersinal em T2. ^167^ Não ocorre perfusão de primeira passagem nem realce tardio em sequências contrastadas ^168^ ( Figura 15 ).

##### 5.2.1.3. Fibroelastoma Papilar

**Figura 16:**
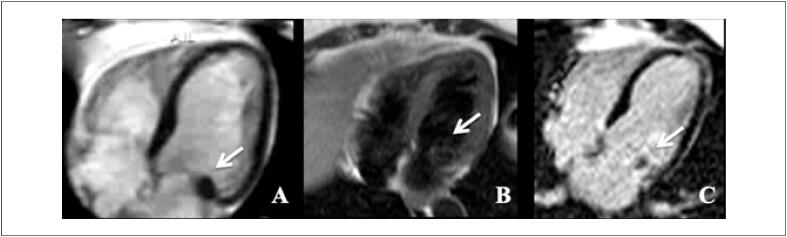
Fibroelastoma papilífero em valva mitral. A. Cinerressonância em quatro câmaras em que se observa a massa hiposinal. B. Imagem ponderada em T1 demonstrando isossinal. C. Sequência de realce tardio precoce com gadolínio demostrando realce leve local.

Nas imagens de cinerressonância, apresenta-se como uma massa hipointensa e extremamente móvel, sem necessariamente causar impacto funcional na valva. Avaliar as características teciduais da tumoração é difícil pelo pequeno tamanho e grande mobilidade, mas pode ser observado um sinal isointenso em T1 e hiperintenso em T2, com ausência deperfusão de primeira passageme hipersinal nas imagens de realce tardio ^167^ ( Figura 16 ).

##### 5.2.1.4. Rabdomioma

**Figura 17:**
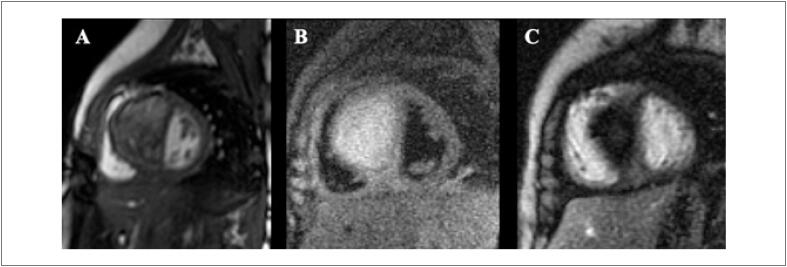
Rabdomioma de ventrículo esquerdo: A. Cinerressonância em eixo curto demonstrando a massa de localização intramural no septo interventricular. B. Imagem ponderada em T2 demonstrando hipersinal. C. Sequência de realce tardio demostrando leve realce local.

Discretamente hiperintenso em imagens ponderadas em T1 e hiperintenso em imagens ponderadas em T2. Apresenta mínimo realce ou até mesmo ausência após o gadolínio ( Figura 17 ).

##### 5.2.1.5. Fibromas

**Figura 18:**
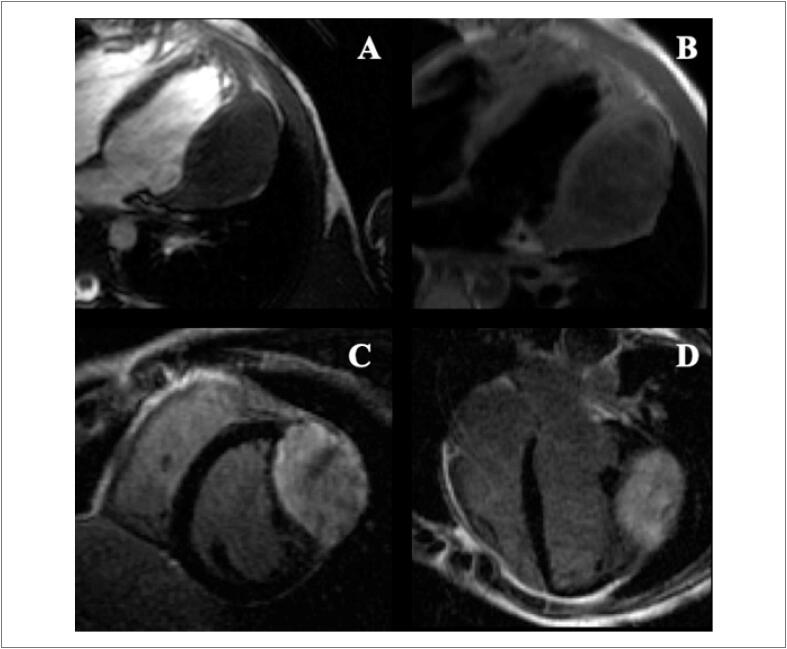
Fibroma intramural. A. Cinerressonância em quatro câmaras mostrando tumor intramural em parede lateral. B. Imagem ponderada em T2 mostrando a massa com hipossinal em relação ao miocárdio. C e D. Imagem de realce tardio em eixo curto e eixo longo mostrando hipersinal homogêneo do tumor compatível com tecido fibroso.

São geralmente iso/hipointensos nas imagens ponderadas em T1 e homogeneinamentehipointensos nas imagens ponderadas em T2. O aspecto na perfusão de primeira passagem gerelmente é ausente pela sua baixa vascularização. No realce tardio, os fibromas apresentam um realce homogêneo intenso, embora algumas vezes as lesões possam apresentar um componente central de baixo sinal que pode estar associado à calcificação local ^169^ ( Figura 18 ).

##### 5.2.1.6. Hemangioma

Apresentarão um aspecto heterogêneo iso ou hiperintenso nas imagens ponderadas em T1 e usualmente hiperintenso em pT2, podendo também ocorrer heterogeneidade com áreas de baixo sinal. O padrão do realce tardio é caracteristicamente heterogêneo e hiperintenso com uma marcada perfusão de primeira passagem. ^168^^,^^169^

#### 5.2.2. Tumores Malignos

Os tumores malignos costumam ser maiores, apresentam mais frequentemente perfusão de primeira passagem do gadolínio e maior prevalência de realce tardio. ^170^

##### 5.2.2.1. Sarcomas

**Figura 19:**
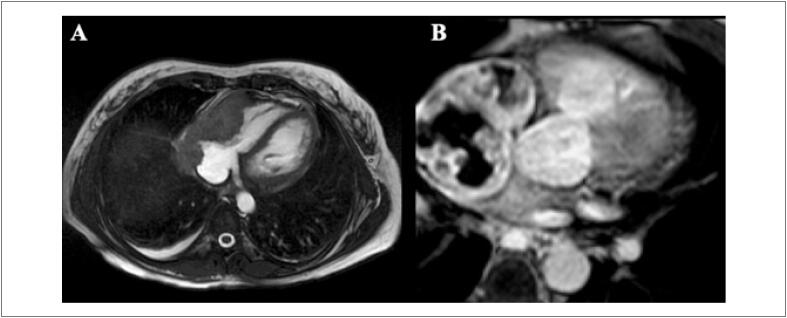
Angiossarcoma em átrio direito. A. Cinerressonância em quatro câmaras mostrando sarcoma com origem na parede posterior do átrio direito com infiltração das suas paredes, da porção basal do ventrículo direito e do pericárdio. B. Imagem de realce tardio com hipersinal heterogêneo da massa em corte axial mais superior.

**Figura 20:**
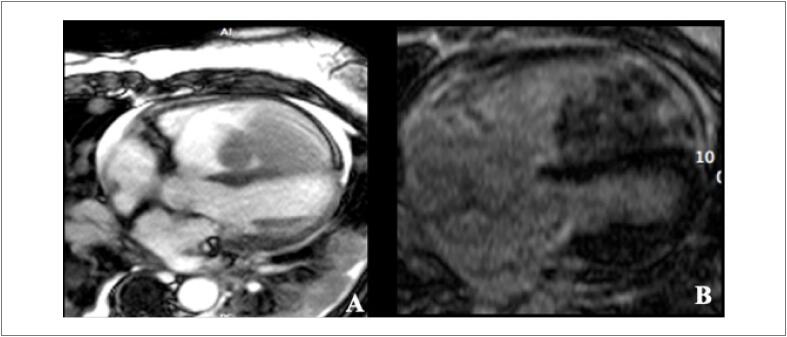
Rabdomiossarcoma em ventrículo direito. A. Cinerressonância em quatro câmaras mostrando massa multilobulada intracavitária em ventrículo direito, de grandes dimensões. B. Imagem de realce tardio com hipersinal heterogêneo da massa.

A maioria dos sarcomas mostra características sugestivas de malignidade como um sinal heterogêneo em virtude de necrose e hemorragia em seu interior, formato nodular ou irregular, bordas mal definidas, infiltração do miocárdio e das estruturas adjacentes, extensão para o pericárdio e derrame pericárdico associado.O angiossarcoma corresponde a 40% dos sarcomas cardíacos e localiza-se, em sua maioria, no átrio direito com infiltração da parede atrial e do pericárdio associado a derrame pericárdico ( Figura 19 ). Na RMC, mostra-se como uma grande massa lobular heterogênea com iso/hipersinal em T1, hipersinal em T2, perfusão de primeira passagem com gadolínio e hipersinal heterogêneo nas imagens de realce tardio ( Figura 20 ). ^147^^,^^169^

##### 5.2.2.2. Linfoma

Apresentam iso/hipossinal nas imagens ponderadas em T1, iso/hipersinal nas imagens ponderadas em T2 e realce tardio heterogêneo.

### 5.3. Contribuição da Medicina Nuclear

#### 5.3.1. ^18^ F-FDG PET-CT

A ^18^ F-FDG PET-CT ainda é pouco utilizada nos tumores cardíacos, principalmente nos tumores cardíacos malignos primários. Em relação às neoplasias cardíacas metastáticas (secundárias), que são as mais frequentes, a ^18^ F-FDG PET-CT é uma técnica já estabelecida. ^171^

Em um estudo tipo série de casos, Rahbar et al. ^172^ avaliaram 24 pacientes consecutivos com tumores cardíacos com ^18^ F-FDG PET-CT antes do tratamento. ^172^ Os pacientes foram divididos de acordo com o subtipo histológico dos tumores, obtidos por ressecção cirúrgica ou biópsia, resultando em: benignos (n = 07), malignos primários (n = 08) e malignos secundários (n = 09). A captação tumoral de ^18^ F-FDG foi observada entre os grupos. Posteriormente, para avaliar sensibilidade e especificidade do método na diferenciação entre benigno e maligno, foram separados apenas em malignos (n = 17) e benignos (n = 7). A captação de ^18^ F-FDG foi quantificada de acordo com o índice do valor máximo de captação padronizada (do inglês, *Maximum Standard Uptake Value* – SUV max) de volumes tridimensionais abrangendo a massa tumoral. No caso de baixa captação tumoral de ^18^ F-FDG, as imagens da TC foram utilizadas para identificar e obter os volumes do tumor. Além disso, em virtude de captação miocárdica fisiológica de ^18^ F-FDG, captação no miocárdio normal e *pool* sanguíneo foram medidos e comparados com os SUVs max dos tumores. Observou-se que a captação de ^18^ F-FDG foi baixa no *pool* sanguíneo e no miocárdio normal, e significativamente alta nos tumores primários malignos em relação aos benignos. Os tumores malignos secundários apresentaram uma captação comparável a dos malignos primários; no entanto, com uma variação de SUV max consideravelmente maior. A média do SUV max foi de 2,8 ± 0,6 no grupo de tumores cardíacos benignos. Esses tumores geralmente não apresentam contraste positivo em relação ao miocárdio normal e são visualizados apenas nas imagens morfológicas da TC. Em contrapartida, os tumores malignos primários apresentam SUV max 2,5 vezes maior em relação aos benignos. Entre os tumores cardíacos metastáticos, a SUV max foi de 10,8 ± 4,9 com uma variação significativa na captação de ^18^ F-FDG de 3,4 a 16,7. Em relação aos tumores malignos primários, a captação de ^18^ F-FDG foi maior do que os benignos e semelhante aos malignos secundários. ^172^

##### 5.3.1.1. Valor de Ponto de Corte do SUV max de ^18^ F-FDG para Melhor Diferenciar Tumor Cardíaco Benigno de Maligno

Diferentes pontos de corte do SUV max têm sido sugeridos na determinação de malignidade por meio da ^18^ F-FDG PET-CT. No estudo de Rahbar et al., ^172^ um valor de 3,5 alcançou uma sensibilidade de 100%, especificidade de 86%, valor preditivo positivo de 94% e negativo de 100%. ^172^ Quando aumentaram esse valor para 4,6 encontraram uma especificidade de 100%, sensibilidade de 94% e um valor preditivo positivo de 100% para malignidade. Dentre as principais limitações deste estudo, estão a natureza retrospectiva, a falta de um padrão diagnóstico para tumores cardíacos e a heterogeneidade dos resultados. Além disso, diversos mixomas não foram incluídos na análise por terem sido diagnosticados ao ecocardiograma e à RMC.

## 6. Situações Especiais

### 6.1. Doença Cardíaca Carcinoide

Os tumores neuroendócrinos são neoplasias raras (2,5 a 5 casos por 100.000 habitantes), podendo surgir em qualquer lugar, sendo mais frequentes no trato gastrointestinal (carcinoides). ^173^ Cerca de 30% a 40% dos casos, em sua maioria localizado no intestino delgado e cólon proximal, manifestam alterações vasomotoras (hipotensão, hipertensão, *flushing* ), diarreia e broncospasmo, chamada síndrome carcinoide, geralmente associada à metástase hepática. ^174^ A presença de biomarcadores como NT-proBNP, cromagraninaA e 5 – hidroxindolacético (5-HIAA) é útil tanto no diagnóstico quanto no prognóstico da doença carcinoide. ^175^

A doença cardíaca carcinoide (DCC), manifestada pela formação de placas, espessamento e fibrose endocárdica, acomete preferencialmente as cavidades direitas, valvas tricúspide e pulmonar, sendo provavelmente relacionada com a exposição crônica ao 5-HIAA. O comprometimento das cavidades esquerdas (15% dos casos), valvas mitral e aórtica é visto na presença de *shunt* direita/esquerda (p. ex., forame oval patente) ou na presença de tumor carcinoide brônquico. ^176^ O diagnóstico precoce e o monitoramento da progressão da DCC impactam drasticamente no prognóstico e na sobrevida alongo prazo (sobrevida média de 1,6 ano *vs.* 4,6 anos na ausência de DCC), uma vez que o tratamento cirúrgico precoce é fundamental para o sucesso.

**Figura 21:**
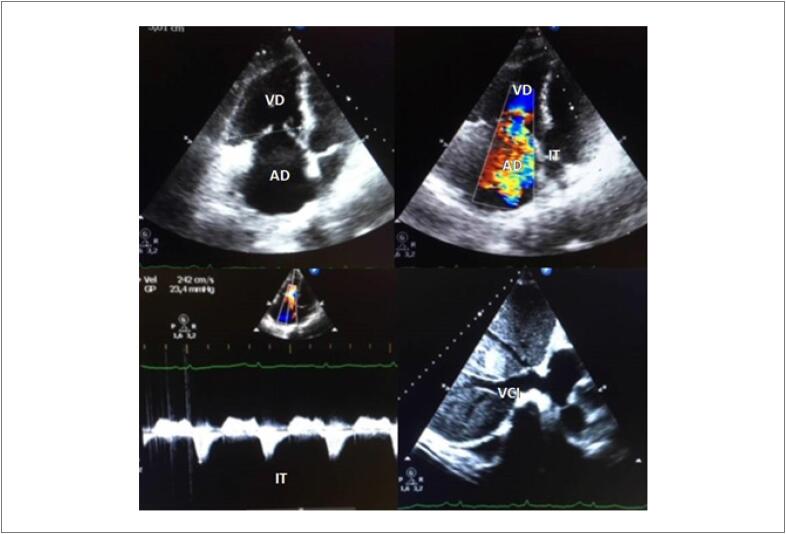
Paciente com tumor carcinoide, metástase hepática, quadro clínico de insuficiência cardíaca direita. VD: ventrículo direito, AD: átrio direito, IT: insuficiência tricúspide, VCI: veia cava inferior.

A ecocardiografia é o padrão-ouro para o diagnóstico e acompanhamento da DCC. ^173^ A avaliação de cavidades cardíacas direitas (átrio e ventrículo), função ventricular direita, espessamento, mobilidade dos folhetos e presença de regurgitação ou estenose valvar (analisadas individualmente) deve ser realizada na suspeita clínica inicial, no surgimento de novo sopro cardíaco, novos sintomas ou a cada 3 a 6 meses conforme a severidade da DCC ( Figura 21 ).

O comprometimento da valva tricúspide levando à regurgitação é a alteração mais frequente, seguida da regurgitação pulmonar, estenose tricúspide e, por último, estenose pulmonar. Na valva tricúspide ocorre principalmente o envolvimento dos folhetos septal e anterior, ficando o folheto posterior relativamente preservado. Dilatação e disfunção do VD são consequências da gravidade das lesões valvares. A identificação de metástase cardíaca (4% dos casos) também é possível pela ecocardiografia, entretanto é mais bem visualizada pela RNM. ^173^^,^^176^

Diversos escores ecocardiográficos foram propostos para avaliação da DCC.Dentre os escores mais simples, o descrito por Westberg et al. ^177^ ( Tabela 5 ) avalia apenas a anatomia e regurgitação da valva tricúspide. A soma dos pontos > 1 é considerada como patológica (acurácica 87%). A sobrevida em 3 anos é inferior a 45% quando escore > 4 contra 75% no escore 0. ^177^

**Tabela 5:** Escore de Westberg ^171^

Característica	Severidade e pontuação				
Espessamento valvar	Normal 0	Leve 1	Moderada 2	Severa 3	Severa com retração valvar 4
Regurgitação	Normal 0	Leve 1	Moderado 2	Importante 3	Extrema 4

Escores mais complexos como o de Bhattacharyya et al. ^173^ ( Tabela 6 ) oferecem um número maior de informações, sendo úteis no acompanhamento e planejamento cirúrgico. Nele, um escore > 8 apresenta uma acurácia de 96% para o diagnóstico de DCC, sendo que o aumento de 5 pontos no escore foi preditor independente da progressão da DCC (RR 2,95) e de mortalidade (RR 2,66) segundo Dobson et al. ^176^^,^^178^

**Tabela 6:** Escore de Bhattacharyya ^167^

Característica	Severidade e pontuação			
Espessamento valvar	< 3 mm 0	≥ 3 e < 4 mm 1	≥ 4 e < 5 mm 2	≥ 5mm 3
Mobilidade valvar	Normal 0	Leve 1	Moderada 2	Severa/fixa 3
Morfologia valvar	Normal 0	Retificada 1	Retração leve 2	Retração moderada/severa 3
Estenose valvar	Normal 0	Leve 1	Moderado 2	Importante 3
Regurgitação valvar	Normal 0	Leve 1	Moderado 2	Importante 3
Diâmetro do ventrículo direito	Normal 0	Aumento leve 1	Aumento moderado 2	Aumento importante 3
Função do ventrículo direito	Normal 0	Redução leve 1	Redução moderada 2	Redução importante 3

Tratamento cirúrgico cardíaco está indicado por ocasião da dilatação e disfunção do VD e sinais de insuficiência cardíaca refratária ao tratamento medicamentoso. O fechamento de FOP com o objetivo de reduzir o *shunt* direita/esquerda e a remoção das metástases devem ser recomendados durante a abordagem. ^175^

### 6.2. Amiloidose Cardíaca (AC)

#### 6.2.1. Introdução

A amiloidose é uma doença multissistêmica, decorrente do depósito no espaço extracelular de material proteináceo composto, de difícil diagnóstico, e, uma vez comprovado o acometimento cardíaco, prognóstico reservado. De acordo com o tipo de proteína precursora, pode ser chamada de amiloidose de cadeia leve (AL), quando os depósitos se originam de proteínas de cadeia leve, e amiloidose relacionada à transtirretina (ATTR), cuja proteína se origina da proteína transportadora de hormônio tiroxina e retinol. ^179^

#### 6.2.2. Tipos Clínicos e Envolvimento Cardíaco

A forma AL pode envolver vários órgãos como os rins, trato gastrointestinal e sistema nervoso autônomo, além do próprio coração. É causada pelo depósito de proteínas derivadas das imunoglobulinas de cadeia leve produzidas pelas células plasmáticas em quadros de discrasia plasmocitária como no mieloma múltiplo (MM). Cerca de 10% dos pacientes com MM podem desenvolver AL e, destes, mais da metade pode apresentar AC. O acometimento cardíaco está em segundo lugar depois do envolvimento dos rins na AL. ^180^^,^^181^

O mecanismo patológico do tipo ATTR é o depósito de derivados da transtirretina, produzida pelo fígado.É subdividida em dois outros tipos: a) ATTR do tipo selvagem, com a sigla ATTRwt (sigla em inglês, “ *wild type* ”), com acometimento predominante do coração, além de frequentemente causar síndrome do túnel do carpo, ruptura do tendão do bíceps e estenose espinhal; b) ATTR mutante (ATTRm), associada a substituições específicas dos genes que codificam a transtirretina e podem acometer o sistema nervoso autônomo e periférico além do próprio coração. ^182^

#### 6.2.3. Contribuição da Ecocardiografia

O ETT é a opção diagnóstica mais usada quando se suspeita do diagnóstico de AC e é usado também para rastrear casos em familiares. É altamente disponível em serviços médicos de complexidade variável, custo relativamente baixo e com a possibilidade de ser repetido de forma seriada com boa reprodutibilidade e consistência dos seus achados. A presença de acometimento cardíaco guarda em si o pior prognóstico deste grupo de pacientes.

##### 6.2.3.1. Aumento da Espessura Miocárdica

**Figura 22:**
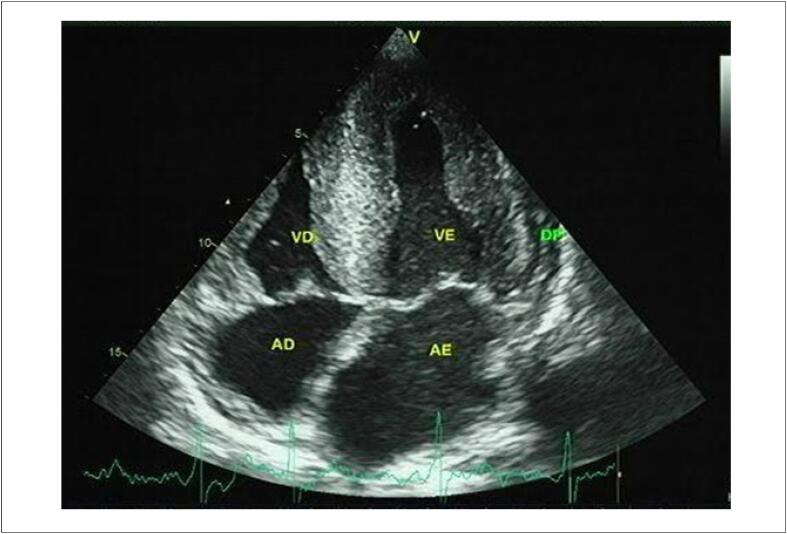
Paciente portador de hipertrofia do ventrículo esquerdo (VE) sem causa determinada. Ecocardiograma evidencia aspecto brilhante ou cintilante granular do miocárdio, sugestivo de amiloidose cardíaca.

O miocárdio apresenta aspecto brilhante ou cintilante granular, melhor apreciado na imagem fundamental, sem o uso da harmônica. O aumento da espessura miocárdica é frequentemente concêntrico e simétrico, diferentemente dos casos de cardiomiopatia hipertrófica que tendem a exibir maior assimetria na distribuição da mesma ( Figura 22 ). A presença de aumento da espessura miocárdica e de ondas de baixa voltagem ao eletrocardiograma consiste em achados que levam à suspeita para AC. ^183^ O termo “aumento da espessura miocárdica” deve ser diferenciado da hipertrofia miocárdica secundária, em que há hipertrofia miocelular, uma vez que, na AC, é o espaço extracelular que se encontra aumentado pela deposição do material amiloide proteico. ^184^ O aumento da espessura miocárdica é difuso e pode incluir todas as paredes do VE, parede livre do VD, septo interatrial e valvas com ou sem regurgitação associada. A espessura miocárdica de 12mm ou superior pode ser usada como valor de corte para suspeita diagnóstica de AC. ^185^ O valor clínico e o prognóstico do aumento da espessura miocárdica já são conhecidos. Quanto mais exuberante, maior será a ocorrência de insuficiência cardíaca congestiva (ICC) e pior a sobrevida.

##### 6.2.3.2. Átrio Esquerdo

Alterações no átrio esquerdo também podem ser encontradas e o aumento do volume atrial esquerdo pode ser devido não somente a graus elevados de disfunção diastólica, mas também à própria deposição de fibrilas amiloides.

Alterações na deformação miocárdica atrial (s *train* e *strain rate)* , assim como em parâmetros de função sistólica atrial, são frequentemente observadas em pacientes com poucas características ecocardiográficas devido à AC, e também em indivíduos com sintomas de ICC, estando associadas à piora no prognóstico. ^186^

##### 6.2.3.3. Função Diastólica

Em fases mais avançadas a avaliação da função diastólica costuma apresentar relação E/e’ aumentada, compatível com aumento das pressões de enchimento do VE. Entretanto, parâmetros da função diastólica encontram-se alteradas mesmo nas fases iniciais da AC. A presença da disfunção diastólica está associada à piora na evolução clínica, como já demonstrado, além sugerir um maior risco de tromboembolismo. ^187^^-^^189^

##### 6.3.3.4. Função Sistólica do Ventrículo Esquerdo

A FEVE pode ainda estar dentro dos limites da normalidade e esta característica permite incluir a AC dentro das causas de insuficiência cardíaca com fração de ejeção preservada (ICFEP). A piora da função sistólica ocorre em fases mais tardias e está associada à piora clínica e prognóstica. ^187^^,^^189^

Considerando as medidas de deformação miocárdica, a AC exibe redução no *SLG* miocárdico principalmente nas regiões basais e médias do VE com preservação dos valores na região apical (preservação apical). ^188^^,^^190^ É útil calcular a relação da média dos valores do SLG apical pela média da soma dos valores do SLG nas regiões basal e média do VE. Esta relação> 1 apresenta alta especificidade para o diagnóstico de AC. ^190^ Quando se considera a relação entre as medidas da FEVE e valores de *strain* miocárdico (da sigla em inglês *“EFS”* ou *ejection fraction and strain relation* ), há uma inversão desta relação com menores valores de deformação miocárdica e FEVE preservada, independentemente dos sintomas claros de ICC. EFS igual ou superior a quatro é util na diferenciação entre AC e CMH, com valores de sensibilidade iguaisa 89,7% e especificidade de 91,7%. ^187^^,^^188^

Valores isolados de *SLG* miocárdico maiores que-15% estão associados a maior gravidade nos casos de AC, bem como aumento na mortalidade. ^191^ Em outro estudo, o valor de *strain superior* a -17% conseguiu separar grupos de pacientes com resposta clínica desfavorável após transplante de medula óssea autólogo. O índice de preservação apical ( *apical sparring* ) com resultado igual ou superior a 1,19 se associou a maior mortalidade e necessidade de transplante cardíaco em 5 anos de seguimento. Este mesmo índice mostrou valor preditivo nas curvas de sobrevida e necessidade de transplante cardíaco quando em associação a menor valor de FEVE. ^192^

Liu et al. ^193^ analisaram o valor do pico da onda diastólica da curva de *strain rate* isoladamente (fase rápida de enchimento diastólico) em 41 pacientes com AC, demonstrando que o valor global do *strain rate* diastólico (LSR_dias_ global > -0,85 S^−1^) foi preditivo de aumento em 4x na mortalidade em pacientes com AC com FEVE preservada (FEVE > 50%). ^193^

**Figura 23:**
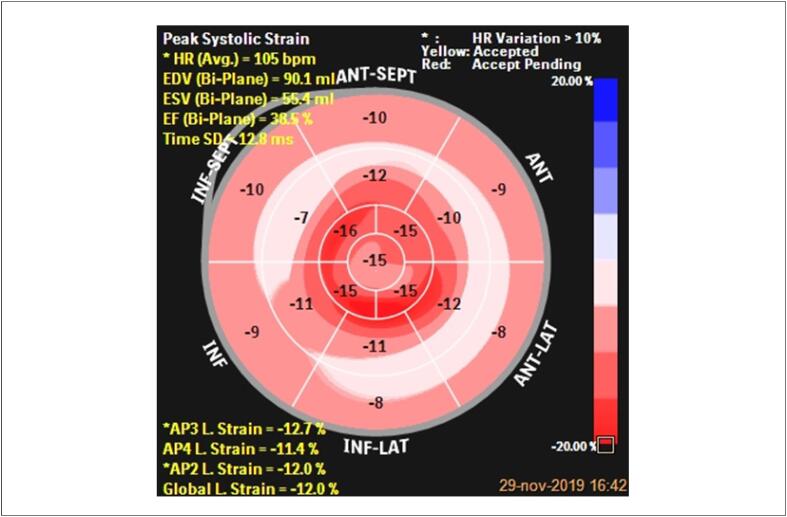
Strain global longitudinal do ventrículo esquerdo em um paciente com amiloidose por transtirretina, demonstrando o seu padrão peculiar que poupa o ápice e acomete mais as porções mediobasais.

As medidas de *strain* são altamente sensíveis e exibem boa especificidade nos casos de AC, porém há que se destacar que medidas ecocardiográficas convencionais da função sistólica ainda são importantes e demonstram ótima correlação prognóstica nesses casos. Assim, o volume sistólico indexado (VSi) e a medida que integra volume sistólico e o volume total da massa miocárdica (denominada fração de contração miocárdica, do inglês *myocardial contraction fraction* ou *MCF* ) mostraram bom valor prognóstico em pacientes com AC independentemente do tipo e comparáveis às medidas do *strain* . Assim, neste grupo de pacientes, o VSi<33mL/min e a MCF <34% com índice cardíaco <2,4 L/min/m^2^ foram os melhores preditores de sobrevida global com acurácia comparável às medidas de *strain*^194^ ( Figura 23 ).

##### 6.2.3.5. Outros Achados

Apesar de não apresentar incidência notável em casos de AC, a presença de trombos intracardíacos já foi relatada e deve ser destacado que não tem necessariamente associação com a presença de fibrilação atrial. ^195^ Alguns autores já demonstraram que o tipo AL e a presença de disfunção diastólica são fatores de risco independentes para presença de trombos intracardíacos. ^196^ A pesquisa por trombos deve ser sempre considerada em pacientes que apresentam critérios de gravidade da AC (altos graus de aumento da espessura miocárdica, diminuição da FEVE e estágios avançados de disfunção diastólica, por exemplo). Vários mecanismos já foram propostos para explicar a ocorrência de trombos, além da própria presença da fibrilação atrial. Assim, podem ser citados: depósitos de substâncias amiloides em região subendocárdica que produzem lesão e maior enrijecimento parietal; estase hemodinâmica como consequência da disfunção diastólica importante geralmente do tipo restritiva; e hipercoagulabilidade plasmática, como mecanismo sistêmico decorrente de várias condições.

Tamponamento cardíaco devido a derrame pericárdico importante é raro e o seu diagnóstico é predominantemente clínico, visto que as pressões elevadas nas câmaras direitas diminuem as chances de colabamento clássico ao ecocardiograma. O derrame pericárdico de menor intensidade é mais comum. As valvas cardíacas também podem estar infiltradas, porém raramente exibem regurgitação valvar severa. ^187^

##### 6.2.3.6. Abordagem Diagnóstica

O diagnóstico da AC se inicia com o exame de ecocardiograma em inúmeras situações clínicas, mas muito frequentemente se dá em uma das duas circunstâncias de avaliação pelo cardiologista.

A primeira diz respeito à necessidade da avaliação cardiológica em pacientes com mieloma múltiplo. Nesses casos, é imperativa a confirmação do acometimento cardíaco, visto que a sobrevida nos casos de AC do tipo AL pode ser extremamente desfavorável. Mesmo que os achados ecocardiográficos clássicos estejam ausentes no primeiro exame, recomenda-se repetir no seguimento do paciente.

Na segunda situação, pacientes são submetidos à avaliação cardiológica seja por apresentarem quadro clínico de ICFEP ou, ainda, pela presença de “hipertrofia” no exame de ecocardiografia (frequentemente associada a complexos com baixa voltagem no ECG).

Nestes casos, além da pesquisa dos achados ecocardiográficos descritos anteriormente, sugere-se prosseguir investigação com outros exames de imagem (RNM e MN) e investigação laboratorial como eletroforese de proteínas e teste de proteínas de cadeia leve.

Nos centros em que o estudo da deformidade miocárdica está disponível, a presença de preservação apical é típica, porém não é exclusiva. Outras causas de hipertrofia ventricular esquerda podem exibir o mesmo padrão, como hipertrofia secundária à HAS e à estenose aórtica.

#### 6.2.4. Contribuição da Ressonância Magnética Cardíaca

A RMC permite o diagnóstico de AC pelas alterações morfológicas e funcionais nas sequências de cinerressonância, assim como o ETT, e de forma mais acurada através das sequências de realce tardio e de mapa T1.

A sequência de realce tardio geralmente detecta o depósito de proteína amiloide no espaço intersticial do miocárdio através do hipersinal (cor branca) presente no músculo normalmente preto nessas imagens. ^197^ Syed et al. ^198^ analisaram a RMC em 120 pacientes com amiloidose. ^198^ Destes, 35 tinham confirmação de envolvimento cardíaco pela histologia. Os outros 85 foram divididos naqueles com ou sem evidência ecocardiográfica de AC. Nos 35 pacientes com diagnóstico histológico, 97% tinhamrealce tardio e 91% apresentavamaumento da espessura parietal pela ecocardiografia. Realce tardio transmural ou subendocárdico foi o padrão mais comumente encontrado (83%), sendo associado a maior depósito intersticial de amiloide; realce tardio focal (6%) e dificuldade de anular o sinal do miocárdico (8%) também foram padrões associados ao envolvimento cardíaco. Nos 85 pacientes sem histologia, o realce tardio esteve presente em 86% dos pacientes com alteração ecocardiográfica e em 47% dos pacientes sem evidência de AC pela ETT. A presença e o padrão de realce tardio foram associados à classe funcional pela NYHA, à baixa voltagem no ECG, ao índice de massa do VE, ao espessamento parietal do VD e aos valores de troponina e peptídio natriurético do tipo B (BNP) séricos. ^198^

Austin et al. ^199^ estudaram 47 pacientes com suspeita de AC que foram submetidos a RMC, ECG, ETT e biópsia. ^199^ Em comparação à biópsia, o realce tardio teve sensibilidade de 88%, especificidade de 90%, valor preditivo positivo de 88% e valor preditivo negativo de 90%, sendo o único com acurácia diagnóstica significativa na análise multivariada. Após 1 ano da biópsia, 19% dos pacientes faleceram, sendo o realce tardio o único fator preditor significativo de mortalidade no período, mostrando o papel prognóstico deste parâmetro na amiloidose.

Comrelação à diferenciação dos tipos de AC, a RMC pode ajudar na predição da AL e da ATTR. Dungu et al. ^200^ mostraram maior aumento da espessura miocárdica (228g x 167g) e maior extensão do realce tardio nos pacientes com ATTR, apesar da pior mortalidade dos pacientes com AL. Neste estudo, 90% dos pacientes com ATTR apresentaram algum segmento com realce tardio transmural em comparação com 37% dos pacientes com AL. Além disso, 100% tiveram realce tardio no VD na ATTR em comparação com 72% na AL. Os autores criaram um escore, o qual denominaram QALE (Query Amyloid Late Enhancement), com valores variando de 0 a 18. Valores superiores ou igual a 13 são capazes de predizer ATTR em vez de AL, com sensibilidade de 82% e especificidade de 76%.

A sequência de mapa T1, técnica mais recente na RNM, também pode ajudar no diagnóstico e prognóstico de pacientes com AC, além de servir no futuro para monitorar o tratamento de novos agentes terapêuticos nesta doença. Os valores de T1 pré-contraste estão aumentados em pacientes com AC, sendo esta uma opção diagnóstica em pacientes ainda sem realce tardio, ou naqueles que não realizam a sequência de realce tardio por contraindicação à injeção de gadolínio. Além disso, com a injeção de gadolínio, é possível calcular o volume extracelular do miocárdio (ECV), que está expandido na AC pelo depósito de fibrilas amiloides no interstício, apresentando boa correlação com a biópsia, tendo valor prognóstico comprovado. ^201^^,^^202^

#### 6.2.5. Contribuição da Medicina Nuclear

**Figura 24:**
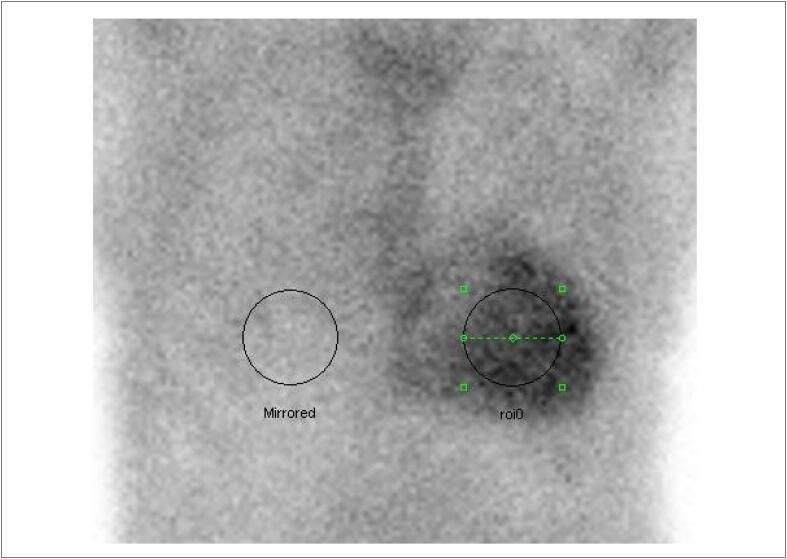
Imagem na projeção anterior do tórax realizada uma hora após a administração intravenosa do pirofosfatomarcado com Tecnécio-99m (^99m^Tc). Cada um dos círculos representa uma região de interesse (ROI) e permite quantificar a concentração do traçador. A relação das contagens entre hemitórax esquerdo e direito (HTE/HTD) deste caso foi de 1,75, sendo, portanto, positiva para amiloidose cardíaca forma transtirretina (ATTR).

**Figura 25:**
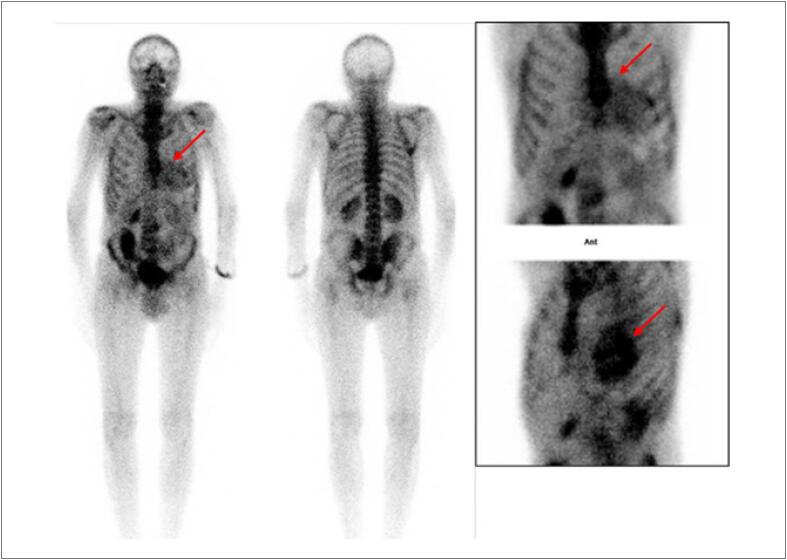
Cintilografia de corpo inteiro e imagens planares do tórax com pirofosfato^99m^Tc demonstrando acúmulo anormal do radiofármaco na projeção do coração (setas) em paciente com insuficiência cardíaca avançada com fração de ejeção preservada. As dosagens séricas de cadeias leves foram negativas e uma biópsia de gordura abdominal foi positiva para amiloidose. O diagnóstico definitivo foi de amiloidose cardíaca por transtirretina.

**Figura 26:**
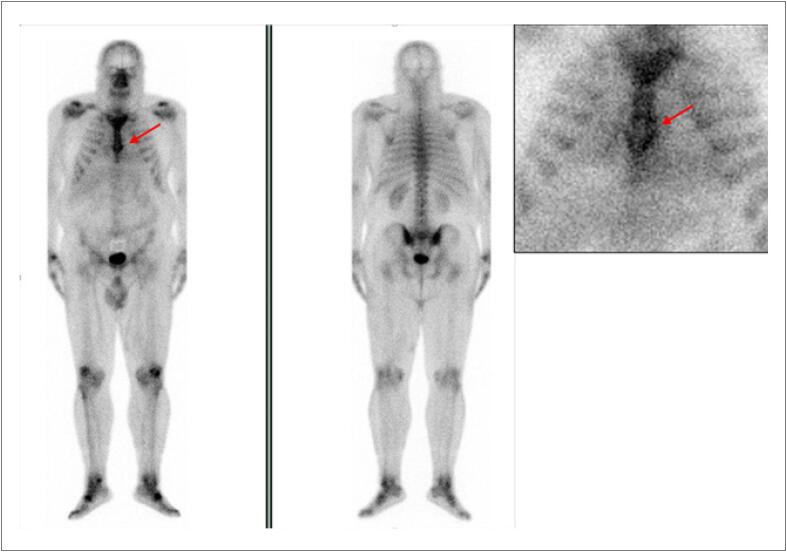
Cintilografia de corpo inteiro e imagens planares do tórax com pirofosfato^99m^Tc demonstrando acúmulo de intensidade leve (grau 1) na projeção do coração ( *seta* ) em paciente com posterior confirmação de amiloidose de cadeia leve (AL) por achado de pico monoclonal na eletroforese de proteínas séricas e urinárias, aumento da dosagem sérica da forma livre dos anticorpos ( *freelight* ) e aspirado de medula óssea demonstrando infiltração plasmocitária com celularidade inferior a 10%. A confirmação final foi realizada por biópsia de gordura abdominal, que foi positiva para amiloidose.

**Figura 27:**
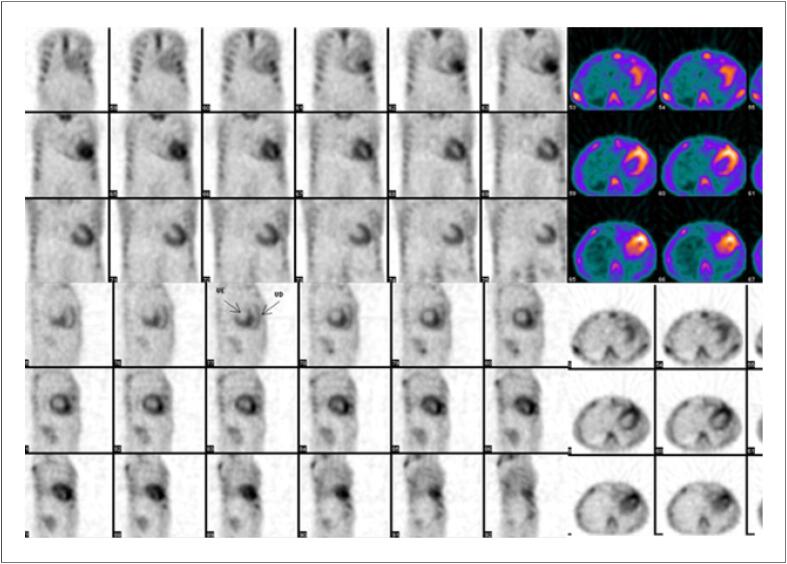
Imagens tomográficas (SPECT) reconstruídas nos eixos axial, coronal e sagital, identificando concentração do pirofosfato^99m^Tc no ventrículo esquerdo e acometimento simultâneo do ventrículo direito (setas) em paciente com suspeita de amiloidose cardíaca forma transtirretina (TTR) mutada, confirmada por teste genético.

**Figura 28:**
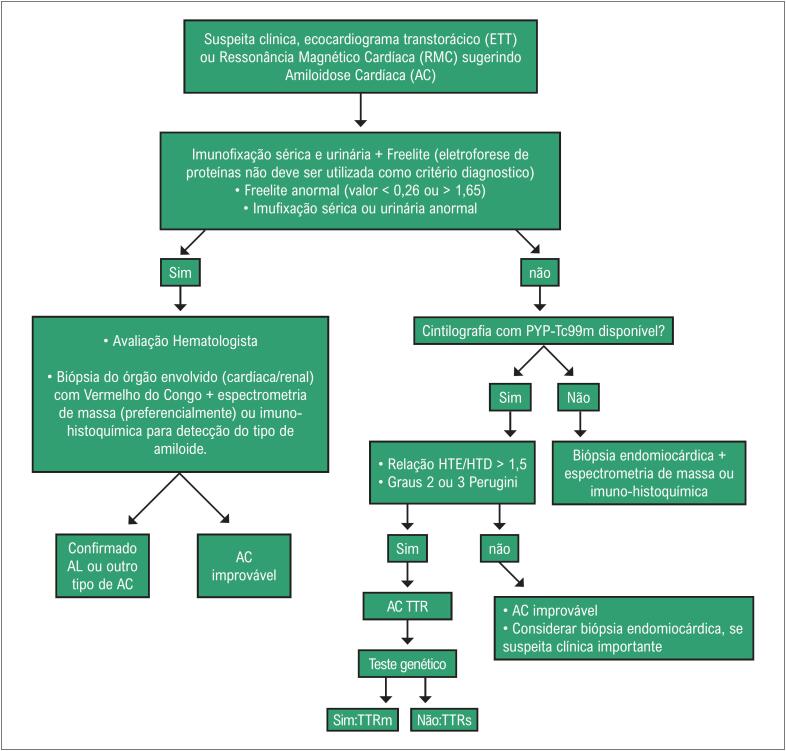
Sequência diagnóstica proposta através da medicina nuclear para casos suspeitos de amiloidose cardíaca (AC). TTR: transtirretina (TTRm: TTR mutada; TTRs: TTR selvagem); AL: amiloidose cardíaca de cadeia leve; PYP: pirofosfato; DPD: ácido 3,3-difosfono-1,2-propanodicarboxílico; ^99m^Tc: tecnécio-99m.

A medicina nuclear (MN) ganhou uma nova relevância na detecção da AC com os estudos que demonstraram que os radiotraçadores com afinidade óssea, como o ácido 3,3-difosfono-1,2-propanodicarboxílico (DPD) e o pirofosfato (ambos marcados com Tecnécio-99 m), apresentam sensibilidade bastante importante para detecção da ATTR. A cintilografia com esses traçadores permite a diferenciação dos subtipos de proteína amiloide, de forma não invasiva, baseada nos graus de concentração do traçador na área cardíaca. Essa diferenciação tem implicações prognósticas e terapêuticas. ^203^ O mais importante estudo sobre o tema até o momento é o de Gilmore et al. ^204^ Eles avaliaram os resultados da cintilografia com radiotraçadores de afinidade óssea em 1.217 pacientes com suspeita de AC encaminhados para avaliação em centros especializados, obtendo valores de sensibilidade de até 99% quando já descartada a forma AL pela análise bioquímica. ^204^ A técnica do exame consiste na injeção do traçador em veia periférica com aquisição de imagens planares do tórax na projeção anterior após uma hora da administração do radiofármaco ( Figura 24 ). Essa imagem permite a realização da análise quantitativa na qual duas regiões de interesse (ROI – region of interest) são desenhadas, uma sobre a área cardíaca no hemitórax esquerdo (HTE) e outra no hemitórax direito (HTD), em imagem espelhada. A relação entre as contagens (representando a quantidade do traçador presente em cada área) do HTE e HTD maior que 1,5 tem elevada sensibilidade diagnóstica (95% de sensibilidade e 79% de especifidade). ^203^ Esta relação do HTE/HTD possui poder diagnóstico, mas também prognóstico, considerando que valores maiores que 1,6 conferem pior prognóstico em seguimento de 5 anos. Após 3 horas são adquiridas imagens do tórax (sendo opcional as imagens de corpo inteiro) nas projeções anterior e posterior, oblíqua e lateral esquerdas. O grau de concentração do traçador na área cardíaca é avaliado em comparação ao gradeado costal, variando de grau 0 a 3: grau 0, ausência de concentração cardíaca; grau 1, concentração cardíaca discreta e menor que ao gradeado costal; grau 2, igual ao gradeado costal; e grau 3, maior que o gradeado costal. Os graus 2 e 3 estão fortemente associados à forma ATTR (> 99% de sensibilidade e 86% de especificidade) no contexto de cadeias leves já excluída ( Figura 25 ). Os graus 0 e 1 podem ainda estar relacionados à forma AL ( Figura 26 ) ou TTRm em fase inicial da doença. A imagem de 3 horas possui maior especificidade diagnóstica para ATTR (sensibilidade de 58% e especifidade de 100%). ^205^^,^^206^ As imagens tomográficas, imagens SPECT (do inglês, *Single Photon Emission Tomography* ), da região do tórax no tempo de 3 horas têm sido utilizadas com mais frequência por melhorar a capacidade de distinção da presença de atividade na cavidade ventricular esquerda ( *blood pool)* e melhorar a avaliação do septo interventricular, sítio passível de biópsia miocárdica nos casos em que essa ainda se faz necessária. Essa imagem adicional também possibilita a comparação quantitativa da concentração miocárdica com o gradeado costal de forma mais precisa. A Figura 27 evidencia acometimento do VD por ATTRm, melhor avaliado às imagens tomográficas. O metileno difosfonado marcado com Tecnécio-99m (MDP-99mTc) é o traçador ósseo de maior disponibilidade no território nacional, mas a sua utilização para fins de avaliação de AC é desencorajada. Estudo comparou os fármacos MPD e DPD marcados com Tecnécio-99m em um grupo de pacientes com amiloidose familiar e a performance diagnóstica deste traçador é considerada subótima, com eventuais falsos negativos. ^207^ A Figura 28 representa uma sequência diagnóstica para casos suspeitos de AC através da MN.

### 6.3. Síndrome de Takotsubo

A síndrome de Takotsubo (ST) é caracterizada por disfunção ventricular esquerda aguda e reversível com comportamento semelhante à síndrome coronariana aguda, geralmente com início abrupto de dor precordial e dispneia. É uma síndrome cardíaca induzida por estresse sem a evidência de DAC obstrutiva, que geralmente tem recuperação espontânea dentro de dias ou semanas.

Nos últimos anos, tem havido um grande interesse em estudar a relação entre câncer e ST. Isso aconteceu principalmente após alguns estudos publicados mostrarem uma forte associação entre essas duas condições. ^208^

A prevalência de neoplasias parece ser maior em pacientes com ST quando comparada com indivíduos na mesma faixa etária e sexo, tanto no momento do diagnóstico desta doença como no seguimento desses pacientes. Enquanto, na população geral, o diagnóstico de ST gira em torno de 1% a 2%, em indivíduos com malignidade essa condição chega a 10%. ^209^

Na maioria dos casos, a ST envolve segmentos apicais e médios do VE, que se mostram acinéticos ou discinéticos (padrão de “balonismo apical”), em oposição aos segmentos basais, que em geral encontram-se hipercinéticos. Algumas formas com padrão variante foram descritas como a forma “medio ventricular” e “invertida”. A “medio ventricular” se caracteriza por acinesia dos segmentos medio ventricular, com hipocinesia ou contração normal dos segmentos apicais e hipercontratilidade da base. O padrão “invertido” caracteriza-se por duas formas diferentes, a primeira definida pela característica de poupar a região apical,apresentando-se com hipocinesia das paredes remanescentes, e a segunda com hipocinesia limitada aos segmentos basais.

A ecocardiografia com *speckle tracking* permite a avaliação da deformação multidirecional do VE. Nos casos típicos (“balonismo apical”), há um comprometimento da deformação longitudinal apical e média,com um gradiente de deformação base-ápice.Ainda nessa fase,todos os componentes da mecânica de torção do VE envolvendo as fases sistólica e diastólica estão comprometidos. Dada a sua maior sensibilidade na detecção de anormalidades sutis em comparação aos parâmetros mais tradicionais, como FEVE e índice de motilidade de parede, a ecocardiografia com *speckle tracking* pode ajudar no diagnóstico de ST. ^210^

A ecocardiografia 3D trouxe a possibilidade de demonstração mais acurada de volumes do VE e FEVE em comparação com a ecocardiografia 2De angiografia. No entanto, estudos adicionais usando essa tecncologia no curso da ST ainda são nescessários para estabelecer o seu papel na prática clínica.

Os tumores mais prevalentes entre pacientes com ST foram: colorretal, mamário, brônquico e melanoma. ^208^ As neoplasias hematológicas foram menos prevalentes.Em relação aos potenciais fatores desencadeantes, identificou-se uma relação com o tratamento oncológico em 57% dos casos: estresse cirúrgico (33%), QT (17%) e RT (7%); os demais foram estresse emocional (30%) e presença de outra doença aguda (13%). ^209^

Os pacientes com malignidade têm um limiar de tolerância reduzido para estressores e um aumento da sensibilidade dos receptores adrenérgicos cardíacos. Nesse cenário, a adição de estressores físicos (dor secundária ao câncer, procedimentos diagnósticos, cirurgias oncológicas) e estressores emocionais (medo de doença ou morte, mudanças na dinâmica familiar), comuns à doença, contribuem para uma maior predisposição paraapresentar a ST. Algumas malignidades, como feocromocitoma e paraganglioma, causam hipercatecolaminemia e podem ser um gatilho da ST. ^211^

### 6.4. Miocardiopatia Siderótica (Sobrecarga de Ferro)

A prevalência de anemia nos pacientes com câncer é elevada, estando presente em mais da metade dos pacientes durante o curso da doença, especialmente em pacientes com neoplasia de origem hematológica. A anemia considerada moderada a grave ocorre em aproximadamente 40% dos pacientes, sendo de causa multifatorial, podendo ser secundária à mielossupressão relacionada com tratamento, sangramento oculto, deficiência funcional de ferro, deficiência de eritropoetina devido à doença renal e/ou envolvimento medular com tumor. ^212^ A principal doença oncológica que leva à sobrecarga de ferro é a síndrome mielodisplásica (SMD), tanto pelo aumento da absorção quanto pelas transfusões regulares. Como consequência, esses pacientes com câncer ou sobreviventes podem sofrer sobrecarga de ferro em órgãos como fígado, coração e adrenal. A sobrecarga de ferro cardíaca usualmente implica pior prognóstico cardiovascular quando comparada à população geral com desfechos presentes em 73,2% *versus* 54,5% em um período de 3 anos. ^213^

A miocardiopatia por depósito de ferro, em fases iniciais, comumente se apresenta com função ventricular normal. O diagnóstico pelo ecocardiograma é muitas vezes um desafio, mas alguns achados podem sugerir o diagnóstico com história clínica compatível.Disfunção diastólica ventricular pode evoluir para fisiologia restritiva, progredindo até cardiopatia dilatada com FEVE reduzida. É possível a apresentação como constrição pericárdica causado pela deposição de ferro no pericárdio. A técnica de *speckle tracking* (STE) apresenta ainda papel conflitante nesse cenário, mas a maior sobrecarga cardíaca de ferro pode estar associada a um valor absoluto reduzido de SLG. Não há descrição pela literatura de um padrão de *strain* que seja específico de sobrecarga de ferro no miocárdio. Os resultados dos estudos que tentaram correlacionar *strain* circunferencial e radial com depósito de ferro foram conflitantes. O *speckle tracking* pelo ecocardiograma 3D também não tem papel estabelecido nesses pacientes, mas vem demostrando resultados favoráveis à detecção de disfunção subclínica precoce. ^214^^-^^216^

Embora a ecocardiografia possa ser usada para rastrear a sobrecarga de ferro, esse método não pode prever com precisão o conteúdo de ferro no miocárdio, diferentemente da RMC ( Tabela 7 ).

**Tabela 7:** Classificação da sobrecarga de ferro miocárdica (MIC) e hepática (LIC) pela ressonância magnética

T2* (ms) 1.5T	R2* (Hz) 1.5T	T2* (ms) 3.0T	R2* (Hz) 3.0T	MIC/LIC (mg/g dw)	Classificação
**Coração**					
> 20	≤ 50	>12,6	≤ 79	≤ 1,16	Normal
10 a 20	50 a 100	5,8 a 12,6	79 a 172	1,16 a 2,71	Sobrecargaleve/moderada
< 10	> 100	< 5,8	> 172	> 2,71	Sobrecarga grave
**Fígado**					
>15,4	≤ 65	> 8,4	≤ 119	≤ 2,0	Normal
4,5 a 15,4	66 a 224	2,3 a 8,4	120 a 435	2,0 a 7,0	Leve
2,1 a 4,5	225 a 475	1,05 a 2,3	436 a 952	7,0 a 15	Moderada
< 2,1	> 475	< 1,05	> 952	> 15	Grave

Devido a essa limitação, as diretrizes atuais de diversas sociedades internacionais e diferentes patologias recomendam o uso de rotina da ressonância magnética para determinação e seguimento do grau de sobrecarga de ferro nos diferentes órgãos. A indicação de ressonância magnética para esses pacientes deverá ser iniciada a partir da detecção de graus elevados de ferritina sérica > 1.000ng/mL ou recebimento de > 10 bolsas transfusionais naqueles pacientes com expectativa de vida superior a 12 meses. ^217^^,^^218^

**Figura 29:**
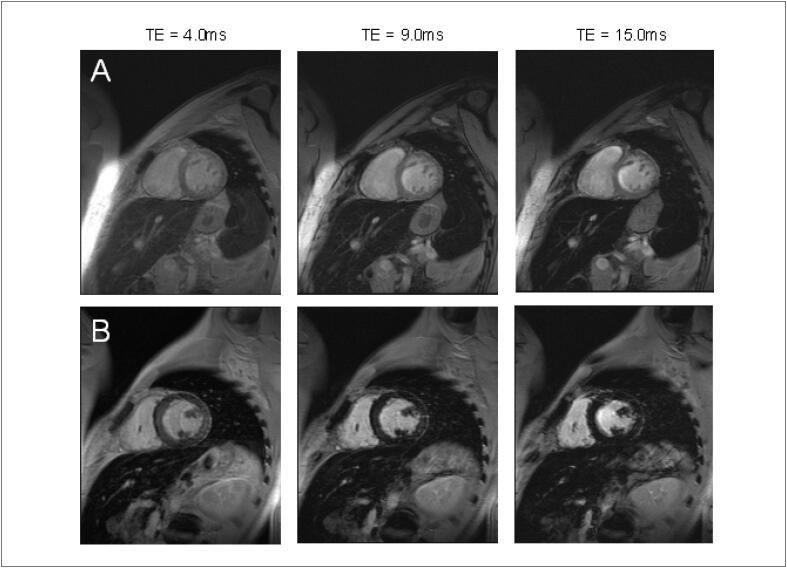
Exemplo de pacientes oncológicos com níveis normais de ferro miocárdico (A) e sobrecarga de ferro significativa (B). O paciente A tem T2* calculado em 22,8 ms com MIC (Myocardial Iron Concentration) = 0,98 mg/g; o paciente B tem T2* de 11,9ms com MIC de 2,2 mg/g. Observa-se o grau maior de escurecimento miocárdico no paciente B com o aumento dos valores de tempo de eco (TE), determinando visualmente o maior grau de sobrecarga miocárdica. O paciente B iniciou terapia de quelação oral de ferro após a determinação da sobrecarga miocárdica pela ressonância.

O exame de ressonância deve avaliar simultaneamente fígado e coração, sendo repetido anualmente ou conforme mudanças terapêuticas do quelante de ferro ou da carga transfusional. A quantificação é feita pelo efeito indireto que as moléculas de ferro exercem sobre o campo magnético local no tecido estudado. Logo, quanto maior a concentração de ferro tecidual, menor será o sinal do tecido medido (mais escuro) ( Figura 29 ).

Atualmente, o exame de ressonância não tem substituto específico, sendo o único exame capaz de determinar quantitativamente a sobrecarga de ferro em diferentes órgãos ao mesmo tempo de forma não invasiva, sem contraste ou radiação ionizante.

## 7. Pericardiopatias

### 7.1. Tumores do Pericárdio

Embora geralmente assintomática, a doença maligna pericárdica pode apresentar sintomas inespecíficos, como dispneia e taquicardia, que costumam estar relacionados com pericardite, constrição pericárdica, invasão miocárdica ou, mais comumente, derrame pericárdico (com ou sem tamponamento). O tamponamento ocorre em pacientes com derrame importante, sem sintomas prévios, em até 1/3 dos casos. ^219^

Em cerca de 20% a 50% dos pacientes com câncer e derrame pericárdico, a causa deste é envolvimento neoplásico, podendo em outros 30% ser decorrente do comprometimento linfático secundário à RT ou infecção viral. Em 10% a 25% dos casos, a presença de derrame pericárdico é o primeiro sinal de neoplasia. ^220^

Os tumores podem ser neoplasia pericárdica primária ou metástase envolvendo o pericárdio (neoplasia pericárdica secundária). A disseminação pode ocorrer via hematogênica, linfática ou invasão direta do pulmão e mediastino. Os tumores que causam mais metástases ao pericárdio são mama, pulmão, carcinoma de células renais, linfomas e melanomas, sendo o padrão histológico mais comum o adenocarcinoma. ^221^

Estudo recente mostrou que a presença de neoplasia hematológica, colorretal, ovariana, renal, pâncreas, mama e bexiga ocultos deve ser suspeitada em pacientes idosos, obesos e/ou tabagistas que apresentem quadro de pericardite aguda e necessidade de internação. O diagnóstico oncológico geralmente foi feito 3 a 12 meses após o episódio de pericardite. ^222^

#### 7.1.1. Ecocardiograma em Pacientes com Neoplasia Pericárdica

O derrame pericárdico pode ser avaliado de forma semiquantitativa pelo ETT. O aumento rápido do volume e a presença de contraste espontâneo no mesmo, com ou sem a visualização de massa pericárdica, são sugestivos de neoplasia pericárdica. Esta costuma apresentar aspecto sólido, ecogenicidade aumentada, podendo infiltrar o miocárdio. ^220^ Como diagnóstico diferencial, a gordura pericárdica tem baixa ecogenicidade e pode ser observada adjacente à parede livre do VD e junção atrioventricular.

Entre os tumores primários, o mesotelioma, originário das células mesoteliais do pericárdio visceral ou parietal, se apresenta com derrame pericárdico, tamponamento ou constrição, sendo que os nódulos no pericárdio podem invadir o miocárdio. Estudo retrospectivo com 64 pacientes com mesotelioma pericárdico maligno primário mostrou que as apresentações ecocardiográficas foram inespecíficas, com derrame pericárdico em 86% dos casos, sendo importantes em 67%, hemorrágicos em 95%, com presença de massas pericárdicas em 36% e espessamento em 17% dos casos.Tamponamento e pericardite constritiva ocorreram em 37% e 27%, respectivamente, sendo que o tamponamento pode estar relacionado com proliferação das células mesoteliais difusas e infiltração miocárdica, reduzindo o relaxamento e a complacência. Reacúmulo de líquido após a pericardiocentese ocorreu em 73% dos casos. ^223^

A mensuração da espessura do pericárdio é limitada pelo ecocardiograma. Esta limitação é superada quando as medidas são superiores a 5mm. De forma geral, a medida da espessura pericárdica é melhor realizada por tomografia computadorizada ou RMC, que permitem uma melhor avaliação do implante e da extensão tumoral. Assim, a associação dos métodos de multimodalidade mostra valor incremental. Já a técnica do *speckle tracking* permite a detecção de disfunção miocárdica subclínica por infiltração miocárdica precoce.

**Figura 30:**
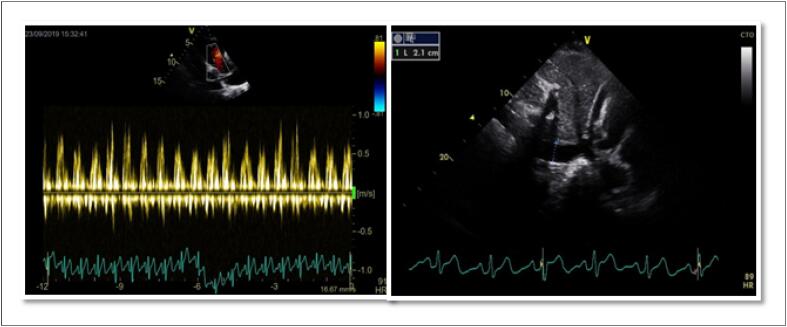
A. Variação do fluxo tricúspide maior que 40%. B. Veia cava inferior túrgida sem variação de seu calibre com manobras respiratórias (paciente com câncer de mama).

Normalmente, o líquido pericárdico só deve ser observado na sístole, mas, com o acúmulo, ele pode ser observado durante todo o ciclo cardíaco. O derrame pericárdico é considerado importante na presença de pelo menos 2cm de líquido pericárdico em volta do coração. No tamponamento cardíaco, há a compressão do coração pelo acúmulo de líquido no espaço pericárdico, o que leva ao aumento da pressão intrapericárdica que excede a intracavitária. Tal acúmulo pode ocorrer rápida ou gradualmente.O consequente colapso das cavidades cardíacas direitas, secundário ao aumento da pressão intrapericárdica, é mais proeminente na expiração, quando o enchimento de AD e VD é reduzido. Entretanto, em presença de HP, o colapso do AD é mais tardio, resistindo até que a pressão intrapericárdica supere as pressões elevadas nas cavidades direitas. Em casos de derrames volumosos, o coração costuma “balançar” dentro do líquido pericárdico a cada batimento cardíaco, podendo apresentar alternância elétrica no registro eletrocardiográfico. Observam-se ainda mudanças respiratórias dos fluxos cardíacos. O aumento das pressões no interior do VD durante a inspiração conduz a um desvio do septo interventricular para a esquerda e consequente aumento do fluxo e volume das câmaras direitas, além da dilatação da veia cava inferior ( Figura 30 A-B ).

A incisão subxifoide é padrão-ouro para acesso cirúrgico para realização de biópsia ou drenagem pericárdica. Entretanto, rotineiramente, a pericardiocentese é guiada por fluoroscopia ou ecocardiografia, com taxa de complicações de 4% a 10% dos casos. Durante a pericardiocentese guiada por ecocardiografia, o uso da solução salina agitada é útil para avaliar a posição da agulha antes de inserir o cateter e, depois, para avaliar a posição do cateter. ^224^ O derrame pericárdico é considerado recorrente se ocorre acúmulo de líquido dentro de 3meses pós-drenagem.

## 8. Cardio-Oncologia na População Infantojuvenil

### 8.1. Considerações Gerais

O número de casos novos de câncer infantojuvenis esperados para o Brasil, para cada ano do triênio 2020-2022, será de 4.310 casos novos no sexo masculino e de 4.150 para o sexo feminino. Esses valores correspondem a um risco estimado de 137 casos novos por milhão no sexo masculino e de 139 por milhão para o sexo feminino. O câncer infantojuvenil consiste em um conjunto de doenças que apresentam características próprias, em relação à histopatologia e ao comportamento clínico. Os tipos predominantes de cânceres pediátricos (entre 0 a 19 anos) são leucemia (28%), sistema nervoso central (26%) e linfomas (8%). Consecutivamente, observam-se tumores do sistema nervoso periférico (neuroblastomas), tumor de Wilms, retinoblastoma, tumores de células germinativas (ovários e testículos), sarcomas de partes moles e osteossarcoma. ^225^

As perspectivas de cura do câncer infantojuvenil são promissoras, com taxa atual de sobrevida na ordem de 80% em instituições qualificadas e diante do diagnóstico precoce. ^226^ Apesar do sucesso terapêutico, complicações inerentes aos fármacos quimioterápicos e/ou à RT comprometem vários sistemas orgânicos em curto, médio ou longo prazo. Existem evidências claras de que os sobreviventes de câncer tratados na infância com antraciclinas e/ou RT apresentam chances significativamente maiores de desenvolver complicações cardiovasculares ao longo de suas vidas, com estimativa que varia entre 5%e 30%, especialmente diante do protocolo utilizado e determinados fatores de risco. A incidência cumulativa de problemas crônicos graves pode chegar a 40% no intervalo de 30 anos de seguimento dos sobreviventes. ^227^

O câncer é a segunda causa mais comum de morte entre crianças de 1 a 14 anos nos EUA, perdendo apenas para os acidentes e mortes violentas. No Brasil, o câncer foi responsável por 8% do total de mortes entre crianças e adolescentes de 1 a 19 anos e 12% dos óbitos na faixa de 1 a 14 anos entre 2009 e 2013. Em 2014, 2.724 mortes por câncer infantojuvenil ocorreram no Brasil. ^225^ Essa realidade demonstra o quanto é importante essa interação entre onco-hematologistas e cardiologistas na segurança do tratamento do câncer infantojuvenil.

As complicações cardiovasculares são as principais causas de morbimortalidade nos sobreviventes de câncer tratados na infância, perdendo apenas para recorrências da própria doença e neoplasias secundárias. Os mecanismos de ação dos diversos quimioterápicos, bem como as lesões físicas determinadas pela RT, comportam-se de modo semelhante entre adultos, crianças e adolescentes. No entanto, a vulnerabilidade dessa faixa etária mais jovem é, particularmente, maior, por estarem em constante processo de crescimento e desenvolvimento físico. Além disso, os cardiomiócitosapresentam uma limitada capacidade de regeneração, e o metabolismo dos fármacos nessa população comporta-se de modo diferente, quando comparados aos adultos. ^228^ Desse modo, lesões consideradas de pequena gravidade na época do tratamento podem comprometer evolutivamente a função miocárdica ao longo do tempo e virem a descompensar na idade adulta.O inadequado ganho de massa ventricular esquerda e a queda da função contrátil, bem como a possível evolução para o modo restritivo de cardiomiopatia progressiva no decorrer dos anos, trazem sequelas cardiovasculares muitas vezes irreversíveis em alguns desses pacientes.

Além da disfunção miocárdica ventricular, outras complicações podem decorrer do emprego dos quimioterápicos: lesões do endotélio arterial (relacionadas ao efeito hipertensivo sistêmico e pulmonar), acometimento da circulação coronariana, lesões do endotélio venoso (relacionadas aos efeitos trombóticos), disfunções valvares, arritmias e pericardiopatias. ^229^

O comprometimento pericárdico pode estar relacionado à própria neoplasia, como também ser consequência da ação de alguns quimioterápicos ou secundários à metástase cardíaca. A pericardite, com ou sem derrame, poderá estar associada ou não à miocardite. A pericardite constritiva é mais associada à cardiotoxicidade induzida pela RT, sendo necessário diferenciá-la da miocardite restritiva. Os exames de imagem cardiovascular são essenciais na elucidação dessas alterações, especialmente em estágios subclínicos.

As antraciclinas (doxorrubicina, daunorrubicina, epirrubicina, idarrubicina e mitoxantrone) são agentes quimioterápicos extremamente potentes e amplamente usados na maioria dos protocolos de tratamento de neoplasias na infância. Existe uma forte relação entre a dose cumulativa de antraciclinas e o risco de insuficiência cardíaca, podendo haver, em alguns casos, um longo período de latência entre a exposição ao quimioterápico e o aparecimento de sintomas cardiovasculares. ^227^ A lesão miocárdica é progressiva e geralmente irreversível, culminando com disfunção cardíaca grave. A ação cardiotóxica das antraciclinasocorre através de vários mecanismos, sendo os mais evidentes a formação de radicais livres citoplasmáticos, resultando em estresse oxidativo nos miócitos, e à ação direta no núcleo, em que as antraciclinas agem sobre a topoisomerase-2 (Top2), impedindo a replicação do DNA que induz à morte celular. Apesar de a fração Top2α, foco do tratamento antineoplásico, estar expressa nas células tumorais, a fração Top2β se expressa nos cardiomiócitos, permitindo ação tóxica semelhante. ^230^ Os protocolos de tratamento onco-hematológicos atuais seguem uma tendência ao emprego de doses menores de antracíclicos. No entanto, mesmo com doses consideradas baixas, alguns pacientes pediátricos apresentam reações cardiotóxicas durante a evolução do tratamento e/ou no seguimento tardio, de forma que alguns autores consideram não haver dose segura. ^231^ A suscetibilidade individual ainda não está completamente elucidada, mas há hipóteses de que algumas características genéticas aumentem a vulnerabilidade e a precocidade da disfunção miocárdica. Algumas ações preventivas são recomendadas, especialmente nos pacientes estratificados como de alto risco de desenvolver complicações cardiovasculares. Estratégia primária, específica contra os efeitos cardiotóxicos das antraciclinas, é dada através da infusão do dexrazoxano, ou pela opção da doxorrubicinalipossomal (pouco utilizada entre pacientes jovens). Estratégia secundária, representada pela administração de fármacos com efeitos hemodinâmicos como os betabloqueadores, em especial, o carvedilol, inibidores da enzima conversora da angiotensina (IECA), e espironolactona, tem sido considerada como coadjuvante no cenário da prevenção ou no tratamento de lesões estabelecidas, cujos estudos prospectivos em crianças ainda estão em andamento. ^228^

Outras substâncias potencialmente cardiotóxicas são usadas para o tratamento de neoplasias em crianças e adolescentes, muitas vezes em associação com antraciclinas. A cardiotoxicidade não é rara com o uso de agentes alquilantes, como a ciclofosfamida, a cisplatina e a ifosfamida, sendo as apresentações mais frequentes a miocardite, as arritmias e a insuficiência cardíaca. A complicação cardiovascular mais comum dos antimetabólitos, como o 5-fluorouracil, é a isquemia miocárdica, relatada principalmente em adultos. Agentes biológicos como os inibidores da tirosino-quinase podem causar prolongamento do intervalo QT, insuficiência cardíaca, hipertensão arterial e IAM. O etoposido é uma podofilotoxina sintética aprovada para o tratamento de algumas neoplasias como sarcoma de Ewing e linfomas. As complicações cardiovasculares são raras, porém dor torácica, angina e IAM têm sido relatados em adultos recebendo etoposido em associação com bleomicina, cisplatina e ifosfamida. Agentes biológicos como as interleucinas e o interferon podem causar hipotensão ou arritmias. Os alcaloides da vinca, como a vincristina e a vimblastina, têm como principal efeito cardiovascular a isquemia miocárdica por vasospasmo coronariano. A imunoterapia também vem ocupando lugar no tratamento do câncer infantojuvenil, com potencial para desencadear miocardite, apesar de rara (< 1%) e pericardite. ^232^^,^^233^

A RT é um método terapêutico frequentemente utilizado no tratamento oncológico pediátrico (cerca de 40% dos protocolos). As complicações cardíacas decorrentes da RT devem-se à inflamação e à fibrose das estruturas cardíacas, podendo envolver pericárdio, miocárdio, valvas cardíacas e artérias coronárias. O pericárdio é a estrutura acometida com maior frequência. As lesões podem permanecer assintomáticas por 5 a 15 anos, mas geralmente são progressivas. A gravidade das lesões é proporcional à dose, volume irradiado e à associação com os quimioterápicos, o que confere maior risco. Os avanços nas técnicas de RT, nas duas últimas décadas, permitiram uma redução no volume e nas doses de radiação na área cardíaca. Com o surgimento de técnicas de planejamento 3D, o delineamento das estruturas adjacentes, o ajuste de doses e a modificação de campos permitiram a redução significativa da dose sobre órgãos de risco, como o coração. ^227^^,^^234^

### 8.2. Principais Fatores de Risco para o Desenvolvimento de Cardiotoxicidade entre Crianças e Adolescentes

**Dose cumulativa:** mesmo doses relativamente baixas de antraciclinas têm sido relatadas como desencadeantes de cardiotoxicidade. O conceito de dose segura de antraciclinas tem sido desconsideradopor alguns autores, como observadoem estudo que mostrou um aumento de 30% de anormalidades ecocardiográficas subclínicas 13 anos após o tratamento de leucemia, mesmo com doses cumulativas de antraciclinas entre 180 e 240mg/m^2^. ^234^^,^^235^

**Associação entre agentes quimioterápicos:** a administração concomitante de mais de um agente quimioterápico sabidamente cardiotóxico não só facilita, como potencializa os efeitos cardiovasculares adversos.

**Idade ao tratamento:** crianças tratadas antes dos 5 anos de idade apresentam maiores chances de complicações cardiovasculares em curto ou em longo prazo, especialmente devidoà limitação funcional miocárdica progressiva diante da demanda do crescimento físico.

**Sexo:** meninas apresentam duas vezes maior incidência de cardiotoxicidade que meninos, provavelmente devido ao maior percentual de gordura corporal que permite maior tempo de exposição e concentração corporal das antraciclinas.

**Velocidade de infusão:** alguns estudos, em adultos, sugeriram uma redução na prevalência de cardiotoxicidade através da infusão contínua de antracíclicos, quando comparada com a administração em *bolus.* Este modo de infusão, porém, não foi cardioprotetor alongo prazo na população pediátrica. ^231^

**Radioterapia mediastinal e neuroeixo:** doses radioterápicas ≥ 30 Gy ou a combinação de antraciclina com RT, mesmo em baixas doses, também representam um agravante ao risco de desenvolvimento de complicações cardiovasculares. A baixa idade quando da exposição à radiação também é fator importante. Esses pacientes podem evoluir com complicações valvares, pericárdicas, miocardiopatia restritiva, coronariopatias e arritmias que podem induzir à morte súbita. ^227^^,^^234^

**Radioterapia do sistema nervoso central:** a longo prazo, crianças que receberam irradiação cranial apresentaram maior perda de massa ventricular, fato atribuído ao comprometimento da hipófise com consequente deficiência secundária da produção de IGF-1, precursor do hormônio do crescimento (GH). ^236^

**Comorbidades:** a presença de duas ou mais comorbidades incrementam o risco cardiovascular nos adultos sobreviventes ao tratamento do câncer na idade pediátrica. O desenvolvimento de hipertensão arterial sistêmica, DM, dislipidemia e DAC, especialmente em associaçãoà obesidade e ao tabagismo, incrementa a precocidade do surgimento das complicações quando comparado aos seus irmãos. A presença de disfunção hepática, disfunção renal e distúrbios eletrolíticos compromete o *clearance* do fármaco, lentifica sua eliminação e aumenta o tempo de exposição ao medicamento.

**Variantes genéticas:** a predisposição individual à cardiotoxicidade tem sido reportada em diversos estudos. Algumas variantes genéticas, como a RARG e UGT1A6, aumentam a Top2 ou lentificam o metabolismo dos fármacos. A incidência de disfunção miocárdica aumenta nove vezes em pacientes portadores da mutação do gene C282Y para hemocromatose hereditária. Portadores de trissomia 21 trazem consigo maiores chances de desenvolver leucemia mieloide aguda e são mais sensíveis aos efeitos tóxicos dos quimioterápicos. Cardiomiopatias familiares (hipertróficas, arritmogênicas) e portadores de cardiopatias congênitas podem apresentar miocárdios mais vulneráveis. ^237^^,^^238^

### 8.3. Monitoramento Cardiológico durante o Tratamento

A metodologia aplicada aos exames de imagem cardiovascular, definições e parâmetros utilizados na avaliação de pacientes infantojuvenis ou sobreviventes ao tratamento do câncer na idade pediátrica são os mesmos aplicados à população adulta.

A ecocardiografia é o principal método indicado para o monitoramento desses pacientes, pois permite a análise dos principais parâmetros de função diastólica e sistólica de ambos os ventrículos, estado anatômico e funcional das estruturas valvares e do pericárdio, além da investigação de massas, trombos e vegetações. A função miocárdica ventricular é quantificada através de diversos parâmetros e metodologias. Os mais preconizados são a FEVE através do método de Simpson biplanar e a análise dos índices de deformação miocárdica ( *strain* ), pela técnica de *speckle tracking* . As medidas de deformação miocárdica podem ser obtidas por ecocardiografia 2De 3De analisadas nos modos longitudinal, radial ou circunferencial, uma vez que não dependem do ângulo de incidência do ultrassom. Esse método tem sido alvo de vários estudos que procuram detectar precocemente alterações subclínicas que podem ser preditoras de cardiotoxicidade na população infantojuvenil. ^239^ O modo longitudinal tem sido o de melhor reprodutibilidade utilizado na grande maioria dos estudos. Apesar de suas vantagens (disponibilidade, acessibilidade e baixo custo), a ecocardiografia apresenta algumas limitações, em especial a variabilidade inter-observador e o comprometimento da qualidade das imagens obtidas diante de janelas acústicas inadequadas. Outra limitação está relacionada com fração de ejeção, comprovadamente de baixa sensibilidade no contexto da detecção precoce de alterações funcionais miocárdicas. Em contrapartida, a análise da deformação miocárdica através da técnica de *speckletracking* permite identificar lesões subclínicas que precedem a queda da FEVE. ^239^^,^^240^

A RMC é outro importante método diagnóstico no contexto cardio-oncológico infantojuvenil. Além de ser opadrão-ouro na quantificação da FEVE, permite também a detecção de edema intersticial precoce e fibrose miocárdica através de métodos contemporâneos associados à utilização de contraste (gadolínio) pelo mapeamento em T1 e identificação de realce tardio. Além disso, tem importante papel na caracterização de massas intra e extracardíacas. Entretanto, devido às suas características intrínsecas, temlimitações quanto ao seu uso como rotina.

A aplicabilidade da MN na área pediátrica exige, além do conhecimento técnico, a necessária avaliação conjunta com a equipe pediátrica sobre o risco *versus* benefício da exposição do paciente infantojuvenil às doses de radiação (embora baixas, com base nos princípios de segurança de radiação). ^241^ A ventriculografia radioisotópica (VR), técnica não invasiva que utiliza as hemácias marcadas com ^99m^Tc como radiofármaco, permite a análise de vários parâmetros da função ventricular (curva de atividade *vs.* tempo, análise de fase e amplitude), possibilitando uma refinada avaliação das regiões de melhor desempenho contrátil (amplitude) e o momento temporal em que esta contração ocorreu (análise de fase), detectando a presença de assincronismo contrátil entre os ventrículos direito e esquerdo e intraventricular esquerdo. No entanto, em relação à função diastólica, as informações fornecidas são limitadas. É discutível o momento ideal da realização da VR durante o tratamento oncológico a fim de minimizar as variáveis que possam confundir a investigação. Segere-se a realização da mesma pelo menos 3 semanas após a terapia com antraciclina, quando os pacientes estão afebris e com hemoglobina superior a 9 g/dL. ^242^ Com relação aos outros métodos nucleares empregados na detecção de cardiotoxicidade como a captação miocárdica de mIBG ^123^ I e PET-CT, ainda não há dados robustos para indicação na população pediátrica.

O diagnóstico de cardiotoxicidade através dos métodos de imagem cardiovascular é feito com base nas alterações detectadas em relação ao exame basal. Desse modo, recomenda-se realizar a avaliação funcional antes do início do tratamento antineoplásico e durante a evolução, como análise comparativa, procurando sempre utilizar os mesmos equipamentos, os mesmos métodos e as mesmas variáveis utilizadas nas avaliações anteriores.

A Tabela 8 mostra as recomendações internacionais de monitoramento ecocardiográfico de pacientes durante e após o tratamento com antraciclinas (principais representantes da cardiotoxicidade na faixa etária infantojuvenil).

**Tabela 8:** Monitoramento ecocardiográfico de pacientes tratados com antraciclinas na infância

DC (mg/m^2^)	Durante o tratamento		Após o tratamento	
	Sem FRP	01 ou + FRP	Sem FRP	01 ou + FRP
Inicial	Sim	Sim	NA	NA
< 200	– Critério clínico	– Cada 2/3 ciclos	– 1 mêspós-TT – Avaliar em 1, 2 e 5 anos – Individualizar SN	– 1 mês pós-TT – Avaliar em 1, 2 e 5 anos
200 a 300	– Critério clínico	– Cada 2/3 ciclos	– 1, 6 e 12 meses pós-TT – Depois: a cada 2 anos (assintomático)	– 1, 6 e 12 meses pós-TT – Depois: anual – Individualizar
300 a 450	– Critério clínico – Individualizar SN	– Cada 2 ciclos	– 1, 6 e 12 meses pós-TT – Depois: anual – Individualizar	– 01 mês pós-TT – Depois: 6/6 meses – Individualizar SN
> 450	– Metade do TT – Individualizar SN	– Cada 2 ciclos – Individualizar SN	– 1 mês pós-TT – Depois: 6/6 meses – Individualizar SN	

DC: dose cumulativa; FRP: fatores de risco preexistentes; TT: tratamento; SN: se necessário; NA: não se aplica.

É necessário levar em consideração o estado hemodinâmico do paciente no momento da avaliação. Sempre que possível, considerar o estado mais estável possível. A fragilidade do paciente pediátrico diante de determinadas situações, como alterações da volemia (desidratação, hiper-hidratação), anemia, febre, hipotermia, choque, sepse, frequência cardíaca e alterações do ritmo, pode influenciar, pontualmente, na interpretação dos resultados. Essas condições afetam a pré e pós-carga e são suficientes para promover uma variação de 5% a 10% no volume sistólico por ciclo cardíaco, com influência na fração de ejeção. As alterações da contratilidade global e segmentar, durante a QT, podem estar relacionadas à cardiomiopatia secundária induzida por estresse transitório (idiopática, sepse, lesão neurológica, liberação de catecolaminas). ^243^ As alterações funcionais diante de intercorrências clínicas poderão ser transitórias ou definitivas. Recomenda-se a reavaliação logo após a compensação do quadro.

A definição de cardiotoxicidade subclínica continua sendo um desafio também para a população infantojuvenil. Alguns estudos relatam alterações segmentares mesmo antes da queda do *strain* global longitudinal, o que tem levantado a atenção quanto à maior acurácia desse método na detecção precoce de disfunção miocárdica. As atuais recomendações internacionais consideram a queda percentual de 15% do *strain* global longitudinal, comparados com os valores iniciais (basais), como indicativa de disfunção subclínica, desde que em condições hemodinâmicas comparativamente semelhantes. ^239^

### 8.4. Seguimento a Longo Prazo dos Sobreviventes

O risco de desenvolvimento de complicações cardiovasculares e a gravidade das lesões aumentam com o passar do tempo. Estudos demonstraram que, entre sobreviventes de câncer tratados na infância e acompanhados por 30 anos, 8% apresentaram insuficiência cardíaca. A detecção precoce da cardiotoxicidade induzida pelos tratamentos antineoplásicos parece ter um efeito significativo no controle da evolução e nos efeitos hemodinâmicos decorrentes da lesão miocárdica. A identificação do melhor método para a detecção precoce da cardiotoxicidade nesses pacientes é de extrema importância. A ecocardiografia e a RMC são os métodos mais recomendados pelas principais diretrizes e estudos internacionais. ^28^^,^^227^^,^^239^

Apesar da escassez de estudos controlados e randomizados na população infantojuvenil, a detecção precoce das lesões desde o estágio subclínico é de fundamental importância para impedir a sua progressão, prevenindo ou revertendo o remodelamento patológico do VE. ^228^

A Sociedade Americana de Oncologia Clínica (ASCO) propõe cinco questões clínicas que podem orientar no planejamento e monitoramento das complicações cardiovasculares dos sobreviventes: ^6^

1. Quais pacientes têm risco aumentado de evoluir com disfunção cardíaca?
2. Quais as estratégias preventivas para minimizar o risco ANTES do início do tratamento?
3. Quais as estratégias preventivas para minimizar os riscos DURANTE o tratamento com fármacospotencialmente cardiotóxicos?
4. Quais os métodos de escolha para monitorar os pacientes de risco DURANTE o tratamento?
5. Quais os métodos de escolha para monitorar os pacientes de risco APÓS o tratamento?

O planejamento de seguimento tardio com basena estratificação dos riscos permite a escolha do melhor método de monitoramento da função miocárdica (biomarcadores e exames de imagem), visando à detecção precoce de disfunção subclínica, com consequente utilização de fármacosque possam prevenir ou reverter o remodelamento do coração.

Com relação ao seguimento tardio após a RT – um dos pilares do tratamento oncológico pediátrico, onde cerca de 40% das crianças, adolescentes e adultos jovens são submetidos em alguma etapa do tratamento da doença – a incidência e a gravidadesão diretamente proporcionais à dose e ao volume irradiado e inversamente proporcionais à idade. A ocorrência também aumenta com o tempo decorrido da RT e uso associado de agentes cardiotóxicos, em particular as antraciclinas, além de eventual doença cardíaca prévia. ^34^^,^^234^ A radiação leva à fibrose miocárdica intersticial difusa, especialmente da parede anterior do VE, relacionada à área irradiada, cuja manifestação clínica será expressa como cardiomiopatia restritiva. Diante da dificuldade de avaliação da função diastólica em crianças, os valores encontrados devem ser comparados com os valores basais do próprio paciente. O dano endotelial favorece o desenvolvimento precoce de placas ateroscleróticas complicadas por hemorragia e inflamação com riscos de ruptura e trombose. O pericárdio é o principal alvo de lesãousualmente iniciada por pericardite fibrinosa e derrame pericárdico, seguidos por espessamento fibroso preferencialmente no pericárdio parietal e VD, podendo estender-se ao mediastino. O comprometimento valvar é menos frequente e, ao contrário do que ocorre no pericárdio, atinge mais o lado esquerdo do coração. As cúspides tornam-se espessadas, podem calcificar e levar à insuficiência e estenose, principalmente das valvas mitral e aórtica, poupando as pontas das cúspides e a região das comissuras valvares. ^244^

Os sobreviventes ao tratamento do câncer na fase infantojuvenil são mais vulneráveis ao desenvolvimento de DAC prematura, inclusive subclínica, com risco de infarto do miocárdio 2 a 8 vezes maior que a população geral. Como os eventos coronarianos são raros em pacientes mais jovens, mesmo naqueles em risco aumentado, o ecocardiograma com estresse farmacológico e exames de imagem nuclear para avaliação da perfusão e viabilidade miocárdicas estão recomendados. ^245^

Os controles tardios em indivíduos sem disfunção ventricular devem ser realizados de rotina após 6 meses a 1 ano e, depois, periodicamente, uma vez que a incidência de insuficiência cardíaca relacionada ao uso de antraciclina, assim como após RT, aumenta com o acompanhamento tardio, podendo surgir depois de 15 anos ou mais.Embora a frequência ideal para avaliação da estrutura e função do VE em sobreviventes expostos à radiação cardíaca não esteja ainda bem estabelecida entre os consensos, quanto maior o número defatores de risco, mais frequentes essas avaliações deverão ser realizadas. ^246^

### 8.5. Gravidez nas Sobreviventes do Câncer Infantojuvenil

Com os avanços no tratamento oncológico e o notável número de sobreviventes do câncer infantojuvenil, um grande número de mulheres atinge a idade fértil e muitas optarão por engravidar. Algumas são portadoras de lesões subclínicas não diagnosticadas e outras se encontram na vigência de tratamento para insuficiência cardíaca ou outras complicações tardias do tratamento antineoplásico. A gravidez está associada a mudanças substanciais no sistema cardiovascular, uma vez que existe um aumento da demanda metabólica no coração da gestante. Aumentos marcantes no volume sanguíneo circulante contribuem para um incremento de 30% a 40% no débito cardíaco em um período relativamente precoce da gestação (20 a 24 semanas), alterações que influenciam na interpretação do *status* cardiovascular. Como consequência, há taquicardia e anemia relativa, além de fenômenos de hipercoagulabilidade.O risco de desenvolvimento de eventos cardíacos durante a gravidez de mulheres previamente expostas a antraciclinas e/ou RT torácica na infância não é claramente conhecido, com poucos dados disponíveis na literatura. ^247^^,^^248^

Regurgitações multivalvulares fisiológicas, especialmente nas câmaras direitas, por aumento volumétrico das câmaras cardíacas, dilatação dos anéis valvares, bem como derrame pericárdico discreto, são frequentes no final da gestação e no período pós-parto, e parecem ser causados por hipervolemia decorrente desse período.

A Tabela 9 relaciona as alterações cardiovasculares fisiológicas maternas de acordo com o trimestre da gestação.

**Tabela 9:** Mudanças ecocardiográficas fisiológicas durante a gravidez

1° Trimestre		2° Trimestre		3° Trimestre		Pós-parto	
↓	RVS	↓	RVS	↓	RVS SLG	↓	FC DDVE Massa VE DC
↑	FC DDVE Massa VE DC	↑	FC DDVE Massa VE DC	↑	FC DDVE Massa VE DC	↑	RVS SLG

RVS:resistência vascular sistêmica; FC: frequência cardíaca; DDVE: diâmetro diastólico do VE; VE: ventrículo esquerdo; SLG: *strain* longitudinal global; DC: débito cardíaco; ↑ = aumenta;↓ = diminui.

#### 8.5.1. Desfechos Cardíacos em Gestantes Sobreviventes do Câncer Infantojuvenil

Alguns estudos clínicos avaliaram desfechos cardíacos durante a gravidez em mulheres previamente expostas à terapia oncológica cardiotóxica.

Van Dalen et al. ^247^ avaliaram, retrospectivamente,53 mulheres sobreviventes ao câncer infantojuvenil que tiveram um ou mais filhos. Dados da função sistólica ventricular esquerda e outros parâmetros ecocardiográficos não estavam disponíveis para análise. O diagnóstico de insuficiência cardíaca foi definido com base em sinais e sintomas. Nenhuma paciente desenvolveu insuficiência cardíaca durante a gravidez ou nos 5 meses após o parto. ^247^

Hines et al. ^248^ aplicaram um questionário a 847 mulheres sobreviventes ao câncer infantojuvenil que tiveram pelo menos uma gravidez completa. Os autores constataram que a maioria das mulheres sobreviventes ao câncer infantojuvenil não apresentoucomplicações cardíacas durante ou após o parto; no entanto, gestantes com histórico de terapias cardiotóxicas devem ser seguidas cuidadosamente durante a gravidez, principalmente aquelas com histórico de exposições à antraciclina e queforamdiagnosticadas com cardiomiopatia subclínica ou sintomática anterior ou atual. ^248^

Em outro estudo, 37 mulheres que receberam doxorrubicina como parte de um protocolo de QT para um distúrbio neoplásico na infância foram acompanhadas no mesmo centro durante as gravidezes (72 gestações) e após o parto.Os autores concluíram que o resultado da gravidez em mulheres que receberam doxorrubicina por malignidade na infância é geralmente favorável. No entanto, aquelas com disfunção ventricular esquerda antes da gestação devem ser consideradas com risco aumentado para pior resultado na gravidez e deterioração adicional da função miocárdica. ^249^

Thompson et al. ^250^ avaliaram uma coorte de 58 mulheres que foram tratadas com antraciclinas e/ou radiação torácica antes dos 20 anos de idade, no *MD Anderson Cancer Center* . A incidência de eventos cardíacos adversos (definidos como FEVE < 50% em pelo menos dois ecocardiogramas ou DAC) foi significativamente maior nas mulheres que tiveram pelo menos uma gravidez (29%) em comparação com um grupo controle de nulíparas pareadas por dose de antraciclina e período de acompanhamento (15%), p < 0,05. Dentre as 58 mulheres grávidas, nove foram diagnosticadas com doença cardiovascular durante a gravidez e cinco foram diagnosticadas posteriormente à gravidez. Nessa pequena coorte, o intervalo entre a exposição às antraciclinas até a primeira gravidez, bem como a dose total de antraciclinas, esteve associado a um risco aumentado de eventos cardíacos adversos. Além disso, a própria gravidez esteva associada a um aumento de 2,4 vezes no risco de desenvolvimento de eventos cardíacos adversos (IC 95%:1,02-5,41, p = 0,045). ^250^

#### 8.5.2. Recomendação de Monitoramento Cardiovascular em Sobreviventes do Câncer Infantojuvenil com Desejo de Gestar

Estudos em populações não oncológicas com cardiomiopatias preexistentes relataram um alto risco de descompensação cardíaca devido às mudanças na fisiologia cardíaca durante a gravidez.A gravidez não está recomendada em portadoras de cardiomiopatias com FEVE < 40%. ^251^

Em sobreviventes do câncer infantojuvenil, existem evidências limitadas sobre monitoramento cardíaco na gravidez. Diretrizes internacionais recomendam uma avaliação cardiovascular antes da gravidez e no primeiro trimestre para todas as mulheres que foram tratadas durante a infância com antraciclinas e/ou RT torácica. *Children’s Oncology Group* recomenda a realização de um ecocardiograma antes e periodicamente durante a gravidez (especialmente durante o terceiro trimestre), além do monitoramento cardíaco durante o trabalho de parto e parto em pacientes que receberam doses de antraciclinas> 300 mg/m^2^, doses de radiação torácica > 30 Gy, e que receberam ambos tratamentos oncológicos, antraciclinas e RT torácica. ^246^^,^^251^

Recomenda-se então:

- Avaliação da função cardíaca pelo ecocardiograma antes da gravidez e, periodicamente, a cada trimestre, em mulheres que foram tratadas com antraciclinas (principalmente em doses > 300mg/m^2^) e/ou RT torácica (especialmente em doses > 30 Gy) com FEVE prévia ≥ 50%;
- Avaliação da função cardíaca pelo ecocardiograma antes da gravidez e no último trimestre de gestação, ou quando indicação clínica, em mulheres que foram tratadas com antraciclinas (doses < 300mg/m^2^) e/ou RT torácica (doses < 30 Gy) com FEVE prévia ≥ 50%;
- Avaliação da função cardíaca pelo ecocardiograma antes da gravidez e, periodicamente, a cada trimestre, ou quando indicação clínica, além de monitoramento cardíaco durante o trabalho de parto e parto em mulheres com FEVE entre 40 e 50%. Considerar a repetição do ecocardiograma um mês após o parto;
- Gravidez não é recomendável em pacientes com FEVE < 40%.

### 8.6. Situações Predisponentes a Eventos Trombóticos Relacionados ao Tratamento de Câncer Infantojuvenil

#### 8.6.1. Trombo Intracardíaco

Grande parte do tratamento quimioterápico dos pacientes com câncer infantojuvenil é realizada por via endovenosa através de cateteres de longa permanência, aumentando o risco para formação de trombos ou vegetações. Além disso, as propriedades pró-coagulantes das células tumorais facilitam a invasão tumoral e as metástases. ^252^

Na população pediátrica, a incidência média de eventos trombóticos (ET) associados ao câncer é de 8%, com maior frequência nasleucemias linfoblásticas agudas (LLA), seguida porsarcomas, linfomas, leucemia mieloide aguda (LMA), tumor de Wilms, neuroblastomas e tumor de sistema nervoso central.Entre as leucemias, a ocorrência pode atingir 36,7%; linfomas (devido à compressão extrínseca de massa no mediastino), tumores sólidos e tumores do sistema nervoso central, até 16%; dentre os sarcomas, o de Ewing é o que mais apresenta riscos para ET, seguido porrabdomiossarcoma e osteossarcoma. ^253^

Várias condições clínicas são relacionadas à etiologia dos ET em crianças e adolescentes: cirurgias recentes, doença cardíaca congênita, imobilização, trauma, síndrome nefrótica, uso de contraceptivos orais, trombofilia congênita e presença de cateter venoso central (CVC), que é o fator predisponente isolado mais importante. ^252^ A L-asparaginase, utilizada em protocolos de leucemia linfoblástica aguda (LLA), pode suprimir anticoagulantes naturais, particularmente antitrombina e plasminogênio. Uso de corticosteroide pode levar à ativação do complexo FVIII-von Willebrande à ativação do inibidor plasminogênio (PAI-1). A combinação de L-asparaginase e corticosteroideé um fator de alto risco para o desenvolvimento de ET, principalmente na fase de indução, em queocorre uma redução da capacidade de inibir a trombina. ^253^

O tromboembolismo pulmonar é bem menos relatado entre crianças e adolescentes, quando comparados aos adultos. Em estudo canadense, foi demonstrada a incidência de 0,86 por 10.000 admissões hospitalares; porém, há escassez de registros de seguimento alongo prazo. ^254^

As características genéticas do hospedeiro também influenciam na maior vulnerabilidade aos ET. A deficiência de anticoagulantes naturais como antitrombina, proteína C e proteína S éo fator genético mais frequentemente relacionado comET na população pediátrica. ^252^

O ecocardiograma transtorácico (ETT) é uma importante ferramenta para a pesquisa de trombos e vegetações intracardíacas, complementado pela ecocardiografia transesofágica (ETE), que apresenta maior sensibilidade. O uso de contraste de microbolhas pode ajudar a detectar trombos e diferenciar trombo avascular de tumores vascularizados. Os trombos intracardíacos são caracterizados por terem aspecto hiperecogênico e bordas geralmente regulares. A ressonância magnética e a angiotomografia podem ser adjuvantes para elucidação diagnóstica; mesmo assim, pode ser difícil diferenciar trombos de outros tumores cardíacos. O diagnóstico de trombo intracardíaco é fruto da combinação da apresentação da imagem, localização e quadro clínico. Em casos incertos, o diagnóstico é frequentemente realizado após um ciclo de anticoagulação e reavaliação pelo método de imagem. ^255^

#### 8.6.2. Cateter Venoso Central

Estima-se que, nos EUA, mais de 5 milhões de cateteres venosos centrais (CVC) sejam implantados anualmente. Crianças com câncer requerem administração endovenosa de quimioterápicos por período considerável de tempo. O uso de CVC após a década de 80 melhorou a qualidade de vida desses pacientes, porém o uso está associado a complicações mecânicas, infecciosas e trombóticas. A incidência de TRC em pacientes oncológicos pediátricos relatada nos últimos anos variou de 4,6% a 7%. ^252^^,^^256^

O ecocardiograma tem papel fundamental na avaliação deste dispositivo. A ponta do cateter deve estar idealmente localizada na veia cava superior próximo à junção com o átrio direito para evitar arritmias e formação de trombos. Deve-se avaliar todo o trajeto venoso visível, bem como o comportamento do fluxo próximo ao dispositivo, nas cavidades cardíacas e nas valvas. ^256^

#### 8.6.3. Diagnóstico Diferencial de Massa Intracardíaca

##### 8.6.3.1. Crista Terminalis Proeminente

A *crista terminalis* é um tecido fibromuscular que demarca os limites do seio venoso embriológico e a parede muscular do átrio direito. Quando proeminente, pode ser equivocadamente diagnosticada como uma massa intracardíaca. Ao ecocardiograma, apresenta-se como uma imagem hiperecogênica na porção posterolateral do átrio direito. Seu conhecimento evita o diagnóstico incorreto de trombo intracardíaco.

##### 8.6.3.2. Calcificação do Anel Mitral

A calcificação do anel mitral (CAM) é a deposição de cálcio e de gordura ao longo do anel fibroso, mais comumente na porção posterior do anel valvar mitral, com aspecto hiperecogênico. Pode ser base para formação de trombos ou vegetações e apresentar componentes móveis calcificados com potencial para embolização.

##### 8.6.3.3. Endocardite Infecciosa

Em pacientes em tratamento de câncer, com hemocultura positiva, deve-se suspeitar do diagnóstico de endocardite infecciosa (EI), visto que esses doentes têmalto risco para infecções hospitalares pelo número de internações e pela imunossupressão. O ETT é mandatório nesses casos. A investigação inclui as valvas cardíacas em busca de sinais de vegetação, abscessos ou fístulas cardíacas, presença de nova regurgitação valvar, além da aparência dos dispositivos endovenosos de longa permanência. A especificidade para o diagnóstico de vegetação endocárdica através do ETT é superior a 90%, porém a sensibilidade varia de 62% a 79%, vistoque imagens menores que 2 a 3mm podem não ser visibilizadas. O ETE possui também especificidade superior a 90%; contudo, temsensibilidade maior para o diagnóstico de EI (> 80% a 90%).

As vegetações endocárdicas têm como características o formato bastante irregular, movimento caótico que independe do movimento valvar, localizam-se habitualmente no lado atrial das valvas atrioventriculares e no lado ventricular das valvas semilunares e associam-se à destruição do aparato valvar, levando à regurgitação ou abscesso valvar. Os abscessos valvares acometem mais comumente a valva aórtica e podem fistulizar para o VE ou para o átrio. Há risco aumentado para acidente vascular encefálico (AVE) e IAM nos pacientes diagnosticados com EI. Os principais agentes responsáveis pelos AVE são as vegetações endocárdicas com dimensões maiores que 10 a 15mm, que acometem mais de uma valva, têmmobilidade excessiva, hemocultura positiva para fungo, *S. aureus* e *S. bovis* .

##### 8.6.3.4. Endocardite Trombótica Não Bacteriana

**Libman-Sacks:** composta por material granular com imunocomplexos e plaquetas, sem bactérias; geralmente tem dimensões pequenas (1 a 4 mm), localiza-se nos mesmos locais que a endocardite, porém é usualmente menos irregular e mais bem delimitada e não está associada à destruição valvar. São lesões, habitualmente, assintomáticas e mais comuns em pacientes com diagnóstico de lúpus.

**Endocardite marântica:** a endocardite marântica é um tipo de endocardite não infecciosa associada à malignidade relacionada com carcinomas sólidos metastáticos e adenocarcinomas de pulmão, pâncreas, gástrico e outros adenocarcinomas. Há descrições de associação com síndromes mielodisplásicas. As vegetações marânticas são compostas por fibrina e plaquetas e raramente leva à disfunção valvar; classicamente, afeta a face atrial da valva mitral e a face ventricular da valva aórtica. Pode embolizar em mais de 50% dos casos. A ecotextura, as dimensões e a localização não são diferentes da EI. No entanto, observa-se frequentemente espessamento valvar difuso significativo, o que pode ajudar no diagnóstico.

##### 8.6.3.5. Excrescência de Lambl

A excrescência de Lambl pode ser definida como estrutura filiforme, menor que 2 mm de largura e 3 a 10mm de comprimento, com movimento ondulante localizado na face atrial da valva mitral e na face ventricular da valva aórtica. Não está associada a eventos tromboembólicos.

### 8.6. Avaliação Cardiovascular Diante do Transplante de Medula Óssea em Pacientes Infantojuvenis

O transplante de medula óssea (TMO), ou transplante de células-tronco hematopoiéticas, seja do tipo autólogo ou do tipo alogênico, tem participação importante no tratamento de diversas neoplasias na infância e adolescência. As mais frequentes são as leucemias, alguns tumores sólidos, aplasia de medula óssea, imunodeficiências, além de algumas doenças hereditárias que acometem o sistema hematopoiético.

Os transplantes autólogos são, em geral, menos passíveis de complicações cardiovasculares, diferentemente dos alogênicos. A incidência de complicações cardiovasculares aumenta de acordo com o tempo de cura desses pacientes, podendo atingir até 17% a partir de 15 anos após o tratamento. Os sobreviventes de TMO apresentam 13 vezes mais risco de complicações cardiovasculares quando comparados aos seus irmãos. ^257^

Fatores que favorecem a toxicidade cardiovascular após o TMO:

- Idade ao TMO;
- QTcardiotóxica anterior (p. ex., antraciclinas);
- Regime de condicionamento mieloablativo (p. ex., ciclofosfamida);
- Irradiação torácica prévia;
- Irradiação corporal total ( *total body irradiation* [TBI]);
- Tipo de transplante;
- Presença de doença do enxerto contra o hospedeiro (DECH);
- Comorbidades (hipertensão arterial, DM, dislipidemia, tabaco, obesidade).

A DECH é a complicação mais frequentemente relacionada ao TMO alogênico e requer avaliações regulares através dos métodos de imagem cardiovascular. Ela desencadeia reações imunológicas que levam ao dano endotelial e processos inflamatórios crônicos, acomete vários órgãos, inclusive os pulmões, podendo simular quadro de hipertensão arterial pulmonar. Favorece o desenvolvimento de vasculite, aterosclerose precoce, hipertensão arterial sistêmica, doenças cerebrovasculares, angina e IAM. ^258^ O uso prolongado de corticosteroide e de outros imunossupressores no tratamento da DECH acelera esses eventos, e a presença de fatores de riscos cardiovasculares convencionais contribuicom o surgimento dessas complicações. O comprometimento cardíaco isolado da DECH é raro e pode se apresentar na forma de pericardite, arritmias e DAC. ^257^

Outro fator a ser considerado é o depósito de ferro no miocárdio, decorrente das múltiplas transfusões sanguíneas, que pode persistir por anos após o TMO e usualmente é avaliadopor meiode ressonância magnética cardíaca e hepática. ^259^

Os sobreviventes de TMO tratados com antraciclinas e/ou irradiação torácica devem ser examinados anualmente, em busca desinais de complicações cardiovasculares. A função cardíaca (sistólica e diastólica) deverá ser avaliada por meioda ecocardiografia, anualmente ou a cada 5 anos, dependendo das doses e da idade a que foram submetidos. Devem ser orientados, preventivamente, quanto aos fatores de risco cardiovasculares modificáveis (hipertensão arterial, DM, dislipidemia, tabagismo).

## Lista de Abreviaturas

^18^ F-FDG: ^18^ F-Fluordesoxiglicose
APBI: Accelerated partial-breast irradiation
AVE: Acidente vascular encefálico
AC: Amiloidose cardíaca
AL: Amiloidose de cadeia leve
ATTr: Amiloidose transtirretina
ACD: Artéria coronária direita
ADA: Artéria descendente anterior
AIT: Ataque isquêmico transitório
CTX: Cardiotoxicidade
DM: Diabetes melito
DAC: Doença arterial coronariana
DACIR: Doença arterial coronariana induzida por radiação
DCC: Doença cardíaca carcinoide
DCIR: Doença cardíaca induzida por radiação
DCV: Doença cardiovascular
DECH: Doença do enxerto contra o hospedeiro
DVIR: Doença valvular induzida por radiação
DVP: Doença vascular periférica
ETE: Ecocardiograma transesofágico
ETT: Ecocardiograma transtorácico
ECA: Endarterectomia de carótida
EI: Endocardite infecciosa
EMI: Espessura mediointimal
ET: Eventos trombóticos
FEVE: Fração de ejeção do ventrículo esquerdo
HAS: Hipertensão arterial sistêmica
HP: Hipertensão pulmonar
IAM: Infarto agudo do miocárdio
IC: Insuficiência cardíaca
ICFEP: Insuficiência cardíaca com fração de ejeção preservada
IMRT: Intensive-modulated radiation therapy
SUV max: Maximum standard uptake value
MN: Medicinal nuclear
MM: Mieloma múltiplo
NE: Norepinefrina
PSAP: Pressão sistólica da artéria pulmonar
QT: Quimioterapia
RT: Radioterapia
RCVE: Reserva contrátil do ventrículo esquerdo
RMC: Ressonância magnética cardíaca
SVC: Síndrome da veia cava inferior
SVCS: Síndrome da veia cava superior
ST: Síndrome de Takotsubo
STE: Speckle tracking echocardiography
SLG: Strainlongitudinal global
TC: Tomografia computadorizada
TMO: Transplante de medula óssea
TRC: Trombose relacionada ao cateter
TAC: Tumor amorfo calcificado
TCPB: Tumores cardíacos primários benignos
TCPM: Tumores cardíacos primários malignos
USV: Ultrassonografia vascular
VCI: Veia cava inferior
VJID: Veia jugular interna direita
VD: Ventrículo direito
VE: Ventrículo esquerdo
WR: Washout Rate

## ANEXO 1

### SUGESTÃO DE LAUDO DE ECOCARDIOGRAMA NA CARDIO-ONCOLOGIA

- **Identificação do paciente:**
  - Nome:
  - Registro hospitalar/Identidade pessoal:
  - Gênero:
  - Data de nascimento: / / (….. anos)
- Diagnóstico oncológico:
- Comorbidades:
- Tratamento:
  - Data do início do tratamento: / /
  - Data do término do tratamento: / /
  - Protocolo de tratamento (fármacosusados):
  - Dose cumulativa de antraciclina:mg/m^2^
  - Radioterapia torácica:Gy
  - Outros medicamentos em uso:
- Momento (timing) do exame:
  □ Exame do início do tratamento (basal)
  □ Exame intermediário (avaliação de rotina). Delta QT: ……. dias
  □ Exame por intercorrência. Qual:….
  □ Exame de término de tratamento
  □ Exame fora de tratamento (FT): ……. meses / ……. anos
    Data do último exame: / /
- Dados antropométricos:
  Peso:……. Kg Estatura:……. m SC:……. m^2^IMC:…….
- Sinais vitais:
  FC: bpm PA: X mmHg FR: ipm Temp.: °
- Condições de volemia:
  □ Hipervolemia
  □ Desidratação
- – **Medidas ecocardiográficas bidimensionais convencionais: (….)**
- **Volume VEd:mLVolume VEs:mL**
- **Massa VE:gÍndice de massa do VE:g/m2**
- **Anatomia cardíaca: (descrição sequencial)**
- **Aparelhos valvares: (descrição estrutural, mobilidade, Doppler…)**
- **Estimativa da pressão sistólica arterial pulmonar:mmHg**
- **Parâmetros de avaliaçãoda função diastólica:**
  - E/A:
  - E/e’ septal:cm/s
  - E/e’lateral:cm/s
  - S:cm/s
  - Volume AEi:mL/m^2^
  - SLG AE:
  - Outros?
- **Parâmetros de avaliação da função sistólica:**
  - FEVE (Simpson biplanar):
  - Fração de ejeção pelo método 3D:
  - *Strain* longitudinal global do VE (SLGVE):
  - Alterações na contratilidade segmentar:
  - Contratilidade global do VD (análise qualitativa):
  - FEVD (Simpson):
  - *Strain* longitudinal global do VD (SLGVD):
  - Outros?
- **Pericárdio:**
- **Conclusões:**
- **Análise comparativa funcional:**
  Queda da FEVE em comparação com o exame basal:%
  Queda do SLG em comparação com o exame basal:
- **Observações:**

## ANEXO 2

### CONSIDERAÇÕES IMPORTANTES NA AVALIAÇÃO DO LAUDO DO ECOCARDIOGRAMA NA CARDIO-ONCOLOGIA

1. A ecocardiografia deve sempre ser realizada antes de qualquer tratamento onco-hematológico para registro do estado funcional inicial do paciente e identificação de cardiopatia preexistente. Esses dados servirão como base comparativa para os exames subsequentes.
2. O tempo ideal para avaliação ecocardiográfica antes do próximo ciclo quimioterápico é usualmente de 3 semanas após a última infusão, ou o mais próximo possível da próxima.
3. Os exames intermediários são indicados de acordo com o protocolo terapêutico (potencial cardiotóxico dos quimioterápicos utilizados) ou pela estratificação do risco cardiovascular individual.
4. Atentar quanto ao estado volêmico do paciente (condições de pré e pós-carga) que poderão influenciar nos valores reais do *strain* .
5. Avaliações em vigência de intercorrências que comprometam o estado hemodinâmico do paciente servirão de informação pontual. O exame deverá ser repetido após a melhora clínica do paciente.
6. Em caso de disfunção detectada (queda da FEVE e/ou do SLG, ou alteração de função diastólica), repetir o exame após 3 semanas para confirmação da suspeita ou interpretação como condição transitória que merecerá atenção evolutiva.
7. Em situações de queda da FEVE e/ou do SLG em uso de fármacossabidamente não cardiotóxicos, considerar outras causas para a disfunção miocárdica (p. ex., infecção, DAC).
8. Obter um bom traçado de ECG para adequada quantificação do *strain* .
9. Usar janelas ecocardiográficas o mais adequadas possíveis, evitando o encurtamento da imagem que induz à supervalorização dos índices de deformação miocárdica.
10. Preferencialmente, ser um mesmo examinador para o mesmo paciente durante todo o acompanhamento.
11. Considerar o uso da mesma marca de equipamento na avaliação do mesmo paciente.
12. Não realizar SLG em pacientes com arritmia cardíaca (fibrilação atrial, taquicardia intensa e bloqueio AV).

## ANEXO 3

### SINAIS DE ALERTA DURANTE A AVALIAÇÃO DO ECOCARDIOGRAMA NA CARDIO-ONCOLOGIA:

**Table:**

Favorável	Atenção	Cuidado	Desfavorável

- FEVE normal + SLG normal + Troponina (-)
- FEVE normal + SLG normal + disfunção diastólica tipo I
  = alteração transitória? Reavaliar a situação no próximo exame eletivo.
  Alterações da contratilidade segmentar + FEVE normal + SLG normal
  = Disfunção subclínica? Alteração transitória?
  = Reavaliar a situação no próximo exame eletivo.
- FEVE nos limites inferiores da normalidade em paciente taquicárdico.
- FEVE normal + SLG <16% ou queda SLG > 15% do valor basal
  = Disfunção subclínica? Considerar medidas cardioprotetoras.
- FEVE anormal + SLG <16% ou queda SLG >15% do valor basal + troponina (+)
  = Discutir risco/benefício do esquema QT + tratamento cardiológico.
  = Repetir ecocardiograma em curto prazo.
- FEVE anormal (<40%)
  = Discutir suspensão temporária do esquema QT + tratamento cardiológico.
  = Repetir ecocardiograma em curto prazo.
- Hipertensão pulmonar com repercussão de ventrículo direito.
- Tamponamento cardíaco.

## References

[1] Santos MO. Estimate 2018: Cancer Incidence in Brazil. Revista Brasileira de Cancerologia. 2018;64(1):119-20.

[2] DeVita VT, Jr., Rosenberg SA. Two hundred years of cancer research. N Engl J Med. 2012;366(23):2207-14. 10.1056/NEJMra1204479 PMC6293471 22646510

[3] Siqueira ASE, Siqueira-Filho AG, Land MGP. Analysis of the Economic Impact of Cardiovascular Diseases in the Last Five Years in Brazil. Arq Bras Cardiol. 2017;109(1):39-46. 10.5935/abc.20170068 PMC5524474 28591251

[4] Zamorano JL, Lancellotti P, Rodriguez Munoz D, Aboyans V, Asteggiano R, Galderisi M, et al.2016 ESC Position Paper on cancer treatments and cardiovascular toxicity developed under the auspices of the ESC Committee for Practice Guidelines: The Task Force for cancer treatments and cardiovascular toxicity of the European Society of Cardiology (ESC). Eur Heart J. 2016;37(36):2768-801. 10.1093/eurheartj/ehw211 27567406

[5] Armenian S, Bhatia S. Predicting and Preventing Anthracycline-Related Cardiotoxicity. Am Soc Clin Oncol Educ Book. 2018;38:3-12. 10.1200/EDBK_100015 30231396

[6] Armenian SH, Lacchetti C, Barac A, Carver J, Constine LS, Denduluri N, et al.Prevention and Monitoring of Cardiac Dysfunction in Survivors of Adult Cancers: American Society of Clinical Oncology Clinical Practice Guideline. J Clin Oncol. 2017;35(8):893-911. 10.1200/JCO.2016.70.5400 27918725

[7] Grupo de Estudos em Insuficiencia Cardiaca da Sociedade Brasileira de C, Sociedade Brasileira de Oncologia C, Instituto do Coracao - Faculdade de Medicina da Universidade de Sao P, Instituto do Cancer do Estado de Sao Paulo - Faculdade de Medicina da Universidade de Sao P, Kalil Filho R, Hajjar LA, et al.[I Brazilian Guideline for Cardio-Oncology from Sociedade Brasileira de Cardiologia]. Arq Bras Cardiol. 2011;96(2 Suppl 1):1-52.

[8] Virani SA, Dent S, Brezden-Masley C, Clarke B, Davis MK, Jassal DS, et al.Canadian Cardiovascular Society Guidelines for Evaluation and Management of Cardiovascular Complications of Cancer Therapy. Can J Cardiol. 2016;32(7):831-41. 10.1016/j.cjca.2016.02.078 27343741

[9] Feldman AM, Lorell BH, Reis SE. Trastuzumab in the treatment of metastatic breast cancer: anticancer therapy versus cardiotoxicity. Circulation. 2000;102(3):272-4. 10.1161/01.cir.102.3.272 10899087

[10] Ewer MS, Lippman SM. Type II chemotherapy-related cardiac dysfunction: time to recognize a new entity. J Clin Oncol. 2005;23(13):2900-2. 10.1200/JCO.2005.05.827 15860848

[11] Cardinale D, Colombo A, Bacchiani G, Tedeschi I, Meroni CA, Veglia F, et al.Early detection of anthracycline cardiotoxicity and improvement with heart failure therapy. Circulation. 2015;131(22):1981-8. 10.1161/CIRCULATIONAHA.114.013777 25948538

[12] Johnson DB, Chandra S, Sosman JA. Immune Checkpoint Inhibitor Toxicity in 2018. JAMA. 2018;320(16):1702-3. 10.1001/jama.2018.13995 30286224

[13] Thavendiranathan P, Grant AD, Negishi T, Plana JC, Popovic ZB, Marwick TH. Reproducibility of echocardiographic techniques for sequential assessment of left ventricular ejection fraction and volumes: application to patients undergoing cancer chemotherapy. J Am Coll Cardiol. 2013;61(1):77-84. 10.1016/j.jacc.2012.09.035 23199515

[14] Hajjar LA, Costa IBSS, Lopes MACQ, Hoff PMG, Diz MDPE, Fonseca SMR, et al. Diretriz Brasileira de Cardio-oncologia – 2020. Arq Bras Cardiol. 2020; [online]. DOI: 10.36660/abc.20201006 PMC8452206 33295473

[15] Plana JC, Thavendiranathan P, Bucciarelli-Ducci C, Lancellotti P. Multi-Modality Imaging in the Assessment of Cardiovascular Toxicity in the Cancer Patient. JACC Cardiovasc Imaging. 2018;11(8):1173-86. 10.1016/j.jcmg.2018.06.003 30092972

[16] Tassan-Mangina S, Codorean D, Metivier M, Costa B, Himberlin C, Jouannaud C, et al.Tissue Doppler imaging and conventional echocardiography after anthracycline treatment in adults: early and late alterations of left ventricular function during a prospective study. Eur J Echocardiogr. 2006;7(2):141-6. 10.1016/j.euje.2005.04.009 15941672

[17] Pavlopoulos H, Nihoyannopoulos P. Strain and strain rate deformation parameters: from tissue Doppler to 2D speckle tracking. Int J Cardiovasc Imaging. 2008;24(5):479-91. 10.1007/s10554-007-9286-9 18074240

[18] Eidem BW. Identification of anthracycline cardiotoxicity: left ventricular ejection fraction is not enough. J Am Soc Echocardiogr. 2008;21(12):1290-2. 10.1016/j.echo.2008.10.008 19041570

[19] Force T. Introduction to cardiotoxicity review series. Circ Res. 2010;106(1):19-20. 10.1161/CIRCRESAHA.109.210724 20056942

[20] Mantovani G, Madeddu C, Cadeddu C, Dessi M, Piras A, Massa E, et al.Persistence, up to 18 months of follow-up, of epirubicin-induced myocardial dysfunction detected early by serial tissue Doppler echocardiography: correlation with inflammatory and oxidative stress markers. Oncologist. 2008;13(12):1296-305. 10.1634/theoncologist.2008-0151 19060235

[21] Thavendiranathan P, Poulin F, Lim KD, Plana JC, Woo A, Marwick TH. Use of myocardial strain imaging by echocardiography for the early detection of cardiotoxicity in patients during and after cancer chemotherapy: a systematic review. J Am Coll Cardiol. 2014;63(25 Pt A):2751-68. 10.1016/j.jacc.2014.01.073 24703918

[22] Ganame J, Claus P, Eyskens B, Uyttebroeck A, Renard M, D’Hooge J, et al.Acute cardiac functional and morphological changes after Anthracycline infusions in children. Am J Cardiol. 2007;99(7):974-7. 10.1016/j.amjcard.2006.10.063 17398195

[23] Ganame J, Claus P, Uyttebroeck A, Renard M, D’Hooge J, Bijnens B, et al.Myocardial dysfunction late after low-dose anthracycline treatment in asymptomatic pediatric patients. J Am Soc Echocardiogr. 2007;20(12):1351-8. 10.1016/j.echo.2007.04.007 17604960

[24] Sawaya H, Sebag IA, Plana JC, Januzzi JL, Ky B, Cohen V, et al.Early detection and prediction of cardiotoxicity in chemotherapy-treated patients. Am J Cardiol. 2011;107(9):1375-80. 10.1016/j.amjcard.2011.01.006 PMC3703314 21371685

[25] Tan TC, Bouras S, Sawaya H, Sebag IA, Cohen V, Picard MH, et al.Time Trends of Left Ventricular Ejection Fraction and Myocardial Deformation Indices in a Cohort of Women with Breast Cancer Treated with Anthracyclines, Taxanes, and Trastuzumab. J Am Soc Echocardiogr. 2015;28(5):509-14. 10.1016/j.echo.2015.02.001 25772019

[26] Almeida ALC, Silva VA, de Souza Filho AT, Rios VG, Lopes JR, de Afonseca SO, et al.Subclinical ventricular dysfunction detected by speckle tracking two years after use of anthracycline. Arq Bras Cardiol. 2015;104(4):274-83. 10.5935/abc.20140209 PMC4415863 25993590

[27] Piveta RB, Rodrigues ACT, Vieira MLC, Fischer CH, Afonso TR, Daminello E, et al.Early Changes in Myocardial Mechanics Detected by 3-Dimensional Speckle Tracking Echocardiography in Patients Treated With Low Doses of Anthracyclines. JACC Cardiovasc Imaging. 2018;11(11):1729-31. 10.1016/j.jcmg.2018.04.014 29909102

[28] Plana JC, Galderisi M, Barac A, Ewer MS, Ky B, Scherrer-Crosbie M, et al.Expert consensus for multimodality imaging evaluation of adult patients during and after cancer therapy: a report from the American Society of Echocardiography and the European Association of Cardiovascular Imaging. Eur Heart J Cardiovasc Imaging. 2014;15(10):1063-93. 10.1093/ehjci/jeu192 PMC4402366 25239940

[29] Liu J, Banchs J, Mousavi N, Plana JC, Scherrer-Crosbie M, Thavendiranathan P, et al.Contemporary Role of Echocardiography for Clinical Decision Making in Patients During and After Cancer Therapy. JACC Cardiovasc Imaging. 2018;11(8):1122-31. 10.1016/j.jcmg.2018.03.025 30092969

[30] Thavendiranathan P, Negishi T, Somerset E, Negishi K, Penicka M, Lemieux J, Aakhus S, Miyazaki S, Shirazi M, Galderisi M, Marwick TH, on behalf of the SUCCOUR investigators, Strain-Guided Management of Potentially Cardiotoxic Cancer Therapy, Journal of the American College of Cardiology (2020), doi: 10.1016/j.jacc.2020.11.020. 33220426

[31] Negishi K, Negishi T, Haluska BA, Hare JL, Plana JC, Marwick TH. Use of speckle strain to assess left ventricular responses to cardiotoxic chemotherapy and cardioprotection. Eur Heart J Cardiovasc Imaging. 2014;15(3):324-31. 10.1093/ehjci/jet159 24057661

[32] Mor-Avi V, Lang RM. Is echocardiography reliable for monitoring the adverse cardiac effects of chemotherapy? J Am Coll Cardiol. 2013;61(1):85-7. 10.1016/j.jacc.2012.10.006 23199517

[33] Nesser HJ, Mor-Avi V, Gorissen W, Weinert L, Steringer-Mascherbauer R, Niel J, et al.Quantification of left ventricular volumes using three-dimensional echocardiographic speckle tracking: comparison with MRI. Eur Heart J. 2009;30(13):1565-73. 10.1093/eurheartj/ehp187 19482868

[34] Armstrong GT, Plana JC, Zhang N, Srivastava D, Green DM, Ness KK, et al.Screening adult survivors of childhood cancer for cardiomyopathy: comparison of echocardiography and cardiac magnetic resonance imaging. J Clin Oncol. 2012;30(23):2876-84. 10.1200/JCO.2011.40.3584 PMC3671529 22802310

[35] Muraru D, Badano LP, Piccoli G, Gianfagna P, Del Mestre L, Ermacora D, et al.Validation of a novel automated border-detection algorithm for rapid and accurate quantitation of left ventricular volumes based on three-dimensional echocardiography. Eur J Echocardiogr. 2010;11(4):359-68. 10.1093/ejechocard/jep217 20042421

[36] Porter TR, Abdelmoneim S, Belcik JT, McCulloch ML, Mulvagh SL, Olson JJ, et al.Guidelines for the cardiac sonographer in the performance of contrast echocardiography: a focused update from the American Society of Echocardiography. J Am Soc Echocardiogr. 2014;27(8):797-810. 10.1016/j.echo.2014.05.011 25085408

[37] Peel AB, Thomas SM, Dittus K, Jones LW, Lakoski SG. Cardiorespiratory fitness in breast cancer patients: a call for normative values. J Am Heart Assoc. 2014;3(1):e000432. 10.1161/JAHA.113.000432 PMC3959685 24419734

[38] Khouri MG, Hornsby WE, Risum N, Velazquez EJ, Thomas S, Lane A, et al.Utility of 3-dimensional echocardiography, global longitudinal strain, and exercise stress echocardiography to detect cardiac dysfunction in breast cancer patients treated with doxorubicin-containing adjuvant therapy. Breast Cancer Res Treat. 2014;143(3):531-9. 10.1007/s10549-013-2818-1 PMC4521213 24390149

[39] Civelli M, Cardinale D, Martinoni A, Lamantia G, Colombo N, Colombo A, et al.Early reduction in left ventricular contractile reserve detected by dobutamine stress echo predicts high-dose chemotherapy-induced cardiac toxicity. Int J Cardiol. 2006;111(1):120-6. 10.1016/j.ijcard.2005.07.029 16242796

[40] Kirkham AA, Virani SA, Campbell KL. The utility of cardiac stress testing for detection of cardiovascular disease in breast cancer survivors: a systematic review. Int J Womens Health. 2015;7:127-40. 10.2147/IJWH.S68745 PMC4315553 25657599

[41] Eidem BW, Sapp BG, Suarez CR, Cetta F. Usefulness of the myocardial performance index for early detection of anthracycline-induced cardiotoxicity in children. Am J Cardiol. 2001;87(9):1120-2, A9. 10.1016/s0002-9149(01)01476-x 11348617

[42] Ishii M, Tsutsumi T, Himeno W, Eto G, Furui J, Hashino K, et al.Sequential evaluation of left ventricular myocardial performance in children after anthracycline therapy. Am J Cardiol. 2000;86(11):1279-81, A9. 10.1016/s0002-9149(00)01222-4 11090811

[43] Dorup I, Levitt G, Sullivan I, Sorensen K. Prospective longitudinal assessment of late anthracycline cardiotoxicity after childhood cancer: the role of diastolic function. Heart. 2004;90(10):1214-6. 10.1136/hrt.2003.027516 PMC1768494 15367528

[44] Negishi K, Negishi T, Hare JL, Haluska BA, Plana JC, Marwick TH. Independent and incremental value of deformation indices for prediction of trastuzumab-induced cardiotoxicity. J Am Soc Echocardiogr. 2013;26(5):493-8. 10.1016/j.echo.2013.02.008 23562088

[45] Tadic M, Cuspidi C, Hering D, Venneri L, Danylenko O. The influence of chemotherapy on the right ventricle: did we forget something? Clin Cardiol. 2017;40(7):437-43. 10.1002/clc.22672 PMC6490398 28191909

[46] Boczar KE, Aseyev O, Sulpher J, Johnson C, Burwash IG, Turek M, et al.Right heart function deteriorates in breast cancer patients undergoing anthracycline-based chemotherapy. Echo Res Pract. 2016;3(3):79-84. 10.1530/ERP-16-0020 PMC5045517 27457966

[47] Calleja A, Poulin F, Khorolsky C, Shariat M, Bedard PL, Amir E, et al.Right Ventricular Dysfunction in Patients Experiencing Cardiotoxicity during Breast Cancer Therapy. J Oncol. 2015;2015:609194. 10.1155/2015/609194 PMC4539180 26339242

[48] van Royen N, Jaffe CC, Krumholz HM, Johnson KM, Lynch PJ, Natale D, et al.Comparison and reproducibility of visual echocardiographic and quantitative radionuclide left ventricular ejection fractions. Am J Cardiol. 1996;77(10):843-50. 10.1016/s0002-9149(97)89179-5 8623737

[49] Nousiainen T, Jantunen E, Vanninen E, Hartikainen J. Early decline in left ventricular ejection fraction predicts doxorubicin cardiotoxicity in lymphoma patients. Br J Cancer. 2002;86(11):1697-700. 10.1038/sj.bjc.6600346 PMC2375393 12087452

[50] Angelidis G, Giamouzis G, Karagiannis G, Butler J, Tsougos I, Valotassiou V, et al.SPECT and PET in ischemic heart failure. Heart Fail Rev. 2017;22(2):243-61. 10.1007/s10741-017-9594-7 28150111

[51] Guimaraes SL, Brandao SC, Andrade LR, Maia RJ, Markman Filho B. Cardiac sympathetic hyperactivity after chemotherapy: early sign of cardiotoxicity? Arq Bras Cardiol. 2015;105(3):228-34. 10.5935/abc.20150075 PMC4592170 26176188

[52] Carrio I, Cowie MR, Yamazaki J, Udelson J, Camici PG. Cardiac sympathetic imaging with mIBG in heart failure. JACC Cardiovasc Imaging. 2010;3(1):92-100. 10.1016/j.jcmg.2009.07.014 20129538

[53] Borde C, Kand P, Basu S. Enhanced myocardial fluorodeoxyglucose uptake following Adriamycin-based therapy: Evidence of early chemotherapeutic cardiotoxicity? World J Radiol. 2012;4(5):220-3. 10.4329/wjr.v4.i5.220 PMC3386534 22761982

[54] Jingu K, Kaneta T, Nemoto K, Ichinose A, Oikawa M, Takai Y, et al.The utility of 18F-fluorodeoxyglucose positron emission tomography for early diagnosis of radiation-induced myocardial damage. Int J Radiat Oncol Biol Phys. 2006;66(3):845-51. 10.1016/j.ijrobp.2006.06.007 17011456

[55] Toubert ME, Vercellino L, Faugeron I, Lussato D, Hindie E, Bousquet G. Fatal heart failure after a 26-month combination of tyrosine kinase inhibitors in a papillary thyroid cancer. Thyroid. 2011;21(4):451-4. 10.1089/thy.2010.0270 21385075

[56] Hu JR, Florido R, Lipson EJ, Naidoo J, Ardehali R, Tocchetti CG, et al.Cardiovascular toxicities associated with immune checkpoint inhibitors. Cardiovasc Res. 2019;115(5):854-68. 10.1093/cvr/cvz026 PMC6452314 30715219

[57] Locatelli L, Cadamuro M, Spirli C, Fiorotto R, Lecchi S, Morell CM, et al.Macrophage recruitment by fibrocystin-defective biliary epithelial cells promotes portal fibrosis in congenital hepatic fibrosis. Hepatology. 2016;63(3):965-82. 10.1002/hep.28382 PMC4764460 26645994

[58] Evans JD, Gomez DR, Chang JY, Gladish GW, Erasmus JJ, Rebueno N, et al.Cardiac (1)(8)F-fluorodeoxyglucose uptake on positron emission tomography after thoracic stereotactic body radiation therapy. Radiother Oncol. 2013;109(1):82-8. 10.1016/j.radonc.2013.07.021 24016676

[59] Thavendiranathan P, Wintersperger BJ, Flamm SD, Marwick TH. Cardiac MRI in the assessment of cardiac injury and toxicity from cancer chemotherapy: a systematic review. Circ Cardiovasc Imaging. 2013;6(6):1080-91. 10.1161/CIRCIMAGING.113.000899 24254478

[60] Jordan JH, Todd RM, Vasu S, Hundley WG. Cardiovascular Magnetic Resonance in the Oncology Patient. JACC Cardiovasc Imaging. 2018;11(8):1150-72. 10.1016/j.jcmg.2018.06.004 PMC6242266 30092971

[61] Jolly MP, Jordan JH, Melendez GC, McNeal GR, D’Agostino RB, Jr., Hundley WG. Automated assessments of circumferential strain from cine CMR correlate with LVEF declines in cancer patients early after receipt of cardio-toxic chemotherapy. J Cardiovasc Magn Reson. 2017;19(1):59. 10.1186/s12968-017-0373-3 PMC5541737 28768517

[62] Ong G, Brezden-Masley C, Dhir V, Deva DP, Chan KKW, Chow CM, et al.Myocardial strain imaging by cardiac magnetic resonance for detection of subclinical myocardial dysfunction in breast cancer patients receiving trastuzumab and chemotherapy. Int J Cardiol. 2018;261:228-33. 10.1016/j.ijcard.2018.03.041 29555336

[63] Drafts BC, Twomley KM, D’Agostino R, Jr., Lawrence J, Avis N, Ellis LR, et al.Low to moderate dose anthracycline-based chemotherapy is associated with early noninvasive imaging evidence of subclinical cardiovascular disease. JACC Cardiovasc Imaging. 2013;6(8):877-85. 10.1016/j.jcmg.2012.11.017 PMC3745801 23643285

[64] Schulz-Menger J, Bluemke DA, Bremerich J, Flamm SD, Fogel MA, Friedrich MG, et al.Standardized image interpretation and post processing in cardiovascular magnetic resonance: Society for Cardiovascular Magnetic Resonance (SCMR) board of trustees task force on standardized post processing. J Cardiovasc Magn Reson. 2013;15(1):35. 10.1186/1532-429X-15-35 PMC3695769 23634753

[65] Sara L, Szarf G, Tachibana A, Shiozaki AA, Villa AV, de Oliveira AC, et al.[II Guidelines on Cardiovascular Magnetic Resonance and Computed Tomography of the Brazilian Society of Cardiology and the Brazilian College of Radiology]. Arq Bras Cardiol. 2014;103(6 Suppl 3):1-86. 10.5935/abc.2014S006 25594284

[66] Oberholzer K, Kunz RP, Dittrich M, Thelen M. [Anthracycline-induced cardiotoxicity: cardiac MRI after treatment for childhood cancer]. Rofo. 2004;176(9):1245-50. 10.1055/s-2004-813416 15346258

[67] Farhad H, Staziaki PV, Addison D, Coelho-Filho OR, Shah RV, Mitchell RN, et al.Characterization of the Changes in Cardiac Structure and Function in Mice Treated With Anthracyclines Using Serial Cardiac Magnetic Resonance Imaging. Circ Cardiovasc Imaging. 2016;9(12)e003584. 10.1161/CIRCIMAGING.115.003584 PMC5147427 27923796

[68] Mahrholdt H, Wagner A, Judd RM, Sechtem U, Kim RJ. Delayed enhancement cardiovascular magnetic resonance assessment of non-ischaemic cardiomyopathies. Eur Heart J. 2005;26(15):1461-74. 10.1093/eurheartj/ehi258 15831557

[69] de Ville de Goyet M, Brichard B, Robert A, Renard L, Veyckemans F, Vanhoutte L, et al.Prospective cardiac MRI for the analysis of biventricular function in children undergoing cancer treatments. Pediatr Blood Cancer. 2015;62(5):867-74. 10.1002/pbc.25381 25597617

[70] Fallah-Rad N, Lytwyn M, Fang T, Kirkpatrick I, Jassal DS. Delayed contrast enhancement cardiac magnetic resonance imaging in trastuzumab induced cardiomyopathy. J Cardiovasc Magn Reson. 2008;10(1):5. 10.1186/1532-429X-10-5 PMC2244612 18272009

[71] Neilan TG, Rothenberg ML, Amiri-Kordestani L, Sullivan RJ, Steingart RM, Gregory W, et al.Myocarditis Associated with Immune Checkpoint Inhibitors: An Expert Consensus on Data Gaps and a Call to Action. Oncologist. 2018;23(8):874-8. 10.1634/theoncologist.2018-0157 PMC6156187 29802220

[72] Mahmood SS, Fradley MG, Cohen JV, Nohria A, Reynolds KL, Heinzerling LM, et al.Myocarditis in Patients Treated With Immune Checkpoint Inhibitors. J Am Coll Cardiol. 2018;71(16):1755-64. 10.1016/j.jacc.2018.02.037 PMC6196725 29567210

[73] Melendez GC, Jordan JH, D’Agostino RB, Jr., Vasu S, Hamilton CA, Hundley WG. Progressive 3-Month Increase in LV Myocardial ECV After Anthracycline-Based Chemotherapy. JACC Cardiovasc Imaging. 2017;10(6):708-9. 10.1016/j.jcmg.2016.06.006 PMC7890530 27544895

[74] Ferreira de Souza T, Quinaglia ACST, Osorio Costa F, Shah R, Neilan TG, Velloso L, et al.Anthracycline Therapy Is Associated With Cardiomyocyte Atrophy and Preclinical Manifestations of Heart Disease. JACC Cardiovasc Imaging. 2018;11(8):1045-55. 10.1016/j.jcmg.2018.05.012 PMC6196358 30092965

[75] Zagrosek A, Abdel-Aty H, Boye P, Wassmuth R, Messroghli D, Utz W, et al.Cardiac magnetic resonance monitors reversible and irreversible myocardial injury in myocarditis. JACC Cardiovasc Imaging. 2009;2(2):131-8. 10.1016/j.jcmg.2008.09.014 19356545

[76] Kim RJ, Fieno DS, Parrish TB, Harris K, Chen EL, Simonetti O, et al.Relationship of MRI delayed contrast enhancement to irreversible injury, infarct age, and contractile function. Circulation. 1999;100(19):1992-2002. 10.1161/01.cir.100.19.1992 10556226

[77] Assomull RG, Prasad SK, Lyne J, Smith G, Burman ED, Khan M, et al.Cardiovascular magnetic resonance, fibrosis, and prognosis in dilated cardiomyopathy. J Am Coll Cardiol. 2006;48(10):1977-85. 10.1016/j.jacc.2006.07.049 17112987

[78] Ylanen K, Poutanen T, Savikurki-Heikkila P, Rinta-Kiikka I, Eerola A, Vettenranta K. Cardiac magnetic resonance imaging in the evaluation of the late effects of anthracyclines among long-term survivors of childhood cancer. J Am Coll Cardiol. 2013;61(14):1539-47. 10.1016/j.jacc.2013.01.019 23500246

[79] Fallah-Rad N, Walker JR, Wassef A, Lytwyn M, Bohonis S, Fang T, et al.The utility of cardiac biomarkers, tissue velocity and strain imaging, and cardiac magnetic resonance imaging in predicting early left ventricular dysfunction in patients with human epidermal growth factor receptor II-positive breast cancer treated with adjuvant trastuzumab therapy. J Am Coll Cardiol. 2011;57(22):2263-70. 10.1016/j.jacc.2010.11.063 21616287

[80] Wassmuth R, Schulz-Menger J. Late gadolinium enhancement in left ventricular dysfunction after trastuzumab. J Am Coll Cardiol. 2011;58(25):2697-8; author reply 9-700. 10.1016/j.jacc.2011.06.071 22152960

[81] Lawley C, Wainwright C, Segelov E, Lynch J, Beith J, McCrohon J. Pilot study evaluating the role of cardiac magnetic resonance imaging in monitoring adjuvant trastuzumab therapy for breast cancer. Asia Pac J Clin Oncol. 2012;8(1):95-100. 10.1111/j.1743-7563.2011.01462.x 22369450

[82] Jordan JH, Vasu S, Morgan TM, D’Agostino RB, Jr., Melendez GC, Hamilton CA, et al.Anthracycline-Associated T1 Mapping Characteristics Are Elevated Independent of the Presence of Cardiovascular Comorbidities in Cancer Survivors. Circ Cardiovasc Imaging. 2016;9(8). 10.1161/CIRCIMAGING.115.004325 PMC5508215 27502058

[83] Varki A. Trousseau’s syndrome: multiple definitions and multiple mechanisms. Blood. 2007;110(6):1723-9. 10.1182/blood-2006-10-053736 PMC1976377 17496204

[84] Khalil J, Bensaid B, Elkacemi H, Afif M, Bensaid Y, Kebdani T, et al.Venous thromboembolism in cancer patients: an underestimated major health problem. World J Surg Oncol. 2015;13:204. 10.1186/s12957-015-0592-8 PMC4486121 26092573

[85] Sallah S, Wan JY, Nguyen NP. Venous thrombosis in patients with solid tumors: determination of frequency and characteristics. Thromb Haemost. 2002;87(4):575-9. 12008937

[86] Cohen AT, Katholing A, Rietbrock S, Bamber L, Martinez C. Epidemiology of first and recurrent venous thromboembolism in patients with active cancer. A population-based cohort study. Thromb Haemost. 2017;117(1):57-65. 10.1160/TH15-08-0686 27709226

[87] Timp JF, Braekkan SK, Versteeg HH, Cannegieter SC. Epidemiology of cancer-associated venous thrombosis. Blood. 2013;122(10):1712-23. 10.1182/blood-2013-04-460121 23908465

[88] Cronin-Fenton DP, Sondergaard F, Pedersen LA, Fryzek JP, Cetin K, Acquavella J, et al.Hospitalisation for venous thromboembolism in cancer patients and the general population: a population-based cohort study in Denmark, 1997-2006. Br J Cancer. 2010;103(7):947-53. 10.1038/sj.bjc.6605883 PMC2965880 20842120

[89] Needleman L, Cronan JJ, Lilly MP, Merli GJ, Adhikari S, Hertzberg BS, et al.Ultrasound for Lower Extremity Deep Venous Thrombosis: Multidisciplinary Recommendations From the Society of Radiologists in Ultrasound Consensus Conference. Circulation. 2018;137(14):1505-15. 10.1161/CIRCULATIONAHA.117.030687 29610129

[90] McGee DC, Gould MK. Preventing complications of central venous catheterization. N Engl J Med. 2003;348(12):1123-33. 10.1056/NEJMra011883 12646670

[91] Verso M, Agnelli G. Venous thromboembolism associated with long-term use of central venous catheters in cancer patients. J Clin Oncol. 2003;21(19):3665-75. 10.1200/JCO.2003.08.008 14512399

[92] Monreal M, Raventos A, Lerma R, Ruiz J, Lafoz E, Alastrue A, et al.Pulmonary embolism in patients with upper extremity DVT associated to venous central lines--a prospective study. Thromb Haemost. 1994;72(4):548-50. 7878630

[93] Saber W, Moua T, Williams EC, Verso M, Agnelli G, Couban S, et al.Risk factors for catheter-related thrombosis (CRT) in cancer patients: a patient-level data (IPD) meta-analysis of clinical trials and prospective studies. J Thromb Haemost. 2011;9(2):312-9. 10.1111/j.1538-7836.2010.04126.x PMC4282796 21040443

[94] Eastman ME, Khorsand M, Maki DG, Williams EC, Kim K, Sondel PM, et al.Central venous device-related infection and thrombosis in patients treated with moderate dose continuous-infusion interleukin-2. Cancer. 2001;91(4):806-14. 11241250

[95] Murray J, Precious E, Alikhan R. Catheter-related thrombosis in cancer patients. Br J Haematol. 2013;162(6):748-57. 10.1111/bjh.12474 23848991

[96] Schmaier AA, Ambesh P, Campia U. Venous Thromboembolism and Cancer. Curr Cardiol Rep. 2018;20(10):89. 10.1007/s11886-018-1034-3 30128839

[97] Dellegrottaglie S, Ostenfeld E, Sanz J, Scatteia A, Perrone-Filardi P, Bossone E. Imaging the Right Heart-Pulmonary Circulation Unit: The Role of MRI and Computed Tomography. Heart Fail Clin. 2018;14(3):377-91. 10.1016/j.hfc.2018.03.004 29966635

[98] Johns CS, Kiely DG, Rajaram S, Hill C, Thomas S, Karunasaagarar K, et al.Diagnosis of Pulmonary Hypertension with Cardiac MRI: Derivation and Validation of Regression Models. Radiology. 2019;290(1):61-8. 10.1148/radiol.2018180603 PMC6314564 30351254

[99] Bane O, Shah SJ, Cuttica MJ, Collins JD, Selvaraj S, Chatterjee NR, et al.A non-invasive assessment of cardiopulmonary hemodynamics with MRI in pulmonary hypertension. Magn Reson Imaging. 2015;33(10):1224-35. 10.1016/j.mri.2015.08.005 PMC4658324 26283577

[100] Johns CS, Kiely DG, Swift AJ. Novel imaging techniques in pulmonary hypertension. Curr Opin Cardiol. 2018;33(6):587-93. 10.1097/HCO.0000000000000559 30124495

[101] Rengier F, Melzig C, Derlin T, Marra AM, Vogel-Claussen J. Advanced imaging in pulmonary hypertension: emerging techniques and applications. Int J Cardiovasc Imaging. 2019;35(8):1407-20. 10.1007/s10554-018-1448-4 30168011

[102] Cutter DJ, Darby SC, Yusuf SW. Risks of heart disease after radiotherapy. Tex Heart Inst J. 2011;38(3):257-8. PMC3113133 21720464

[103] Groarke JD, Nguyen PL, Nohria A, Ferrari R, Cheng S, Moslehi J. Cardiovascular complications of radiation therapy for thoracic malignancies: the role for non-invasive imaging for detection of cardiovascular disease. Eur Heart J. 2014;35(10):612-23. 10.1093/eurheartj/eht114 PMC3945797 23666251

[104] Lee MS, Finch W, Mahmud E. Cardiovascular complications of radiotherapy. Am J Cardiol. 2013;112(10):1688-96. 10.1016/j.amjcard.2013.07.031 24012026

[105] Darby SC, Ewertz M, McGale P, Bennet AM, Blom-Goldman U, Bronnum D, et al.Risk of ischemic heart disease in women after radiotherapy for breast cancer. N Engl J Med. 2013;368(11):987-98. 10.1056/NEJMoa1209825 23484825

[106] Nolan MT, Russell DJ, Negishi K, Marwick TH. Meta-Analysis of Association Between Mediastinal Radiotherapy and Long-Term Heart Failure. Am J Cardiol. 2016;118(11):1685-91. 10.1016/j.amjcard.2016.08.050 27692592

[107] Venkatesulu BP, Mahadevan LS, Aliru ML, Yang X, Bodd MH, Singh PK, et al.Radiation-Induced Endothelial Vascular Injury: A Review of Possible Mechanisms. JACC Basic Transl Sci. 2018;3(4):563-72. 10.1016/j.jacbts.2018.01.014 PMC6115704 30175280

[108] Gujral DM, Lloyd G, Bhattacharyya S. Radiation-induced valvular heart disease. Heart. 2016;102(4):269-76. 10.1136/heartjnl-2015-308765 26661320

[109] Jaworski C, Mariani JA, Wheeler G, Kaye DM. Cardiac complications of thoracic irradiation. J Am Coll Cardiol. 2013;61(23):2319-28. 10.1016/j.jacc.2013.01.090 23583253

[110] Cheng YJ, Nie XY, Ji CC, Lin XX, Liu LJ, Chen XM, et al.Long-Term Cardiovascular Risk After Radiotherapy in Women With Breast Cancer. J Am Heart Assoc. 2017;6(5). 10.1161/JAHA.117.005633 PMC5524103 28529208

[111] Cuomo JR, Javaheri SP, Sharma GK, Kapoor D, Berman AE, Weintraub NL. How to prevent and manage radiation-induced coronary artery disease. Heart. 2018;104(20):1647-53. 10.1136/heartjnl-2017-312123 PMC6381836 29764968

[112] Cheng SW, Ting AC, Ho P, Wu LL. Accelerated progression of carotid stenosis in patients with previous external neck irradiation. J Vasc Surg. 2004;39(2):409-15. 10.1016/j.jvs.2003.08.031 14743145

[113] Lam WW, Ho SS, Leung SF, Wong KS, Metreweli C. Cerebral blood flow measurement by color velocity imaging in radiation-induced carotid stenosis. J Ultrasound Med. 2003;22(10):1055-60. 10.7863/jum.2003.22.10.1055 14606561

[114] Moutardier V, Christophe M, Lelong B, Houvenaeghel G, Delpero JR. Iliac atherosclerotic occlusive disease complicating radiation therapy for cervix cancer: a case series. Gynecol Oncol. 2002;84(3):456-9. 10.1006/gyno.2001.6525 11855888

[115] Xu J, Cao Y. Radiation-induced carotid artery stenosis: a comprehensive review of the literature. Interv Neurol. 2014;2(4):183-92. 10.1159/000363068 PMC4188157 25337087

[116] Muzaffar K, Collins SL, Labropoulos N, Baker WH. A prospective study of the effects of irradiation on the carotid artery. Laryngoscope. 2000;110(11):1811-4. 10.1097/00005537-200011000-00007 11081590

[117] Wilbers J, Dorresteijn LD, Haast R, Hoebers FJ, Kaanders JH, Boogerd W, et al.Progression of carotid intima media thickness after radiotherapy: a long-term prospective cohort study. Radiother Oncol. 2014;113(3):359-63. 10.1016/j.radonc.2014.10.012 25466374

[118] Meeske KA, Siegel SE, Gilsanz V, Bernstein L, Nelson MB, Sposto R, et al.Premature carotid artery disease in pediatric cancer survivors treated with neck irradiation. Pediatr Blood Cancer. 2009;53(4):615-21. 10.1002/pbc.22111 PMC4412314 19533651

[119] Mavrogeni S, Dimitroulas T, Chatziioannou SN, Kitas G. The role of multimodality imaging in the evaluation of Takayasu arteritis. Semin Arthritis Rheum. 2013;42(4):401-12. 10.1016/j.semarthrit.2012.07.005 22920236

[120] Carmody BJ, Arora S, Avena R, Curry KM, Simpkins J, Cosby K, et al.Accelerated carotid artery disease after high-dose head and neck radiotherapy: is there a role for routine carotid duplex surveillance? J Vasc Surg. 1999;30(6):1045-51. 10.1016/s0741-5214(99)70042-x 10587388

[121] Tallarita T, Oderich GS, Lanzino G, Cloft H, Kallmes D, Bower TC, et al.Outcomes of carotid artery stenting versus historical surgical controls for radiation-induced carotid stenosis. J Vasc Surg. 2011;53(3):629-36 e1-5. 10.1016/j.jvs.2010.09.056 21216558

[122] Protack CD, Bakken AM, Saad WE, Illig KA, Waldman DL, Davies MG. Radiation arteritis: a contraindication to carotid stenting? J Vasc Surg. 2007;45(1):110-7. 10.1016/j.jvs.2006.08.083 17210394

[123] Fokkema M, den Hartog AG, Bots ML, van der Tweel I, Moll FL, de Borst GJ. Stenting versus surgery in patients with carotid stenosis after previous cervical radiation therapy: systematic review and meta-analysis. Stroke. 2012;43(3):793-801. 10.1161/STROKEAHA.111.633743 22207504

[124] Pham HD, Prather MG, Rush DS. Percutaneous Treatment of Superficial Femoral Artery Stenosis Secondary to Radiation Arteritis. Am Surg. 2016;82(11):1098-100. 28206937

[125] Lind PA, Pagnanelli R, Marks LB, Borges-Neto S, Hu C, Zhou SM, et al.Myocardial perfusion changes in patients irradiated for left-sided breast cancer and correlation with coronary artery distribution. Int J Radiat Oncol Biol Phys. 2003;55(4):914-20. 10.1016/s0360-3016(02)04156-1 12605969

[126] Hardenbergh PH, Munley MT, Bentel GC, Kedem R, Borges-Neto S, Hollis D, et al. Cardiac perfusion changes in patients treated for breast cancer with radiation therapy and doxorubicin: preliminary results. Int J Radiat Oncol Biol Phys. 2001;49(4):1023-8. 10.1016/s0360-3016(00)01531-5 11240243

[127] Donnellan E, Phelan D, McCarthy CP, Collier P, Desai M, Griffin B. Radiation-induced heart disease: A practical guide to diagnosis and management. Cleve Clin J Med. 2016;83(12):914-22. 10.3949/ccjm.83a.15104 27938516

[128] Adams MJ, Lipshultz SE, Schwartz C, Fajardo LF, Coen V, Constine LS. Radiation-associated cardiovascular disease: manifestations and management. Semin Radiat Oncol. 2003;13(3):346-56. 10.1016/S1053-4296(03)00026-2 12903022

[129] Lancellotti P, Nkomo VT, Badano LP, Bergler-Klein J, Bogaert J, Davin L, et al. Expert consensus for multi-modality imaging evaluation of cardiovascular complications of radiotherapy in adults: a report from the European Association os Cardiovascular Imaging and the American Society of Echocardiography. Eur Heart J Cardiovasc Imaging 2013; 14(8):721-40. 10.1093/ehjci/jet123 23847385

[130] Mulrooney DA, Nunnery SE, Armstrong GT, Ness KK, Srivastava D, Donovan FD, et al. Coronary artery disease detected by coronary computed tomography angiography in adult survivors of childhood Hodgkin lymphoma. Cancer. 2014;120(22):3536-44. 10.1002/cncr.28925 PMC4221540 25041978

[131] Girinsky T, M’Kacher R, Lessard N, Koscielny S, Elfassy E, Raoux F, et al. Prospective coronary heart disease screening in asymptomatic Hodgkin lymphoma patients using coronary computed tomography angiography: results and risk factor analysis. Int J Radiat Oncol Biol Phys. 2014;89(1):59-66. 10.1016/j.ijrobp.2014.01.021 24613809

[132] van Rosendael AR, Daniels LA, Dimitriu-Leen AC, Smit JM, van Rosendael PJ, Schalij MJ, et al. Different manifestation of irradiation induced coronary artery disease detected with coronary computed tomography compared with matched non-irradiated controls. Radiother Oncol. 2017;125(1):55-61. 10.1016/j.radonc.2017.09.008 28987749

[133] Greenwood JP, Maredia N, Younger JF, Brown JM, Nixon J, Colin C Everett CC, et al. Cardiovascular magnetic resonance and single-photon emission computed tomography for diagnosis of coronary heart disease (CE-MARC): a prospective trial. Lancet, 2012; 379(9814):453-60. 10.1016/S0140-6736(11)61335-4 PMC3273722 22196944

[134] Schelbert EB, Cao JJ, Sigurdsson S, Aspelund T, Kellman P, Aletras AH, et al. Prevalence and prognosis of unrecognized myocardial infarction determined by cardiac magnetic resonance in older adults. JAMA 2012; 308(9): 890-6. 10.1001/2012.jama.11089 PMC4137910 22948699

[135] Burke A, Tavora F. The 2015 WHO Classification of Tumors of the Heart and Pericardium. J Thorac Oncol. 2016;11(4):441-52. 10.1016/j.jtho.2015.11.009 26725181

[136] Mankad R, Herrmann J. Cardiac tumors: echo assessment. Echo Res Pract. 2016;3(4):R65-R77. 10.1530/ERP-16-0035 PMC5292983 27600455

[137] Strachinaru M, Damry N, Duttmann R, Wauthy P, Catez E, Lutea M, et al.Ultrasound Contrast Quantification for the Diagnosis of Intracardiac Masses. JACC Cardiovasc Imaging. 2016;9(6):747-50. 10.1016/j.jcmg.2015.06.025 27085435

[138] Tang QY, Guo LD, Wang WX, Zhou W, Liu YN, Liu HY, et al.Usefulness of contrast perfusion echocardiography for differential diagnosis of cardiac masses. Ultrasound Med Biol. 2015;41(9):2382-90. 10.1016/j.ultrasmedbio.2015.05.010 26087885

[139] Ganame J, D’Hooge J, Mertens L. Different deformation patterns in intracardiac tumors. Eur J Echocardiogr. 2005;6(6):461-4. 10.1016/j.euje.2005.02.006 16293533

[140] Zaragoza-Macias E, Chen MA, Gill EA. Real time three-dimensional echocardiography evaluation of intracardiac masses. Echocardiography. 2012;29(2):207-19. 10.1111/j.1540-8175.2011.01627.x 22283202

[141] Singu T, Inatomi Y, Yonehara T, Ando Y. Calcified Amorphous Tumor Causing Shower Embolism to the Brain: A Case Report with Serial Echocardiographic and Neuroradiologic Images and a Review of the Literature. J Stroke Cerebrovasc Dis. 2017;26(5):e85-e9. 10.1016/j.jstrokecerebrovasdis.2017.02.019 28318955

[142] Tzani A, Doulamis IP, Mylonas KS, Avgerinos DV, Nasioudis D. Cardiac Tumors in Pediatric Patients: A Systematic Review. World J Pediatr Congenit Heart Surg. 2017;8(5):624-32. 10.1177/2150135117723904 28901236

[143] Jain S, Maleszewski JJ, Stephenson CR, Klarich KW. Current diagnosis and management of cardiac myxomas. Expert Rev Cardiovasc Ther. 2015;13(4):369-75. 10.1586/14779072.2015.1024108 25797902

[144] Gowda RM, Khan IA, Nair CK, Mehta NJ, Vasavada BC, Sacchi TJ. Cardiac papillary fibroelastoma: a comprehensive analysis of 725 cases. Am Heart J. 2003;146(3):404-10. 10.1016/S0002-8703(03)00249-7 12947356

[145] Tamin SS, Maleszewski JJ, Scott CG, Khan SK, Edwards WD, Bruce CJ, et al.Prognostic and Bioepidemiologic Implications of Papillary Fibroelastomas. J Am Coll Cardiol. 2015;65(22):2420-9. 10.1016/j.jacc.2015.03.569 26046736

[146] Shen Q, Shen J, Qiao Z, Yao Q, Huang G, Hu X. Cardiac rhabdomyomas associated with tuberous sclerosis complex in children. From presentation to outcome. Herz. 2015;40(4):675-8. 10.1007/s00059-014-4078-1 24609800

[147] Tao TY, Yahyavi-Firouz-Abadi N, Singh GK, Bhalla S. Pediatric cardiac tumors: clinical and imaging features. Radiographics. 2014;34(4):1031-46. 10.1148/rg.344135163 25019440

[148] Capotosto L, Elena G, Massoni F, De Sio S, Carnevale A, Ricci S, et al.Cardiac Tumors: Echocardiographic Diagnosis and Forensic Correlations. Am J Forensic Med Pathol. 2016;37(4):306-16. 10.1097/PAF.0000000000000271 27617419

[149] Cetrano E, Polito A, Carotti A. Primitive intrapericardial teratoma associated with yolk sac tumour. Cardiol Young. 2015;25(1):158-60. 10.1017/S1047951113002187 24447774

[150] Li W, Teng P, Xu H, Ma L, Ni Y. Cardiac Hemangioma: A Comprehensive Analysis of 200 Cases. Ann Thorac Surg. 2015;99(6):2246-52. 10.1016/j.athoracsur.2015.02.064 25921258

[151] Semionov A, Sayegh K. Multimodality imaging of a cardiac paraganglioma. Radiol Case Rep. 2016;11(4):277-81. 10.1016/j.radcr.2016.08.002 PMC5128366 27920843

[152] Almobarak AA, AlShammari A, Alhomoudi RI, Eshaq AM, Algain SM, Jensen EC, et al.Benign Pericardial Schwannoma: Case Report and Summary of Previously Reported Cases. Am J Case Rep. 2018;19:90-4. 10.12659/AJCR.907408 PMC5789752 29362352

[153] Oliveira GH, Al-Kindi SG, Hoimes C, Park SJ. Characteristics and Survival of Malignant Cardiac Tumors: A 40-Year Analysis of >500 Patients. Circulation. 2015;132(25):2395-402. 10.1161/CIRCULATIONAHA.115.016418 26467256

[154] Randhawa JS, Budd GT, Randhawa M, Ahluwalia M, Jia X, Daw H, et al.Primary Cardiac Sarcoma: 25-Year Cleveland Clinic Experience. Am J Clin Oncol. 2016;39(6):593-9. 10.1097/COC.0000000000000106 25036471

[155] Kupsky DF, Newman DB, Kumar G, Maleszewski JJ, Edwards WD, Klarich KW. Echocardiographic Features of Cardiac Angiosarcomas: The Mayo Clinic Experience (1976-2013). Echocardiography. 2016;33(2):186-92. 10.1111/echo.13060 26460068

[156] Valles-Torres J, Izquierdo-Villarroya MB, Vallejo-Gil JM, Casado-Dominguez JM, Roche Latasa AB, Auquilla-Clavijo P. Cardiac Undifferentiated Pleomorphic Sarcoma Mimicking Left Atrial Myxoma. J Cardiothorac Vasc Anesth. 2019;33(2):493-6. 10.1053/j.jvca.2018.02.010 29551281

[157] Jang JJ, Danik S, Goldman M. Primary cardiac lymphoma: diagnosis and treatment guided by transesophageal echocardiogram perfusion imaging. J Am Soc Echocardiogr. 2006;19(8):1073 e7-9. 10.1016/j.echo.2006.04.006 16880110

[158] Restrepo CS, Vargas D, Ocazionez D, Martinez-Jimenez S, Betancourt Cuellar SL, Gutierrez FR. Primary pericardial tumors. Radiographics. 2013;33(6):1613-30. 10.1148/rg.336135512 24108554

[159] Hudzik B, Miszalski-Jamka K, Glowacki J, Lekston A, Gierlotka M, Zembala M, et al.Malignant tumors of the heart. Cancer Epidemiol. 2015;39(5):665-72. 10.1016/j.canep.2015.07.007 26239627

[160] Bussani R, De-Giorgio F, Abbate A, Silvestri F. Cardiac metastases. J Clin Pathol. 2007;60(1):27-34. 10.1136/jcp.2005.035105 PMC1860601 17098886

[161] Burazor I, Aviel-Ronen S, Imazio M, Goitein O, Perelman M, Shelestovich N, et al.Metastatic cardiac tumors: from clinical presentation through diagnosis to treatment. BMC Cancer. 2018;18(1):202. 10.1186/s12885-018-4070-x PMC5819646 29463229

[162] Lestuzzi C, De Paoli A, Baresic T, Miolo G, Buonadonna A. Malignant cardiac tumors: diagnosis and treatment. Future Cardiol. 2015;11(4):485-500. 10.2217/fca.15.10 26235817

[163] Mousavi N, Cheezum MK, Aghayev A, Padera R, Vita T, Steigner M, et al.Assessment of Cardiac Masses by Cardiac Magnetic Resonance Imaging: Histological Correlation and Clinical Outcomes. J Am Heart Assoc. 2019;8(1):e007829. 10.1161/JAHA.117.007829 PMC6405700 30616453

[164] Pazos-Lopez P, Pozo E, Siqueira ME, Garcia-Lunar I, Cham M, Jacobi A, et al.Value of CMR for the differential diagnosis of cardiac masses. JACC Cardiovasc Imaging. 2014;7(9):896-905. 10.1016/j.jcmg.2014.05.009 25129516

[165] Kassi M, Polsani V, Schutt RC, Wong S, Nabi F, Reardon MJ, et al.Differentiating benign from malignant cardiac tumors with cardiac magnetic resonance imaging. J Thorac Cardiovasc Surg. 2019;157(5):1912-22 e2. 10.1016/j.jtcvs.2018.09.057 30551963

[166] Colin GC, Dymarkowski S, Gerber B, Michoux N, Bogaert J. Cardiac myxoma imaging features and tissue characteristics at cardiovascular magnetic resonance. Int J Cardiol. 2016;202:950-1. 10.1016/j.ijcard.2015.10.111 26493410

[167] O’Donnell DH, Abbara S, Chaithiraphan V, Yared K, Killeen RP, Cury RC, et al.Cardiac tumors: optimal cardiac MR sequences and spectrum of imaging appearances. AJR Am J Roentgenol. 2009;193(2):377-87. 10.2214/AJR.08.1895 19620434

[168] Sparrow PJ, Kurian JB, Jones TR, Sivananthan MU. MR imaging of cardiac tumors. Radiographics. 2005;25(5):1255-76. 10.1148/rg.255045721 16160110

[169] Braggion-Santos MF, Koenigkam-Santos M, Teixeira SR, Volpe GJ, Trad HS, Schmidt A. Magnetic resonance imaging evaluation of cardiac masses. Arq Bras Cardiol. 2013;101(3):263-72. 10.5935/abc.20130150 PMC4032307 23887734

[170] Esposito A, De Cobelli F, Ironi G, Marra P, Canu T, Mellone R, et al.CMR in the assessment of cardiac masses: primary malignant tumors. JACC Cardiovasc Imaging. 2014;7(10):1057-61. 10.1016/j.jcmg.2014.08.002 25323167

[171] Bhattacharyya S, Khattar RS, Gujral DM, Senior R. Cardiac tumors: the role of cardiovascular imaging. Expert Rev Cardiovasc Ther. 2014;12(1):37-43. 10.1586/14779072.2014.872031 24345096

[172] Rahbar K, Seifarth H, Schafers M, Stegger L, Hoffmeier A, Spieker T, et al.Differentiation of malignant and benign cardiac tumors using 18F-FDG PET/CT. J Nucl Med. 2012;53(6):856-63. 10.2967/jnumed.111.095364 22577239

[173] Bhattacharyya S, Toumpanakis C, Burke M, Taylor AM, Caplin ME, Davar J. Features of carcinoid heart disease identified by 2- and 3-dimensional echocardiography and cardiac MRI. Circ Cardiovasc Imaging. 2010;3(1):103-11. 10.1161/CIRCIMAGING.109.886846 19920029

[174] Patel C, Mathur M, Escarcega RO, Bove AA. Carcinoid heart disease: current understanding and future directions. Am Heart J. 2014;167(6):789-95. 10.1016/j.ahj.2014.03.018 24890526

[175] Davar J, Connolly HM, Caplin ME, Pavel M, Zacks J, Bhattacharyya S, et al.Diagnosing and Managing Carcinoid Heart Disease in Patients With Neuroendocrine Tumors: An Expert Statement. J Am Coll Cardiol. 2017;69(10):1288-304. 10.1016/j.jacc.2016.12.030 28279296

[176] Dobson R, Burgess MI, Valle JW, Pritchard DM, Vora J, Wong C, et al.Serial surveillance of carcinoid heart disease: factors associated with echocardiographic progression and mortality. Br J Cancer. 2014;111(9):1703-9. 10.1038/bjc.2014.468 PMC4453728 25211656

[177] Westberg G, Wangberg B, Ahlman H, Bergh CH, Beckman-Suurkula M, Caidahl K. Prediction of prognosis by echocardiography in patients with midgut carcinoid syndrome. Br J Surg. 2001;88(6):865-72. 10.1046/j.0007-1323.2001.01798.x 11412260

[178] Dobson R, Cuthbertson DJ, Jones J, Valle JW, Keevil B, Chadwick C, et al.Determination of the optimal echocardiographic scoring system to quantify carcinoid heart disease. Neuroendocrinology. 2014;99(2):85-93. 10.1159/000360767 24603343

[179] Mesquita ET, Jorge AJL, Souza CVJ, Andrade TR. Cardiac Amyloidosis and its New Clinical Phenotype: Heart Failure with Preserved Ejection Fraction. Arq Bras Cardiol. 2017;109(1):71-80. 10.5935/abc.20170079 PMC5524478 28678923

[180] Sperry BW, Reyes BA, Ikram A, Donnelly JP, Phelan D, Jaber WA, et al.Tenosynovial and Cardiac Amyloidosis in Patients Undergoing Carpal Tunnel Release. J Am Coll Cardiol. 2018;72(17):2040-50. 10.1016/j.jacc.2018.07.092 30336828

[181] Hasserjian RP, Goodman HJ, Lachmann HJ, Muzikansky A, Hawkins PN. Bone marrow findings correlate with clinical outcome in systemic AL amyloidosis patients. Histopathology. 2007;50(5):567-73. 10.1111/j.1365-2559.2007.02658.x 17394492

[182] Merlini G, Bellotti V. Molecular mechanisms of amyloidosis. N Engl J Med. 2003;349(6):583-96. 10.1056/NEJMra023144 12904524

[183] Agha AM, Parwani P, Guha A, Durand JB, Iliescu CA, Hassan S, et al.Role of cardiovascular imaging for the diagnosis and prognosis of cardiac amyloidosis. Open Heart. 2018;5(2):e000881. 10.1136/openhrt-2018-000881 PMC6173267 30305910

[184] Aljaroudi WA, Desai MY, Tang WH, Phelan D, Cerqueira MD, Jaber WA. Role of imaging in the diagnosis and management of patients with cardiac amyloidosis: state of the art review and focus on emerging nuclear techniques. J Nucl Cardiol. 2014;21(2):271-83. 10.1007/s12350-013-9800-5 24347127

[185] Falk RH, Alexander KM, Liao R, Dorbala S. AL (Light-Chain) Cardiac Amyloidosis: A Review of Diagnosis and Therapy. J Am Coll Cardiol. 2016;68(12):1323-41. 10.1016/j.jacc.2016.06.053 27634125

[186] Mohty D, Damy T, Cosnay P, Echahidi N, Casset-Senon D, Virot P, et al.Cardiac amyloidosis: updates in diagnosis and management. Arch Cardiovasc Dis. 2013;106(10):528-40. 10.1016/j.acvd.2013.06.051 24070600

[187] Cueto-Garcia L, Reeder GS, Kyle RA, Wood DL, Seward JB, Naessens J, et al.Echocardiographic findings in systemic amyloidosis: spectrum of cardiac involvement and relation to survival. J Am Coll Cardiol. 1985;6(4):737-43. 10.1016/s0735-1097(85)80475-7 4031287

[188] Pagourelias ED, Mirea O, Duchenne J, Van Cleemput J, Delforge M, Bogaert J, et al.Echo Parameters for Differential Diagnosis in Cardiac Amyloidosis: A Head-to-Head Comparison of Deformation and Nondeformation Parameters. Circ Cardiovasc Imaging. 2017;10(3):e005588. 10.1161/CIRCIMAGING.116.005588 28298286

[189] Barros-Gomes S, Williams B, Nhola LF, Grogan M, Maalouf JF, Dispenzieri A, et al.Prognosis of Light Chain Amyloidosis With Preserved LVEF: Added Value of 2D Speckle-Tracking Echocardiography to the Current Prognostic Staging System. JACC Cardiovasc Imaging. 2017;10(4):398-407. 10.1016/j.jcmg.2016.04.008 27639764

[190] Phelan D, Collier P, Thavendiranathan P, Popovic ZB, Hanna M, Plana JC, et al.Relative apical sparing of longitudinal strain using two-dimensional speckle-tracking echocardiography is both sensitive and specific for the diagnosis of cardiac amyloidosis. Heart. 2012;98(19):1442-8. 10.1136/heartjnl-2012-302353 22865865

[191] Senapati A, Sperry BW, Grodin JL, Kusunose K, Thavendiranathan P, Jaber W, et al.Prognostic implication of relative regional strain ratio in cardiac amyloidosis. Heart. 2016;102(10):748-54. 10.1136/heartjnl-2015-308657 26830665

[192] Pun SC, Landau HJ, Riedel ER, Jordan J, Yu AF, Hassoun H, et al.Prognostic and Added Value of Two-Dimensional Global Longitudinal Strain for Prediction of Survival in Patients with Light Chain Amyloidosis Undergoing Autologous Hematopoietic Cell Transplantation. J Am Soc Echocardiogr. 2018;31(1):64-70. 10.1016/j.echo.2017.08.017 PMC5985664 29111123

[193] Liu D, Hu K, Stork S, Herrmann S, Kramer B, Cikes M, et al.Predictive value of assessing diastolic strain rate on survival in cardiac amyloidosis patients with preserved ejection fraction. PLoS One. 2014;9(12):e115910. 10.1371/journal.pone.0115910 PMC4277448 25542015

[194] Milani P, Dispenzieri A, Scott CG, Gertz MA, Perlini S, Mussinelli R, et al.Independent Prognostic Value of Stroke Volume Index in Patients With Immunoglobulin Light Chain Amyloidosis. Circ Cardiovasc Imaging. 2018;11(5):e006588. 10.1161/CIRCIMAGING.117.006588 PMC5963522 29752392

[195] Feng D, Syed IS, Martinez M, Oh JK, Jaffe AS, Grogan M, et al.Intracardiac thrombosis and anticoagulation therapy in cardiac amyloidosis. Circulation. 2009;119(18):2490-7. 10.1161/CIRCULATIONAHA.108.785014 19414641

[196] Dubrey S, Pollak A, Skinner M, Falk RH. Atrial thrombi occurring during sinus rhythm in cardiac amyloidosis: evidence for atrial electromechanical dissociation. Br Heart J. 1995;74(5):541-4. 10.1136/hrt.74.5.541 PMC484078 8562243

[197] Tuzovic M, Yang EH, Baas AS, Depasquale EC, Deng MC, Cruz D, et al.Cardiac Amyloidosis: Diagnosis and Treatment Strategies. Curr Oncol Rep. 2017;19(7):46. 10.1007/s11912-017-0607-4 28528458

[198] Syed IS, Glockner JF, Feng D, Araoz PA, Martinez MW, Edwards WD, et al.Role of cardiac magnetic resonance imaging in the detection of cardiac amyloidosis. JACC Cardiovasc Imaging. 2010;3(2):155-64. 10.1016/j.jcmg.2009.09.023 20159642

[199] Austin BA, Tang WH, Rodriguez ER, Tan C, Flamm SD, Taylor DO, et al.Delayed hyper-enhancement magnetic resonance imaging provides incremental diagnostic and prognostic utility in suspected cardiac amyloidosis. JACC Cardiovasc Imaging. 2009;2(12):1369-77. 10.1016/j.jcmg.2009.08.008 20083070

[200] Dungu JN, Valencia O, Pinney JH, Gibbs SD, Rowczenio D, Gilbertson JA, et al.CMR-based differentiation of AL and ATTR cardiac amyloidosis. JACC Cardiovasc Imaging. 2014;7(2):133-42. 10.1016/j.jcmg.2013.08.015 24412186

[201] Karamitsos TD, Piechnik SK, Banypersad SM, Fontana M, Ntusi NB, Ferreira VM, et al.Noncontrast T1 mapping for the diagnosis of cardiac amyloidosis. JACC Cardiovasc Imaging. 2013;6(4):488-97. 10.1016/j.jcmg.2012.11.013 23498672

[202] Banypersad SM, Fontana M, Maestrini V, Sado DM, Captur G, Petrie A, et al.T1 mapping and survival in systemic light-chain amyloidosis. Eur Heart J. 2015;36(4):244-51. 10.1093/eurheartj/ehu444 PMC4301598 25411195

[203] Amyloidosis Support Groups - HOW FAR ARE YOU FROM TREATMENT AND SUPPORT? [Acessado em 04/07/2020]. Disponível em: https://www.amyloidosissupport.org/. 2020.

[204] Gillmore JD, Maurer MS, Falk RH, Merlini G, Damy T, Dispenzieri A, et al.Nonbiopsy Diagnosis of Cardiac Transthyretin Amyloidosis. Circulation. 2016;133(24):2404-12. 10.1161/CIRCULATIONAHA.116.021612 27143678

[205] Merlini G, Dispenzieri A, Sanchorawala V, Schonland SO, Palladini G, Hawkins PN, et al.Systemic immunoglobulin light chain amyloidosis. Nat Rev Dis Primers. 2018;4(1):38. 10.1038/s41572-018-0034-3 30361521

[206] Milani P, Merlini G, Palladini G. Light Chain Amyloidosis. Mediterr J Hematol Infect Dis. 2018;10(1):e2018022. 10.4084/MJHID.2018.022 PMC5841939 29531659

[207] Ribeiro-Silva C, Gilberto S, Gomes RA, Mateus E, Monteiro E, Barroso E, et al.The relative amounts of plasma transthyretin forms in familial transthyretin amyloidosis: a quantitative analysis by Fourier transform ion-cyclotron resonance mass spectrometry. Amyloid. 2011;18(4):191-9. 10.3109/13506129.2011.614295 22080762

[208] Burgdorf C, Kurowski V, Bonnemeier H, Schunkert H, Radke PW. Long-term prognosis of the transient left ventricular dysfunction syndrome (Tako-Tsubo cardiomyopathy): focus on malignancies. Eur J Heart Fail. 2008;10(10):1015-9. 10.1016/j.ejheart.2008.07.008 18692439

[209] Giza DE, Lopez-Mattei J, Vejpongsa P, Munoz E, Iliescu G, Kitkungvan D, et al.Stress-Induced Cardiomyopathy in Cancer Patients. Am J Cardiol. 2017;120(12):2284-8. 10.1016/j.amjcard.2017.09.009 29096885

[210] Heggemann F, Hamm K, Kaelsch T, Sueselbeck T, Papavassiliu T, Borggrefe M, et al.Global and regional myocardial function quantification in Takotsubo cardiomyopathy in comparison to acute anterior myocardial infarction using two-dimensional (2D) strain echocardiography. Echocardiography. 2011;28(7):715-9. 10.1111/j.1540-8175.2011.01430.x 21545518

[211] Frustaci A, Loperfido F, Gentiloni N, Caldarulo M, Morgante E, Russo MA. Catecholamine-induced cardiomyopathy in multiple endocrine neoplasia. A histologic, ultrastructural, and biochemical study. Chest. 1991;99(2):382-5. 10.1378/chest.99.2.382 1671211

[212] Gilreath JA, Stenehjem DD, Rodgers GM. Diagnosis and treatment of cancer-related anemia. Am J Hematol. 2014;89(2):203-12. 10.1002/ajh.23628 24532336

[213] Lutz K, von Komorowski G, Durken M, Engelhardt R, Dinter DJ. Myocardial iron overload in transfusion-dependent pediatric patients with acute leukemia. Pediatr Blood Cancer. 2008;51(5):691-3. 10.1002/pbc.21663 18623223

[214] Wasserman AJ, Richardson DW, Baird CL, Wyso EM. Cardiac hemochromatosis simulating constrictive pericarditis. Am J Med. 1962;32:316-23. 10.1016/0002-9343(62)90299-1 14005128

[215] Di Odoardo LAF, Giuditta M, Cassinerio E, Roghi A, Pedrotti P, Vicenzi M, et al.Myocardial deformation in iron overload cardiomyopathy: speckle tracking imaging in a beta-thalassemia major population. Intern Emerg Med. 2017;12(6):799-809. 10.1007/s11739-017-1670-4 28456904

[216] Li SJ, Hwang YY, Ha SY, Chan GC, Mok AS, Wong SJ, et al.Role of Three-Dimensional Speckle Tracking Echocardiography in the Quantification of Myocardial Iron Overload in Patients with Beta-Thalassemia Major. Echocardiography. 2016;33(9):1361-7. 10.1111/echo.13266 27158922

[217] Schempp A, Lee J, Kearney S, Mulrooney DA, Smith AR. Iron Overload in Survivors of Childhood Cancer. J Pediatr Hematol Oncol. 2016;38(1):27-31. 10.1097/MPH.0000000000000444 26422286

[218] Roy NB, Myerson S, Schuh AH, Bignell P, Patel R, Wainscoat JS, et al.Cardiac iron overload in transfusion-dependent patients with myelodysplastic syndromes. Br J Haematol. 2011;154(4):521-4. 10.1111/j.1365-2141.2011.08749.x 21689086

[219] El Haddad D, Iliescu C, Yusuf SW, William WN, Jr., Khair TH, Song J, et al.Outcomes of Cancer Patients Undergoing Percutaneous Pericardiocentesis for Pericardial Effusion. J Am Coll Cardiol. 2015;66(10):1119-28. 10.1016/j.jacc.2015.06.1332 PMC4560839 26337990

[220] Lestuzzi C, Berretta M, Tomkowski W. 2015 update on the diagnosis and management of neoplastic pericardial disease. Expert Rev Cardiovasc Ther. 2015;13(4):377-89. 10.1586/14779072.2015.1025754 25797903

[221] Yared K, Baggish AL, Picard MH, Hoffmann U, Hung J. Multimodality imaging of pericardial diseases. JACC Cardiovasc Imaging. 2010;3(6):650-60. 10.1016/j.jcmg.2010.04.009 20541720

[222] Sogaard KK, Sorensen HT, Smeeth L, Bhaskaran K. Acute Pericarditis and Cancer Risk: A Matched Cohort Study Using Linked UK Primary and Secondary Care Data. J Am Heart Assoc. 2018;7(16):e009428. 10.1161/JAHA.118.009428 PMC6201410 30369322

[223] Kong L, Li Z, Wang J, Lv X. Echocardiographic characteristics of primary malignant pericardial mesothelioma and outcomes analysis: a retrospective study. Cardiovasc Ultrasound. 2018;16(1):7. 10.1186/s12947-018-0125-z PMC5922299 29695235

[224] Virk SA, Chandrakumar D, Villanueva C, Wolfenden H, Liou K, Cao C. Systematic review of percutaneous interventions for malignant pericardial effusion. Heart. 2015;101(20):1619-26. 10.1136/heartjnl-2015-307907 26180077

[225] Brasil. Ministério da SaúdeInstituto Nacional de Cancer. Estimativa 2020. [Acessado em 05/07/2020]. Disponível em: https://www.inca.gov.br. 2020.

[226] Chow EJ, Leger KJ, Bhatt NS, Mulrooney DA, Ross CJ, Aggarwal S, et al.Paediatric cardio-oncology: epidemiology, screening, prevention, and treatment. Cardiovasc Res. 2019;115(5):922-34. 10.1093/cvr/cvz031 PMC6452306 30768157

[227] Armenian SH, Hudson MM, Mulder RL, Chen MH, Constine LS, Dwyer M, et al.Recommendations for cardiomyopathy surveillance for survivors of childhood cancer: a report from the International Late Effects of Childhood Cancer Guideline Harmonization Group. Lancet Oncol. 2015;16(3):e123-36. 10.1016/S1470-2045(14)70409-7 PMC4485458 25752563

[228] Lipshultz SE, Diamond MB, Franco VI, Aggarwal S, Leger K, Santos MV, et al.Managing chemotherapy-related cardiotoxicity in survivors of childhood cancers. Paediatr Drugs. 2014;16(5):373-89. 10.1007/s40272-014-0085-1 PMC4417358 25134924

[229] Yeh ET, Bickford CL. Cardiovascular complications of cancer therapy: incidence, pathogenesis, diagnosis, and management. J Am Coll Cardiol. 2009;53(24):2231-47. 10.1016/j.jacc.2009.02.050 19520246

[230] Vejpongsa P, Yeh ET. Topoisomerase 2beta: a promising molecular target for primary prevention of anthracycline-induced cardiotoxicity. Clin Pharmacol Ther. 2014;95(1):45-52. 10.1038/clpt.2013.201 24091715

[231] Levitt GA, Dorup I, Sorensen K, Sullivan I. Does anthracycline administration by infusion in children affect late cardiotoxicity? Br J Haematol. 2004;124(4):463-8. 10.1111/j.1365-2141.2004.04803.x 14984495

[232] Manolis AA, Manolis TA, Mikhailidis DP, Manolis AS. Cardiovascular safety of oncologic agents: A double-edged sword even in the era of targeted therapies - part 1. Expert Opin Drug Saf. 2018;17(9):875-92. 10.1080/14740338.2018.1513488 30126304

[233] Chow EJ, Antal Z, Constine LS, Gardner R, Wallace WH, Weil BR, et al.New Agents, Emerging Late Effects, and the Development of Precision Survivorship. J Clin Oncol. 2018;36(21):2231-40. 10.1200/JCO.2017.76.4647 PMC6053298 29874142

[234] Mulrooney DA, Yeazel MW, Kawashima T, Mertens AC, Mitby P, Stovall M, et al.Cardiac outcomes in a cohort of adult survivors of childhood and adolescent cancer: retrospective analysis of the Childhood Cancer Survivor Study cohort. BMJ. 2009;339:b4606. 10.1136/bmj.b4606 PMC3266843 19996459

[235] Vandecruys E, Mondelaers V, De Wolf D, Benoit Y, Suys B. Late cardiotoxicity after low dose of anthracycline therapy for acute lymphoblastic leukemia in childhood. J Cancer Surviv. 2012;6(1):95-101. 10.1007/s11764-011-0186-6 PMC3279635 21630046

[236] Landy DC, Miller TL, Lipsitz SR, Lopez-Mitnik G, Hinkle AS, Constine LS, et al.Cranial irradiation as an additional risk factor for anthracycline cardiotoxicity in childhood cancer survivors: an analysis from the cardiac risk factors in childhood cancer survivors study. Pediatr Cardiol. 2013;34(4):826-34. 10.1007/s00246-012-0539-6 PMC3594453 23080542

[237] Aminkeng F, Bhavsar AP, Visscher H, Rassekh SR, Li Y, Lee JW, et al.A coding variant in RARG confers susceptibility to anthracycline-induced cardiotoxicity in childhood cancer. Nat Genet. 2015;47(9):1079-84. 10.1038/ng.3374 PMC4552570 26237429

[238] Hefti E, Blanco JG. Anthracycline-Related Cardiotoxicity in Patients with Acute Myeloid Leukemia and Down Syndrome: A Literature Review. Cardiovasc Toxicol. 2016;16(1):5-13. 10.1007/s12012-015-9307-1 PMC4514565 25616318

[239] Pignatelli RH, Ghazi P, Reddy SC, Thompson P, Cui Q, Castro J, et al.Abnormal Myocardial Strain Indices in Children Receiving Anthracycline Chemotherapy. Pediatr Cardiol. 2015;36(8):1610-6. 10.1007/s00246-015-1203-8 26049414

[240] Colan SD, Shirali G, Margossian R, Gallagher D, Altmann K, Canter C, et al.The ventricular volume variability study of the Pediatric Heart Network: study design and impact of beat averaging and variable type on the reproducibility of echocardiographic measurements in children with chronic dilated cardiomyopathy. J Am Soc Echocardiogr. 2012;25(8):842-54 e6. 10.1016/j.echo.2012.05.004 PMC3568492 22677278

[241] Roca-Bielsa I, Vlajkovic M. Pediatric nuclear medicine and pediatric radiology: modalities, image quality, dosimetry and correlative imaging: new strategies. Pediatr Radiol. 2013;43(4):391-2. 10.1007/s00247-013-2656-7 23525765

[242] Corbett JR, Akinboboye OO, Bacharach SL, Borer JS, Botvinick EH, DePuey EG, et al.Equilibrium radionuclide angiocardiography. J Nucl Cardiol. 2006;13(6):e56-79. 10.1016/j.nuclcard.2006.08.007 17174797

[243] Bybee KA, Prasad A. Stress-related cardiomyopathy syndromes. Circulation. 2008;118(4):397-409. 10.1161/CIRCULATIONAHA.106.677625 18645066

[244] Yusuf SW, Sami S, Daher IN. Radiation-induced heart disease: a clinical update. Cardiol Res Pract. 2011;2011:317659. 10.4061/2011/317659 PMC3051159 21403872

[245] Heidenreich PA, Schnittger I, Strauss HW, Vagelos RH, Lee BK, Mariscal CS, et al.Screening for coronary artery disease after mediastinal irradiation for Hodgkin’s disease. J Clin Oncol. 2007;25(1):43-9. 10.1200/JCO.2006.07.0805 17194904

[246] Shankar SM, Marina N, Hudson MM, Hodgson DC, Adams MJ, Landier W, et al.Monitoring for cardiovascular disease in survivors of childhood cancer: report from the Cardiovascular Disease Task Force of the Children’s Oncology Group. Pediatrics. 2008;121(2):e387-96. 10.1542/peds.2007-0575 18187811

[247] van Dalen EC, van der Pal HJ, van den Bos C, Kok WE, Caron HN, Kremer LC. Clinical heart failure during pregnancy and delivery in a cohort of female childhood cancer survivors treated with anthracyclines. Eur J Cancer. 2006;42(15):2549-53. 10.1016/j.ejca.2006.04.014 16919450

[248] Hines MR, Mulrooney DA, Hudson MM, Ness KK, Green DM, Howard SC, et al.Pregnancy-associated cardiomyopathy in survivors of childhood cancer. J Cancer Surviv. 2016;10(1):113-21. 10.1007/s11764-015-0457-8 PMC4670599 26044903

[249] Bar J, Davidi O, Goshen Y, Hod M, Yaniv I, Hirsch R. Pregnancy outcome in women treated with doxorubicin for childhood cancer. Am J Obstet Gynecol. 2003;189(3):853-7. 10.1067/s0002-9378(03)00837-8 14526329

[250] Thompson KA, Hildebrandt MA, Ater JL. Cardiac Outcomes With Pregnancy After Cardiotoxic Therapy for Childhood Cancer. J Am Coll Cardiol. 2017;69(5):594-5. 10.1016/j.jacc.2016.11.040 28153113

[251] Stergiopoulos K, Shiang E, Bench T. Pregnancy in patients with pre-existing cardiomyopathies. J Am Coll Cardiol. 2011;58(4):337-50. 10.1016/j.jacc.2011.04.014 21757110

[252] Athale U, Siciliano S, Thabane L, Pai N, Cox S, Lathia A, et al.Epidemiology and clinical risk factors predisposing to thromboembolism in children with cancer. Pediatr Blood Cancer. 2008;51(6):792-7. 10.1002/pbc.21734 18798556

[253] Caruso V, Iacoviello L, Di Castelnuovo A, Storti S, Mariani G, de Gaetano G, et al.Thrombotic complications in childhood acute lymphoblastic leukemia: a meta-analysis of 17 prospective studies comprising 1752 pediatric patients. Blood. 2006;108(7):2216-22. 10.1182/blood-2006-04-015511 16804111

[254] Biss TT, Brandao LR, Kahr WH, Chan AK, Williams S. Clinical features and outcome of pulmonary embolism in children. Br J Haematol. 2008;142(5):808-18. 10.1111/j.1365-2141.2008.07243.x 18564359

[255] Kuderer NM, Ortel TL, Francis CW. Impact of venous thromboembolism and anticoagulation on cancer and cancer survival. J Clin Oncol. 2009;27(29):4902-11. 10.1200/JCO.2009.22.4584 PMC2799059 19738120

[256] Revel-Vilk S, Yacobovich J, Tamary H, Goldstein G, Nemet S, Weintraub M, et al.Risk factors for central venous catheter thrombotic complications in children and adolescents with cancer. Cancer. 2010;116(17):4197-205. 10.1002/cncr.25199 20533566

[257] Chow EJ, Anderson L, Baker KS, Bhatia S, Guilcher GM, Huang JT, et al.Late Effects Surveillance Recommendations among Survivors of Childhood Hematopoietic Cell Transplantation: A Children’s Oncology Group Report. Biol Blood Marrow Transplant. 2016;22(5):782-95. 10.1016/j.bbmt.2016.01.023 PMC4826622 26802323

[258] Armenian SH, Sun CL, Kawashima T, Arora M, Leisenring W, Sklar CA, et al.Long-term health-related outcomes in survivors of childhood cancer treated with HSCT versus conventional therapy: a report from the Bone Marrow Transplant Survivor Study (BMTSS) and Childhood Cancer Survivor Study (CCSS). Blood. 2011;118(5):1413-20. 10.1182/blood-2011-01-331835 PMC3152502 21652685

[259] Armand P, Kim HT, Cutler CS, Ho VT, Koreth J, Alyea EP, et al.Prognostic impact of elevated pretransplantation serum ferritin in patients undergoing myeloablative stem cell transplantation. Blood. 2007;109(10):4586-8. 10.1182/blood-2006-10-054924 PMC1885508 17234738

