# Supplementary material for: Posicionamento Brasileiro sobre o Uso da Multimodalidade de Imagens na Cardio-Oncologia – 2021
Source: Arq Bras Cardiol. 2021 Oct 6;117(4):845–909. [Article in Portuguese] doi: 10.36660/abc.20200266 (PMC8528353; doi:10.36660/abc.20200266)
Supplement: Translation

# Translation: Brazilian Position Statement on the Use Of Multimodality Imaging in Cardio-Oncology – 2021

**Authors:** Marcelo Dantas Tavares de Melo, Marcelo Goulart Paiva, Maria Verônica Câmara Santos, Carlos Eduardo Rochitte, Valéria de Melo Moreira, Mohamed Hassan Saleh, Simone Cristina Soares Brandão, Claudia Cosentino Gallafrio, Daniel Goldwasser, Eliza de Almeida Gripp, Rafael Bonafim Piveta, Tonnison Oliveira Silva, Thais Harada Campos Espirito Santo, Waldinai Pereira Ferreira, Vera Maria Cury Salemi, Sanderson A. Cauduro, Silvio Henrique Barberato, Heloísa M. Christovam Lopes, José Luiz Barros Pena, Heron Rhydan Saad Rached, Marcelo Haertel Miglioranza, Aurélio Carvalho Pinheiro, Bárbara Athayde Linhares Martins Vrandecic, Cecilia Beatriz Bittencourt Viana Cruz, César Higa Nomura, Fernanda Mello Erthal Cerbino, Isabela Bispo Santos da Silva Costa, Otavio Rizzi Coelho, Adriano Camargo de Castro Carneiro, Ursula Maria Moreira Costa Burgos, Juliano Lara Fernandes, Marly Uellendahl, Eveline Barros Calado, Tiago Senra, Bruna Leal Assunção, Claudia Maria Vilas Freire, Cristiane Nunes Martins, Karen Saori Shiraishi Sawamura, Márcio Miranda Brito, Maria Fernanda Silva Jardim, Renata Junqueira Moll Bernardes, Tereza Cristina Diógenes, Lucas de Oliveira Vieira, Claudio Tinoco Mesquita, Rafael Willain Lopes, Elry Medeiros Vieira Segundo, Letícia Rigo, Valeska Leite Siqueira Marin, Marcelo José Santos, Gabriel Blacher Grossman, Priscila Cestari Quagliato, Monica Luiza de Alcantara, José Aldo Ribeiro Teodoro, Ana Cristina Lopes Albricker, Fanilda Souto Barros, Salomon Israel do Amaral, Carmen Lúcia Lascasas Porto, Marcio Vinícius Lins Barros, Simone Nascimento dos Santos, Armando Luís Cantisano, Ana Cláudia Gomes Pereira Petisco, José Eduardo Martins Barbosa, Orlando Carlos Glória Veloso, Salvador Spina, Ricardo Pignatelli, Ludhmilla Abrahão Hajjar, Roberto Kalil, Marcelo Antônio Cartaxo Queiroga Lopes, Marcelo Luiz Campos Vieira, André Luiz Cerqueira Almeida

**Affiliations:** 1 João PessoaPB Brazil Universidade Federal da Paraíba, João Pessoa, PB – Brazil; 2 São PauloSão Paulo Brazil Hospital 9 de Julho, Cardiologia, São Paulo, São Paulo – Brazil; 3 São PauloSP Brazil Sociedade Brasileira de Oncologia Pediátrica, São Paulo, SP – Brazil; 4 São PauloSP Brazil Instituto do Coração (Incor) do Hospital das Clínicas da Faculdade de Medicina da Universidade de São Paulo (HCFMUSP), São Paulo, SP – Brazil; 5 São PauloSP Brazil Hospital do Coração (HCOR), São Paulo, SP – Brasil.; 6 São PauloSP Brazil Instituto Dante Pazzanese de Cardiologia, São Paulo, SP – Brazil; 7 RecifePE Brazil Hospital das Clínicas, Universidade Federal de Pernambuco, Recife, PE - Brazil; 8 João PessoaPB Brazil Clínica Diagson, João Pessoa, PB – Brazil; 9 São PauloSP Brazil Instituto de Oncologia Pediátrica, São Paulo, SP – Brazil; 10 Rio de JaneiroRJ Brazil Hospital Federal de Ipanema, Rio de Janeiro, RJ – Brazil; 11 Rio de JaneiroRJ Brazil Hospital Copa D'Or, Rio de Janeiro, RJ – Brazil; 12 Rio de JaneiroRJ Brazil Casa de Saúde São José, Rio de Janeiro, RJ – Brazil; 13 Rio de JaneiroRJ Brazil Hospital Pró-Cardíaco, Rio de Janeiro, RJ – Brazil; 14 Rio de JaneiroRJ Brazil Hospital Universitário Antônio Pedro, Rio de Janeiro, RJ – Brazil; 15 São PauloSP Brazil Hospital Israelita Albert Einstein, São Paulo, SP – Brazil; 16 SalvadorBA Brazil Hospital Cardio Pulmonar – Centro de Estudos em Cardiologia, Salvador, BA – Brazil; 17 SalvadorBA Brazil Escola Bahiana de Medicina e Saúde Pública, Salvador, BA – Brazil; 18 SalvadorBA Brazil Hospital Ana Nery, Salvador, BA – Brazil; 19 SalvadorBA Brazil Diagnoson/Fleury, Salvador, BA – Brazil; 20 São PauloSP Brazil ACCamargoCancer Center – IMAGE, São Paulo, SP – Brazil; 21 CuritibaPR Brazil Hospital Erasto Gaertner, Curitiba, PR – Brazil; 22 CuritibaPR Brazil CardioEco Centro de Diagnóstico Cardiovascular, Curitiba, PR – Brazil; 23 CuritibaPR Brazil Quanta Diagnóstico, Curitiba, PR – Brazil; 24 São PauloSP Brazil Hospital de Amor, Barretos, São Paulo, SP – Brazil; 25 Belo HorizonteMG Brazil Faculdade de Ciências Médicas de Minas Gerais, Belo Horizonte, MG – Brazil; 26 São PauloSP Brazil Hospital Leforte Liberdade, São Paulo, SP – Brazil; 27 Porto AlegreRS Brazil Instituto de Cardiologia do Rio Grande do Sul – Laboratório de Pesquisa e Inovação em Imagem Cardiovascular, Porto Alegre, RS – Brazil; 28 Porto AlegreRS Brazil Hospital Mãe de Deus, Porto Alegre, RS – Brazil; 29 ManausAM Brazil Hospital Adventista de Manaus, Manaus, AM – Brazil; 30 Nova LimaMG Brazil Biocor Instituto, Nova Lima, MG – Brazil; 31 São PauloSP Brazil Hospital Sírio-Libanês, São Paulo, SP – Brazil; 32 Rio de JaneiroRJ Brazil Clínica de Diagnóstico por Imagem, Rio de Janeiro, RJ – Brazil; 33 Rio de JaneiroRJ Brazil Diagnósticos da América AS, Rio de Janeiro, RJ – Brazil; 34 São PauloSP Brazil Universidade de São Paulo Instituto do Câncer do Estado de São Paulo, São Paulo, SP – Brazil; 35 CampinasSP Brazil Universidade Estadual de Campinas (UNICAMP), Campinas, SP – Brazil; 36 AracajuSE Brazil Universidade Tiradentes, Aracaju, SE – Brazil; 37 CampinasSP Brazil Radiologia Clínica de Campinas, Campinas, SP – Brazil; 38 CampinasSP Brazil Instituto de Ensino e Pesquisa José Michel Kalaf, Campinas, SP – Brazil; 39 São PauloSP Brazil Universidade Federal de São Paulo (UNIFESP), São Paulo, SP – Brazil; 40 RecifePE Brazil Hospital das Clínicas da Universidade Federal de Pernambuco, Recife, PE – Brazil; 41 Belo HorizonteMG Brazil Universidade Federal de Minas Gerais (UFMG), Belo Horizonte, MG – Brazil; 42 Belo HorizonteMG Brazil ECOCENTER, Belo Horizonte, MG – Brazil; 43 São PauloSP Brazil Instituto da Criança da Universidade de São Paulo (USP), São Paulo, SP – Brazil; 44 AraguainaTO Brazil Universidade Federal do Tocantins – Campus de Araguaina, Araguaina, TO – Brazil; 45 AraguainaTO Brazil Hospital Municipal de Araguaina, Araguaina, TO – Brazil; 46 São PauloSP Brazil Hospital Samaritano de São Paulo, São Paulo, SP – Brazil; 47 Rio de JaneiroRJ Brazil Instituto D'Or de Pesquisa e Ensino, Rio de Janeiro, RJ – Brazil; 48 FortalezaCE Brazil Hospital Infantil Albert Sabin, Fortaleza, CE – Brazil; 49 SalvadorBA Brazil Hospital São Rafael, Salvador, BA – Brazil; 50 SalvadorBA Brazil Rede D'Or, Salvador, BA – Brazil; 51 Rio de JaneiroRJ Universidade Federal Fluminense (UFF), Rio de Janeiro, RJ - Brazil; 52 Brazil Hospital Vitória, Rio de Janeiro, RJ – Brazil; 53 São PauloSP Brazil Hospital Beneficência Portuguesa, São Paulo, SP – Brazil; 54 São PauloSP Brazil Hospital Samaritano, São Paulo, SP – Brazil; 55 São PauloSP Brazil Santa Casa de Misericórdia, São Paulo, SP – Brazil; 56 BarretosSP Brazil Hospital de Câncer de Barretos, Barretos, SP – Brazil; 57 Porto AlegreRS Brazil Clínica Cardionuclear, Porto Alegre, RS – Brazil; 58 Porto AlegreRS Brazil Hospital Moinhos de Vento, Porto Alegre, RS – Brazil; 59 Rio de JaneiroRio Brazil Americas Medical City, Rio de Janeiro, Rio de Janeiro, RJ – Brazil; 60 Rio de JaneiroRJ Brazil Americas Serviços Médicos, Rio de Janeiro, RJ – Brazil; 61 Rio de JaneiroRJ Brazil Rede D'Or, Rio de Janeiro, RJ – Brazil; 62 Ribeirão PretoSP Brazil Prenoto Medicina Diagnóstica, Ribeirão Preto, SP – Brazil; 63 Belo HorizonteMG Brazil Centro Universitário Unihorizontes, Belo Horizonte, MG – Brazil; 64 VitóriaES Brazil Angiolab Vitória – Diagnóstico Vascular, Vitória, ES – Brazil; 65 Rio de JaneiroRJ Brazil Casa de Saúde Nossa Senhora do Carmo, Rio de Janeiro, RJ – Brazil; 66 Rio de JaneiroRJ Brazil Universidade do Estado do Rio de Janeiro Faculdade de Ciências Médicas, Rio de Janeiro, RJ – Brazil; 67 Belo HorizonteMG Brazil Mater Dei Rede de Saúde, Belo Horizonte, MG – Brazil; 68 Belo HorizonteMG Brazil Hospital Vera Cruz, Belo Horizonte, MG – Brazil; 69 BrasíliaDF Brazil Hospital Brasília – Ecocardiografia, Brasília, DF – Brazil; 70 BrasíliaDF Brazil Eccos Diagnóstico Cardiovascular Avançado, Brasília, DF – Brazil; 71 Rio de JaneiroRJ Brazil Hospital Barra D'Or, Rio de Janeiro, RJ – Brazil; 72 Rio de JaneiroRJ Brazil Rede UHG, Rio de Janeiro, RJ – Brazil; 73 Buenos Aires Argentina Hospital Aeronáutico Central, Buenos Aires – Argentina; 74 HoustonTexas USA Texas Children's Hospital, Houston, Texas – USA; 75 HoustonTexas USA Baylor College of Medicine, Houston, Texas – USA; 76 João PessoaPB Brazil Hospital Alberto Urquiza Wanderley – Hemodinâmica e Cardiologia Intervencionista, João Pessoa, PB – Brazil; 77 João PessoaPB Brazil Hospital Metropolitano Dom José Maria Pires, João Pessoa, PB – Brazil; 78 Rio de JaneiroRJ Brazil Sociedade Brasileira de Cardiologia, Rio de Janeiro, RJ – Brazil; 79 Feira de SantanaBA Brazil Santa Casa de Misericórdia de Feira de Santana – Cardiologia, Feira de Santana, BA – Brazil; 80 São PauloSP Brazil Departamento de Imagem Cardiovascular da Sociedade Brasileira de Cardiologia, São Paulo, SP – Brazil

**Table:** 

Declaration of potential conflict of interests of authors/collaborators of the Brazilian Position Statement on the Use Of Multimodality Imaging in Cardio-Oncology – 2021							
If, within the last 3 years, the author/collaborator of the statement:							
Names of statement collaborators	Participated in clinical and/or experimental studies sponsored by pharmaceutical or equipment companies related to thxxxis statement	Spoke at events or activities sponsored by industry related to this statement	Was (is) a member of a board of advisors or a board of directors of a pharmaceutical or equipment industry	Participated in normative committees of scientific research sponsored by industry	Received personal or institutional funding from industry	Wrote scientific papers in journals sponsored by industry	Owns stocks in industry
Adriano Camargo de Castro Carneiro	No	No	No	No	No	No	No
Ana Cláudia Gomes Pereira Petisco	No	No	No	No	No	No	No
Ana Cristina Lopes Albricker	No	No	No	No	No	No	No
André Luiz Cerqueira de Almeida	No	No	No	No	No	No	No
Armando Luís Cantisano	No	No	No	No	No	No	No
Aurélio Carvalho Pinheiro	No	No	No	No	No	No	No
Bárbara Arhayde Lihares Martins Vrandecic	No	No	No	No	No	No	No
Bruna Leal Assunção	No	No	No	No	No	No	No
Carlos Eduardo Rochitte	No	No	No	No	No	No	No
Carmen Lucia Lascasas Porto	No	No	No	No	No	No	No
Cecilia Beatriz Bittencourt Viana Cruz	No	No	No	No	No	No	No
César Higa Nomura	No	No	No	No	No	No	No
Cláudia Cosentino Gallafrio	No	No	No	No	No	No	No
Cláudia Maria Vilas Freire	No	No	No	No	No	No	No
Claudio Tinoco Mesquita	No	No	No	No	No	No	No
Cristiane Nunes Martins	No	No	No	No	No	No	No
Daniel Goldwasser	No	No	No	No	No	No	No
Eliza de Almeida Gripp	No	No	No	No	No	No	No
Elry Medeiros Vieira Segundo Neto	No	No	No	No	No	No	No
Eveline Barros Calado	No	No	No	No	No	No	No
Fanilda Souto Barros	No	No	No	No	No	No	No
Fernanda Mello Erthal Cerbino	No	No	No	No	No	No	No
Gabriel Blacher Grossman	No	No	No	No	No	No	No
Heloísa Helena M. Christovam Lopes	No	No	No	No	No	No	No
Heron Rhydan Saad Rached	No	No	No	No	No	No	No
Isabela Bispo Santos da Silva Costa	No	No	No	No	No	No	No
José Aldo Ribeiro Teodoro	No	No	No	No	No	No	No
José Eduardo Martins Barbosa	No	No	No	No	No	No	No
José Luiz Barros Pena	No	No	No	No	No	No	No
Juliano Lara Fernandes	No	No	No	No	No	No	No
Karen Saori Shiraishi Sawamura	No	No	No	No	No	No	No
Letícia Rigo	No	No	No	No	No	No	No
Lucas de Oliveira Vieira	No	No	No	No	No	No	No
Ludhmila Abrahão Hajjar	No	No	No	No	No	No	No
Marcelo Antônio Cartaxo Queiroga Lopes	No	No	No	No	No	No	No
Marcelo Dantas Tavares de Melo	No	No	No	No	No	No	No
Marcelo Goulart Paiva	No	No	No	No	No	No	No
Marcelo Haertel Miglioranza	No	No	No	No	No	No	No
Marcelo Luiz Campos Vieira	No	No	No	No	No	No	No
Marcelo Santos	No	No	No	No	No	No	No
Márcio Miranda Brito	No	No	No	No	No	No	No
Márcio Vinícius Lins Barros	No	No	No	No	No	No	No
Maria Fernanda Silva Jardim	No	No	No	No	No	No	No
Maria Verônica Câmara dos Santos	No	No	No	No	No	No	No
Marly Uellendahl	No	No	No	No	No	No	No
Mohamed Hassan Saleh	No	No	No	No	No	No	No
Mônica Luiza de Alcantara	No	No	No	No	No	No	No
Orlando Carlos Glória Veloso	No	No	No	No	No	No	No
Otávio Rizzi Coelho-Filho	No	No	No	No	No	No	No
Priscila Cestari Quagliato	No	No	No	No	No	No	No
Rafael Bonafim Piveta	No	No	No	No	No	No	No
Rafael Willain Lopes	No	No	No	No	No	No	No
Renata Junqueira Moll Bernardes	No	No	No	No	No	No	No
Ricardo Pignatelli	No	No	No	No	No	No	No
Roberto Kalil Filho	No	No	No	No	No	No	No
Salomon Israel do Amaral	No	No	No	No	No	No	No
Salvador Spina	No	No	No	No	No	No	No
Sanderson A. Cauduro	No	No	No	No	No	No	No
Silvio Henrique Barberato	No	No	No	No	No	No	No
Simone Cristina Soares Brandão	No	No	No	No	No	No	No
Simone Nascimento dos Santos	No	No	No	No	No	No	No
Tereza Cristina Diógenes	No	No	No	No	No	No	No
Thais Harada Campos Espirito Santo	No	No	No	No	No	No	No
Tiago Senra	No	No	No	No	No	No	No
Tonnison de Oliveira Silva	No	No	No	No	No	No	No
Ursula Maria Moreira Costa Burgos	No	No	No	No	No	No	No
Valéria de Melo Moreira	No	No	No	No	No	No	No
Valeska Leite	No	No	No	No	No	No	No
Vera Maria Cury Salemi	No	No	No	No	No	No	No
Waldinai P. Ferreira	No	No	No	No	No	No	No
Vera Maria Cury Salemi	No	No	No	No	No	No	No
Waldinai P. Ferreira	No	No	No	No	No	No	No

## 1. General Aspects

### 1.1. Current Situation of Cardio-Oncology in Brazil and Worldwide

The estimated incidence of cancer in Brazil was 600 thousand cases per year in 2018 and 2019. ^1^ Only as of 2005, the survival rate surpassed that of overall cancer mortality, leading to an increased number of survivors exposed to the risk of cardiotoxicity (CTX), which is currently the second leading cause of morbidity and mortality in this population. ^2^

Cardiovascular complications resulting from cancer treatment, which are the focus of this consensus statement, may result in premature deaths, costly hospitalizations, and absence from work, leading to the need for early diagnosis and interventions. ^3^

Age (children and older adults), previous myocardial or coronary heart disease, hypertension, diabetes mellitus (DM), smoking, alcohol consumption, and sedentary lifestyle are factors associated with increased risk of CTX. ^4^

Recent studies suggest that genotypic variants may modify the susceptibility to CTX, turning genetic mapping into a promising field for identification of risk subgroups. ^5^

It is recommended that patients at high-risk for development of CTX be considered those whose treatment includes: ^6^

- High-dose anthracycline (doxorubicin > 250 mg/m² or epirubicin > 600 mg/m²);
- Radiotherapy at a dose ≥ 30 Gy (involving the heart) or > 2 Gy/session;
- Lower doses of combined anthracycline and radiotherapy;

➢ Lower doses of anthracyclines or trastuzumab alone, but associated with:
➢ More than two cardiovascular risk factors (smoking, hypertension, DM, dyslipidemia, obesity – during or after therapy);
➢ Age ≥ 60 years;
➢ Structural heart disease before or during treatment (ejection fraction: 50% to 55%, acute myocardial infarction [AMI], moderate/important valve disease);
➢ Combination of low doses of anthracycline and trastuzumab.

### 1.2. Definition of Cardiotoxicity

The definition of CTX based on the degree of left ventricular ejection fraction (LVEF) reduction ignores the changes that precede the fall in LVEF and all other toxic effects that occur in addition to this parameter. ^6^^-^^8^ Lack of a more comprehensive definition and, sometimes, clinical, laboratory, and imaging limitations to document some events in the initial stage make CTX an underdiagnosed clinical condition. The European Society of Cardiology revised in 2017 the definition of CTX to cover any structural or functional changes in the heart and circulation, both in the presence or in the immediate or late post-treatment of cancer, and considered chemotherapy, radiotherapy, or the disease itself as aggressive agents. ^4^

### 1.3. Mechanisms of Cardiotoxicity

Although we are aware of some CTX-related mechanisms, identifying the predominant mechanism remains a great challenge, as the combination of different drugs and treatment protocols as well as constitutional factors inherent to each patient account for a complex interaction that results in damage to the cardiovascular system ( Table 1 ). Depending on the chemotherapy agent class, cell damage may occur directly or indirectly and with or without potential for reversibility. ^9^ Ewer et al. ^10^ proposed in 2005 a classification of CTX in types 1 and 2; despite being the subject of much criticism, it has helped divide CTX into irreversible cell damage (type 1), attributed to anthracyclines, and reversible dysfunctions (type 2), attributed to trastuzumab. With the development of new anticancer therapies, including Bruton tyrosine kinase inhibitors, proteasome inhibitors, checkpoint inhibitors, among others known to be potentially cardiotoxic, it seems that this classification proposal deserves to be revised and expanded.

**Table 1:** Summary of the main suggested mechanisms of cardiotoxicity by group of drugs

Anthracyclines	DNA double-strand break (topoisomerase IIB)
	Oxidative stress (reactive oxygen species)
	Cell membrane hyperpermeability (lipid peroxidation)
	Ultrastructural changes
	Cytoplasmic vacuolization
	Apoptosis
Trastuzumab	Interruption of HER-2/ERBB2 receptor signaling – Neuregulin 1
	Inhibits cell repair
	Cell dysfunction
Cisplatin Cyclophosphamide	Direct endothelial injury
	Platelet activation and aggregation
	Coronary thrombosis
5-Fluorouracil	Acts on the molecular signaling pathway that regulates smooth muscle tone
	Vasospasm – vasoconstriction
Vascular endothelial growth factor (VEGF) inhibitors	Inhibit nitric oxide synthase activity
	Increase endothelin production
	Inhibit rho-kinase activation
	Vasospasm
Protease inhibitors	Interference with the degradation of dysfunctional proteins
	Functional changes in the myocyte
Immune checkpoint inhibitors	Increased T-cell activity
	Autoimmune activity in the heart muscle

### 1.4. Clinical Manifestations of Cardiotoxicity

The cardiovascular clinical manifestations arising from cancer treatment are the tip of an iceberg whose base consists of structural and functional changes that precede signs and symptoms. For didactic purposes, we chose to divide CTX manifestations into three subgroups: clinical, laboratory, and imaging/tracing ( Table 2 ). It should be noted that such proposal may be criticized at first, as routine genetic mapping is not yet feasible to determine more accurately the culpability of the phenotypic expression.

**Tabble:** Cardiotoxicity phenotypes

Clinical status	Hypertension
	Pulmonary hypertension
	Venous and arterial embolic events
	Carotid artery disease
	Heart failure/myocarditis
	Pericardial effusion/pericarditis
	Valve dysfunctions
	Myocardial ischemia/infarction
	Pericardial disease
Laboratory	Elevated troponins (T or I) and/or CK-MB
	Elevated natriuretic peptide (BNP/NT-proBNP)
Imaging/Tracing	Heart rhythm disorders (extrasystoles, blocks, supraventricular and ventricular tachycardias, bradyarrhythmias, increased corrected QT interval on electrocardiogram)
	Dilated cardiac chambers with preserved LVEF
	LVEF reduction > 10% (baseline) or > 15% (global longitudinal strain)
	Left ventricular diastolic dysfunction
	Pericardial thickening and/or effusion
	Valve dysfunctions (stenosis, failures)
	Changes in imaging tests that indicate active inflammatory signs or necrosis (scintigraphy/cardiac MRI)
	Changes in coronary CT angiography or calcium score that were initiated or aggravated during or after cancer treatment (chemotherapy and/or radiotherapy)

BNP: brain natriuretic peptide; CT: computed tomography; LVEF: left ventricular ejection fraction; MRI: magnetic resonance imaging.

Anthracyclines and anti-HER2 monoclonal antibodies account for most documented cases of left ventricular (LV) dysfunction. Cardinale et al. ^11^ demonstrated that the incidence of CTX for anthracycline use in a population of 2,625 patients was 9%, with 98% of cases occurring in the first year of treatment. ^11^ Alkylating agents, proteasome inhibitors, and some tyrosine kinase inhibitors also cause dysfunction by means of several mechanisms. ^4^ Severe inflammatory myocarditis may be associated with immune checkpoint inhibitors in 0.27% of patients in use of a combination of nivolumab and ipilimumab. ^12^

Coronary artery disease (CAD), clinically manifested as stable or unstable angina or AMI, may be secondary to direct endothelial injury, acute arterial thrombosis, or vasospasm, depending on the therapeutic class that was used. Obstructive atherosclerosis, plaque rupture and coronary thrombosis, annular/valvular degenerations, and pericarditis are related to mediastinal radiotherapy and are dependent on the radiation dose that was used. Hypertension is closely linked to the use of endothelial growth factor inhibitors. Deep venous thrombosis (DVT), peripheral artery disease, and pulmonary hypertension are also within the range of clinical manifestations of CTX. ^4^

## 2. Myocardial Cardiotoxicity

### 2.1. Contribution from Echocardiography

#### 2.1.1. Myocardial Structural and Functional Assessment of the Left Ventricle

##### 2.1.1.1. Standard Doppler Echocardiography

When myocardial dysfunction was recognized as a potential adverse effect of cancer treatment, several strategies were then tested to monitor myocardial function. Initially considered to be the most accurate method, endomyocardial biopsy quickly fell into disuse because of its invasive nature, being then replaced by serial monitoring of LV systolic function by noninvasive cardiovascular imaging tests.

Echocardiography has become a consolidated method for monitoring CTX using LVEF, as it is widely available, cost-effective, and harmless, allowing for it to be repeated multiple times. Additionally, it provides several other anatomical and functional findings.

Administration of the Simpson method improves the estimation of ventricular volumes, overcoming the limitations of fractional shortening and the Teichholz formula, obtained from linear measurements of M-mode or two-dimensional (2D) echocardiography. However, sensitivity to detect small longitudinal variations in systolic function remains low, mainly because of frequent preload and afterload variations during chemotherapy and intra- and inter-observer variability (one of the most accepted parameters for diagnosis of CTX), which may reach up to 10%. ^13^ It is important to remember that, because of those variations, tests with results outside the expected parameters should be repeated and confirmed 2 to 3 weeks after the initial finding.

The risk of CTX is considered to range from 3.6 to 11.8 times for use of cardiotoxic drugs (especially anthracyclines) if pre-treatment LVEF is between 50% and 55%. During monitoring and after cancer treatment, CTX identification should be based on a fall > 10% in LVEF (compared to pre-treatment values) to less than 50. ^14^ This situation is the subject of an important debate in medical teams regarding cardiological risk and cancer benefit. The discussed approaches include replacing it with lower cardiotoxic risk treatments, using cardioprotective measures, and even discontinuing treatment (if LVEF < 45% for anthracyclines and < 40% for the other classes), a decision always made together with the oncologist. ^14^^,^^15^

The assessment of longitudinal systolic function, especially when advanced methods (three-dimensional [3D] echocardiography and myocardial deformation analysis) are unavailable, should be performed jointly. Although there are no reference ranges for diagnosis, a progressive decline in the measurement of mitral annular peak systolic velocity by tissue Doppler imaging (s' wave) and mitral annular plane systolic excursion (MAPSE) is significant. ^16^

The number of times that echocardiographic imaging is required remains controversial in the literature, varying according to individual risk, therapeutic protocol (drugs used and total dosage), and identification of CTX signs and symptoms.

It is important to remember that CTX, in the form of quantitative changes in conventional parameters for assessing systolic function, may not be evident until there is a substantial reduction in myocardial reserve. Thus, cardiac damage may not become apparent for years or even decades after the end of cardiotoxic treatment, a fact that is particularly applicable to adult survivors of tumors during childhood.

##### 2.1.1.2. Myocardial Strain

Strain, or deformation, is defined as the amount of deformation or the fractional change in the length of a myocardial segment from initial length. Such parameter is expressed as a percentage (%) and with the negative sign. ^17^

Two-dimensional strain imaging, deriving from speckle tracking, is not dependent on the angle (a limiting factor when tissue Doppler imaging is used), which makes it more reproducible and more commonly used in general clinical practice to detect early changes in myocardial mechanics. ^17^ Three-dimensional strain imaging represents an improvement of the technique. In this modality, an entire pyramidal volume is obtained from the apical view and then analyzed, being much faster than the other modalities but having lower spatial and temporal resolution.

The fall in LVEF is a late marker of myocardial damage and translates into a poor prognosis, with reduced chance of ventricular function recovery in 58% of patients, despite any intervention with cardioprotective drugs. Cardiac dysfunction only becomes evident when myocardial damage is significant; therefore, absence of LVEF reduction does not exclude CTX. ^18^^,^^19^

Thus, administration of speckle-tracking strain imaging to analyze ventricular mechanics is gradually extending to all heart diseases, especially those associated with the use of anticancer agents, such as anthracyclines and trastuzumab. ^20^ The possibility of detecting subclinical lesions has been one of the great advantages. Overall, although early change detection is conceptually important, the value of actual changes must be proven to correlate with the outcomes.

A review of several studies demonstrated the ability of strain imaging to detect myocardial deformation changes earlier than the fall in LVEF, either immediately after therapy infusion or in later stages. ^21^

Ganame et al. ^22^ demonstrated the acute effects of anthracyclines, which are able to induce systolic dysfunction. ^22^ The same group of investigators studied 56 patients without any risk factors for cardiovascular disease (CVD), diagnosed with lymphoma, leukemia, and other malignant tumors, treated only with anthracyclines (dose lower than 300 mg/m^2^), and compared them with a control group. ^23^ After a mean follow-up of 5.2 years, a significant reduction in global longitudinal strain (GLS) was demonstrated at a time point when LVEF was still normal, showing that new diagnostic tools are able to predict this decline early in time.

Sawaya et al. ^24^ used 2D speckle tracking to demonstrate that GLS and troponin were predictors of systolic dysfunction in patients with breast cancer treated with anthracyclines and trastuzumab. ^24^ Forty-three patients underwent echocardiography at baseline and at three and six months of treatment. LVEF using the Simpson biplane method, GLS, radial and circumferential strain, and biomarkers were assessed. In that study, GLS was able to predict CTX in seven out of nine patients, with a sensitivity of 78% and a specificity of 79%. The event occurred at three months of follow-up in one of the patients and at six months in the others.

Tan et al. ^25^ examined LVEF and GLS in 19 patients with breast cancer using trastuzumab and followed-up for 34 months (mean 24.7 months). They observed that changes in ventricular function persisted for a long period, with increased LV chamber dimensions and reduced GLS throughout the entire follow-up, questioning the reversibility of the damage caused by trastuzumab. ^25^

Almeida et al. evaluated 40 patients with breast cancer who had used doxorubicin two years prior to undergoing an echocardiogram and compared them with 41 healthy women. The authors demonstrated that GLS and S’ wave of the mitral annulus were reduced in patients who underwent chemotherapy, but LVEF remained normal, suggesting the presence of subclinical ventricular dysfunction. The authors also showed that age and previous use of doxorubicin were independent markers of GLS reduction. ^26^

Recently, Piveta et al. ^27^ evaluated the role of 3D strain in patients with breast cancer treated with anthracyclines. After exposure to a low anthracycline dose (120 mg/m^2^), only 3D circumferential strain and 3D area strain showed changes, while 2D strain parameters remained unchanged. ^27^

A systematic review of 1,504 patients showed that a relative reduction of 10% to 15% in GLS from baseline was an important predictor for a decline in LVEF. Radial and circumferential strain measurements also show changes, but such variables are not yet routinely used. When pre-chemotherapy values are not available for comparison, GLS values greater than -19% are suggestive of CTX, and the association with biomarkers, especially ultrasensitive troponin, increases the sensitivity for diagnosis of CTX. It is worth noting that reference ranges may vary depending on the software used in the devices and age and sex of the patients, hence the recommendation to repeat the tests using always the same device and, preferably, the same examiner. ^21^

An expert consensus of the American and European Cardiovascular Imaging Societies suggests that changes in deformation precede ventricular dysfunction. ^28^ A reduction > 15% in GLS immediately after or during anthracycline therapy is the most useful parameter in predicting CTX, while a reduction > 8% will probably exclude the diagnosis of CTX ( Figure 1 ).

**Figure 1:** Clinical monitoring and management during cardiotoxic therapy.

Liu et al. ^29^ described in 2018 an algorithm to follow-up patients treated with anticancer agents that used LVEF and GLS as echocardiographic measures. In patients with LVEF > 60%, the recommendation was to optimize the control of existing cardiovascular risk factors. Those with LVEF between 50% and 59% and with GLS lower than -16% or at the lower limit of normal were classified as preserved myocardial function; those with GLS greater than -16% or a 15% reduction from baseline were considered to have subclinical dysfunction. Patients with LVEF between 40% and 49% were considered to have myocardial dysfunction; thus, this specific group was indicated for initiation of cardioprotective therapy and a joint evaluation with the oncologist on the risks and benefits of anticancer therapy, with an occasional dose reduction or medication change. In patients with LVEF < 40%, it is recommended that cardioprotective therapy is initiated and the use of a noncardiotoxic alternative therapy is discussed with the oncologist.

There is no consensus on systolic function indices to be monitored during treatment. ^14^^,^^15^^,^^29^ However, recently, the SUCCOUR (Strain sUrveillance of Chemotherapy for improving Cardiovascular Outcomes) study was published. It showed that treatment guided by a greater than 12% drop in the LV global longitudinal strain in patients treated with anthracyclines is able to prevent the drop in ejection fraction and cardiotoxicity in 1 year. ^30^ In addition to the diagnosis of CTX, the identification of GLS reduction has prognostic value, as it has been associated with higher late mortality in a retrospective study involving 120 patients followed-up for 21.6 ± 13.9 months. ^31^

##### 2.1.1.3. Left Ventricular Ejection Fraction by 3D Imaging

Three-dimensional echocardiographic imaging is the method of choice for calculating LVEF during cancer treatment ( Figure 2 ). ^32^ By providing greater resemblance to cardiac anatomy, it is much consistent with cardiac magnetic resonance imaging (MRI) in the calculation of volumes, mass, and LVEF. ^33^ The 3D analysis is not dependent on geometric assumptions, as is the case of 2D analysis, in addition to minimizing limitations related to that technique such as “apical shortening.”

**Figure 2:** Example of a tree-dimensional echocardiogram with full volume analysis and estimated volumes and left ventricular ejection fraction.

The predominant CTX change for a consequent decrease in LVEF is an increase in LV end-systolic volume. ^15^ In the oncology population, studies suggest that 3D imaging is preferable to 2D imaging mainly because the former has demonstrated greater reproducibility and greater accuracy in the recognition of borderline or slightly reduced LVEF. In survivors of cancer treated with anthracyclines, Armstrong et al. ^34^ demonstrated that 3D imaging had greater ability to identify patients with LVEF < 50% than 2D imaging, with an accuracy very similar to that of cardiac MRI, allowing for earlier identification of subclinical CTX. ^34^ The SUCCOUR study used two criteria of cardiotoxicity by preferentially 3D echocardiography: a fall of more than 5% in patients with symptoms of heart failure, or greater than 10% in asymptomatic patients, compared with the baseline test for values of ejection fraction lower than 55%. ^30^

In patients undergoing chemotherapy, Thavendiranathan et al. ^13^ compared different echocardiographic techniques for sequential assessment of LVEF over 1 year and demonstrated that 3D imaging had the lowest intra- and inter-observer temporal variability (5.6%). ^13^ This finding suggests that, in addition to being reliable, 3D imaging is a consistent and reproducible method for evaluation of patients with cancer. ^32^ Other papers also highlight the greater reproducibility of 3D imaging in the calculation of LVEF, mainly because, as a semiautomatic technique for endocardial tracing, it is less affected by variability in image acquisition. ^35^

##### 2.1.1.4. Contrast Echocardiography

Inadequate visualization of LV endocardial borders often occurs in patients undergoing chemotherapy for breast cancer, particularly when following mastectomy and radiotherapy. Consequently, underestimated volumes and inaccurate LVEF determination may occur. According to international guidelines, an ultrasound contrast agent should be used to improve the definition of endocardial borders and the analysis of LV function when endocardial visualization is limited in two or more segments. ^36^ Conversely, contrast agents are not recommended when LVEF is estimated on 3D echocardiogram, as they lead to less reproducibility and greater temporal variability in LVEF compared to 3D imaging alone. ^13^

##### 2.1.1.5. Stress Echocardiography

Exercise or pharmacological stress echocardiography is an established method for detecting obstructive CAD and subclinical changes in myocardial function. Patients with cancer often have a decrease in global cardiovascular reserve, attributed to the direct effects of adjuvant cancer therapy and/or the indirect effects of lifestyle changes associated with treatment. ^37^ Thus, the potential uses for stress echocardiography in patients undergoing cancer therapy include the following: (a) initial investigation of presence of obstructive CAD in patients with intermediate-to-high pre-test probability, noninterpretable ECG (exercise) or unable to exercise (dobutamine), especially if receiving chemotherapy associated with ischemia or after long-term radiotherapy; (b) determination of left ventricular contractile reserve (LVCR) as a predictor of CTX in patients with normal rest LVEF and GLS; (c) determination of LVCR in established CTX, as transient recovery of LV function during stress could indicate a better prognosis. ^28^ Despite those potentialities, stress echocardiography has been scarcely used in the field of cardio-oncology.

Using exercise stress echocardiography in 57 asymptomatic women with normal LVEF treated for breast cancer with anthracyclines, Khouri et al. ^38^ found a 12% reduction in stroke volume and a 24% reduction in cardiac index from rest when compared to controls, suggesting impaired LVCR. ^38^

Civelli et al. ^39^ prospectively measured LVCR (defined as the difference between peak and rest LVEF) using low-dose dobutamine stress echocardiography during and after high-dose chemotherapy in 49 women with advanced breast cancer. An asymptomatic decline ≥ 5% in LVCR from baseline was able to predict a fall in LVEF to < 50%. ^39^

The only published systematic review on the utility of cardiac stress methods for detecting CVD in survivors of breast cancer concluded that there seems to be evidence that stress echocardiography is beneficial to early prognostic evaluation and late follow-up after anthracycline therapy. ^40^

Before stress echocardiography can be routinely added to clinical practice in cardio-oncology, further studies are needed to determine the best stressor, which parameters should be measured during the test, the best time to perform the test according to the different types of treatment, cost-benefit and feasibility in the oncology population, and, finally, the presence of incremental prognostic value over traditional parameters measured at rest (LVEF and GLS).

##### 2.1.1.6. Diastolic Function

Abnormal parameters related to diastolic function, such as E and A waves, E/A ratio, isovolumic relaxation time, and myocardial performance index, have been described early after chemotherapy. ^41^^,^^42^ However, longitudinal studies have not been able to reproduce the prognostic value of those findings and there is insufficient evidence to recommend such assessment in the diagnosis of chemotherapy-induced CTX. ^43^

Studies have demonstrated the utility of tissue Doppler-derived measurements in the assessment of diastolic function in patients undergoing cancer treatment. Some papers have shown a reduction in tissue Doppler-derived early diastolic velocity (e’ wave) of the mitral annulus in patients treated with anthracyclines, which remained reduced during treatment and years later, but no predictive value for CTX was demonstrated. ^16^ Negishi et al. ^44^ revealed that a 10% reduction in e’ wave velocity was observed in patients who developed CTX after treatment with higher cumulative doses of doxorubicin, but this parameter was not shown to have a predictive role for LVEF fall. ^44^

The use of diastolic dysfunction as a specific CTX marker has been questioned. Abnormalities may occur due to preload changes resulting from volume replacements associated with cancer treatment or volume depletion associated with side effects of chemotherapy, such as nausea, vomiting, and diarrhea. In such cases, they may not represent an actual change in LV diastolic performance.

#### 2.1.2. Myocardial Structural and Functional Assessment of the Right Ventricle

The prevalence and prognostic value of right ventricular (RV) involvement have not yet been properly studied. Data on the influence of chemotherapy on RV remodeling, function, and mechanics are scarce and, at times, conflicting.

The difficulty inherent to RV assessment and hemodynamic variations suggests that parameters such as tricuspid annular plane systolic excursion (TAPSE), tissue velocity of the basal segment of the RV free wall (S’ wave), RV fractional area change (FAC), as well as myocardial strain are ideally measured by echocardiography, while the assessment of right ventricular ejection fraction (RVEF) is left to MRI or 3D echocardiography.

The drugs most frequently related to RV dysfunction and changes in pulmonary circulation are anthracyclines, trastuzumab, cyclophosphamide, and dasatinib. ^45^ Boczar et al. assessed RV longitudinal strain in patients with breast cancer treated with anthracycline. After 3 months, they observed that strain reduced from -16.2% to -13.81%. ^46^ The difference in RV longitudinal strain is more pronounced when the septum is excluded, suggesting a greater sensitivity of the RV myocardium. ^47^

#### 2.1.3. Late Echocardiographic Follow-up

Recommendations regarding patient follow-up after completion of chemotherapy will vary according to the clinical characteristics of the study population, the protocol used in chemotherapy (with or without radiotherapy), and the cumulative dose of drugs (especially trastuzumab and anthracyclines). Because of the increased risk of developing CTX within 12 months of anthracycline use, the fall in LVEF during trastuzumab use, and the appearance of late complications following radiotherapy, we suggest performing late follow-up as shown in Figure 3 .

**Figure 3:** Recommendations for late echocardiographic follow-up.

### 2.2. Contribution from Nuclear Medicine

#### 2.2.1. Radionuclide Ventriculography

Radionuclide ventriculography, often referred to as a multigated acquisition (MUGA) scan, is a scintigraphic test performed using a gamma chamber in the nuclear medicine (NM) department. This is a noninvasive method that produces low radiation exposure and does not cause severe side effects, with excellent reproducibility and low inter- and intra-observer variability in assessing ventricular function. ^48^

Together with echocardiography, radionuclide ventriculography is the most widely accepted method for assessing LVEF in patients before and during cancer treatment and identifying the risk for chronic HF (90% sensitivity and 72% specificity). ^49^

With a normal baseline LVEF, the next measurements should be made at cumulative doses of 250 to 300 and 400 to 450 mg/m^2^. For patients with abnormal baseline LVEF (< 50%), serial studies are recommended before each subsequent dose of doxorubicin. Figure 4 shows examples of LVEF behavior assessed by radionuclide ventriculography in patients undergoing chemotherapy.

**Figure 4:** Examples of two patients who underwent radionuclide ventriculography to measure left ventricular ejection fraction (LVEF) before and after anthracycline chemotherapy. The upper left image shows the baseline LVEF of the first patient, which was 71% (normal) and then decreased to 50% after the end of chemotherapy and start of trastuzumab therapy (upper right). The lower left image shows the baseline LVEF (52%) of a patient with breast cancer before the start of chemotherapy, which decreased to 46% during follow-up (lower right), leading to the use of cardioprotective medication. The images were kindly provided by Dr. Márcia Modesto (Nuclear Medicine Department of the Instituto Brasileiro de Controle do Câncer, São Paulo, Brazil).

#### 2.2.2. Assessment of Cardiac Sympathetic Activity with mIBG

Meta-iodobenzylguanidine (mIBG) is a molecule with a structure similar to that of norepinephrine and selectively acts on sympathetic nerves without, however, being metabolized by monoamine oxidase or catechol-O-methyltransferase, and not having a stimulatory effect as norepinephrine does. Cardiac ^123^ I-mIBG scintigraphy directly assesses the global and regional sympathetic function of the heart, including uptake, reuptake, storage, and release processes of norepinephrine at presynaptic nerve terminals. ^50^

Guimarães et al. ^51^ performed cardiac ^123^ I-mIBG scintigraphy in 20 women with breast cancer and normal LVEF who had been treated with anthracycline derivatives combined with trastuzumab or alone. The authors observed that the combined use of anthracycline and trastuzumab provided a higher frequency and intensity of cardiac adrenergic hyperactivity. ^51^ Another study identified abnormal ^123^ I-mIBG uptake in patients who used anthracycline. Additionally, ^123^ I-mIBG uptake from heart-to-mediastinum ratio decreased as the cumulative dose of the drug increased. ^52^ Those results support the hypothesis that early cardioprotection with beta-blockers may be beneficial for those patients.

#### 2.2.3. Myocardial Metabolism – ^18^ F-FDG PET-CT

Recently, Borde et al. ^53^ retrospectively analyzed myocardial uptake of ^18^ F-fluorodeoxyglucose (FDG) in patients with lymphoma treated with adriamycin-based chemotherapy. ^53^ The authors demonstrated that increased myocardial metabolic activity may be a potential marker of cell alteration preceding the CTX cascade. In that study, the increase in glucose metabolism in the heart muscle was directly proportional to the cumulative dose of doxorubicin (in mg/m^2^). Similar findings have also been suggested in patients with radiation-induced cardiac damage. ^54^ Toubert et al. ^55^ demonstrated a change in myocardial ^18^ F-FDG uptake in a patient treated with a combination of tyrosine kinase inhibitors (imatinib and sorafenib) who subsequently developed fatal HF ( Table 3 ). ^55^

**Table 3:** PET-CT studies that assessed the relationship of 18F-FDG myocardial uptake in patients undergoing chemotherapy or radiotherapy ^55^

Myocardial ^18^ F-FDG PET-CT vs. drugs: what do the studies show?	
Borde et al. ^53^	Adriamycin: increased ^18^ F-FDG uptake in the myocardium may precede a reduction in left ventricular function, and the degree of cardiotoxicity is proportional to the received dose.
Toubert et al. ^55^	Tyrosine kinase inhibitors (imatinib and sorafenib): a case report showed a change in ^18^ F-FDG myocardial uptake prior to a fatal cardiac event.
Evans et al. ^58^	Increased ^18^ F-FDG uptake in patients who received thoracic radiotherapy of > 20 Gy to > 5 cm^2^of the heart: indicator of myocardial injury.

^18^ F-FDG: ^18^ F-fluorodeoxyglucose; PET-CT: positron emission tomography–computed tomography.

Positron emission tomography with computed tomography (PET-CT) scans using ^18^ F-FDG, as well as those obtained by MRI, may be useful in the diagnosis of CTX induced by immune checkpoint inhibitors, as they allow detecting, evaluating the extent, and even quantifying the inflammatory process of several CVDs, such as myocarditis, pericarditis, and vasculitis. ^56^^-^^58^

### 2.3. Contribution from Cardiac Magnetic Resonance Imaging

#### 2.3.1. Assessment of Cardiotoxicity during Antineoplastic Treatment

Cardiac MRI is considered the gold standard for measuring LVEF and ventricular mass and volume, making it a valuable tool to assess chemotherapy-induced CTX. ^34^^,^^59^^,^^60^ LVEF may be reduced by changing one or both volumes. In general, reduced diastolic volume is related to preload status that undergoes dynamic, constant changes in a patient with cancer due to vomiting, bleeding, diarrhea, or dehydration. These are readily reversible with blood volume restoration. However, a progressive increase in stroke volume is considered a marker of chemotherapy-related myocardial injury. Another important parameter that should be monitored during chemotherapy is ventricular mass. CTX causes an initial increase in ventricular mass, probably due to induction of inflammatory response by chemotherapy, leading to an increased volume of the interstitium and cardiomyocytes. However, in later stages, usually after 6 months, there is a process of ventricular mass reduction (apoptosis, fibrosis, cardiomyocyte hypotrophy, etc.). Thus, when we are facing an increase in stroke volume and a reduction in ventricular mass, this combination is very suggestive of CTX, even if ejection fraction is preserved.

More recently, myocardial strain analysis by cardiac MRI has been gaining prominence. In the population undergoing chemotherapy, the literature highlights the potential of the test to diagnose subclinical changes. ^61^^-^^63^ Jolly et al. ^61^ studied 72 patients undergoing chemotherapy for breast cancer, sarcoma, or lymphoma with serial cardiac MRI. ^61^ There was a significant impairment in global circumferential strain 3 months after treatment compared to baseline. Moreover, global circumferential strain strongly correlated with a subclinical fall in LVEF. In a different study of 41 patients with breast cancer treated with trastuzumab, both GLS and global circumferential strain decreased during treatment and correlated with a fall in LVEF. ^62^ Cardiac MRI strain is not yet widely used in clinical practice, but studies have demonstrated that the method is potentially applicable to cardio-oncology patients.

#### 2.3.2. Cardiac Magnetic Resonance Imaging in Late Follow-up

Late evaluation with cardiac MRI provides important diagnostic and prognostic findings when complemented with tissue analysis. It is important to understand that early changes during treatment are markers of some late changes, such as presence of delayed enhancement. Myocardial edema is suggestive of an inflammatory process in the myocardium, and the greater the water content, the greater the local signal intensity. ^64^ A useful sequence in this context is triple inversion recovery, which is obtained by adding a third saturation pulse, to eliminate any adipose tissue signal (fat saturation) from the image. ^64^^,^^65^ Some studies have shown myocardial edema in cardiac MRI soon after anthracycline therapy using T2-weighted sequences. ^66^ Early increase in edema and subacute increase in fibrosis seen in mice that received anthracyclines were strongly related and are both indicators of late mortality. ^67^

Delayed enhancement imaging is a widely validated noninvasive technique to identify and quantify myocardial fibrosis using gadolinium, a paramagnetic contrast agent. Different delayed enhancement patterns are recognized in cardiac MRI, which allows for identification of different types of myocardial involvement, thus distinguishing between ischemic and nonischemic cardiomyopathies. ^68^ This is of particular importance when assessing chemotherapy-induced cardiomyopathy in individuals with preexisting cardiac conditions, such as CAD.

The prevalence of delayed enhancement in CTX is low. ^59^ Despite being the gold standard for identifying focal areas of myocardial fibrosis, delayed enhancement imaging is not suitable for assessing diffuse interstitial fibrosis, which is generally found in cardiomyopathy caused by CTX. There is no typical pattern of delayed enhancement for CTX related to anthracyclines and trastuzumab, and LVEF may fall even without delayed enhancement. ^60^^,^^69^ If present, its characteristics resemble the known patterns of nonischemic cardiomyopathies (epicardial, mesocardial, focal, and/or junctional) ( Figure 5 ). ^68^^,^^70^

**Figure 5:** Focal delayed enhancement in the left ventricular inferolateral wall of a patient with breast cancer treated with anthracycline and trastuzumab.

Myocarditis is a severe, potentially fatal complication in patients undergoing immunotherapy, with a mortality rate of approximately 40%. ^71^ It may have a higher incidence and even manifest as a fulminant condition in patients using immune checkpoint inhibitors, especially if they are in combination therapy and on average 34 days after therapy is initiated. ^72^

#### 2.3.3. Tissue Characterization by Cardiac Magnetic Resonance Imaging

##### 2.3.3.1. T2 Mapping

Cardiac MRI has the advantage of providing detailed information on cardiac remodeling, which is complementary to the traditional morphological and functional assessment of the myocardium. Consistent data suggest that edema, inflammation, expansion of connective tissue matrix, and change in regional myocardial deformation occur before myocardial dysfunction in patients undergoing cardiotoxic treatment, reinforcing the potential applicability of that imaging modality in individuals exposed to cardiotoxic therapies.

Clinical studies have recently shown that the use of cardiac MRI protocols that incorporate multiparametric T1 and T2 mapping sequences led to the detection of preclinical and early changes in CTX. ^73^^,^^74^ The detection of myocardial edema, which is usually based on increased signal intensity on T2-weighted images in the myocardium normalized to skeletal muscle values, has been successfully used for ischemic and nonischemic heart muscle disease. ^75^^-^^77^

Using sequences widely available and simpler than T2 mapping, Ferreira de Souza et al. documented a significant increase in T2 signal intensity in women treated with moderate doses of doxorubicin (240 mg/m^2^). ^74^ Several groups are currently investigating whether T2 mapping is applicable to the assessment of myocardial edema in survivors of cancer treated with antineoplastic therapies, but, to date, limited clinical data are available to warrant their use in everyday practice.

##### 2.3.3.2. T1 Mapping

Although delayed enhancement imaging accurately demonstrates the presence of scarring and replacement fibrosis, this technique is not able to provide complete and definitive data on the total amount of fibrosis in the myocardium, especially on interstitial fibrosis. ^78^ Because delayed enhancement imaging is based on relative differences in T1 signal intensity after administration of gadolinium-based contrast agents, visualization of diffuse interstitial fibrosis may be incomplete or limited by this technique.

In both retrospective and prospective studies, delayed enhancement has not been uniformly detected in patients treated with anthracycline-based chemotherapy. ^79^^,^^80^ In fact, a cardiac MRI scan with negative delayed enhancement or without areas of fibrosis does not necessarily exclude the presence of myocardial fibrosis, thus reinforcing the limitation of this method in this context. Although Fallah-Rad et al. have demonstrated the presence of subepicardial delayed enhancement in all patients who had LV dysfunction induced by trastuzumab, ^79^ other studies have found conflicting results. Lawley et al. ^81^ showed that < 10% of women treated with trastuzumab had positive delayed enhancement. ^81^

New techniques based on sequential T1 measurements performed before and after the administration of gadolinium-based contrast agents provide a more accurate assessment of myocardial extracellular volume (ECV), a marker of connective tissue matrix in the myocardium and interstitial fibrosis. Even before the introduction of T1 mapping, authors had already shown that T1 measurements were able to detect increased distribution of gadolinium in the myocardium in survivors of cancer treated with chemotherapy. ^79^ These findings suggest that the expansion of connective tissue matrix assessed by T1 mapping emerges as an early marker of CTX and is able to predict impaired functional status.

Patients previously exposed to anthracyclines have significantly higher ECV values when compared to healthy controls matched for age and sex. Jordan et al. investigated patients with and without cancer, including some treated and not treated with anthracycline as well as some treated with other chemotherapy agents. ^82^ Native T1 values (prior to contrast administration) were consistently higher before and after treatment in those with cancer compared to those without cancer. The study also demonstrated that ECV increased significantly after anthracycline therapy (30.4 ± 0.7% vs. 27.8 ± 0.7%; P < 0.01). Moreover, in multivariate statistical models, both native T1 and ECV values remained consistently higher in patients with cancer regardless of adjusting for other variables.

As recently demonstrated, quantifying intracellular water lifetime (T1) in the myocardium emerges as an innovative approach to detect myocardial remodeling following cancer treatment, providing additional information to the assessment of ECV alone. ^15^ A recent study evaluated 27 women with breast cancer before and after a cumulative doxorubicin dose of 240 mg/m^2^, including the measurement of biomarkers. From 1 to 2 years following doxorubicin use, there was a decrease in LVEF and indexed LV mass. Conversely, there was an increase in ECV and a reduction in intracellular water lifetime. ^74^

## 3. Vascular Toxicity

### 3.1. Contribution from Vascular Ultrasonography

#### 3.1.1. Venous Thromboembolism and Cancer

##### 3.1.1.1. Introduction

The association between cancer and superficial venous thrombosis or DVT was first reported by Armand Trousseau in 1865. ^83^ The relative risk of venous thromboembolism (VTE) is approximately seven times higher in patients with active cancer, and VTE may be the first manifestation of occult cancer (7% to 12% of cases), consisting of a “paraneoplastic phenomenon.” ^84^^,^^85^

Occurrence of thrombosis complicates the management of patients with cancer because of the need for anticoagulant therapy and the potential risk of bleeding. Patients with cancer and acute VTE are at a higher risk of recurrent thrombosis than those without cancer. Finally, an episode of VTE increases the mortality of patients with cancer and this may be the result of massive pulmonary embolism or tumor progression. ^86^

##### 3.1.1.2. Epidemiology

There are several associated factors that may increase the risk of thrombosis in patients with cancer, such as treatment (radiotherapy and/or chemotherapy), postoperative period, and intravascular devices. ^87^ The incidence of DVT in patients with cancer is 4.7-fold higher than in those without cancer after adjusting for comorbidities, as demonstrated in a general population in Denmark. ^88^ Two-year incidence rate ranges from 0.8% to 8.0% and is associated with high rates of recurrence and mortality. A higher incidence of VTE within 1 year of diagnosis occurs in those with advanced neoplastic disease of the brain, lung, uterus, bladder, pancreas, stomach, and kidney (4- to 13-fold higher in metastatic disease than in localized disease). Chemotherapy increases the risk of VTE by 7 times in patients with cancer.

##### 3.1.1.3. Diagnosis of Deep Venous Thrombosis

Venous ultrasonography is the standard imaging test for patients with suspected (deep and/or superficial) venous thrombosis in central vessels, abdominal vessels, or extremities (cervical spine, upper and lower limbs). In addition to confirming or discarding the presence of thrombi, it allows for a differential diagnosis against other diseases.

In clinical practice, the definition of the body segment to be examined depends on the signs and symptoms presented by the patient. In cases of suspected pulmonary embolism and no evidence of the location of the thrombi, venous ultrasound screening should prioritize the vessels in the lower limbs (site of 85% of DVT cases). If the result is negative for that site, the investigation will then include the iliac veins and the inferior vena cava, and, finally, cervical and upper limb vessels.

##### 3.1.1.4. Venous Ultrasound Protocols

It is recommended that patients with a Wells score ≥ 2 undergo venous ultrasonography (involving the compression of the entire lower limb every 2 cm from the inguinal ligament to the ankle, including the posterior tibial and peroneal veins as well as the gastrocnemius and soleus muscle veins in the calf; and color images with a spectral analysis of at least the common femoral and popliteal veins) because if only the femoropopliteal segment is examined and doubt remains, a complete examination should be repeated within 5 to 7 days to discard DVT (delaying diagnosis and increasing risks and costs). If there are symptomatic areas not included in the protocol, these must be assessed (e.g., anterior tibial veins, plantar veins, etc.). ^89^

If a complete examination cannot be promptly conducted, a two-point compression ultrasound should be initially performed until a new test with complete protocol can be carried out. It is known that the availability of this diagnostic modality is limited in smaller medical centers and outpatient clinics as well as in the evenings and weekends.

Specific examinations of the iliac veins and the inferior vena cava should be reserved for doubtful cases; rhizomelic thrombosis initiating in the upper thigh ( Figure 6 A-B ); or when spectral flow curves (pulsed-wave Doppler) in the common femoral vein have a continuous pattern (loss of phasicity), suggesting proximal involvement.

**Figure 6:** A and B – Rhizomelic edema in an adolescent with cancer and thrombotic occlusion of the right iliac vein (RIV).

##### 3.1.1.5. Differential Diagnosis of Deep Venous Thrombosis

Patients with cancer (occult or active, bedridden or not, undergoing chemotherapy or radiotherapy, using anticoagulants, with a venous catheter for drug infusion) and other comorbidities may have complications that require a differential diagnosis against venous thrombosis. The most frequent complications are unilateral or bilateral edema at any site and calf pain.

Ultrasonography, in addition to discarding the presence of thrombi in the lumen of vessels in edematous segments, allows for diagnosis of tumor masses compressing the iliac veins and causing unilateral or bilateral edema; May-Thurner syndrome and left lower limb edema; extensive cellulite; and mediastinal masses compressing the superior vena cava and causing upper limb and neck edema. As bilateral edema is rarely caused by thrombosis, systemic conditions (heart failure [HF], liver failure, kidney failure, hypothyroidism) or medication use should be discarded.

Another advantage of ultrasonography in cases of significant increase in limb volume due to tumor mass compression, extensive DVT, or large hematomas is the rapid assessment of possible impairment associated with regional arterial flow (i.e., compartment syndrome), which may lead to severe ischemia.

#### 3.1.2. Catheter-Related Thrombosis in Patients with Cancer

##### 3.1.2.1. Introduction

Long-term catheter use is frequent in patients with cancer because of the need for infusion of chemotherapy agents and intravenous administration of supportive therapies. Catheter-related thrombosis (CRT) is defined as a mural thrombus that extends from the catheter to the lumen of the vessel, leading to partial obstruction or occlusion of the catheter, with or without clinical symptoms. ^90^

Most cases of CRT occur within 100 days of catheter insertion. CRT rate is between 14% and 18% when venography or vascular ultrasound screening is performed. Of those, less than 5% of patients are symptomatic. ^91^

CRT may cause pulmonary embolism and loss of vascular access in 15% and 10% of patients, respectively, which significantly increases the costs related to treatment and management. ^92^

##### 3.1.2.2. Risk Factors

A meta-analysis that included five randomized studies of individuals with and without cancer prospectively evaluated CRT-related variables and found that the following were significant: ^93^

- Subclavian vein × upper limb insertion (odds ratio [OR] 2.16; 95% confidence interval [CI] 1.07-4.34);
- Previous history of DVT (OR 2.03; 95% CI 1.05-3.92);
- Improper catheter tip position (OR 1.92; 95% CI 1.22-3.02).

In multivariate analysis, implanted ports showed a 57% reduction in the risk of CRT when compared to peripherally implanted central venous catheters. ^93^

##### 3.1.2.3. Diagnosis and Complications

In thrombosis involving veins of the arm, axillary vein, distal subclavian vein, or jugular vein, sensitivity and specificity are very high (> 95%) ( Figure 7 ). These values, however, fall to about 55% in thrombosis involving the proximal subclavian vein, the brachiocephalic veins, and the superior vena cava. This is due to a naturally difficult approach imposed by the anatomical barrier of bone and lung in this region. An alternative approach is the use of probes with a smaller contact surface area such as pediatric and/or adult sector probes and microconvex probes. The most frequent complications of thrombi are secondary infection (bacterial colonization), pulmonary embolism, and postthrombotic syndrome. ^94^^,^^95^

**Figure 7:** Vascular ultrasound of the proximal supraclavicular region showing thrombus in the proximal right internal jugular vein (RIJV), brachiocephalic venous trunk (BCVT), and proximal right subclavian vein (RSCV). A central venous catheter (CATH) is observed in the RSCV directed towards the BCVT.

#### 3.1.3. Pulmonary Hypertension in Patients with Cancer

Pulmonary hypertension is defined as an increase in mean arterial pressure greater than 20 mm Hg detected through an invasive procedure of right heart catheterization. The most common form is due to LV involvement (World Health Organization group II).

Echocardiogram remains the first-line test in the diagnostic investigation of pulmonary hypertension; however, it is important to reinforce that etiological diagnosis of pulmonary hypertension in patients with cancer is clinically complex, as we may find:

– Masses or tumors in the left atrium (LA) causing obstruction and thereby leading to increased capillary pressure;
– Extrinsic tumor compression, as seen in cases of large thymomas, lymphomas, or fibrosing mediastinitis;
– Pulmonary thromboembolism secondary to the use of tyrosine kinase inhibitors such as dasatinib or to LV involvement (CTX, preexisting heart disease, valve diseases, etc.). ^96^

In patients with pulmonary hypertension, computed tomography (CT) has a special value for assessing pulmonary vascularization and pulmonary parenchyma, providing information on possible mechanisms and consequences of pulmonary hypertension. Pulmonary parenchymal findings on CT scans of patients with pulmonary hypertension may vary and depend on the etiology. ^97^

Noncontrast CT allows for detection of abnormalities related to pulmonary hypertension, such as enlarged pulmonary artery and dilated right chambers, and identifies conditions secondary to pulmonary disease, thus being sufficient for assessing most lung diseases. The test is indicated for assessing pulmonary parenchyma in diffuse lung diseases, such as interstitial lung diseases and chronic obstructive pulmonary disease, for detecting arteriovenous malformations, and for diagnosing veno-occlusive disease or pulmonary capillary hemangiomatosis.

Contrast CT can define the pulmonary vascular anatomy and structure, obtaining accurate angiographic images from the pulmonary artery down to the subsegmental vessels. The technique is widely available, easy to perform, and more sensitive than invasive angiography in the detection of pulmonary emboli and thus has become the standard modality for noninvasive diagnosis of acute pulmonary embolism.

In pulmonary hypertension, the RV will reflect pressure overload with hypertrophy and dilation, and may show ventricular dysfunction. Significant distortion of ventricular geometry may be present, with the RV taking on a globose shape, leading to LV compression in more advanced stages. Patients with pulmonary hypertension with RV dilation and reduced RV ejection fraction have a poorer prognosis. ^98^

Cine imaging provides an accurate assessment of ventricular morphology and function with images acquired in short-axis orientation, obtaining complete volumetric coverage of the RV, although images acquired in transaxial orientation may also be used. Several data are obtained, such as end-systolic and end-diastolic volumes, ejection fraction, cardiac output, and myocardial mass. ^99^ RV mass is measured by tracing the epicardial and endocardial borders, and ventricular mass index is usually calculated as the ratio between RV mass and LV mass.

Interventricular septal geometry is also assessed by MRI, and projection to the left during systole indicates increased pressure in the right chambers. As the pressure in the lungs increases, systolic pressure of the RV may exceed that of the LV and lead to impaired LV stroke volume. In cases of more severe pulmonary hypertension, progressive RV failure affects LV diastolic function because the interventricular septum projects also during diastole, thus compromising LV diastolic filling and further reducing cardiac output. Interventricular septal curvature is an effective measure of geometric deformation involving the ventricles, with a strong correlation between paradoxical interventricular septal curvature and severity of pulmonary hypertension. ^100^

New techniques are being implemented, such as myocardial strain analysis, phase-contrast imaging, and delayed enhancement imaging, with potential application in cases of patients with pulmonary hypertension, allowing for analysis of regional myocardial function, hemodynamic repercussions, and extent of RV impairment. ^101^

## 4. Radiotherapy-Induced Cardiotoxicity

### 4.1. Role of Echocardiography

#### 4.1.1. Epidemiology

Radiotherapy holds an important place in cancer treatment. In the United States (US), over 3 million survivors of cancer underwent radiotherapy in 2016, accounting for 29% of all survivors 5 years after the initial diagnosis. The proportion of patients with cancer undergoing thoracic radiotherapy reaches 45.6% if those with breast cancer and lymphoma are included. Several studies have demonstrated an increased incidence of CAD, AMI, and sudden cardiac death in patients undergoing radiotherapy, especially those with Hodgkin lymphoma or breast cancer, reinforcing the potential side effects of the treatment. ^102^^,^^103^

Radiation-induced heart disease (RIHD) is known as the multiple deleterious effects generated by the total cumulative dose of radiotherapy and enhanced by adjuvant chemotherapy on the cardiovascular system. The spectrum is highly variable and includes the involvement of any cardiovascular system structure, and late manifestation is much more frequent. Acute complications, usually more subtle and clinically less important, consist of myopericarditis and pericarditis. Late findings, which appear years or decades after exposure, tend to be clinically relevant and include cases of chronic pericarditis, valve diseases, medium- and large-vessel diseases (porcelain aorta and carotid stenosis), cardiomyopathies (dilated and restrictive types), conduction disorders, and CAD. ^104^ There is abundant literature demonstrating that exposure to radiation, especially of the left chest, is associated with increased cardiovascular mortality, attributed mainly to CAD and a higher risk of developing HF. ^105^^,^^106^ The most relevant risk factors for developing RIHD are shown in Table 4 .

**Table 4:** Risk factors for radiation-induced heart disease (RIHD)

RISK FACTORS FOR RIHD	
	< 50 years of age
	Cumulative radiation dose > 30 Gy
	High fractional doses of radiation > 2 Gy/day
	Presence and extent of a tumor close to the heart
	Concomitant use of chemotherapy agents (mainly anthracyclines)
	Risk factors for cardiovascular disease (hypertension, DM, dyslipidemia, smoking)
	Preexisting cardiovascular disease
	Inadequate cardiac protection
	Irradiation in the anterior region or left hemithorax
**Definition of high risk: exposure to thoracic radiotherapy in the anterior or left chest region in addition to at least one of the listed factors**	

DM: diabetes mellitus.

#### 4.1.2. Pathophysiology

Cardiomyocytes are relatively resistant to radiation damage because of their postmitotic state. However, endothelial cells remain sensitive to radiation, and the pathophysiology of most forms of RIHD seems to be related to these cells. Other mechanisms that perpetuate cellular injury also seem to be implicated, such as ischemia and tissue hypoxia, in addition to the direct injury manifested by accelerated atherosclerosis, thrombosis, oxidative stress induction, myocytolysis, and activation of neurohumoral mechanisms leading to atherosclerosis and, finally, fibrosis. ^107^

#### 4.1.3. Initial Evaluation and Follow-Up

In patients with cancer, complementary cardiovascular evaluation is classically dictated by symptom status or by suggestive findings on physical examination. Echocardiography plays a key role in the assessment of heart morphology and function and is considered the first imaging modality in most cases.

Regarding post-radiotherapy follow-up, annual clinical evaluations are recommended, including targeted history taking, clinical examination, and resting electrocardiogram. In the presence of symptoms and/or new suggestive findings on physical examination, a new transthoracic echocardiogram (TTE) is suggested. In asymptomatic patients classified as low risk for developing RIHD, TTE surveillance is recommended after 10 years of exposure with subsequent reassessments every 5 years. In asymptomatic individuals classified as high risk, however, evaluation should be anticipated, including the investigation of myocardial ischemia using a noninvasive functional test, as shown in Figure 8 . ^104^^,^^108^^,^^109^

**Figure 8:** Algorithm for echocardiographic monitoring following thoracic radiotherapy. ^104^^,^^108^^,^^109^ CAD: coronary artery disease; ECG: electrocardiogram; echo: echocardiogram; RF: risk factor; RIHD: radiation-induced heart disease; TTE: transthoracic echocardiogram.

#### 4.1.4. Radiation-Induced Heart Disease and Role of Echocardiogram

##### 4.1.4.1. Radiation-Induced Pericardial and Myocardial Disease

Pericardial disease is described as the most common side effect of thoracic radiotherapy, usually appearing a few weeks after treatment. New protocols including lower doses and implementation of cardioprotective techniques decreased the incidence from 20% to 2.5%, with a correlation proportional to the dose and volume exposed during treatment. ^103^^,^^104^ Pericarditis is typically self-limiting. However, 10% to 20% of patients develop chronic or constrictive pericarditis 5 to 10 years after treatment, which is indicative of a more reserved prognosis. ^109^ TTE provides diagnosis, allows for quantification of pericardial effusions, and serves as a guide to pericardiocentesis. A great advantage of the method is allowing for analysis of constrictive physiology, characterized by echocardiographic findings such as pericardial thickening, septal bounce, restrictive diastolic filling pattern, significant inspiratory variation in mitral inflow (> 25%), inferior vena cava plethora, reduced circumferential strain, and hepatic vein diastolic expiratory flow reversal. The impact on survival has been attributed to the concomitant finding of other radiation-associated cardiac injuries, being commonly accompanied by myocardial fibrosis, premature coronary artery stenosis, and important valve injuries.

##### 4.1.4.2. Radiation-Induced Coronary Artery Disease

Treatment of Hodgkin lymphoma and breast cancer typically includes thoracic radiotherapy, resulting in cardiac tissue exposure to radiation. The cumulative incidence of radiation-induced coronary artery disease (RICAD) is estimated to reach almost 60% in survivors of Hodgkin lymphoma, with a relative risk 3.2 times greater than that of the general population. ^109^ Regarding breast cancer, which is the most frequent cancer among women, meta-analyses showed that the relative risk of developing RICAD is higher in patients undergoing left thoracic radiotherapy. ^110^

Therefore, patients with thoracic neoplasms are at a considerably higher risk of developing RICAD compared to the general population, especially in the presence of risk factors such as the cumulative dose used (in general > 30 Gy), early age at exposure, affected tissue volume, lack of cardiac protection, and presence of preexisting traditional cardiovascular risk factors. ^104^^,^^105^^,^^109^

Although RIHD manifests late, CAD, in particular, may appear earlier, as of the fifth year after exposure. ^105^ RICAD is known to mostly exhibit ostial and proximal involvement, typically affecting the left main coronary artery, the left anterior descending artery, or the right coronary artery. ^107^^,^^111^ Radiotherapy predisposes to or accelerates atherosclerosis, even in those without classic cardiovascular risk factors, and may have sudden death as the first clinical presentation. Because of high morbidity and mortality rates and heterogeneous and sometimes atypical clinical presentation, we thus recommend performing stress TTE screening using exercise or pharmacological (dobutamine) stress whenever relevant. This noninvasive functional method offers advantages such as satisfactory accuracy, no radiation, relatively low cost, and high availability.

##### 4.1.4.3. Radiation-Induced Valve Disease

A higher prevalence of radiation-induced valve disease (RIVD) is reported in the subgroups of patients with Hodgkin lymphoma (17%) and breast cancer (4.2%). Similar to RICAD, the risk is related to total radiation dose and radiation field.

Valve involvement with hemodynamic repercussions usually occurs after 10 years of radiotherapy and most commonly affects left heart valves. The aortic valve is more frequently affected than the mitral valve. Valve regurgitation manifests earlier than stenosis, which usually appears about 20 years after radiotherapy. Echocardiographic findings are variable and may range from minimal changes without any associated valve dysfunction to diffuse fibrosis, thickening, and calcification. The involvement of any component of the valve apparatus is typical, but the commissures are spared, thus allowing for differentiation from a rheumatic etiology. Aortomitral curtain impairment (i.e., thickening and calcification) is a typical post-radiotherapy finding associated with poor long-term survival. ^103^^,^^108^

With the progression of symptoms and severity of the lesions, surgical intervention or percutaneous management may be required. Specific echocardiographic techniques such as 3D echocardiography and transesophageal echocardiography (TEE) contribute to improved anatomical and functional assessment, especially in valve lesions with greater hemodynamic and structural repercussions. However, caution is recommended when performing TEE in patients undergoing thoracic radiotherapy, given the possibility of associated esophageal impairment.

### 4.2. Radiation-Induced Arteritis – Actinic Arteritis

Radiation-induced arteritis, also known as actinic arteritis, is the result of changes in the arterial wall leading to stenosis or occlusion. These phenomena may lead to an accelerated atherosclerotic process of inflammatory etiology, secondary to endothelial cell injury, which can occur in medium- or large-sized arteries. In cases of radiotherapy for head and neck tumors, the affected arteries may be both intracranial and extracranial.

Radiation-induced carotid artery stenosis had its course investigated by Cheng et al. ^112^ The authors demonstrated that there was an annual progression of the degree of stenosis from less than 50% to more than 50% in 15.4% of irradiated patients versus 4.8% of nonirradiated patients. There was no difference in symptom onset or mortality between the study groups. However, such changes may lead to the onset of cerebrovascular symptoms, and symptomatic patients with carotid artery lesions following radiotherapy have been shown to exhibit reduced cerebral flow. ^113^

Peripheral artery disease, although rare, has also been reported following radiotherapy for the treatment of cervical cancer, with involvement of the iliac and femoral arteries. Symptoms may be those of chronic ischemia, although cases of acute arterial occlusion have been observed. ^114^

Peripheral artery disease, although comparatively rare, has also been reported following radiotherapy, e.g., for the treatment of cervical cancer, with involvement of the iliac and femoral arteries. Symptoms may be those of chronic ischemia, although cases of acute arterial occlusion have been observed. ^114^

#### 4.2.1. Diagnosis

The diagnosis of carotid artery stenosis may be clinically inferred by neck auscultation for a bruit in the irradiated region. Imaging modalities such as vascular ultrasonography, CT angiography, MRI, or conventional contrast angiography are able to detect and quantify a stenotic lesion. ^115^

Vascular ultrasonography has the advantages of being noninvasive and low-cost, using no nephrotoxic contrast, and thus being safe for medium- and long-term follow-up.

#### 4.2.2. Ultrasonographic Features

Arterial injuries induced by ionizing radiation may occur in the various arteries of the body, and have been described especially in the supraaortic vessels (i.e., the carotid and subclavian arteries). In the first year after treatment of localized head and neck cancers with radiotherapy, a process of accelerated and progressive intima-media thickening occurs in both carotid arteries, which may lead to significant luminal narrowing. ^116^ Wilbers et al. found similar results 7 years after radiotherapy was used in this region. ^117^ A 20-year follow-up study of young adults who underwent radiotherapy showed the following findings: a higher prevalence of atherosclerotic plaques in the irradiated vessels than the nonirradiated vessels (18% vs. 2%), greater intima-media thickness (IMT) in treated patients than controls, and a linear increase in IMT over time after radiotherapy ^118^ ( Figures 9 and 10 ).

**Figure 9:** Transverse plane of the right common carotid artery showing significant intima-media thickening with consequent luminal narrowing.

**Figure 10:** Patient with actinic arteritis 6 years after radiotherapy. A. Stenosis > 70% of the right common carotid artery on color Doppler image – significant luminal narrowing and turbulent flow are observed. B. Large increase in intima-media thickness of the right common carotid artery on B-mode image causing luminal narrowing. C. Pulsed-wave Doppler image showing increased flow velocity. D. Occluded left common carotid artery.

This accelerated atherosclerotic process often affects the common carotid artery, although the internal carotid artery may also be involved. Differential diagnosis using ultrasound imaging alone is not always possible, and a history of radiotherapy in the affected region is crucial for the diagnosis to be established. Chronic atherosclerotic disease and Takayasu arteritis are the main differential diagnoses. In chronic atherosclerotic disease, involvement is more common in the carotid bifurcation and the internal carotid artery. The common carotid artery may also be affected ( Figure 11 ). In actinic arteritis, involvement of the common carotid artery is more evident, with more diffuse, pronounced, and progressive atherosclerosis ^112^^,^^115^ .

**Figure 11:** Atherosclerotic plaque in the carotid bifurcation.

In Takayasu arteritis, there is a homogeneous and concentric thickening that may lead to significant stenosis and occlusion of the vessel, and there may also be dilations (aneurysms). It typically affects the common carotid artery up to the bifurcation, but spares the internal carotid ^119^ ( Figure 12 A-B ).

**Figure 12:** Takayasu arteritis in a 17-year-old female patient. A. Longitudinal plane of the left common carotid artery (LCCA) showing a significant concentric wall thickening. ^119^ B. Power Doppler image showing great luminal narrowing of the common carotid artery with preserved lumen from the carotid bifurcation.

#### 4.2.3. Arterial Stenosis Follow-Up

Because of the progressive nature of radiation-induced arterial stenoses, a more rigorous follow-up using imaging methods is suggested to detect significant but still treatable stenoses. Initially, vascular ultrasonography may be performed annually and then individualized, with shorter intervals in case of rapid disease progression as well as after treatment. ^120^

Carotid endarterectomy (ECA) and endovascular approaches (carotid angioplasty and stenting) have proven effective in the treatment of radiotherapy-induced stenosis. ^121^ The extent of the disease and a large arterial area affected may facilitate the development of embolic phenomena during stenting, with consequent cerebrovascular events. Moreover, stenting may favor intimal hyperplasia with restenosis ( Figure 13 ). The mechanism that leads to the emergence of intrastent intimal hyperplasia in those patients seems to be related to the proliferation of smooth muscle cells ^122^ Restenosis has been shown to be a problem following endovascular treatment, appearing to be greater in previously irradiated patients compared to controls. The rate of restenosis >50% is higher in patients treated with stenting compared to those treated with carotid endarterectomy. ^123^

**Figure 13:** Patient after 10 years of radiotherapy with a stent in the left common carotid artery; longitudinal color Doppler image showing intimal hyperplasia and intrastent flow; pulsed-wave Doppler image showing increased local velocity.

The treatment of involved arteries other than cervical arteries should be individualized. There are case reports of patients treated with surgery and endovascularly, with angioplasty and stenting. ^114^^,^^124^

### 4.3. Cardiovascular Evaluation after Radiotherapy and Role of Nuclear Medicine

#### 4.3.1. Coronary Artery Disease Evaluation and Follow-Up after Radiotherapy

Lind et al. ^125^ evaluated 69 women undergoing thoracic radiotherapy at doses of 46 to 50 Gy, with fractions of up to 2 Gy per session, using proper cardiac shielding. They underwent rest myocardial perfusion imaging with technetium-99m (^99m^Tc) sestamibi or^99m^Tc tetrofosmin before and at 6, 12, and 18 months following radiotherapy. Myocardial perfusion changes were observed at 6 months, especially in the left anterior descending artery. ^125^ This finding is consistent with that of Hardenbergh et al., ^126^ who showed myocardial perfusion changes in about 60% of patients 6 months after thoracic radiotherapy. ^126^ Ventricular function analysis on echocardiography showed that only one of the patients had a reduction of over 10% in LVEF. Although an association with microcirculation damage was found, the authors highlighted the importance of those changes, which may subsequently lead to diastolic and systolic dysfunction.

Both microvascular and macrovascular changes in the coronary arteries are recognized late sequelae of thoracic radiotherapy. The importance of regular coronary artery screening for effects of radiotherapy is known. However, specific screening regimens for this population are still lacking, despite a large arsenal of noninvasive methods available. ^127^^,^^128^

### 4.4. Coronary Artery Disease Evaluation after Radiotherapy – Role of Computed Tomography

Radiotherapy-induced cardiotoxicity involves several pathophysiological mechanisms that trigger various forms of harm to the cardiovascular system. In the coronary arteries, there may be a direct vasospastic effect, endothelial damage or changes in lipid metabolism and consequent premature atherosclerosis. The risk of subsequent coronary events is proportional to the radiation dose.

The high accuracy of coronary CT angiography for detection of CAD gives this method the potential to provide accurate information on suspected CAD in cancer patients, especially in those presenting with chest pain and symptoms of heart failure of potentially ischemic etiology.

Several studies have reported an increased incidence of CAD, acute myocardial infarction, and sudden cardiac death in patients undergoing radiotherapy, especially in patients with Hodgkin lymphoma or breast cancer, which highlights the potential of this treatment modality to cause harm. Current guidelines suggest screening for CAD within 5 to 10 years after irradiation of the chest to screen for late cardiotoxicity. ^129^ Multiple studies, including CT studies, have confirmed these clinical and epidemiological findings in patients with Hodgkin lymphoma. Mulrooney et al. ^130^ identified plaque on CT angiography in 39% of survivors. In a prospective study of 179 patients with a mean follow-up of 9.5 years, Girinsky et al. ^131^ found plaque in 26% of those who had undergone radiotherapy. On multivariate analysis, the radiation dose applied to the origin of the coronary arteries was associated with subsequent detection of plaque. Van Rosendael et al. ^132^ compared 71 patients undergoing radiotherapy versus 237 nonirradiated controls, matched for multiple CAD risk factors, and found a higher prevalence of plaques, more proximal disease, greater degree of stenosis, and a greater number of vessels with significant lesions in the group undergoing radiotherapy.

### 4.5. Coronary Artery Disease Evaluation after Radiotherapy – Role of Magnetic Resonance Imaging

CMR has excellent accuracy for the diagnosis of myocardial ischemia when combined with pharmacological stress testing, and is considered the gold standard for assessment of infarction areas, myocardial viability, and ventricular function, with prognostic value in predicting cardiovascular events.

The CE-MARC study compared investigation of ischemia by stress myocardial perfusion CMR versus scintigraphy in 628 patients undergoing cine coronary angiography as the gold-standard reference, in a population with a 39% baseline prevalence of obstructive CAD. ^133^ Both methods had similar specificities (CMR, 83.4%; SPECT, 82.6%, p = 0.916), but CMR was significantly more sensitive for the diagnosis of myocardial ischemia (86.5%) than SPECT (66.5%) (p < 0.0001).

The late gadolinium enhancement (LGE) technique, in which images are acquired after infusion of gadolinium contrast without the need for stressors, is considered the leading noninvasive method for detection of myocardial fibrosis. Analysis of the extent of myocardial infarction predicts myocardial viability in the segment under investigation. Assessment of the presence and extent of infarction by CMR has established prognostic value; detection of previously unknown infarcts in patients undergoing CMR is associated with worse cardiovascular prognosis and mortality. ^134^

## 5. Cardiac Tumors and Masses

### 5.1. Contribution from Echocardiography

Cardiac masses are often challenging to echocardiographic diagnosis. Thrombi, vegetations, and pseudotumors account for 75% of cases. Primary cardiac tumors (PCTs), in turn, are extremely rare with an estimated frequency of 0.02%. They may be benign or malignant in terms of histology. Cardiac metastases are up to 100 times more frequent. The World Health Organization recently updated the classification of cardiac and pericardial tumors. ^135^

Transthoracic echocardiography (TTE) assesses location, morphology, dimensions, echogenicity, mobility, extent, calcifications, type of insertion, and presence or absence of pericardial effusion. ^136^ Different Doppler imaging modalities allow for assessment of blood flow and consequent hemodynamic repercussions, such as flow obstruction or valve dysfunction. Color flow mapping reveals the presence of vascularization in some types of tumors, and the use of contrast perfusion allows for identification and differentiation of thrombi from tumors. In general, thrombi do not enhance and are well delineated in the contrasted chamber. Contrast agents also assist in the differentiation of benign from malignant tumors. ^137^ Malignant tumors tend to be completely enhanced, while benign tumors are only partially enhanced. Perfusion imaging analysis of post-impulse contrast replenishment may be qualitative using visual inspection or quantitative using time-signal intensity curves and parametric maps (color-coded signal intensity). The difference in post-impulse ( *A* ) maximal steady-state pixel intensity between the mass and the adjacent myocardium (Delta *A*_mass-myocardium_) is calculated. Reported sensitivity and specificity of Delta *A*_mass-myocardium_in differentiating thrombi from benign and malignant tumors were 93% and 100%, respectively, with a cutoff value of 23.2 dB, and were 100% and 97%, with a cutoff value of 0.45 dB. ^138^ Estimated volume, velocity, and microvascular blood flow of the masses may also be obtained. Strain imaging can differentiate the nature and contractile pattern of some types of tumors, such as fibroma and rhabdomyoma. In rhabdomyomas, deformation is opposite to that of the myocardium, while fibromas have no deformation. ^139^ TEE has greater sensitivity (> 95%) and acoustic resolution than TTE, providing a better characterization of tumors and masses in terms of location, insertion, and relationship with the vena cava, especially in atrial and valvular lesions. TEE plays an important role in cases of systemic embolization, in intraoperative assessment of tumor resection, as well as in ventricular lesions in patients with limited thoracic acoustic window. Real-time 3D echocardiogram is the latest technique for assessing cardiac masses, providing more accurate data on volume, morphology, and relationship with adjacent structures, as well as aspects such as location and type of insertion, homogeneity, vascularization, calcification, and areas of necrosis. ^140^

Any cardiac mass detected on echocardiography must be analyzed within the respective clinical and epidemiological context. Age, sex, symptoms, and concomitant oncology disease are important inputs for diagnosis. Artifacts should be excluded by analyzing multiple cut planes and adjusting the imaging parameters. Eustachian valve and prominent crista terminalis, Chiari network, and coumadin ridge are anatomic variants that may be misinterpreted as masses. Lambl excrescences are filiform structures ranging from 3 to 5 mm found at sites of valve closure, downstream of valve. They do not cause valve dysfunction but may embolize. Lipomatous hypertrophy of the interatrial septum is characterized by infiltration of mature fat, which spares the fossa ovalis resulting in a typical dumbbell shape. Calcified amorphous tumors are heterogeneous, hyperechoic, nonneoplastic masses with hypoechoic or isoechoic areas, of variable mobility, often associated with mitral annular calcification and considered to be potential sources of embolism. ^141^ Pericardial cysts are hypoechoic, usually loculated masses with varying dimensions that are adjacent to the cardiac border. Thrombi may be found in any chamber and vary in size, morphology, and mobility but do not usually occur outside predisposing conditions. When inside the LA, they are present mainly in patients who have spontaneous echo contrast, atrial fibrillation, stenosis, or mitral prosthesis. In the right atrium (RA), they are relatively common in oncology patients with a long-term catheter and a hypercoagulable state. Migrating thrombi are seen on the right side of the heart in patients with DVT and pulmonary embolism. LV thrombus is associated with changes in segmental contractility and dilated cardiomyopathy. Vegetations mainly affect the left heart valves, have great mobility, and move independently. They are found on the atrial surface of the atrioventricular valves and on the ventricular surface of the semilunar valves, often causing valve dysfunction in endocarditis.

#### 5.1.1. Benign Primary Cardiac Tumors

From 85% to 90% of all PCTs are benign. Of these, nearly 50% are myxomas. ^136^ Rhabdomyoma is the most common type in children, accounting for approximately 50% of cases, followed by fibroma and myxoma. ^142^

##### 5.1.1.1. Cardiac Myxomas

These are more frequent in patients aged from 30 to 60 years, presenting with constitutional, obstructive, or embolic symptoms. They are in the LA in 75% of cases, in the RA in 20%, and in the ventricles in 5%. Typically, they are described as oscillating polypoid masses attached to the interatrial septum through a narrow pedicle. They are heterogeneous tumors with echogenic nuclei, cystic areas, and smooth surfaces. Depending on their size, they may obstruct the flow of the atrioventricular valve. Papillary myxomas are smaller and have a stretched appearance, irregular surface, greater mobility, and greater risk of embolization. Both 2D and 3D TEE and the use of contrast perfusion assist in the characterization of those tumors. Cardiac myxomas may be recurrent when associated with Carney complex. In such cases, TTE is the optimal imaging technique for follow-up. ^143^

##### 5.1.1.2. Papillary Fibroelastomas

These are small (usually < 10mm) sessile or pedunculated, rounded, gelatinous, avascular tumors with multiple narrow, elongated, branched papillary projections. They resemble sea anemones when placed under water. These characteristics provide them with friability and predispose them to adherent thrombi. ^144^ Embolization is more common in tumors larger than 10 mm and may be due to adherent thrombi or fragmented papillae. They are usually single, mobile, and attached to any endocardial surface by a short pedicle. They affect the endocardial surface of valve in 77% of cases and occur mainly in older adults. They are commonly seen in the middle section of the left heart valve leaflets. They usually project into the arterial lumen in the aortic valve and into the atrial surface in the mitral valve. ^144^^,^^145^ On echocardiogram, they exhibit independent mobility, stippled and sparkling edges, and a narrow central pedicle. They usually cause no valve dysfunction. TEE plays an important role in the assessment of those tumors.

##### 5.1.1.3. Rhabdomyomas

These are usually diagnosed in the first year of life or during fetal life. They are associated with tuberous sclerosis, are usually multiple, and tend to regress spontaneously. ^146^ They mainly affect the interventricular septum, ventricular walls, and atrioventricular valves. They appear as multiple nodular or pedunculated, lobulated, well-defined, homogeneous masses that are hyperechogenic to the surrounding myocardium. They show deformation opposite to that of the myocardium on strain imaging. ^139^

##### 5.1.1.4. Cardiac Fibromas

These rarely occur in adults. Approximately 3% to 5% of cases are associated with Gorlin syndrome (also called nevoid basal cell carcinoma syndrome). ^147^ They are characterized as a single intramural, hyperechogenic, well-defined, noncontractile solid mass with calcifications that extend into the LV chamber. They may cause arrhythmia, ventricular dysfunction, and flow obstruction. They show no deformation on strain imaging. ^139^

##### 5.1.1.5. Cardiac Lipomas

These are masses of encapsulated adipose tissue that are found mainly in the LV subendocardium but also occur in other chambers, the pericardium, and cardiac valves, reaching several centimeters in size. They are broad-based, homogeneous, immobile tumors without calcification. They are hyperechoic in the chamber and hypoechoic in the pericardium. ^148^

##### 5.1.1.6. Teratomas

These are germ cell tumors found in infants, children, and fetuses. ^147^ They may be mature or immature and rarely occur in adults. They are preferably located in the pericardial space and may cause cardiac tamponade or compression of adjacent structures. ^149^ They are characterized as heterogeneous masses containing solid, cystic areas and calcifications, often accompanied by pericardial effusion.

##### 5.1.1.7. Cardiac Hemangiomas

These vascular tumors are rarely found and may occur at any age, including fetal life. They are located mainly in the LV and RA, measuring between 2 and 10 cm in most cases. They are pedunculated and mobile in half of the cases. They appear as well circumscribed masses that may be cystic or solid, unilobulated or multilobulated, depending on their type, i.e., cavernous, capillary, or arteriovenous. ^150^

##### 5.1.1.8. Cardiac Paragangliomas

These are very rare neuroendocrine tumors. They are associated with several syndromes, such as Von Hippel-Lindau disease, neurofibromatosis type I, and Carney complex. They may be benign or malignant and hormonally active or inactive. They are extremely vascularized and nonencapsulated. They occur most often between the 4^th^and 5^th^decades of life and are located mainly in the pericardial space, next to the atrioventricular groove, at the root of the great vessels, and in the LA. They are characterized as broad-based, usually ovoid, well-demarcated echogenic masses with an average size of 5cm, surrounding the coronary artery and possibly compressing adjacent structures. ^151^

##### 5.1.1.9. Cardiac Schwannomas

These are extremely rare slow-growing tumors with only 25 cases reported in the English literature (18 intracardiac and 7 intrapericardial tumors). They arise from the cardiac plexus or branches of the vagus nerve and involve the right side of the heart more often. They are benign but may exhibit malignant behavior. They are echogenic, hypodense, nonpedunculated, demarcated masses that may reach several centimeters in size and cause signs and symptoms related to the compression of adjacent structures. ^152^

#### 5.1.2. Malignant Primary Cardiac Tumors

These are extremely rare tumors whose frequency is 0.008%. From 10% to 15% of all PCTs are malignant. They are represented by different types of sarcomas, lymphomas, and mesotheliomas. They mainly affect adults in the fifth decade of life. Sarcomas account for 65% to 95% of cases in adults and 70% in children and adolescents (< 18 years). ^143^^,^^154^ Angiosarcoma is the most common type. Usually it produces symptoms such as dyspnea and pleuritic chest pain. ^155^

##### 5.1.2.1. Angiosarcomas

These exhibit an aggressive behavior and may metastasize. They are preferably located in the RA. They present as voluminous, homogeneous, immobile, nonpedunculated, stalkless, lobulated, echogenic masses with a broad base of endocardial insertion and smooth intracardiac borders. Because they are located next to the atrioventricular groove, angiosarcomas usually cause obstructions in the tricuspid valve and, in most cases, affect the pericardium causing hemorrhagic effusions. Partly because of their slender vascular channels, they do not significantly enhance with the use of contrast perfusion. The sensitivity of TTE for identification is 75%. ^155^

###### 5.1.2.1.1. Undifferentiated Sarcomas

These are typically located in the LA and may mimic myxomas. They tend to involve the mitral valve causing flow obstruction. They have a broad base, some mobility, and a smooth and homogeneous appearance. ^156^

###### 5.1.2.1.2. Rhabdomyosarcomas

These are the most common type in children. They usually arise from the ventricular walls and often interfere with valve function because of intracavitary growth. ^148^ They tend to occur in more than one location causing obstruction at multiple levels. They grow rapidly and usually invade the pericardium. ^136^

###### 5.1.2.1.3. Leiomyosarcomas

These arise most commonly from vessels such as the inferior vena cava and pulmonary artery but may also occur in the LA.

##### 5.1.2.2. Primary Cardiac Lymphomas

These account for approximately 27% of malignant PCTs, and diffuse large B-cell lymphoma is the most common type. ^153^ They predominate in the 6th decade of life but also occur between the 1st and 9th decades, and are frequently seen in immunocompromised patients. They are located mainly on the right side of the heart and in the pericardium. They involve the superior vena cava and the interatrial septum in 25% and 48% of cases, respectively. They appear as infiltrating or nodular masses. Infiltrating forms are homogeneous and lead to wall thickening and restrictive hemodynamics. Nodular forms intrude into the chambers and may compromise the atrioventricular node, right coronary artery, and pericardium. ^136^ More than one chamber is involved in 75% of cases. Depending on the location and dimensions, they may cause inflow obstruction, superior vena cava syndrome, arrhythmias, atrioventricular block, restrictive or constrictive syndromes, pericardial effusion, and embolic phenomena. Both 2D and 3D TEE and the use of contrast perfusion are useful for the characterization of those tumors. ^157^

##### 5.1.2.3. Primary Malignant Pericardial Mesotheliomas

These account for 8% of malignant PCTs and approximately 50% of primary pericardial tumors. ^153^^,^^158^ They are more frequent between the 5th and 7th decades of life and show low survival. In many cases, they are associated with exposure to asbestos and radiation. ^159^ Symptoms are vague and include constrictive pericarditis and pericardial effusion with cardiac tamponade. Myocardial infiltration may occur and lead to conduction abnormalities. TTE has low sensitivity for detection. ^158^

#### 5.1.3. Metastatic Cardiac Tumors

These are by definition malignant, and their incidence has increased in recent decades, reaching 18.35% in patients with advanced cancer. ^159^^,^^160^ They predominate between the 6^th^and 7^th^decade of life. Cardiac metastases may occur by direct extension of the tumor, hematogenous spread, lymphatic spread, or intracavitary extension from the inferior vena cava or pulmonary veins. With the exception of central nervous system tumors, any malignant tumor may spread to the heart, but carcinomas of the lung, breast, and esophagus, melanoma, and hematologic neoplasms (leukemia and lymphoma) are responsible for most cases. ^159^^,^^161^ Malignant melanomas are the most likely tumors to affect the heart. Pericardium, epicardium, myocardium, and endocardium are, in descending order of frequency, the most affected sites. If intracavitary, they are preferably located in the right chambers. Pericardial effusions are frequent and may be asymptomatic. Cardiac involvement should be suspected in all patients with cancer who develop new cardiovascular symptoms. ^162^ TTE is the initial assessment method for detecting tumor thrombi in the inferior vena cava and cardiac chambers as well as pericardial effusion. Other imaging modalities are required to better characterize the lesions, including their extent and relationship with adjacent structures, as well as to reveal the primary tumor site. ^161^

### 5.2. Contribution from Cardiac Magnetic Resonance Imaging

Cardiac MRI, mainly because of the property of tissue characterization, has been established as a robust method in the assessment of cardiac masses, especially in differentiating benign from malignant tumors. In addition to tissue characterization, cardiac MRI will provide important information regarding location, dimensions, extent, and signs of compression or obstruction of cardiac structures. ^163^

Steady-state free precession MRI sequences are used to assess the morphology and mobility of the mass and its relationship with the myocardium, as well as whether the mass is causing hemodynamic obstruction to the valve structures. Pre-contrast T1- and T2-weighted black-blood sequences assist in defining the relationship with the adjacent anatomy, size, extent, and characterization of the mass. Signal intensity of the mass on MRI depends on T1 and T2 relaxation time. The signal intensity should be evaluated in relation to that of the myocardium, i.e., whether the mass signal is isointense, hypointense, or hyperintense to the myocardial signal. ^164^

Fat saturation allows for identification of a fat signal in the composition of the mass, as this is hyperintense on T1- and T2-weighted sequences; saturation of the signal suggests a fatty component in the mass. This technique can detect benign structures such as lipomatous infiltration of the interatrial septum, benign tumors such as lipoma, and the presence of fat as a component of a liposarcoma.

Vascularization of the tumor may be assessed using first-pass perfusion imaging for presence or absence of perfusion and whether this is isoperfused, hypoperfused, or hyperperfused in relation to the LV myocardium, while delayed enhancement detects the presence of fibrosis in the mass composition. ^165^

The cardiac MRI protocol for assessing a cardiac or pericardial mass should be initiated with high-resolution axial slices, followed by cine sequences for location and analysis of cardiac function and hemodynamic repercussions. Then, according to the diagnostic suspicion, slices are made over the mass for proper tissue characterization. T1- and T2-weighted sequences may be used, as well as fat suppression sequences to assess the lipid component of the suspected mass, first-pass perfusion with gadolinium to assess vascularization, and delayed enhancement sequences to assess fibrosis.

If a thrombus is suspected, delayed enhancement sequences with a long inversion time may also be used to confirm the diagnosis. Cardiac MRI allows for differentiation of thrombus from tumor, as there is no perfusion in the thrombus by the paramagnetic contrast agent. The thrombus appears very dark on delayed enhancement sequences, even with a prolonged inversion time of up to 600 ms, and an adjacent hyperintense area may be observed by the surrounding blood. A slight delayed enhancement may be present in very organized, old thrombi.

#### 5.2.1. Benign Primary Tumors

##### 5.2.1.1. Myxomas

Pre-contrast findings may be explained by their histology: high signal intensity on T2-weighted sequences is related to the myxoid matrix with a high content of water and polysaccharides, while low signal intensity is related to areas of hemorrhage, hemosiderin (degradation of hemoglobin), calcifications, and surface thrombi. Myxomas are isointense on T1-weighted images and hyperintense on T2-weighted images ( Figure 14 ). A slight enhancement on first-pass perfusion imaging was observed in 16% to 66% of cases. Delayed enhancement has a variable and heterogeneous pattern, with a more diffuse distribution (> 50% of the tumor – more frequent) and a less intense pattern (< 50% of the tumor). ^166^ From 10% to 20% of myxomas have calcifications and some may have thrombi on their surface.

**Figure 14:** Myxoma in the left atrium.

##### 5.2.1.2. Lipomas

These arise from the subendocardial layer in 50% of cases and from the mesocardial or epicardial layers in the other 50%. They may determine hemodynamic changes, depending on their size. They show a homogeneous signal similar to that of fatty tissue: hyperintense on T1-weighted images and with signal dropout on fat saturation sequences and isointense or hyperintense on T2-weighted images. ^167^ There is no first-pass perfusion or delayed enhancement on contrasted sequences ^168^ ( Figure 15 ).

**Figure 15:** Lipoma in the right atrium. A. Four-chamber cine image of a large lipoma in the right atrium. B. T1-weighted image showing that the lipoma is hyperintense. C. No local delayed enhancement.

##### 5.2.1.3. Papillary Fibroelastomas

On cine imaging, they present as a hypointense and extremely mobile mass, without necessarily causing a functional impact on the valve. Assessing tissue characteristics of those tumors is difficult because of their small size and great mobility, but intermediate signal intensity on T1- and high signal intensity on T2-weighted images can be observed, with no first-pass perfusion or high signal intensity on delayed enhancement images ^167^ ( Figure 16 ).

**Figure 16:** Papillary fibroelastoma in the mitral valve. A. Four-chamber cine image of a hypointense mass. B. T1-weighted image showing intermediate signal intensity. C. Early late gadolinium enhancement sequence showing a slight local enhancement.

##### 5.2.1.4. Rhabdomyomas

These are slightly hyperintense on T1-weighted images and hyperintense on T2-weighted images. They show minimal or even no enhancement with gadolinium ( Figure 17 ).

**Figure 17:** Rhabdomyoma in the left ventricle: A. Short-axis cine image of an intramural mass in the interventricular septum. B. T2-weighted image showing high signal intensity. C. Delayed enhancement sequence showing a slight local enhancement.

##### 5.2.1.5. Fibromas

These are usually isointense or hypointense on T1-weighted images and homogeneously hypointense on T2-weighted images. In general, they do not appear on first-pass perfusion imaging because of their low vascularity. On delayed enhancement imaging, fibromas are intensely and homogeneously enhanced, although sometimes the lesions may have a central component of low signal intensity that may be associated with local calcification ^169^ ( Figure 18 ).

**Figure 18:** Intramural fibroma. A. Four-chamber cine image of an intramural tumor on the lateral wall. B. T2-weighted image of a hypointense mass in relation to the myocardium. C and D. Short- and long-axis delayed enhancement image showing homogeneously high signal intensity of the tumor compatible with fibrous tissue.

##### 5.2.1.6. Hemangiomas

These are heterogeneously isointense or hyperintense on T1- weighted images and usually hyperintense on T2-weighted images, and heterogeneous areas of low signal intensity may also occur. The delayed enhancement pattern is typically heterogeneous and hyperintense with a marked first-pass perfusion. ^168^^,^^169^

#### 5.2.2. Malignant Tumors

Malignant tumors tend to be larger, are more frequently perfused with gadolinium, and have a higher prevalence of delayed enhancement. ^170^

##### 5.2.2.1. Sarcomas

Most sarcomas have characteristics suggestive of malignancy, such as a heterogeneous signal due to necrosis and internal hemorrhage, nodular or irregular shape, ill-defined borders, infiltration of the myocardium and adjacent structures, extension to the pericardium, and associated pericardial effusion. Angiosarcoma accounts for 40% of cardiac sarcomas and is mostly located in the RA with infiltration of the atrial wall and the pericardium associated with pericardial effusion ( Figure 19 ). On cardiac MRI, it is shown as a large heterogeneous lobular mass that is isointense or hyperintense on T1-weighted images, hyperintense on T2-weighted images, perfused with gadolinium on first-pass images, and heterogeneously hyperintense on delayed enhancement images ( Figure 20 ). ^147^^,^^169^

**Figure 19:** Angiosarcoma in the right atrium. A. Four-chamber cine image of a sarcoma arising from the posterior wall of the right atrium and invading the walls, the basal portion of the right ventricle, and the pericardium. B. Delayed enhancement image of a heterogeneously hyperintense mass on upper axial plane.

**Figure 20:** Rhabdomyosarcoma in the right ventricle. A. Four-chamber cine image of a large multilobulated intracavitary mass in the right ventricle. B. Delayed enhancement image showing a heterogeneously hyperintense mass.

##### 5.2.2.2. Lymphomas

These are isointense or hypointense on T1-weighted images, isointense or hyperintense on T2-weighted images, and heterogeneously enhanced on delayed enhancement images.

### 5.3. Contribution from Nuclear Medicine

#### 5.3.1. ^18^ F-FDG PET-CT

Scanning with ^18^ F-FDG PET-CT remains seldom used in cardiac tumors, especially malignant PCTs. In secondary malignant cardiac tumors (metastases), which are most frequent, ^18^ F-FDG PET-CT is an established technique. ^171^

A case series conducted by Rahbar et al. ^172^ evaluated 24 consecutive patients with cardiac tumors using ^18^ F-FDG PET-CT scanning before treatment. ^172^ The patients were divided according to histological subtype of the tumors, obtained by surgical resection or biopsy, into: benign (n = 7), primary malignant (n = 8), or secondary malignant (n = 9). Tumor ^18^ F-FDG uptake was observed between groups. Then, to assess sensitivity and specificity of the method in differentiating benign from malignant tumors, they were divided into malignant (n = 17) or benign (n = 7) only. ^18^ F-FDG uptake was quantified according to the maximum standardized uptake value (SUV_max_) of 3D volumes covering the tumor mass. In case of low tumor ^18^ F-FDG uptake, CT images were used to identify and obtain tumor volumes. Additionally, because of physiological myocardial ^18^ F-FDG uptake, uptake in the normal myocardium and blood pool were measured and compared with SUV_max_of the tumors. They found that ^18^ F-FDG uptake was low in the blood pool and normal myocardium, and significantly high in the primary malignant tumors compared to the benign ones. Secondary malignant tumors showed an uptake comparable to that of primary malignant tumors but with a considerably higher SUV_max_variation. The mean SUV_max_was 2.8 ± 0.6 in the benign cardiac tumor group. Such tumors usually show no positive contrast to the normal myocardium and are only seen on morphologic CT images. In contrast, SUV_max_of primary malignant tumors is 2.5 times greater than that of benign tumors. Among metastatic cardiac tumors, SUV_max_was 10.8 ± 4.9 with a significant variation in ^18^ F-FDG uptake from 3.4 to 16.7. In primary malignant tumors, ^18^ F-FDG uptake was greater than that of benign tumors and similar to that of secondary malignant tumors. ^172^

##### 5.3.1.1. Cutoff SUV_max_value for ^18^ F-FDG to better differentiate benign from malignant cardiac tumors

Different cutoff SUV_max_values have been suggested for determination of malignancy using ^18^ F-FDG PET-CT scanning. Rahbar et al. ^172^ demonstrated that a value of 3.5 reached a sensitivity of 100%, a specificity of 86%, a positive predictive value of 94%, and a negative predictive value of 100%. ^172^ When they increased this value to 4.6, they found a specificity of 100%, a sensitivity of 94%, and a positive predictive value of 100% for malignancy. The main limitations of that study were the retrospective nature, the lack of a diagnostic pattern for cardiac tumors, and the heterogeneity of the results. Also, several myxomas were not included in the analysis because they were diagnosed on echocardiogram and cardiac MRI.

## 6. Special Situations

### 6.1. Carcinoid Heart Disease

Neuroendocrine tumors are rare neoplasms (2.5 to 5 cases per 100,000 population) that may occur anywhere but often involve the gastrointestinal tract (carcinoid tumors). ^173^ In about 30% to 40% of cases, mostly in the small intestine and proximal colon, patients experience vasomotor changes (hypotension, hypertension, flushing), diarrhea, and bronchospasm. This is called carcinoid syndrome, and it is usually associated with hepatic metastasis. ^174^ The presence of biomarkers such as N-terminal pro-B-type natriuretic peptide (NT-proBNP), chromogranin A (CgA), and 5-hydroxyindoleacetic acid (5-HIAA) is useful both in diagnosis and prognosis of carcinoid disease. ^175^

Carcinoid heart disease (CHD), manifested by plaque formation, thickening, and endocardial fibrosis, is probably related to chronic exposure to 5-HIAA and preferably affects the right chambers and the tricuspid and pulmonary valves. Impairment of left chambers (15% of cases) and mitral and aortic valves is seen in the presence of a right-to-left shunt (e.g., patent foramen ovale) or a bronchial carcinoid tumor. ^176^ Early diagnosis and monitoring of disease progression have a dramatic impact on prognosis and long-term survival (mean survival of 1.6 years vs. 4.6 years in patients without CHD), as early surgical intervention is essential for a successful management.

Echocardiography is considered the gold standard for the diagnosis and monitoring of CHD. ^173^ The assessment of right heart chambers (atrium and ventricle), RV function, thickening, leaflet mobility, and presence of valve regurgitation or stenosis (individually analyzed) should be performed on initial clinical suspicion, in case of a new bruit, new symptoms, or every 3 to 6 months according to CHD severity ( Figure 21 ).

**Figure 21:** Patient with a carcinoid tumor, liver metastasis, and clinical status of right heart failure. RV: right ventricle, RA: right atrium, TR: tricuspid regurgitation, IVC: inferior vena cava.

Tricuspid valve involvement leading to regurgitation is the most frequent alteration, followed by pulmonary regurgitation, tricuspid stenosis and, finally, pulmonary stenosis. In the tricuspid valve, there is main involvement of the anterior and septal leaflets, and the posterior leaflet is relatively preserved. RV dilation and dysfunction are consequences of the severity of the valve lesions. Cardiac metastases (4% of cases) may be identified on echocardiography but are more visible on MRI. ^173^^,^^176^

Several echocardiographic scores have been proposed for CHD assessment. Among the simplest ones, Westberg et al. ^177^ ( Table 5 ) evaluates only tricuspid valve regurgitation and anatomy. A score > 1 is considered pathological (87% accuracy). A score > 4 has a 3-year survival rate < 45% vs. 75% for a score of 0. ^177^

**Table 5:** Westberg score ^171^

Characteristics	Severity and scoring				
Valve thickening	Normal 0	Mild 1	Moderate 2	Severe 3	Severe with valve retraction 4
Regurgitation	Normal 0	Mild 1	Moderate 2	Significant 3	Extreme 4

More complex scores provide a greater amount of information and are useful for surgical monitoring and planning. In Bhattacharyya et al. ^173^ ( Table 6 ), a score > 8 has a diagnostic accuracy of 96% for CHD, and a 5-point increase in the score is an independent predictor of CHD progression (RR 2.95) and mortality (RR 2.66), according to Dobson et al. ^176^^,^^178^

**Table 6:** Bhattacharyya score ^167^

Characteristics	Severity and scoring			
Valve thickening	< 3 mm 0	≥ 3 and < 4 mm 1	≥ 4 and < 5 mm 2	≥ 5 mm 3
Valve mobility	Normal 0	Mild 1	Moderate 2	Severe/fixed 3
Valve morphology	Normal 0	Rectified 1	Slight retraction 2	Moderate/severe retraction 3
Valve stenosis	Normal 0	Mild 1	Moderate 2	Significant 3
Valve regurgitation	Normal 0	Mild 1	Moderate 2	Significant 3
Right ventricle diameter	Normal 0	Slight increase 1	Moderate increase 2	Significant increase 3
Right ventricle function	Normal 0	Slight reduction 1	Moderate reduction 2	Significant reduction 3

Surgical cardiac intervention is indicated in case of RV dilation and dysfunction and signs of HF refractory to drug treatment. Closure of the patent foramen ovale, aiming to reduce right-to-left shunt, and removal of metastases are recommended during the approach. ^175^

### 6.2. Cardiac Amyloidosis

#### 6.2.1. Introduction

Amyloidosis is a multisystemic disease arising from the deposition of compound proteinaceous material in the extracellular space that is difficult to diagnose and, once cardiac involvement is proven, has a poor prognosis. According to the type of precursor protein, it can be classified as light-chain (AL) amyloidosis (when the deposits originate from light-chain proteins) and transthyretin (ATTR) amyloidosis (when the protein carries thyroxine and retinol). ^179^

#### 6.2.2. Clinical Types and Cardiac Involvement

Besides the heart, AL amyloidosis may affect several organs such as the kidneys, the gastrointestinal tract, and the autonomic nervous system. It is caused by the deposition of proteins deriving from immunoglobulin light chains produced by plasma cells in cases of plasma dyscrasia, such as in multiple myeloma. Approximately 10% of patients with multiple myeloma may develop AL amyloidosis, and more than half of them may have cardiac amyloidosis. Cardiac involvement is second to kidney involvement in AL amyloidosis. ^180^^,^^181^

The pathological mechanism of ATTR amyloidosis is the deposition of transthyretin derivatives produced by the liver. It is further divided into two subtypes: a) wild-type ATTR (ATTRwt), with predominant involvement of the heart and often causing carpal tunnel syndrome, biceps tendon rupture, and spinal stenosis; and b) mutant ATTR (ATTRm), associated with specific substitutions of transthyretin-coding genes that may affect, besides the heart, the autonomic and peripheral nervous systems. ^182^

#### 6.2.3. Contribution from Echocardiography

TTE is the most widely used diagnostic option when cardiac amyloidosis is suspected, being also used to screen for cases in family members. It is highly available in medical services of variable complexity, has a relatively low cost, and allows for serial repetition with good reproducibility and consistent findings. Cardiac involvement has the poorest prognosis in this group of patients.

##### 6.2.3.1. Increased Myocardial Thickness

The myocardium has a bright or speckled granular appearance, which is more visible on conventional imaging without harmonics. The increase in myocardial thickness is often concentric and symmetrical, unlike that of hypertrophic cardiomyopathy, which tends to show greater asymmetry in distribution ( Figure 22 ). The presence of increased myocardial thickness and low-voltage waves on electrocardiogram is a finding that leads to suspected cardiac amyloidosis. ^183^ The term "increased myocardial thickness" must be distinguished from secondary myocardial hypertrophy: there is myocellular hypertrophy in the latter, while in the former it is the extracellular space that is increased by the deposition of amyloid protein material. ^184^ The increase in myocardial thickness is diffuse and may include all LV walls, RV free wall, interatrial septum, and valves with or without associated regurgitation. Myocardial thickness ≥ 12 mm may be used as a cutoff value for suspected cardiac amyloidosis. ^185^ The clinical value and prognosis of increased myocardial thickness are already known: the more exuberant, the greater the occurrence of congestive HF and the poorer the survival.

**Figure 22:** Patient with left ventricular hypertrophy with no determined cause. Echocardiogram showing shiny or granular scintillating aspect of the myocardium suggestive of cardiac amyloidosis. PE: pericardial effusion; LA: left atrium; LV: left ventricle; RV: right ventricle; RA: right atrium.

##### 6.2.3.2. Left Atrium

There may also be changes in the LA, and the increase in LA volume may be due not only to high degrees of diastolic dysfunction but also to the deposition of amyloid fibrils.

Changes in atrial myocardial deformation (strain and strain rate) and in atrial systolic function parameters are frequently observed in patients with few echocardiographic characteristics related to cardiac amyloidosis and in those with congestive HF symptoms, which are associated with a poor prognosis. ^186^

##### 6.2.3.3. Diastolic Function

In more advanced stages, the assessment of diastolic function usually reveals increased E/e' ratio compatible with increased LV filling pressures. However, diastolic function parameters are abnormal even in the early stages of cardiac amyloidosis. The presence of diastolic dysfunction is associated with poor clinical outcomes, as already demonstrated, and suggests greater risk of thromboembolism. ^187^^-^^189^

##### 6.3.3.4. Left Ventricular Systolic Function

LVEF may still be within normal limits, and this feature allows for cardiac amyloidosis to be included as a cause of HF with preserved ejection fraction. Deteriorated systolic function occurs in later stages and is associated with poor clinical outcomes and prognosis. ^187^^,^^189^

Regarding myocardial deformation measurements, cardiac amyloidosis exhibits a reduction in myocardial GLS mainly in the basal and middle segments of the LV with preserved values in the apical segment (i.e., apical sparing). ^188^^,^^190^ It is useful to calculate the ratio of the mean apical GLS values by the sum of mean GLS values in the basal and middle LV segments. A ratio > 1 means a high specificity for diagnosis of cardiac amyloidosis. ^190^ When considering the relationship between LVEF measurements and myocardial strain values (referred to as ejection fraction-to-strain ratio [EFSR]), there is an inversion of the ratio with lower values of myocardial deformation and preserved LVEF, regardless of clear congestive HF symptoms. An EFSR ≥ 4 is useful in differentiating cardiac amyloidosis from hypertrophic cardiomyopathy, with 89.7% sensitivity and 91.7% specificity. ^187^^,^^188^

In cardiac amyloidosis, isolated myocardial GLS values > -15% have been associated with greater severity and increased mortality. ^191^ In another study, a strain value > -17% managed to separate groups of patients with unfavorable clinical response after autologous bone marrow transplantation (BMT). An apical sparing index ≥ 1.19 was associated with higher mortality and need for heart transplantation at 5 years of follow-up. This index also showed a predictive value in the survival curves and the need for heart transplantation when associated with a lower LVEF value. ^192^

Liu et al. ^193^ analyzed the value of the diastolic wave peak of the strain rate curve alone (rapid diastolic filling phase) in 41 patients with cardiac amyloidosis, demonstrating that the global value of the diastolic strain rate (global LSR_dias_> - 0.85 S^−1^) was predictive of a 4-fold increase in mortality in patients with cardiac amyloidosis with preserved LVEF (LVEF > 50%). ^193^

Strain measurements are highly sensitive and exhibit good specificity in cases of cardiac amyloidosis; however, it should be noted that conventional echocardiographic measurements of systolic function are still important and demonstrate excellent prognostic correlation in such cases. Thus, stroke volume index (SVi) and the measure that associates stroke volume with total myocardial mass volume (referred to as myocardial contraction fraction [MCF]) showed a good prognostic value in patients with cardiac amyloidosis, regardless of type and comparable to that of strain measurements. Therefore, in this group of patients, SVi < 33 mL/min and MCF < 34% with cardiac index < 2.4 L/min/m^2^were the best predictors of overall survival with an accuracy comparable to that of strain measurements ^194^ ( Figure 23 ).

**Figure 23:** Global longitudinal strain of the left ventricle in a patient with transthyretin amyloidosis, demonstrating a peculiar pattern that preserves the apex and majorly affects the middle and basal segments.

##### 6.2.3.5. Other Findings

Despite not having a notable incidence in cases of cardiac amyloidosis, the presence of intracardiac thrombi has been reported and, of note, is not necessarily associated with the presence of atrial fibrillation. ^195^ Some authors have demonstrated that AL amyloidosis and diastolic dysfunction are independent risk factors for intracardiac thrombi. ^196^ The search for thrombi should always be considered in patients who have criteria for severe cardiac amyloidosis (e.g., high degrees of increased myocardial thickness, decreased LVEF, and advanced stages of diastolic dysfunction). Several mechanisms have already been proposed to explain the occurrence of thrombi in addition to the presence of atrial fibrillation, such as: amyloid deposits in the subendocardial region that produce damage and greater parietal stiffness; hemodynamic stasis as a consequence of important diastolic dysfunction, usually of the restrictive type; and plasma hypercoagulability as a systemic mechanism resulting from various conditions.

Cardiac tamponade due to major pericardial effusion is rare, and diagnosis is predominantly clinical, as the high pressures in the right chambers decrease the chances of classic echocardiographic collapse. Less intense pericardial effusion is more common. Heart valves may also be infiltrated but rarely exhibit severe valve regurgitation. ^187^

##### 6.2.3.6. Diagnostic Approach

In several clinical situations, the diagnosis of cardiac amyloidosis is initiated with an echocardiographic examination but very often occurs in one out of two contexts of cardiac evaluation.

The first situation concerns the need for cardiac evaluation in patients with multiple myeloma. In such cases, confirmation of cardiac involvement is imperative, as the survival rate in AL amyloidosis may be extremely unfavorable. Even if the classic echocardiographic findings are absent in the first examination, it is recommended that the examination is repeated in a follow-up visit.

In the second situation, patients undergo cardiac evaluation because they have either clinical manifestations of HF with preserved ejection fraction or “hypertrophy” on echocardiogram (often associated with low-voltage complexes on electrocardiogram).

In such cases, in addition to the echocardiographic findings described above, investigation should continue with other imaging tests (MRI and NM imaging) and laboratory tests such as protein electrophoresis and light-chain protein testing.

In centers where myocardial deformation analysis is available, the presence of apical sparing is typical but not exclusive. Other causes of LV hypertrophy may exhibit the same pattern, such as hypertrophy secondary to hypertension and aortic stenosis.

#### 6.2.4. Contribution from Cardiac Magnetic Resonance Imaging

Cardiac MRI allows for diagnosis of cardiac amyloidosis by morphological and functional changes on cine sequences, as is the case with TTE, and more accurately through delayed enhancement and T1 map sequences.

A delayed enhancement sequence usually detects the deposition of amyloid protein in the myocardial interstitial space through the presence of hyperenhancement (white color) against the usually black muscle on the images. ^197^ Syed et al. ^198^ analyzed cardiac MRIs of 120 patients with amyloidosis, ^198^ of which 35 had confirmed cardiac involvement by histology. The other 85 patients were divided into those with or without echocardiographic evidence of cardiac amyloidosis. In the 35 patients with histological diagnosis, 97% showed delayed enhancement and 91% had increased wall thickness on echocardiography. Transmural or subendocardial delayed enhancement was the most common pattern (83%), associated with greater interstitial amyloid deposition; focal delayed enhancement (6%) and difficulty in canceling the myocardial signal (8%) were other patterns associated with cardiac involvement. In the 85 patients without histological diagnosis, delayed enhancement was present in 86% of those with echocardiographic abnormalities and in 47% of those without evidence of cardiac amyloidosis on TTE. The presence and pattern of delayed enhancement were associated with the New York Heart Association (NYHA) functional class, low voltage on electrocardiogram, LV mass index, RV wall thickness, and serum troponin and type B natriuretic peptide (BNP) values. ^198^

Austin et al. ^199^ evaluated 47 patients with suspected cardiac amyloidosis who underwent cardiac MRI, electrocardiogram, TTE, and biopsy. ^199^ Compared to biopsy, delayed enhancement had a sensitivity of 88%, a specificity of 90%, a positive predictive value of 88%, and a negative predictive value of 90%, thus being the only parameter with significant diagnostic accuracy in multivariate analysis. One year after biopsy, 19% of patients died, and delayed enhancement was the only significant predictor of mortality in this period, showing the prognostic role of this parameter in amyloidosis.

Regarding the differentiation of types of cardiac amyloidosis, cardiac MRI is useful in predicting AL and ATTR amyloidosis. Dungu et al. ^200^ showed a greater increase in myocardial thickness (228 g vs. 167 g) and a greater extent of delayed enhancement in patients with ATTR amyloidosis, although mortality in patients with AL amyloidosis was higher. In that study, 90% of patients with ATTR amyloidosis had some segment with transmural delayed enhancement compared to 37% of patients with AL amyloidosis. In addition, 100% of patients had delayed RV enhancement in ATTR amyloidosis compared to 72% in AL amyloidosis. The authors created a score called Query Amyloid Late Enhancement (QALE) with values ranging from 0 to 18. Values ≥ 13 can predict ATTR instead of AL amyloidosis, with a sensitivity of 82% and a specificity of 76%.

T1 mapping sequence, the most recent MRI technique, is useful in the diagnosis and prognosis of patients with cardiac amyloidosis. It may also be used to monitor the treatment of new therapeutic agents in this disease. Pre-contrast T1 values are increased in patients with cardiac amyloidosis; this is a diagnostic option for patients without delayed enhancement or those who do not undergo the delayed enhancement sequence because of contraindication to gadolinium injection. In addition, the gadolinium injection makes it possible to calculate myocardial ECV, which is expanded in cardiac amyloidosis by the deposition of amyloid fibrils in the interstitium, showing a good correlation with biopsy, with proven prognostic value. ^201^^,^^202^

#### 6.2.5. Contribution from Nuclear Medicine

NM has gained new relevance in the diagnosis of cardiac amyloidosis, with studies showing that radiotracers with affinity for bone, such as 3,3-diphosphono-1,2-propanedicarboxylic acid (DPD) and pyrophosphate, both labeled with technetium-99m [^99m^Tc], have very high sensitivity for the detection of ATTR. Scintigraphy with those tracers allows for noninvasive differentiation of amyloid protein subtypes based on the concentration levels of the tracer in the cardiac area. This differentiation has prognostic and therapeutic implications. ^203^ Gilmore et al. ^204^ have conducted the most important study on the subject to date. They evaluated the results of scintigraphy with bone-affine radiotracers in 1,217 patients with suspected cardiac amyloidosis referred for evaluation in specialty centers, and obtained sensitivity values of up to 99% when AL amyloidosis had already been ruled out by biochemical testing. ^204^ The examination technique consists of injecting the tracer into a peripheral vein and then acquiring planar images of the chest in the anterior projection 1 hour post radiopharmaceutical administration ( Figure 24 ). This allows for a quantitative analysis to be performed in which two mirrored regions of interest (ROIs) are drawn, one over the cardiac area in the left hemithorax and another in the right hemithorax. A ratio between left and right hemithorax counts (representing the amount of tracer present in each area) > 1.5 has a high diagnostic sensitivity (95% sensitivity and 79% specificity), ^203^ as well as prognostic value; ratios > 1.6 are associated with poor prognosis on 5-year follow-up. After 3 hours, whole-body images are acquired in the anterior, posterior, oblique, and left lateral projections. The level of concentration of the tracer in the cardiac area is compared to that of the ribcage and graded on a scale of 0 to 3, in which grade 0 denotes no cardiac concentration; grade 1, slight cardiac concentration, lower than that of the ribcage; grade 2, equal to that of the ribcage; and grade 3, greater than that of the ribcage. Grades 2 and 3 are strongly associated with ATTR amyloidosis (> 99% sensitivity and 86% specificity) ( Figure 25 ) if AL disease has already been ruled out. Grades 0 and 1 may be related to AL amyloidosis ( Figure 26 ) or early-stage familial ATTR amyloidosis. Three-hour imaging has greater diagnostic specificity for ATTR amyloidosis (58% sensitivity and 100% specificity). ^205^^,^^206^ Three-hour single-photon emission computed tomography (SPECT) of the chest has been frequently used because it improves the ability to distinguish the presence of activity in the LV (blood pool) and assess the interventricular septum, which is the usual site of myocardial biopsy if this procedure is necessary. This additional scan also allows for a more accurate quantitative comparison of the myocardial concentration of radiotracer to that of the ribcage. Figure 27 shows RV involvement by familial ATTR amyloidosis, best evaluated on SPECT images. Technetium-99m-labeled methylene diphosphonate (^99m^Tc MDP) is the most widely available bone-affine radiotracer in Brazil, but its use for assessment of cardiac amyloidosis is discouraged. A study compared^99m^Tc MDP and DPD in a group of patients with familial amyloidosis and found that MDP performed suboptimally, with occasional false-negative findings. ^207^

**Figure 24:** Anterior image of the chest 1 hour after intravenous administration of technetium-99m-labeled pyrophosphate. Each of the circles represents a region of interest (ROI) and allows for quantification of tracer concentration. The ratio between left and right hemithorax counts, in this case, was 1.75, therefore positive for transthyretin amyloidosis.

**Figure 25:** Whole-body scintigraphy and planar chest images with technetium-99m-labeled pyrophosphate show abnormal radiopharmaceutical accumulation projecting from the heart (arrows) of a patient with advanced heart failure with preserved ejection fraction. Serum light-chain measurements were negative, and abdominal fat biopsy was positive for amyloidosis. The definitive diagnosis was transthyretin amyloidosis.

**Figure 26:** Whole-body scintigraphy and planar chest images with technetium-99m-labeled pyrophosphate showing light-intensity accumulation (grade 1) projecting from the heart (arrow) of a patient with subsequent confirmation of light-chain amyloidosis due to a monoclonal spike in serum and urine electrophoresis, increased serum free light-chain antibodies, and bone marrow aspirate showing plasma cell infiltrate < 10%. Final confirmation was performed by abdominal fat biopsy, which was positive for amyloidosis.

**Figure 27:** Reconstructed single-photon emission computed tomography scans of the axial, coronal, and sagittal axes showing accumulation of technetium-99m-labeled pyrophosphate in the left ventricle and simultaneous involvement of the right ventricle (arrows) in a patient with suspected mutant transthyretin amyloidosis, confirmed by a genetic test.

Figure 28 shows an algorithm for NM diagnosis of suspected cases of cardiac amyloidosis.

**Figure 28:** Proposed nuclear medicine diagnostic sequence for suspected cases of cardiac amyloidosis (CA). TTE: transthoracic echocardiogram; MRI: magnetic resonance imaging; TTR: transthyretin amyloidosis (TTRm: mutant TTR; TTRwt: wild-type TTR); AL: light-chain amyloidosis; PYP: pyrophosphate; DPD: 3,3-diphosphono-1,2-propanodicarboxylic acid;^99m^Tc: technetium-99m.

### 6.3. Takotsubo Syndrome

Takotsubo syndrome (TTS) is characterized by acute and reversible LV dysfunction with a behavior similar to that of acute coronary syndrome, usually with abrupt onset of precordial pain and dyspnea. It is a stress-induced cardiac syndrome without any evidence of obstructive CAD that usually resolves spontaneously within days or weeks.

In recent years, there has been a growing interest in studying the relationship between cancer and TTS. This happened especially after some published studies showed a strong association between the two conditions. ^208^

The prevalence of neoplasms seems to be higher in patients with TTS when compared to individuals of the same age and sex, both at the time of diagnosis and at follow-up. While the diagnosis of TTS is around 1% to 2% in the general population, it reaches 10% in individuals with malignancies. ^209^

In most cases, TTS involves apical and middle LV segments, which are akinetic or dyskinetic (apical ballooning pattern), as opposed to basal segments, which are generally hyperkinetic. Some forms with a variant pattern have been described such as the midventricular and inverted variants. The midventricular variant is characterized by akinesia of the midventricular segments, with hypokinesia or normal contraction of the apical segments and hypercontractility of the base. The inverted variant has two different forms: the first is characterized by apical sparing, presenting with hypokinesia of the remaining walls, and the second by hypokinesia limited to the basal segments.

Speckle tracking echocardiography allows for the assessment of multidirectional LV deformation. In typical cases (apical ballooning pattern), there is a compromise of apical and midventricular longitudinal deformation, with a base-to-apex strain gradient. In this phase, all components of the LV torsion mechanics involving the systolic and diastolic phases are compromised. Given its greater sensitivity in detecting subtle abnormalities compared to that of more traditional parameters, such as LVEF and wall motility index, speckle tracking echocardiography is useful in the diagnosis of TTS. ^210^

Three-dimensional echocardiography enabled a more accurate demonstration of LV volumes and LVEF compared to 2D echocardiography and angiography. However, further studies using this technology during the course of TTS are still required to establish its role in clinical practice.

The most prevalent tumors among patients with TTS are colorectal, breast, and bronchial malignancies and melanoma. ^208^ Hematologic neoplasms are less prevalent. Regarding potential triggering factors, a relationship with cancer treatment was identified in 57% of cases, including surgical stress (33%), chemotherapy (17%), and radiotherapy (7%); the others were emotional stress (30%) and the presence of another acute illness (13%). ^209^

Patients with malignancies have a reduced tolerance threshold for stressors and an increased sensitivity of cardiac adrenergic receptors. In this context, the addition of physical (pain secondary to cancer, diagnostic procedures, cancer surgery) and emotional (fear of illness or death, changes in family dynamics) stressors, which are common to the disease, contributes to a greater predisposition to TTS. Some malignancies, such as pheochromocytoma and paraganglioma, cause hypercatecholaminemia and may be a trigger of TTS. ^211^

### 6.4. Siderotic Cardiomyopathy (Iron Overload)

The prevalence of anemia in patients with cancer is high, and the condition is found in more than half of patients during the course of the disease, especially in those with hematologic neoplasms. Moderate-to-severe anemia occurs in approximately 40% of patients, and the cause is multifactorial; it may be secondary to treatment-related myelosuppression, occult bleeding, functional iron deficiency, erythropoietin deficiency due to kidney disease, and/or marrow involvement with tumor. ^212^ The main oncologic disease leading to iron overload is myelodysplastic syndrome, due to both increased absorption and regular transfusions. Consequently, patients or survivors of cancer may experience iron overload in the liver, heart, and adrenal gland. Patients with cardiac iron overload usually have poorer cardiovascular prognosis compared to the general population, with outcomes present in 73.2% versus 54.5% over a 3-year period. ^213^

Iron overload cardiomyopathy in early stages commonly presents with normal ventricular function. Echocardiographic diagnosis is often challenging, but some findings may suggest a diagnosis compatible with clinical history. Ventricular diastolic dysfunction may progress to restrictive physiology, leading to dilated heart disease with reduced LVEF. It may manifest as pericardial constriction due to iron deposition in the pericardium. Speckle tracking still has a conflicting role in this context, but increased cardiac iron overload may be associated with a reduced absolute GLS value. There is no description in the literature of a strain pattern that is specific to myocardial iron overload. The results of studies that attempted to correlate circumferential and radial strain with iron deposits were conflicting. Three-dimensional speckle tracking echocardiography also has no established role in these patients but has been showing favorable results for the detection of early subclinical dysfunction. ^214^^-^^216^

Although echocardiography may be used to screen for iron overload, the method is not able to accurately predict myocardial iron content, unlike cardiac MRI ( Table 7 ).

**Table 7:** Classification of myocardial and hepatic iron overload (MIO/HIO) by magnetic resonance imaging

T2* (ms) 1.5 T	R2* (Hz) 1.5 T	T2* (ms) 3.0 T	R2* (Hz) 3.0 T	MIO/HIO (mg/g DW)	Classification
Heart					
> 20	≤ 50	> 12.6	≤ 79	≤ 1.16	Normal
10 to 20	50 to 100	5.8 to 12.6	79 to 172	1.16 to 2.71	Slight/moderate overload
< 10	> 100	< 5.8	> 172	> 2.71	Severe overload
Liver					
> 15.4	≤ 65	> 8.4	≤ 119	≤ 2.0	Normal
4.5 to 15.4	66 to 224	2.3 to 8.4	120 to 435	2.0 to 7.0	Mild
2.1 to 4.5	225 to 475	1.05 to 2.3	436 to 952	7.0 to 15	Moderate
< 2.1	> 475	< 1.05	> 952	> 15	Severe

DW: dry weight.

Because of this limitation, current guidelines of several international societies for different diseases recommend routine use of MRI to determine and monitor the level of iron overload in different organs. MRI is recommended for patients with cancer with detected serum ferritin levels > 1,000 ng/mL or for patients with a life expectancy > 12 months who received > 10 transfusion bags. ^217^^,^^218^

The MRI scan should evaluate both liver and heart, and be repeated annually or according to therapeutic changes in the iron chelator or transfusion load. The quantification is made by the indirect effect that the iron molecules exert on the local magnetic field in the tissue under analysis. Therefore, the higher the concentration of tissue iron, the lower the signal of the measured tissue (darker); thus, iron quantification in proportion to signal intensity is obtained from several images with consecutive modifications of echo time ( Figure 29 ).

**Figure 29:** Example of patients with cancer with normal myocardial iron levels (A) and significant iron overload (B). T2* in patient A was 22.8 ms with a myocardial iron concentration (MIC) of 0.98 mg/g; in patient B, T2* was 11.9 ms with an MIC of 2.2 mg/g. More myocardial darkening is observed in patient B with increased echo time values, visually determining the highest degree of myocardial overload. Patient B was initiated on oral iron chelation therapy after myocardial overload was determined by magnetic resonance imaging.

Currently, the MRI scan has no specific substitute, being the only test capable of quantitatively determining the levels of iron overload in different organs simultaneously, noninvasively, and without any contrast or ionizing radiation.

## 7. Pericardial Diseases

### 7.1. Pericardial Tumors

Although usually asymptomatic, pericardial malignancy may manifest as nonspecific symptoms such as dyspnea and tachycardia, which are often related to pericarditis, pericardial constriction, myocardial invasion, or, more commonly, pericardial effusion (with or without tamponade). Tamponade occurs in patients with major effusion and no previous symptoms in up to one third of cases. ^219^

In 20% to 50% of patients with cancer and pericardial effusion, the latter is caused by neoplastic involvement; in 30%, it may be due to lymphatic impairment secondary to radiotherapy or viral infection. In 10% to 25% of cases, pericardial effusion is the first sign of cancer. ^220^

The tumors may be either primary pericardial neoplasms or metastases involving the pericardium (secondary pericardial neoplasm). The dissemination may occur through hematogenic spread, lymphatic spread, or direct invasion of the lung and mediastinum. The most common metastatic tumors involving the pericardium are lymphomas, melanomas, and lung, breast, and renal cell carcinomas; adenocarcinoma is the most common histological pattern. ^221^

A recent study showed that the presence of occult hematologic, colorectal, ovarian, kidney, pancreas, breast, and bladder neoplasms should be suspected in older adults, obese patients, and/or smokers who present with acute pericarditis and need for hospitalization. The cancer diagnosis was usually made 3 to 12 months following the episode of pericarditis. ^222^

#### 7.1.1. Echocardiogram in Patients with Pericardial Neoplasm

Pericardial effusion may be assessed semiquantitatively by TTE. Rapid volume increase and presence of spontaneous contrast on TTE, with or without identification of pericardial mass, are suggestive of pericardial neoplasm. It usually has a solid appearance, increased echogenicity, and may infiltrate the myocardium. ^220^ As a differential diagnosis, pericardial fat has low echogenicity and may be seen adjacent to the RV free wall and atrioventricular junction.

Among the primary tumors, mesothelioma, arising from the mesothelial cells of visceral or parietal pericardium, presents with pericardial effusion, tamponade, or constriction, and the nodules in the pericardium may invade the myocardium. A retrospective study of 64 patients with primary malignant pericardial mesothelioma showed that echocardiographic presentations were nonspecific, with pericardial effusion in 86% of cases (of which 67% were massive and 95% were bloody), pericardial masses in 36%, and thickening in 17%. Tamponade and constrictive pericarditis occurred in 37% and 27% of cases, respectively. Tamponade may be associated with the proliferation of diffuse mesothelial cells and myocardial infiltration, which may cause decreased relaxation and complacency. Reaccumulation of effusion following pericardiocentesis occurred in 73% of cases. ^223^

The measurement of pericardial thickness is limited on echocardiography, except when the measures are greater than 5 mm. In general, the measurement of pericardial thickness is best performed by CT or cardiac MRI, which allow for a better evaluation of the implant and tumor extent. Thus, the use of multimodality imaging shows incremental value. Speckle tracking, in turn, can detect subclinical myocardial dysfunction because of early myocardial infiltration.

Usually, pericardial fluid should only be seen on systole, but accumulation makes it present during the entire cardiac cycle. Pericardial effusion is considered significant in the presence of at least 2cm of pericardial fluid around the heart. In cardiac tamponade, the heart is compressed by the accumulation of fluid in the pericardial space, which leads to increased intrapericardial pressure that exceeds the intracavitary pressure. Such accumulation may occur rapidly or gradually. The consequent collapse of the right heart chambers, secondary to increased intrapericardial pressure, is more prominent on expiration, when RA and RV filling is reduced. However, in the presence of pulmonary hypertension, RA collapse is delayed, resisting until the intrapericardial pressure exceeds the high pressures in the right chambers. In cases of massive effusions, the heart tends to “swing” inside the pericardial fluid at each heartbeat and may have an electrical alternation on the electrocardiographic record. There are also respiratory changes in cardiac flows. The pressure increase inside the RV during inspiration leads to a deviation of the interventricular septum to the left and a consequent increase in the flow and volume of the right chambers, in addition to dilation of the inferior vena cava ( Figure 30 A-B ).

**Figure 30:** A. Tricuspid valve flow variation > 40%. B. Dilated inferior vena cava (IVC) with no caliber variation with respiratory maneuvers (patient with breast cancer).

Subxiphoid incision is the gold standard for surgical approach in biopsy or pericardial drainage. However, pericardiocentesis is routinely guided by fluoroscopy or echocardiography, with a complication rate of 4% to 10%. During echocardiography-guided pericardiocentesis, agitated saline is useful to assess the position of the needle before inserting the catheter and then to assess the position of the catheter. ^224^ Pericardial effusion is considered to be recurrent if reaccumulation of fluid occurs within 3 months of drainage.

## 8. Cardio-Oncology in Children and Adolescents

### 8.1. General Considerations

The number of new cases of childhood and adolescent cancer expected in Brazil, for each year of the 2020-2022 triennium, is of 4,310 new cases in males and 4,150 in females. These figures correspond to an estimated risk of 137 new cases per million in males and 139 per million in females. Childhood and adolescent cancer consists of a set of diseases with their own characteristics in relation to histopathology and clinical behavior. The predominant types of pediatric cancer (aged 0 to 19 years) are leukemia (28%), central nervous system neoplasms (26%), and lymphomas (8%), followed by tumors of the peripheral nervous system (neuroblastomas), Wilms’ tumor, retinoblastoma, germ cell tumors (ovaries and testes), soft tissue sarcomas, and osteosarcoma. ^225^

The prospects for childhood and adolescent cancer cure are promising, with current survival rates approaching 80% in high-quality institutions if diagnosed early. ^226^ Despite successful treatment, complications inherent to chemotherapy drugs and/or radiotherapy compromise several organ systems in the short, medium, or long term. There is clear evidence that survivors of cancer treated during childhood with anthracyclines and/or radiotherapy are significantly more likely to develop cardiovascular complications during their lifetime, estimated at 5% to 30%, especially considering the protocol used and certain risk factors. The cumulative incidence of severe chronic problems may reach 40% in the 30-year follow-up period for survivors. ^227^

Cancer is the second most common cause of death among children aged 1 to 14 years in the US, second only to accidents and violent deaths. In Brazil, cancer accounted for 8% of the total deaths among children and adolescents aged 1 to 19 years and 12% of deaths in the age range of 1 to 14 years between 2009 and 2013. In 2014, 2,724 deaths from childhood and adolescent cancer occurred in Brazil. ^225^ This reality demonstrates how important the interaction between hematologist-oncologists and cardiologists is for safety in the treatment of this type of cancer.

Cardiovascular complications are the main causes of morbidity and mortality in survivors of cancer treated during childhood, second only to disease recurrences and secondary neoplasms. The mechanisms of action of the various chemotherapy agents as well as the physical injuries caused by radiotherapy are comparable among adults, children, and adolescents. However, the younger age group is particularly more vulnerable in view of the ongoing process of growth and physical development. In addition, cardiomyocytes have a limited regeneration capacity, and the drug metabolism in this population behaves differently from that of adults. ^228^ Therefore, injuries considered to be minor at the time of treatment may progressively compromise myocardial function over time, leading to decompensation in adulthood. Inadequate gain in LV mass, decreased contractile function, and possible development of progressive restrictive cardiomyopathy over the years lead to cardiovascular sequelae that are often irreversible in some of these patients.

In addition to ventricular myocardial dysfunction, other complications can result from the use of chemotherapy agents: arterial endothelial injuries (related to the effects of systemic and pulmonary hypertension), impaired coronary blood flow, venous endothelial injuries (related to thrombotic effects), valve dysfunction, arrhythmias, and pericardial diseases. ^229^

Pericardial impairment may be directly related to the neoplasm, may result from the action of some chemotherapy agents, or may be secondary to cardiac metastasis. Pericarditis, with or without stroke, may or may not be associated with myocarditis. Constrictive pericarditis is more commonly associated with radiotherapy-induced CTX, and it is necessary to differentiate it from restrictive myocarditis. Cardiovascular imaging examinations are essential to elucidate these changes, especially in subclinical stages.

Anthracyclines (doxorubicin, daunorubicin, epirubicin, idarubicin, and mitoxantrone) are extremely potent chemotherapy agents widely used in most childhood cancer treatment protocols. There is a strong relationship between the cumulative dose of anthracyclines and the risk of HF, and in some cases there may be a long latency period between exposure to chemotherapy and the onset of cardiovascular symptoms. ^227^ Myocardial injury is progressive and usually irreversible, culminating in severe cardiac dysfunction. There are several mechanisms by which the cardiotoxic action of anthracyclines may occur. The most common ones are the formation of cytoplasmic free radicals, resulting in oxidative stress in myocytes, and a direct action on the nucleus, in which anthracyclines act on topoisomerase 2 (Top2) by preventing the replication of DNA that induces cell death. Although the Top2α fraction, which is the focus of anticancer treatment, is expressed in tumor cells, the Top2β fraction is expressed in cardiomyocytes, thus allowing a similar toxic action. ^230^ Current hematology-oncology treatment protocols follow a trend toward the use of lower doses of anthracyclines. However, even at doses that are considered low, some pediatric patients have cardiotoxic reactions during the course of treatment and/or late follow-up, so that some authors believe that there is no safe dose. ^231^ Individual susceptibility is not yet fully elucidated, but there are hypotheses that some genetic characteristics increase vulnerability and early occurrence of myocardial dysfunction. Some preventive actions are recommended, especially in patients stratified as being at high risk of developing cardiovascular complications. The primary strategy, specifically against the cardiotoxic effects of anthracyclines, is given by infusion of dexrazoxane or by using liposomal doxorubicin (not commonly used in young patients). The secondary strategy, represented by the administration of drugs with hemodynamic effects, such as beta-blockers (in particular carvedilol), angiotensin-converting enzyme inhibitors, and spironolactone, has been considered an adjuvant in the prevention or treatment of established lesions, whose prospective studies in children are still ongoing. ^228^

Other potentially cardiotoxic substances are used to treat neoplasms in children and adolescents, often in combination with anthracyclines. CTX is not uncommon with the use of alkylating agents, such as cyclophosphamide, cisplatin, and ifosfamide, with the most frequent presentations being myocarditis, arrhythmias, and HF. The most common cardiovascular complication of antimetabolites, such as 5-fluorouracil, is myocardial ischemia, reported mainly in adults. Biological agents such as tyrosine kinase inhibitors can cause QT interval prolongation, HF, hypertension, and AMI. Etoposide is a synthetic podophyllotoxin approved for the treatment of some neoplasms such as Ewing's sarcoma and lymphomas. Cardiovascular complications are rare, but chest pain, angina, and AMI have been reported in adults receiving etoposide in combination with bleomycin, cisplatin, and ifosfamide. Biological agents such as interleukins and interferon may cause hypotension or arrhythmias. The main cardiovascular effect of vinca alkaloids, such as vincristine and vinblastine, is myocardial ischemia due to coronary vasospasm. Immunotherapy has also been important in the treatment of childhood and adolescent cancer, with the potential to trigger myocarditis, although rare (< 1%), and pericarditis. ^232^^,^^233^

Radiotherapy is a therapeutic method often used in pediatric cancer treatment (about 40% of protocols). Cardiac complications resulting from radiotherapy are due to inflammation and fibrosis of cardiac structures, which may involve the pericardium, myocardium, heart valves, and coronary arteries. The pericardium is the most frequently affected structure. Although the lesions may remain asymptomatic for 5 to 15 years, they are usually progressive. Lesion severity is proportional to the dose, irradiated volume, and the combination with chemotherapy agents, which confers greater risk. Advances in radiotherapy techniques in the past two decades have allowed a reduction in the volume and doses of radiation in the cardiac area. With the emergence of 3D planning techniques, the demarcation of adjacent structures, dose adjustments, and field modifications have allowed a significant reduction in the doses applied to organs at risk, such as the heart. ^227^^,^^234^

### 8.2. Main Risk Factors for the Development of Cardiotoxicity in Children and Adolescents

**Cumulative dose**: even at relatively low doses, anthracyclines have been reported to trigger CTX. The concept of a safe anthracycline dose has been disregarded by some authors, as observed in a study that showed a 30% increase in subclinical echocardiographic abnormalities 13 years after leukemia treatment, even with cumulative doses of anthracyclines ranging from 180 to 240mg/m^2^. ^234^^,^^235^

**Combination of chemotherapy agents**: concomitant administration of more than one chemotherapy agent known to be cardiotoxic not only facilitates but also potentiates adverse cardiovascular effects.

**Age at treatment**: children treated before 5 years of age are more likely to have cardiovascular complications in the short or long term, especially due to progressive myocardial functional limitation resulting from the demands of physical growth.

**Sex**: girls have a 2-fold higher incidence of CTX than boys, probably due to a higher percentage of body fat that allows longer exposure time and body concentration of anthracyclines.

**Infusion rate**: some studies in adults suggested a reduction in the prevalence of CTX by continuous infusion of anthracyclines, compared to bolus administration. This mode of infusion, however, was not cardioprotective in the long term in the pediatric population. ^231^

**Mediastinal and neuroaxis radiotherapy**: radiotherapy doses ≥ 30 Gy or the combination of anthracycline with radiotherapy, even at low doses, may also aggravate the risk of developing cardiovascular complications. Exposure to radiation at a young age is also an important factor. These patients may progress with valve complications, pericardial complications, restrictive cardiomyopathy, CAD, and arrhythmias that can induce sudden cardiac death. ^227^^,^^234^

**Radiotherapy of the central nervous system**: in the long term, children who received cranial irradiation showed greater loss of ventricular mass, a fact attributed to the impairment of the pituitary gland with consequent secondary deficiency in the production of insulin-like growth factor 1, a precursor of growth hormone. ^236^

**Comorbidities**: the presence of two or more comorbidities increases cardiovascular risk in adults surviving cancer treatment in the pediatric age. The development of hypertension, DM, dyslipidemia, and CAD, especially in association with obesity and smoking, increases the risk of early onset of complications when compared to their siblings. The presence of liver dysfunction, renal dysfunction, and electrolyte disturbances compromises drug clearance, slows down its elimination, and increases drug exposure time.

**Genetic variants**: individual predisposition to CTX has been reported in several studies. Some genetic variants, such as RARG and UGT1A6, increase Top2 or slow down drug metabolism. The incidence of myocardial dysfunction is increased 9-fold in patients with C282Y mutation for hereditary hemochromatosis. People with trisomy 21 are more likely to develop acute myeloid leukemia and more sensitive to the toxic effects of chemotherapy agents. Familial (hypertrophic, arrhythmogenic) cardiomyopathies and patients with congenital heart defects may have a more vulnerable myocardium. ^237^^,^^238^

### 8.3. Cardiac Monitoring during Treatment

The method of cardiovascular imaging tests, definitions, and parameters used in the evaluation of children and adolescents or survivors of cancer treatment at pediatric age is the same method that is used in the adult population.

Echocardiography is the main imaging test indicated for monitoring those patients, as it provides information on the main parameters of diastolic and systolic function of both ventricles, anatomical and functional status of the valve structures and the pericardium, in addition to investigating masses, thrombi, and vegetations. The ventricular myocardial function is quantified using several parameters and methods. The most recommended ones are LVEF using the Simpson biplane method and myocardial strain analysis using the speckle tracking technique. Myocardial deformation measurements are obtained using 2D and 3D echocardiography and then analyzed on longitudinal, radial, or circumferential modes, as they do not depend on the angle of ultrasound incidence. This method has been the subject of several studies that seek to detect early subclinical changes that may be predictors of CTX in children and adolescents. ^239^ The longitudinal mode has been the one with best reproducibility used in the vast majority of studies. Despite its advantages (availability, accessibility, and low cost), echocardiography has some limitations, especially interobserver variability and compromised quality of the images obtained because of inadequate acoustic windows. Another limitation is related to ejection fraction, which has been proven to be of low sensitivity in the context of early detection of myocardial functional changes. In contrast, myocardial strain analysis using speckle tracking allows for the identification of subclinical lesions that precede the decrease in LVEF. ^239^^,^^240^

Cardiac MRI is another important diagnostic test in childhood and adolescent cardio-oncology. In addition to being the gold standard for LVEF quantification, it can detect early interstitial edema and myocardial fibrosis using contemporary methods associated with the use of gadolinium contrast by T1 mapping and delayed enhancement identification. Also, it plays an important role in the characterization of intracardiac and extracardiac masses. However, because of intrinsic characteristics, it has limitations for routine use.

The use of NM imaging in the pediatric area requires, in addition to technical knowledge, a joint assessment with the pediatric team on risk versus benefit of the exposure of children and adolescents to radiation doses (although low, based on radiation safety principles). ^241^ Radionuclide ventriculography, a noninvasive technique that uses^99m^Tc-labeled red blood cells as a radiopharmaceutical, allows for analysis of several ventricular function parameters (time activity curve, phase analysis, and amplitude), providing a refined assessment of the regions with better contractile performance (amplitude) and the time point when this contraction occurred (phase analysis), detecting the presence of interventricular and left intraventricular contractile dyssynchrony. However, information on diastolic function is limited. The timing for performing radionuclide ventriculography during cancer treatment to minimize the confounding variables is debatable. The suggestion is to perform it at least 3 weeks after anthracycline therapy, when patients have no fever and hemoglobin above 9 g/dL. ^242^ Regarding other NM imaging methods used to detect CTX such as myocardial uptake of ^123^ I-mIBG and PET-CT, there are still no robust data for indication in the pediatric population.

The diagnosis of CTX using cardiovascular imaging methods is based on the changes detected in the baseline examination. Thus, it is recommended that the functional assessment is conducted before the start of cancer treatment and then during the course of treatment for comparison purposes, making an effort to always use the same equipment, the same methods, and the same variables used in previous assessments.

Table 8 shows the international recommendations for echocardiographic monitoring of patients during and after treatment with anthracyclines (main representatives of CTX in children and adolescents).

**Table 8:** Echocardiographic monitoring of patients treated with anthracyclines in childhood

CD (kg/m^2^)	During treatment		After treatment	
	No PRF	At least 1 PRF	No PRF	At least 1 PRF
Baseline	Yes	Yes	N/A	N/A
< 200	– Clinical criterion	– Every 2/3 cycles	– 1 month after treatment – Evaluate in 1, 2, and 5 years – Individualize if required	– 1 month after treatment – Evaluate in 1, 2, and 5 years
200 to 300	– Clinical criterion	– Every 2/3 cycles	– 1, 6, and 12 months after treatment – Then every 2 years (asymptomatic)	– 1, 6, and 12 months after treatment – Then annually – Individualize
300 to 450	– Clinical criterion – Individualize if required	– Every 2 cycles	– 1, 6, and 12 months after treatment – Then annually – Individualize	– 1 month after treatment – Then every 6 months – Individualize if required
> 450	– Halfway through treatment – Individualize if required	– Every 2 cycles – Individualize if required	– 1 month after treatment – Then every 6 months – Individualize if required	

CD: cumulative dose; PRF: preexisting risk factor; N/A: not applicable.

Each patient's hemodynamic status must be considered at the time of assessment. Whenever possible, the status should be stable. The frailty of the pediatric patient in the face of certain situations, such as changes in blood volume (dehydration, hyperhydration), anemia, fever, hypothermia, shock, sepsis, abnormal heart rate, and changes in rhythm, may occasionally influence the interpretation of results. These conditions affect preload and afterload and can promote a variation of 5% to 10% in stroke volume per cardiac cycle, influencing the ejection fraction. Changes in global and segmental contractility during chemotherapy may be related to secondary cardiomyopathy induced by transient stress (idiopathic cause, sepsis, neurological injury, catecholamine release). ^243^ Functional changes due to clinical complications may be transient or definitive. The recommendation is to reevaluate the patient soon after compensation.

The definition of subclinical CTX remains challenging for children and adolescents. Some studies have reported segmental changes even before the fall in GLS, which has suggested the greater accuracy of this method for early detection of myocardial dysfunction. Current international recommendations consider a 15% fall in GLS from baseline as indicative of subclinical dysfunction, provided that hemodynamic conditions are comparatively similar. ^239^

### 8.4. Long Term Follow-up of Survivors

The risk of developing cardiovascular complications and the severity of lesions increase over time. Studies have shown that, among survivors of cancer treated during childhood and followed-up for 30 years, 8% had HF. The early detection of CTX induced by antineoplastic therapy seems to have a significant effect on the control of outcomes and on the hemodynamic effects resulting from myocardial injury. The identification of the best method for early detection of CTX in those patients is extremely important. Echocardiography and cardiac MRI are the most recommended tests by the main international guidelines and studies. ^28^^,^^227^^,^^239^

Despite the lack of randomized controlled studies in children and adolescents, early detection of subclinical lesions is of fundamental importance to stop their progression, which will then allow for prevention or reversal of LV pathological remodeling. ^228^

The American Society of Clinical Oncology (ASCO) proposes five clinical questions that will guide management planning and cardiovascular monitoring of survivors: ^6^

1. Which patients are at increased risk for developing cardiac dysfunction?
2. Which preventative strategies will minimize risk BEFORE initiation of therapy?
3. Which preventive strategies will minimize risk DURING potentially cardiotoxic therapy?
4. What are the preferred surveillance and monitoring approaches DURING treatment?
5. What are the preferred surveillance and monitoring approaches AFTER treatment?

Late follow-up planning based on risk stratification contributes to the choice of the best method for monitoring myocardial function (biomarkers and imaging tests). The aim is to detect subclinical dysfunction early, with consequent use of drugs that can prevent or reverse cardiac remodeling.

With regard to late follow-up after radiotherapy—which is one of the pillars of pediatric cancer treatment and about 40% of children, adolescents, and young adults undergo at some stage of the treatment— incidence and severity are directly proportional to the dose and irradiated volume and inversely proportional to age. Occurrence also increases with elapsed radiotherapy time and associated use of cardiotoxic agents, in particular anthracyclines, in addition to possible previous heart disease. ^34^^,^^234^ Radiation leads to diffuse interstitial myocardial fibrosis related to the irradiated area, especially of the LV anterior wall, and clinical manifestation will consist of restrictive cardiomyopathy. In view of the difficulty in assessing diastolic function in children, each patient’s follow-up values must be compared to baseline values. Endothelial damage favors early development of atherosclerotic plaques complicated by hemorrhage and inflammation with risk of rupture and thrombosis. The pericardium is the main target of injury usually initiated by fibrinous pericarditis and pericardial effusion, followed by fibrous thickening, preferably in the parietal pericardium and RV, and may extend to the mediastinum. Valve involvement is less frequent and, unlike pericardial involvement, affects the left side of the heart more often. The cusps become thickened and may calcify and lead to insufficiency and stenosis, especially of the mitral and aortic valves, sparing the tips of the cusps and the valve commissures. ^244^

Survivors of cancer treatment in childhood and adolescence are more vulnerable to the development of premature CAD, including in subclinical stages, with a risk of myocardial infarction 2 to 8 times greater than that of the general population. As coronary events are rare in younger patients, even in those at increased risk, pharmacological stress echocardiography and NM imaging tests to assess myocardial perfusion and viability are recommended. ^245^

Late follow-up of individuals without ventricular dysfunction should be performed routinely after 6 months to 1 year and then periodically, given that the incidence of HF related to the use of anthracycline and radiotherapy increases with late monitoring; the condition may appear after 15 years or more. Although the ideal frequency for assessing LV structure and function in survivors exposed to cardiac radiation is not yet well established in consensus statements, the greater the number of risk factors, the greater the frequency that assessments should be conducted. ^246^

### 8.5. Pregnancy in Survivors of Childhood and Adolescent Cancer

With advances in cancer treatment and the remarkable number of survivors of childhood and adolescent cancer, more women reach the childbearing age, and many will choose to become pregnant. Some have undiagnosed subclinical lesions and others are undergoing treatment for HF or other late complications of antineoplastic treatment. Pregnancy is associated with substantial changes in the cardiovascular system, as there is an increased metabolic demand in the pregnant woman's heart. Marked increases in circulating blood volume contribute to an additional 30% to 40% in cardiac output in a relatively early period of pregnancy (20 to 24 weeks), and these changes will influence the interpretation of cardiovascular status. The consequences are tachycardia, relative anemia, and hypercoagulability phenomena. The risk of developing cardiac events during pregnancy in women previously exposed to anthracyclines and/or thoracic radiotherapy in childhood is not clearly known, with limited data available in the literature. ^247^^,^^248^

Physiological multivalve regurgitations, especially in the right heart chambers, due to chamber enlargement, annular dilation, as well as mild pericardial effusion, are frequent in late pregnancy and postpartum, and seem to be caused by hypervolemia resulting from this period.

Table 9 lists maternal physiological cardiovascular changes according to each trimester of pregnancy.

**Table 9:** Physiological echocardiographic changes during pregnancy

1^st^trimester		2^nd^trimester		2^nd^trimester		Postpartum	
↓	SVR	↓	SVR	↓	SVR GLS	↓	HR LV end-diastolic diameter LV mass CO
↑	HR LV end-diastolic diameter LV mass CO	↑	HR LV end-diastolic diameter LV mass CO	↑	HR LV end-diastolic diameter LV mass CO	↑	SVR GLS

SVR: systemic vascular resistance; HR: heart rate; LV: left ventricle; GLS: global longitudinal strain; CO: cardiac output; ↑ = increases; ↓ = decreases.

#### 8.5.1. Cardiac Outcomes in Pregnant Survivors of Childhood and Adolescent Cancer

Some clinical studies have evaluated cardiac outcomes in pregnant women previously exposed to cardiotoxic cancer therapy.

Van Dalen et al. ^247^ retrospectively evaluated 53 female survivors of childhood and adolescent cancer who had one or more children. Data on LV systolic function and other echocardiographic parameters were not available for analysis. HF diagnosis was defined based on signs and symptoms. No patient developed HF during pregnancy or within 5 months of delivery. ^247^

Hines et al. ^248^ administered a questionnaire to 847 female survivors of childhood and adolescent cancer who had at least one complete pregnancy. The authors found that most female survivors of childhood and adolescent cancer did not experience cardiac complications during or after delivery; however, pregnant women with a history of cardiotoxic therapy should be followed-up carefully during pregnancy, especially those with a history of anthracycline exposure and who have been diagnosed with previous or current subclinical or symptomatic cardiomyopathy. ^248^

In another study, 37 women who received doxorubicin as part of a chemotherapy protocol for a childhood neoplastic disorder were followed-up at the same center during pregnancy (72 gestations) and after delivery. The authors concluded that the pregnancy outcome in women who received doxorubicin for childhood malignancy is generally favorable. However, those with LV dysfunction before pregnancy should be considered at increased risk of a poor pregnancy outcome and further deterioration of myocardial function. ^249^

Thompson et al. ^250^ evaluated a cohort of 58 women who were treated with anthracyclines and/or thoracic radiotherapy before the age of 20 at the MD Anderson Cancer Center. The incidence of adverse cardiac events (defined as LVEF < 50% on at least two echocardiograms or CAD) was significantly higher in women who had at least one pregnancy (29%) compared to nulliparous controls matched for anthracycline dose and follow-up period (15%), with p < 0.05. Among the 58 pregnant women, nine were diagnosed with CVD during pregnancy and five were diagnosed after pregnancy. In this small cohort, the time from exposure to anthracyclines to the first pregnancy and the total anthracycline dose were associated with an increased risk of adverse cardiac events. In addition, pregnancy was associated with a 2.4-fold increase in the risk of developing adverse cardiac events (95% CI: 1.02-5.41, p = 0.045). ^250^

#### 8.5.2. Cardiovascular Monitoring Recommendation in Survivors of Childhood and Adolescent Cancer Wishing to Become Pregnant

Studies of non-cancer populations with preexisting cardiomyopathies have reported a high risk of cardiac decompensation due to changes in cardiac physiology during pregnancy. Pregnancy is not recommended in patients with cardiomyopathies with LVEF < 40%. ^251^

In survivors of childhood and adolescent cancer, there is limited evidence on cardiac monitoring during pregnancy. International guidelines recommend a cardiovascular evaluation before pregnancy and in the first trimester for all women who were treated during childhood with anthracyclines and/or thoracic radiotherapy. The Children's Oncology Group recommends performing an echocardiogram before and then periodically during pregnancy (especially during the third trimester), in addition to cardiac monitoring during labor and delivery in patients receiving anthracycline doses > 300 mg/m^2^, thoracic radiation doses > 30 Gy, and those who received both cancer treatments, i.e., anthracyclines and thoracic radiotherapy. ^246^^,^^251^

Recommendations are as follows:

- Echocardiographic assessment of cardiac function before pregnancy and then periodically, every trimester, in women who were treated with anthracyclines (especially at doses > 300 mg/m^2^) and/or thoracic radiotherapy (especially at doses > 30 Gy) with previous LVEF ≥ 50%.
- Echocardiographic assessment of cardiac function before pregnancy and in the last trimester of pregnancy, or when clinically indicated, in women who were treated with anthracyclines (doses < 300 mg/m^2^) and/or thoracic radiotherapy (doses < 30 Gy) with previous LVEF ≥ 50%.
- Echocardiographic assessment of cardiac function before pregnancy and then periodically, every trimester, or when clinically indicated, in addition to cardiac monitoring during labor and delivery in women with LVEF between 40% and 50%. Consider repeating the echocardiogram 1 month after delivery.
- Pregnancy is not recommended for patients with LVEF < 40%.

### 8.6. Predisposing Situations to Thrombotic Events Related to Childhood and Adolescent Cancer Treatment

#### 8.6.1. Intracardiac thrombus

Much of the chemotherapy treatment of patients with childhood and adolescent cancer is performed intravenously using long-term catheters, which increase the risk of thrombus or vegetation formation. In Also, the procoagulant properties of tumor cells favor tumor invasion and metastasis. ^252^

In the pediatric population, the mean incidence of cancer-associated thrombotic events is 8%, most frequently in acute leukemias, followed by sarcomas, lymphomas, acute myeloid leukemia, Wilms' tumor, neuroblastomas, and central nervous system tumors. Occurrence may reach 36.7% among leukemias and 16% among lymphomas (due to extrinsic mass compression in the mediastinum), solid tumors and central nervous system tumors. Among sarcomas, Ewing's is the one at most risk for thrombotic events, followed by rhabdomyosarcoma and osteosarcoma. ^253^

Several clinical conditions are related to the etiology of thrombotic events in children and adolescents, such as recent surgery, congenital heart disease, immobilization, trauma, nephrotic syndrome, use of oral contraceptives, congenital thrombophilia, and presence of a central venous catheter, which is the most important predisposing factor alone. ^252^ L-asparaginase, used in acute lymphoblastic leukemia protocols, may suppress natural anticoagulants, particularly antithrombin and plasminogen. Corticosteroid use may lead to activation of both factor VIII-von Willebrand factor complex and plasminogen activator inhibitor-1. The combination of L-asparaginase and corticosteroids is a high-risk factor for the development of thrombotic events, especially in the induction phase, when there is reduced ability to inhibit thrombin. ^253^

Pulmonary thromboembolism is much less reported among children and adolescents compared to adults. In a Canadian study, an incidence of 0.86 per 10,000 hospital admissions was demonstrated; however, there are limited data on long-term follow-up. ^254^

The genetic characteristics of the host also contribute to a greater vulnerability to thrombotic events. A deficiency in natural anticoagulants such as antithrombin, protein C, and protein S is the genetic factor most often related to thrombotic events in the pediatric population. ^252^

TTE is an important tool for the investigation of intracardiac thrombi and vegetations, being complemented by TEE, which is more sensitive. The use of microbubble contrast agents helps detect thrombi and differentiate avascular thrombus from vascularized tumors. Intracardiac thrombi are characterized by their hyperechogenic appearance and generally regular borders. MRI and CT angiography may be adjuvant for diagnostic clarification; however, it may remain difficult to differentiate thrombi from other cardiac tumors. A diagnosis of intracardiac thrombus results from a combination of image presentation, location, and clinical status. In uncertain cases, diagnosis is often made after a anticoagulation cycle and imaging reassessment. ^255^

#### 8.6.2. Central Venous Catheter

According to estimates, more than 5 million central venous catheters are implanted annually in the US. Children with cancer require intravenous administration of chemotherapy for a considerable amount of time. The use of central venous catheters after the 1980s improved the quality of life of these patients, but use is associated with mechanical, infectious, and thrombotic complications. The incidence of CRT in pediatric patients with cancer reported in recent years has ranged from 4.6% to 7%. ^252^^,^^256^

Echocardiogram has a key role in the evaluation of this device. The tip of the catheter should ideally be in the superior vena cava close to the junction with the RA to prevent arrhythmias and thrombus formation. The entire visible venous path should be evaluated, as well as the flow behavior close to the device, in the cardiac chambers, and in the valves. ^256^

#### 8.6.3. Differential Diagnosis of Intracardiac Mass

##### 8.6.3.1. Prominent Crista Terminalis

The crista terminalis is a fibromuscular tissue that demarcates the limits of the embryological venous sinus and the RA muscular wall. When prominent, it can be mistakenly diagnosed as an intracardiac mass. On echocardiography, it appears as a hyperechogenic image in the posterolateral portion of the RA. Identification avoids incorrect diagnosis of intracardiac thrombus.

##### 8.6.3.2. Mitral Annular Calcification

Mitral annular calcification is the deposition of calcium and fat throughout the fibrous annulus, most commonly in the posterior portion of the mitral annulus, with a hyperechogenic appearance. It may be the basis for the formation of thrombi or vegetations and exhibits mobile calcified components with potential for embolization.

##### 8.6.3.3. Infective Endocarditis

In patients undergoing cancer treatment with positive blood culture, infective endocarditis (IE) should be suspected, as these individuals are at high risk for hospital infections because of the number of hospitalizations and immunosuppression. TTE is mandatory in such cases. The investigation includes cardiac valves in search of signs of vegetation, abscess, or cardiac fistula, presence of new valve regurgitation, as well as the appearance of long-term intravenous devices. The specificity for diagnosis of endocardial vegetation using TTE is greater than 90%, but the sensitivity ranges from 62 to 79%, as images smaller than 2 to 3 mm may not be visible. TEE also has a specificity greater than 90%; however, it has a higher sensitivity for diagnosis of IE (> 80% to 90%).

Endocardial vegetations are characterized by very irregular shapes and chaotic movement that is independent from valve movement. They are usually located on the atrial side of atrioventricular valves and on the ventricular side of semilunar valves and are associated with the destruction of the valve apparatus, leading to valve regurgitation or abscess. Valve abscesses most commonly affect the aortic valve and may fistulate to the LV or the atrium. There is an increased risk for stroke and AMI in patients diagnosed with IE. The main agents responsible for stroke are endocardial vegetations with dimensions greater than 10 to 15 mm that affect more than one valve, have excessive mobility, and show positive blood culture for fungus, *Staphylococcus aureus* , and *Streptococcus bovis* .

##### 8.6.3.4. Nonbacterial Thrombotic Endocarditis

**Libman-Sacks endocarditis:** this consists of granular material with immune complexes and platelets, without bacteria; it usually has small dimensions (1 to 4 mm), is found at the same sites as endocarditis, but is usually less irregular and better defined and is not associated with valve destruction. These lesions are usually asymptomatic and are more common in patients diagnosed with lupus.

**Marantic endocarditis:** this is a type of noninfectious endocarditis associated with malignancy, especially solid metastatic carcinomas and adenocarcinomas of the lung, pancreas, stomach, and other adenocarcinomas. There are descriptions of association with myelodysplastic syndromes. Marantic vegetations are composed of fibrin and platelets and rarely lead to valve dysfunction; classically, they affect the atrial face of the mitral valve and the ventricular face of the aortic valve. They may embolize in more than 50% of cases. Echotexture, dimensions, and location are no different from those of IE. However, significant diffuse valve thickening is frequently observed, which may help in the diagnosis.

##### 8.6.3.5. Lambl Excrescences

Lambl excrescences are defined as filiform structures, less than 2 mm wide and 3 to 10 mm long, with undulating motion, located on the atrial face of the mitral valve and on the ventricular face of the aortic valve. They are not associated with thromboembolic events.

### 8.6. Cardiovascular Evaluation in Case of Bone Marrow Transplantation in Children and Adolescents

BMT, or hematopoietic stem cell transplantation, either autologous or allogeneic, plays an important role in the treatment of various neoplasms during childhood and adolescence. The most frequent ones are leukemias, some solid tumors, bone marrow aplasia, immunodeficiencies, and some inherited diseases that affect the hematopoietic system.

Autologous transplants are, in general, less susceptible to cardiovascular complications compared to allogeneic transplants. The incidence of cardiovascular complications increases according to the time of cure of these patients, reaching up to 17% after 15 years of treatment. BMT survivors are at a 13-fold higher risk for cardiovascular complications compared to their siblings. ^257^ The following factors favor CTX after BMT:

- Age at BMT;
- Anterior cardiotoxic chemotherapy (e.g., anthracyclines);
- Myeloablative conditioning regimen (e.g., cyclophosphamide);
- Prior chest irradiation;
- Total body irradiation;
- Type of transplant;
- Presence of graft-versus-host disease (GvHD);
- Comorbidities (e.g., hypertension, DM, dyslipidemia, smoking, obesity).

GvHD is the most common complication in allogeneic BMT and requires regular assessments using cardiovascular imaging methods. It triggers immune responses that lead to endothelial damage and chronic inflammatory processes, affects several organs, including the lungs, and may simulate pulmonary arterial hypertension. It favors the development of vasculitis, early atherosclerosis, hypertension, cerebrovascular diseases, angina, and AMI. ^258^ Prolonged use of corticosteroids and other immunosuppressants in GvHD treatment accelerates those events, and presence of conventional cardiovascular risk factors contributes to the appearance of these complications. Cardiac involvement alone in GvHD is rare and may manifest as pericarditis, arrhythmias, and CAD. ^257^

Another factor to be considered is the deposition of iron in the myocardium resulting from multiple blood transfusions, which may persist for years after BMT and is usually assessed by means of cardiac and liver MRI. ^259^

Survivors of BMT treated with anthracyclines and/or chest irradiation should be examined annually for signs of cardiovascular complications. Both systolic and diastolic cardiac function should be assessed using echocardiography, either annually or every 5 years, depending on dosage and age of exposure. They must be preventively advised regarding modifiable cardiovascular risk factors (hypertension, DM, dyslipidemia, smoking).

## List of Abbreviations

^18^ F-FDG: ^18^ F-fluorodeoxyglucose
AMI: Acute myocardial infarction
BMT: Bone marrow transplantation
CHD: Carcinoid heart disease
CTX: Cardiotoxicity
CVD: Cardiovascular disease
CRT: Catheter-related thrombosis
CT: Computed tomography
CAD: Coronary artery disease
DVT: Deep venous thrombosis
DM: Diabetes mellitus
ECV: Extracellular volume
GLS: Global longitudinal strain
GvHD: Graft-versus-host disease
HF: Heart failure
IE: Infective endocarditis
IMT: Intima-media thickness
LA: Left atrium/atrial
LV: Left ventricle/ventricular
LVCR: Left ventricular contractile reserve
LVEF: Left ventricular ejection fraction
AL amyloidosis: Light-chain amyloidosis
MRI: Magnetic resonance imaging
SUV_max_: Maximum standardized uptake value
NM: Nuclear medicine
PCT: Primary cardiac tumor
RICAD: Radiation-induced coronary artery disease
RIHD: Radiation-induced heart disease
RIVD: Radiation-induced valve disease
RA: Right atrium/atrial
RV: Right ventricle/ventricular
TTS: Takotsubo syndrome
^99m^Tc: Technetium-99m
TEE: Transesophageal echocardiography
TTE: Transthoracic echocardiography
ATTR amyloidosis: Transthyretin amyloidosis
Top2: Topoisomerase 2
VTE: Venous thromboembolism

## APPENDIX 1 SUGGESTED ECHOCARDIOGRAM REPORT FOR CARDIO-ONCOLOGY

- **Patient identification:**
  - Name:
  - Hospital registration/personal ID:
  - Sex:
  - Date of birth: // (… years old)
- **Cancer diagnosis:**
- **Comorbidities:**
- **Treatment:**
  Date of start of treatment: / /
  Date of end of treatment: / /
  Treatment protocol (drugs used):
  Cumulative anthracycline dose: mg/m^2^
  Thoracic radiotherapy: Gy
  Other medications being used:
- **Timing of examination:**
  □ Baseline examination
  □ Intermediate examination (routine). Delta chemo: ……. days
  □ Examination due to complications. Describe: ….
  □ Examination at the end of treatment.
  □ Examination after treatment: ….. months / … years
  □ Date of last examination: / /
- **Anthropometric data:**
  Weight: kg Height: m BSA: m^2^BMI:
- **Vital signs:**
  HR: bpm BP: X mm Hg RR: bpm Temp.: °
- **Blood volume status:**
  □ Hypervolemia
  □ Dehydration
- **Conventional two-dimensional echocardiographic measurements:** (…)
- **LVd volume: mL LVs volume: mL**
- **LV mass: g LV mass index: g/m2**
- Cardiac anatomy: (sequential description)
- **Valve devices: (structural description, mobility, Doppler)**
- **Estimated pulmonary artery systolic pressure: (mm Hg)**
- **Parameters for diastolic function assessment:**
  - E/A:
  - Septal E/e’: cm/s
  - Lateral E/e’: cm/s
  - S: cm/s
  - LAVI: mL/m^2^
  - LA GLS:
  - Others?
- **Parameters for systolic function assessment:**
  - LVEF (Simpson biplane method):
  - Ejection fraction by 3D imaging:
  - LV global longitudinal strain (GLS):
  - Changes in segmental contractility:
  - RV global contractility (qualitative analysis):
  - RVEF (Simpson):
  - RV global longitudinal strain (GLS)
  - Other?
- **Pericardium:**
- **Conclusions:**
- **Functional comparative analysis:**
  - Decrease in LVEF from baseline:
  - Decrease in GLS from baseline:
- **Observations:**

## APPENDIX 2 IMPORTANT CONSIDERATIONS IN THE EVALUATION OF ECHOCARDIOGRAM REPORT IN CARDIO-ONCOLOGY

1. Echocardiography should always be performed before any hematology-oncology treatment to record the patient's initial functional status and identify preexisting heart disease. These data will serve as a comparative basis for subsequent examinations.
2. Before the next cycle of chemotherapy, echocardiographic tests should ideally be performed 3 weeks after the last infusion or as close as possible to the next.
3. Intermediate examinations are recommended according to the treatment protocol (cardiotoxic potential of the chemotherapeutic drugs used) or stratification of individual cardiovascular risk.
4. Consider the patient's blood volume status (preload and afterload) as it may influence the actual strain values.
5. Evaluations in case of complications that compromise the patient's hemodynamic status will serve as isolated information. The examination must be repeated after improvement of the patient’s clinical symptoms.
6. In case of detected dysfunctions (decreased LVEF and/or GLS or changes in diastolic function), the examination must be repeated after 3 weeks for confirmation of suspected condition or interpretation as a transient condition that will require future consideration.
7. In case of decreased LVEF and/or GLS when using known noncardiotoxic drugs, other causes for myocardial dysfunction must be considered (e.g., infection, CAD).
8. A good ECG tracing must be obtained for adequate strain quantification.
9. Use adequate echocardiographic windows whenever possible, avoiding image shortening as it leads to overvalued myocardial deformation indices.
10. Ideally, the same examiner should assess the patient during the entire follow-up.
11. When assessing a patient, consider using equipment of the same manufacturer during the entire follow-up.
12. GLS should not be performed in patients with cardiac arrhythmia (atrial fibrillation, severe tachycardia, and atrioventricular block).

## APPENDIX 3 WARNING SIGNS DURING ECHOCARDIOGRAM EVALUATION IN CARDIO-ONCOLOGY

**Table:**

Favorable	Attention	Caution	Unfavorable

- Normal LVEF + normal GLS + Troponin (-)
- Normal LVEF + normal GLS + type I diastolic dysfunction
  = transient change? Reassess situation during the next elective examination.
  Changes in segmental contractility + normal LVEF + normal GLS
  = Subclinical dysfunction? Transient change?
  = Reassess situation during the next elective examination.

- LVEF in the lower limit of normal in tachycardic patient.
- Normal LVEF + GLS < 16% or > 15% decrease from baseline GLS
  = Subclinical dysfunction? Consider cardioprotective measures.

- Abnormal LVEF + GLS < 16% or > 15% decrease from baseline GLS + troponin (+)
  = Discuss risks/benefits of chemotherapy regimen + cardiovascular treatment.
  = Repeat echocardiogram soon.

- Abnormal LVEF (< 40%)
  = Discuss temporary cessation of chemotherapy regimen + cardiovascular treatment.
  = Repeat echocardiogram soon.
- Pulmonary hypertension with right ventricle involvement.
- Cardiac tamponade.
